# Longitudinal Associations between Monetary Value of the Diet, DASH Diet Score and the Allostatic Load among Middle-Aged Urban Adults

**DOI:** 10.3390/nu11102360

**Published:** 2019-10-03

**Authors:** May A. Beydoun, Amelie Nkodo, Marie T. Fanelli-Kuczmarski, Ana I. Maldonado, Hind A. Beydoun, Barry M. Popkin, Michele K. Evans, Alan B. Zonderman

**Affiliations:** 1 Laboratory of Epidemiology and Population Sciences, National Institute on Aging, Intramural Research Program, NIA/NIH/IRP, Baltimore, MD 21224, USA; ankodo1@gmail.com (A.N.); ana.maldonado@nih.gov (A.I.M.); EvansM@grc.nia.nih.gov (M.K.E.); zondermana@mail.nih.gov (A.B.Z.); 2 Department of Behavioral Health and Nutrition, University of Delaware, Newark, DE 19717, USA; mfk@udel.edu; 3 Department of Research Programs, Fort Belvoir Community Hospital, Fort Belvoir, VA 22060, USA; hindb1972@gmail.com; 4 Department of Nutrition and Carolina Population Center, University of North Carolina at Chapel Hill, Chapel Hill, NC 27517, USA; popkin@unc.edu

**Keywords:** monetary value of diet, DASH diet, allostatic load, urban adults

## Abstract

Lower cost can lead to poorer-quality diets, potentially worsening metabolic profiles. We explored these pathways among urban adults. Longitudinal data were extracted from 1224–1479 participants in the Healthy Aging in Neighborhoods of Diversity across the Life Span (HANDLS) study. DASH_(mean)_ (Dietary Approaches to Stop Hypertension) score was computed using four 24 h recalls (v1/v2: 2004–2013) linked with a national food price database to estimate monetary value of the diet [MVD_(mean)_]. Allostatic load (AL) was measured at visits 2 (v2) and 3 (v3) in 2009–2018. Mixed-effects regression and structural equation modeling (SEM) were conducted, linking MVD_(mean)_/DASH_(mean)_ to AL [v2 and annual change(v3–v2)] and exploring mediating pathways between MVD_(mean)_ and AL_(v3)_ through DASH_(mean)_, stratifying by sex, race and poverty status. MVD_(mean)_ tertiles were linearly associated with contemporaneous DASH_(mean)_, after energy adjustment. In mixed-effects regression models, DASH_(mean)_ was consistently linked to lower AL_(v2)_. DASH_(mean)_ and MVD_(mean)_ were positively associated with higher serum albumin_(v2)_. In SEM, MVD_(mean)_ was linked to AL_(v3)_ through DASH_(mean)_, mainly among Whites and specifically for the cholesterol and Waist-Hip-Ratio AL components. In summary, energy and other covariate-adjusted increase in MVD may have a sizeable impact on DASH which can reduce follow-up AL among urban White middle-aged adults. More studies are needed to replicate findings in comparable samples of urban adults.

## 1. Introduction 

Dietary patterns are important predictors of allostatic load (AL), an indicator of multisystem physiological dysregulation over the life course [1]. The Dietary Guidelines for Americans (DGA) detail US federal recommendations for optimal quality of the total diet, while the Healthy People objectives set benchmarks for the everyday application of these guidelines [2]. The objectives outlined in the Healthy People 2020 and the Dietary Guidelines for Americans [3] provide evidence-based information on healthfulness of diets, including the DASH (Dietary Approaches to Stop Hypertension) Eating Plan [4]. Thus, various indices have been developed to measure adherence to the DASH dietary pattern [5]. The DASH diet [6,7] has been shown to improve or reduce cardiovascular [7], metabolic [8,9] and inflammatory [10] components of AL. Despite this evidence, the DASH diet has not been widely adopted amongst US individuals with hypertension [11]

One of the reasons the DASH diet has not been adopted is financial constraints, which determine food expenditure relative to other portions of household expenditures [12]. Indeed, most studies indicate a direct relationship between the monetary value of the diet (MVD) and diet quality (or an inverse relationship with consumption of energy-dense foods) [13,14,15,16,17,18,19,20,21,22,23,24,25,26,27,28,29,30,31,32,33,34,35,36,37,38,39,40,41,42,43,44] however this finding was not replicated in other studies [45,46,47,48,49]. In fact, accordance with the DASH dietary pattern is associated with higher dietary costs, [50,51] and higher income level; [51] while higher socioeconomic status (SES) retail environments are associated with greater availability of DASH foods [52]. This was substantiated by a review of the literature indicating an inverse association between SES and AL, which was measured with heterogenous biomarkers [53,54,55].

Within National Health and Nutrition Examination Survey (NHANES) data, poverty status, lower education, lower income gradients, and inferior neighborhood socioeconomic status were associated with increasing AL [53]. Based on research suggesting that, firstly, there is a direct link between SES (or MVD) and DASH diet adherence, and second, an inverse association between DASH diet and AL, healthy dietary patterns (such as DASH diet) may mediate the pathway from low SES to increased AL [56]. To our knowledge, the present study is the first to examine the net association between cumulative exposure to MVD and DASH diet scores over five years and rates of change and follow-up AL across sex, race and poverty status, using data from a large sample of low-income urban US adults, the Healthy Aging in Neighborhoods of Diversity across the Life Span (HANDLS). We hypothesize that the association between MVD and AL will be mediated by the DASH diet score.

## 2. Materials and Methods

### 2.1. Database

HANDLS is an ongoing prospective cohort study initiated in 2004. It focuses primarily on disparities in cardiovascular and cognitive health of a socioeconomically diverse sample of Whites and African-Americans aged 30–64 y at baseline and living in selected neighborhoods of Baltimore, Maryland. In brief, HANDLS used an area probability sampling strategy of thirteen neighborhoods, with details provided elsewhere [57]. Phase 1 of the baseline visit conducted between 2004 and 2009 [also known as (aka) visit 1] consisted of screening followed by recruitment, household interviews, a first 24 h dietary recall, while phase 2 of this baseline visit (also 2004–2009) consisted of the second 24 h dietary recall and in-depth examinations in mobile Medical Research Vehicles (MRV), including measurements of blood pressure, anthropometrics and a fasting blood draw among others. Two 24-h recalls were also collected at the follow-up visit [aka visit 2: 2009–2013] as were most of the measurements on the MRV. The final outcome, AL was measured at visits 2 and 3 (2009–2013 and 2013–2018), using at each visit a combination of blood pressure, anthropometric and laboratory data. All clinical laboratory indices were obtained at the Quest Diagnostics (Chantilly, VA, USA). Mean follow-up time (years) ± SD was 4.19 ± 1.47 (range: 0.58–7.45) between visits 2 and 3, among participants with complete dietary data at visits 1 and 2 and complete AL data at visit 3.

Participants provided written informed consent after reviewing a protocol booklet written in layman’s terms and watching a video detailing all procedures and future re-contacts. HANDLS study was approved ethically by the Institutional Review Board of the National Institutes of Health, National Institute of Environmental Health Sciences (NIEHS/NIH).

### 2.2. Study Sample

Of the original HANDLS sample selected at visit 1 (N = 3720), 2177 had complete data on two 24-h dietary recalls at baseline collected both at Phase 1 (household visit), and Phase 2 (MRV visit). At visit 2, N = 2140 completed two 24 h dietary recalls, and for both visits 1 and 2, N = 1516. From these dietary data, DASH diet score and MVD were estimated, as means between visits 1 and 2. No further exclusions were made for mixed-effects regression models with AL as the outcome (i.e., complete at visits 2 or 3), whereby a sample of N = 1516 was available. For SEM models, whereby AL at visit 3 was the final outcome, data was available on N = 1252. Accounting for missing data on covariates, the final analytic sample for SEM models consisted of N = 1224, while for mixed models, it consisted of 1479 participants and 2703 observations (Figure S1).

### 2.3. Dietary Assessment

At each of two visits (1 and 2), two 24-h dietary recalls were collected using the US Department of Agriculture (USDA) Automated Multiple Pass Method, a well-established computerized structured interview [58]. Several measurement aids were utilized, including measuring cups, spoons, rulers, and an illustrated Food Model Booklet which allowed participants to report accurate food and beverage quantities that were consumed. During the visit 1 study period (2004–2009), both recalls were administered in person by trained interviewer, 4 to 10 days apart. During the visit 2 study period (2009–2013), the second 24 h recall was administered by telephone interview, whereby participants had a copy of the illustrated food model booklet, while the first was conducted during the MRV visit. Trained nutrition professionals coded the dietary recalls using the Survey Net statistical software, [59] in order to match foods consumed with 8-digit codes identified in the Food and Nutrient Database for Dietary Studies (FNDDS) version 3.0 for baseline visit 1 and version 5 for the follow-up visit 2 [60].

### 2.4. Key Outcome Measure

A total AL score was computed using a method described in a previous study (Method S1) [1]. AL total score components are cardiovascular (systolic and diastolic blood pressure, pulse rate), metabolic (total cholesterol, high-density lipoprotein-cholesterol (HDL-C), glycosylated Hb, sex-specific waist-to-hip ratio) and inflammatory [albumin and high-sensitivity C-reactive protein (hsCRP)] risk indicators. The clinical criteria used for each risk indicator is summarized in Table 1 of Method S1. Indicators were summed with equal weighting to compute total AL score (range: 0–9). Contract laboratories measured total cholesterol (mg/dL), HDL-cholesterol (mg/dL), CRP (mg/dL), albumin (g/dL) and glycosylated hemoglobin (%) using reference analytical methods. Trained examiners measured waist-to-hip ratio, radial pulse (beats/min), and systolic and diastolic blood pressure (mmHg) using standard protocols. Specifically, blood pressure was measured using a mercury sphygmomanometer [61]. The arithmetic mean of three systolic and diastolic pressures was used in the analysis. AL at visits 2, 3 and means across both visits were estimated.

### 2.5. Key Exposure Measures

#### 2.5.1. Dietary Approaches to Stop Hypertension (DASH)

The score for DASH diet adherence was determined for each participant using the formula reported by Mellen et al. [11]. The DASH score is subdivided into nine target nutrients, specifically total fat, saturated fat, protein, fiber, cholesterol, calcium, magnesium, sodium and potassium. Micronutrient goals were expressed per 1000 kcal. The total DASH score was generated by the sum of all nutrient targets met: a value 1 was assigned if the participant achieved the DASH target for a nutrient, a value of 0.5 was achieved if the intermediate target was achieved, and a value of zero was assigned if neither target was met. DASH adherence (not considered in this study) was defined by a total score ≥4.5 out of 9 [11]. Those estimates were subsequently averaged to obtain the mean DASH total and component scores for both days combined for each of two visits: DASH_(mean)_ = [DASH_(v1)_ + DASH_(v2)_]/2.

#### 2.5.2. Monetary Value of Diet (MVD) Estimation

The Global Food Research Program at University of North Carolina’s (UNC) Packaged Food Purchase and Price Database, 2004–2013, provides average national and market-specific prices of ~3700 foods in “as-purchased” form per quarter [62,63]. The database was generated by linking food and beverage purchase data from the Nielsen Homescan Consumer Panel to Nutrition Facts Panel data from various sources including the Mintel Global New Products Database [64,65]. The detailed design of the Homescan study is provided in Method S2. Products were categorized at the barcode-level into the 34 foods and 8 beverages UNC Homescan groups based on nutritional content and consumption patterns; using a methodology previously described [66]. Market-specific price per 100 g for each food group in each quarter was calculated by dividing the survey-weighted dollars spent by weighted volume of purchases for all products in a given food group by households in that market during the quarter [62]. To account for inflation and allow comparability over time, the average real prices of each food group for all households was scaled to the first quarter of 2004. The 8-digit codes in the Food and Nutrient Database for Dietary Studies (FNDDS) versions 4.1, 5.0 and 2011–2012 reported by NHANES participants from stores or vending in 2007–2008 to 2011–2012 were matched to UNC Homescan food groups to generate food prices per 100 g at the national level for about 3700 FNDDS food codes from 1,934,441 barcoded products (814,481 unique Nutrition Facts Panel records).

The linkage between NHANES FNDDS data and the UNC Homescan food groups facilitated the calculation of MVD per day estimates from HANDLS FNDDS grouping. To this end, a team of trained RD-MPH fulltime staff did this linkage over a several years period with Robert Wood Johnson Foundation and later using NIH funding. This involved reconstituting and cooking many foods and doing other changes to shift as purchased to as consumed. It also involved thousands of hours in work to link the foods with the correct FCT number. Details on the method are found in this paper [67]. Due to the limited linkage between Homescan food prices with NHANES 2007–2008 and 2011–2012, half of the remaining FNDDS codes reported by HANDLS participants were imputed by matching computed food groups for HANDLS (60 food and beverage groups) with 42 food and beverage groups computed by UNC. For the remaining codes without a match, the nearest neighbor code was used for imputation. The result was the harmonization of all HANDLS FNDDS food codes into one of the 42 UNC food groups linked to a singular food price, given the year and quarter in which they were reported. The imputation techniques were validated by the similar prevalence of high rank food groups in both imputed and non-imputed observations (Method S3). Within individual HANDLS ID, total cost was measured by adding individual food prices given the amount consumed per food code per recall. The estimated MVD per individual across visits was calculated as average MVD across the four recalls: MVD_(mean)_ = [MVD_(v1)_ + MVD_(v2)_]/2.

### 2.6. Covariates

The baseline or fixed covariates considered as potential confounders and/or effect modifiers included age, sex, race (White vs. African American), completed years of education (<High School (HS); HS and >HS), literacy [Wide Range Achievement Test, third version (WRAT-3) total score], poverty status, a design-based binary variable in HANDLS based on poverty income ratio (PIR < 125%: below poverty; PIR ≥ 125%: above poverty), current tobacco smoking status (0: “never or former smoker” and 1 “current smoker”) and current drug use (0:”never or former drug user and 1 “current drug user”). Illicit drugs included in this measure were marijuana, opiates and coke. Current drug use had a time frame of 6 months or less. The reading subtest of the Wide Range Achievement Test-3rd Edition (WRAT-3), a widely validated measure of literacy, assessed participants’ ability to recognize and name letters and words, with a total score computed as “total correctly pronounced letters + total correctly pronounced words” [68]. Other baseline covariates included employment status, body mass index (weight/squared-height, kg·m^−2^) and self-rated health (0: Poor/fair, 1: Good, 2: Very good/excellent). All these covariates have been shown to be associated with dietary quality, including elements of the DASH score, MVD and several metabolic disturbances included in the AL measure [38,67,69,70,71]. Since the MVDs were based on estimated prices of foods as sold in grocery stores and prepared at home rather than away-from-home setting, all models were adjusted for the % of energy consumed at home. Additionally, energy intake (kcal/day) from total diet was adjusted for in all regression models, including mixed-effects and SEM models. The mean values across the first two visits were used rather than baseline values for energy intakes (total and from grocery stores).

### 2.7. Data Handling and Statistical Analysis

Stata release 15.0 (StataCorp, College Station, TX, USA) was used to complete all statistical analyses [72]. First, study sample characteristics were assessed by tertiles of mean MVD (MVD_(mean)_ tertile). To test linear trend relationship between MVD tertiles and continuous characteristics, a bivariate ordinary least square (OLS) regression was used with MVD entered as an ordinal predictor of each continuous variable of interest. Associations between categorical study characteristics and MVD tertiles were evaluated with χ^2^ tests. Second, linear regression models were conducted to test associations between MVD_(mean)_ and DASH total scores across socio-demographic groups, with two models presented: Model 1 (Crude); Model 2 [adjusted for total caloric intake: (kcal_v1_ + kcal_v2_)/2]. Third, multiple linear mixed-effects regression models were conducted to test associations between MVD_(mean)_, DASH_(mean)_ and longitudinal change in the AL between first and second follow-up, adjusting for baseline and fixed characteristics that are listed in the Covariates section, including socio-demographic, lifestyle and health-related factors. Each model included *TIME* [set at zero for visit 2 AL, and time elapsed to visit 3 AL] and 2-way interaction terms between *TIME* and key exposures (MVD and DASH scores) and between *TIME* and each of the covariates. Those interaction terms are interpreted as the effects of exposures and covariates on the slope or annual rate of change in the AL (between 2009–2013 and 2013–2018). Main effects of exposures and covariates were also included in each model and are interpreted as effects of those variables on baseline outcome, in this case AL at visit 2. To ease interpretation of the intercept, continuous exposures and covariates were centered at their mean. Repeated outcome measures ranged between 1 and 2, with a mean of 1.8 visits per participant. We assumed the unavailability of outcomes to be missing at random (Method S4) [73]. In the overall sample, three models were ran: Model 1 (Both MVD and DASH), Model 2 (MVD alone) and Model 3 (DASH alone). In addition to the overall models, mixed-effects regression models were stratified separately by sex, race and poverty status. Heterogeneity in the effect of exposure on change in outcome was formally tested by adding 2-way and 3-way interaction terms between sex/race/poverty status, *TIME* and exposure (MVD/DASH).

AL at visit 3 was considered as an endogenous variable that was potentially associated with both MVD_(mean)_ and DASH_(mean)_. To test mediation, two methods were used. First, structural equations models (SEM) were carried out whereby MVD_(mean)_, socio-demographic, lifestyle and health-related factors (see covariates section) were exogenous to DASH_(mean)_ and AL (see Equations (1)–(3).

(1) $$zMVD\left(mean\right)\;=\;\overset{k}{\underset{i\;=\;1}{\sum }}\alpha_{Z_{i_{1i}}}Z_{i}+e_{1}$$

(2) $$zDASH\left(mean\right)\;=\;\alpha_{21}{}_{}zMVD\left(mean\right)+\overset{k}{\underset{i\;=\;1}{\sum }}\alpha_{Z_{i_{2}}}Z_{i}+e_{2}$$

(3) $$zAL\left(v3\right)\;=\;\alpha_{31}{}_{}X_{}+\alpha_{32}zDASH\left(mean\right)+\overset{k}{\underset{i\;=\;1}{\sum }}\alpha_{Z_{i_{3}}}Z_{i}+e_{3}$$

where zMVD_(mean)_ is the standardized score of mean MVD between visits 1 and 2, zAL_(v3)_ stands for AL at visit 3 (also as standardized z-score), zDASH_(mean)_ is standardized z-score for mean DASH scores between visits 1 and 2, and i is the number of covariate terms included, Z_i_ is a vector of socio-demographic, lifestyle and health-related variables (see Covariates section). Based on Equations (1)–(3): α_31_ = direct effect; α_21_ × α_32_ = indirect effect; total effect = α_31_ + α_21_ × α_32_ [67,74].

Second, when relaxing the assumption of additivity (RAA) between zMVD_(mean)_ and the zDASH_(mean)_ score by including an interaction term, we further computed four estimates with their SEE and *p*-values, namely the controlled direct effect (CDE), the natural direct effect (NDE), the natural indirect effect (NIE) and the marginal total effect (MTE). Details about this latter approach are provided elsewhere [75]. The CDE is the effect of setting X [i.e., zMVD_(mean)_] to 1 versus 0 (i.e., 1 SD higher than the mean vs. the mean) while controlling M to some defined reference value *m*. In this case, M is the continuous zDASH_(mean)_ which is set at a value close to the mean, namely zero. The NDE is the same setting of the exposure X, but this time M (zMVD_(mean)_) is set not to a single pre-defined value m, but instead a value that is potentially distinct for every person in the data set. It is the value that m would have taken at the referent value of the exposure (in this case, the exposure level that is at the mean). The NIE is the outcome contrast observed when holding exposure X [i.e., MVD_(mean)_] constant at its mean, and contrasting two different M [DASH_(mean)_] values: the value of the DASH_(mean)_ score that would be observed for that person under the X value [MVD_(mean)_] of the population mean and the value of DASH that would be observed for that person under the 1 SD higher X value. The total effect is the sum of the NIE and the NDE. It is the total effect of varying X by 1 SD, irrespective of M (or the DASH score) [73]. In both methods, mediation was determined by the statistical significance and direction of the indirect effect at a type I error of 0.05 and was interpreted based on the direction of that indirect effect (+ or −). It is expected that MVD would have an inverse association with AL through DASH. A sensitivity analysis was conducted for each DASH_(mean)_ component with the final outcome being AL_(v3)_. No RAA was assumed in these models and thus interaction terms were excluded. All models were conducted overall, and stratified separately by sex, race, and poverty status.

The non-random selection of participants with complete data from the target study population can often lead to selection bias. To account for this type of bias, a 2-stage Heckman selection model was constructed, [76] using a probit model to obtain an inverse Mills ratio at the first stage (derived from the predicted probability of being selected out of the sample with complete 24 h recalls at baseline (N = 2177, see Figure S1), conditional on the covariates in the probit model, mainly baseline age, sex, race, poverty status and education), as was done in earlier studies [77,78,79]. Specifically, participants included the final analytic sample used for the mixed-effects linear regression models (N = 1480) differed from the remaining sample of out of the initial N = 3720 by having a smaller proportion male (40% vs. 48%, *p* < 0.05) and a greater proportion with >HS education (36% vs. 27%, *p* < 0.05). Similar patterns of socio-demographic differences were noted when comparing the final analytic sample used for SEM models to the excluded group from the initial sample (N = 3720). 

Type I error was set at 0.05 for main effects and 0.10 for interaction terms due to the latter’s reduced statistical power compared to the former [80]. Methods S4 and S5 provide description of the mixed-effects regression models and the Stata syntax for the entire analysis, respectively. 

## 3. Results

### 3.1. Characteristics of Study Participants by MVD Tertiles

Table 1 presents crude sample characteristic distribution by tertiles of mean MVD between visits 1 and 2. Most notably, higher MVD was inversely related to the proportion African-American and to age at all visits, while being directly related to the proportion male. In addition, higher MVD was linked to higher SES in terms of poverty status, education and literacy and employment status. It was also directly linked to drug use, but inversely related to body mass index (BMI). The higher the MVD, the greater the total energy and energy consumed from grocery stores. Overall, no linear crude association was detected between MVD_(mean)_ tertiles and DASH total score (v1, v2 and mean) and between MVD_(mean)_ tertiles and AL measured at either visits (or mean).

### 3.2. DASH Diet Score by MVD Tertiles: Crude and Energy-Adjusted

When examining the stratum-specific association between MVD and DASH total score (means: v1/v2), in Table 2, we found a direct linear dose-response relationship after adjustment for mean energy intake. The crude model detected a positive association between MVD and total DASH score only among women (*p* < 0.05 for sex × MVD tertile interaction). 

### 3.3. DASH Diet Score, MVD and Allostatic Load (AL) over Time: Mixed-Effects Regression Models

The mixed models in Table 3 depict the cross-sectional and longitudinal associations of MVD and DASH total score (mean, v1/v2) with AL and its components (measured repeatedly at v2 and 3). Overall, a better dietary quality (higher DASH score) was associated with a lower baseline AL, after adjusting for all potential confounders, including MVD, whose direct association with AL was a positive one in Model 1. The total effect of MVD on baseline AL (Model 2) was not detectable, while the DASH diet score was inversely related to AL (Model 3) without adjustment for MVD. For components of AL, MVD was positively associated with baseline albumin levels, reflecting lower AL, overall, among African-Americans and among individuals living above poverty. A higher DASH score in the total sample was also directly associated with baseline serum albumin, reflecting lower AL. While DASH total score was linked to lower HbA1c among men and slower increase in HbA1c over time among women, the results were not indicative of a protective direct effect of MVD on this outcome. Heterogeneity by sex, race and poverty status was found for both cross-sectional and longitudinal associations of MVD/DASH with AL and its components.

### 3.4. DASH Diet Score as Mediator between MVD and AL at Last Visit: Relaxing the Assumption of Additivity (RAA) and Structural Equation Modeling (SEM) Mediation Models

Using a mediation model (RAA, Table 4), we found that only among Whites, the natural indirect effect was statistically significant, indicating the existence of a pathway whereby an inverse relationship between MVD_(mean)_ and AL_(v3)_ was completely mediated through DASH_(mean)_. In the sensitivity analysis, this was consistently the case for the fiber, magnesium and potassium components of DASH (data not shown). Without RAA (Table 5), the results were very similar in the SEM models, stratified by socio-demographic factors, although the significant indirect effect was also found in the total sample. This result was mostly driven by a complete mediation of the inverse relationship between MVD and total serum cholesterol (CHOL) and MVD and WHR through a higher DASH diet score among Whites. 

## 4. Discussion

In this study, we examined the association between the monetary value of MVD, the DASH diet score and the AL using longitudinal and SEM techniques, while stratifying by key socio-demographic factors. Among our key findings, MVD_(mean)_ tertiles were linearly associated with contemporaneous DASH_(mean)_, after energy adjustment. In mixed-effects regression models, DASH_(mean)_ was consistently linked to lower baseline AL_(v2)_. Both DASH_(mean)_ and MVD_(mean)_ were positively associated with higher serum albumin_(v2)_. Using SEM, we found an independent pathway linking MVD_(mean)_ to AL_(v3)_ through DASH_(mean)_, mainly among Whites and specifically for the cholesterol and Waist-Hip-Ratio components of AL, which remained significant after relaxing the additivity assumption.

### 4.1. The Link between MVD and DASH Score or Other Diet Quality Indices

This study provides evidence of a direct linear response relationship between MVD and diet quality, assessed by DASH score, adjusting for energy intake. This relationship has been confirmed by other researchers using alternate measures of diet quality [20,26,31,37,38]. Energy-adjusted MVD (uppermost vs. lowest quintile) was associated with a 30 point higher Alternative Healthy Eating Index score in women enrolled in the US Nurses’ Health Study (N = 78,191) [26]. Using data from the NHANES 2001–2002 and 2007–2010 Rehm and colleagues corroborated findings of the direct association between MVD and dietary quality, measured by the Healthy Eating Index [HEI]-2005 and HEI-2010 indices [31,37]. Additionally, the highest tertile of the Mean Adequacy Ratio of French adults was associated with the lowest dietary energy density and the highest diet costs [20].

A meta-analysis of data from 24 studies from 10 countries revealed marked pricing differences between food groups, which increased the cost of healthful diets [44]. Darmon and Drewnowski found that healthier diets were uniformly associated with higher costs, with food budgets in poverty being insufficient to ensure optimum diets [39]. In fact, Rehm and colleagues found that lower dietary costs were associated with lower consumption of vegetables, fruits, whole grains, and seafood, and higher consumption of refined grains, solid fat, alcohol and added sugars [31,37]. Aggarwal and colleagues reported MVD to be directly associated with dietary intakes of fiber, vitamins A, C, D, E, and B-12, β-carotene, and folate, as well as the minerals -iron, calcium, potassium and magnesium. In contrast, they found an inverse relationship between MVD and intakes of saturated fats, trans fats, and added sugars [33]. 

### 4.2. The Link between DASH Score and AL or Other Metabolic Disturbance Indices

Previous studies have evaluated the relationship of DASH diet adherence with various cardiometabolic indicators and have uncovered for the most part a potential protective effect. Those indicators included weight status or BMI [9,50,75,76,77,78,79,81,82,83], waist circumference [9,75,77,78,79,81,82,84,85,86,87], hip circumference, [78,79] waist-to-hip ratio, [78,88] total cholesterol, [75,76,86,89,90,91] LDL-C, [75,76,79,86,88,90,91] HDL-C, [9,75,76,79,85,86,88,91,92] triglycerides, [9,75,76,79,85,86,88,92] HbA1c, [86,87] glucose, [76,85,87,88,90,93,94] blood pressure, [8,9,76,85,86,88,90,93,95,96,97,98,99,100,101] fibrinogen, [85] CRP, [9,79,85,102,103,104,105] IL-6, [79,103,105] IL-17A, [104] TNF-alpha, [79,105] insulin resistance, [79,92] type 2 diabetes biomarkers, [106] metabolic syndrome, [84,85,86,88,107,108] adipokines, [79,92] lipids [79,87,91,94,109], body composition, [81] stress hormones, [86] sex hormones [92] and cardiovascular risk factors [8,110,111].

A limited number of studies included examined the association between dietary factors with AL [56,86,112,113]. A cross-sectional study involving 3387 subjects by Rosenberg et al. assessed serum levels of α-carotene, β-carotene, β-cryptoxanthin, lutein/zeaxanthin and lycopene in relation to AL [112]. Serum β-carotene concentration was inversely associated with high AL after adjusting for age, education, race/ethnicity, serum cotinine, alcohol consumption, physical activity and other carotenoids (α-carotene, α-cryptoxanthin, lycopene, lutein/zeaxanthin) [112]. Another cross-sectional study of 1318 subjects by Mattei et al. evaluated AHA diet score in relation to AL as well as metabolic syndrome [86]. The study found associations of the diet-only score with insulin, waist circumference, and HDL-C [86]. The study also found associations between the continuous diet score and AL components, namely an inverse association with urinary cortisol and a positive association with serum dehydroepiandrosterone sulfate in women as well as an inverse association with urinary norepinephrine in men [86]. Every 10 AHA diet score units were associated with 22% (95% confidence interval (CI): 1, 38; *p* = 0.043) lower odds of having dysregulated AL components in women. In men, every 10 diet score units were associated with lower odds of MetS (odds ratio (OR): 0.69; 95%CI: 0.52, 0.93; *p* = 0.016) [86]. A cross-sectional study of 1002 older adults by Forrester et al. [113] examined smoking, poor diet, physical activity, alcohol use as predictors of four classes of biological indicators of AL from latent class analysis. The study found that Metabolic + BP class [(1) Metabolic + Cholesterol (high on these), (2) Metabolic + BP (high on these), (3) BP (high on this), (4) Low (on everything)] reported less physical activity and less alcohol use compared to the low class [113].

In a cross-sectional study involving 96 older adults, Kusano et al. examined dietary patterns in relation to AL [114]. For women, AL was lower when they ate more green/yellow vegetables (versus less) and when they ate more meat (versus less). For men, Al was higher for those who drank more (versus less) [114]. Another study of middle-aged adults by Kim et al. evaluated dietary patterns in relation to AL and found that, among males, there was a negative relationship between consuming/preferring fat in the diet and AL for BMIs in the second quartile (BMI > 30) compared to the first quartile [115]. There was also a negative relationship between appetite control and AL in individuals with BMI < 25 (1st quartile vs. 3rd quartile). However, there was a positive relationship between appetite control and AL among individuals with BMI 25–30. Finally, there was a positive relationship between eating due to food cues and AL among individuals with BMI 25–30 (1st quartile vs. 4th quartile) [115].

A cohort study involving 999 subjects, diet was evaluated as one of the mediators between socioeconomic position and AL [56]. The study found that negative behavioral and poorer material factors accounted for much of the association between higher socioeconomic position and lower AL; home ownership and low income, but not car ownership, attenuated the socioeconomic position–AL association by between approximately 60% and 80%. Smoking, but not alcohol consumption, poor diet or low physical activity, attenuated the socioeconomic position–AL association by a third. Adjustment for GHQ-12, a measure of psychological circumstances, had next to no attenuating effect [56].

### 4.3. Strengths, Limitations and Future Directions

Our study has many notable strengths, including its use of the DASH diet score in relation to MVD and AL using repeated measures over time, thus ascertaining temporality of relationships. Specifically, annual rates of change in AL (between visits 2 and 3) as well as AL at follow-up (visit 3) was tested against cumulative exposures over two visits (v1/v2) of data (visits 1 and 2), by taking their respective averages. Furthermore, it is among few studies to test those associations across race, sex and income groups. Although our study setting was Baltimore city, our findings can be generalized to comparable populations across the United States, mainly 14 cities of similar racial group composition. A key strength in estimating MVD in this study is the use of Baltimore city quarterly food prices as opposed to using US-wide estimates. Those estimates were additionally deflated to the first quarter of 2004 for comparability.

Nevertheless, given notable study limitations, our findings should be interpreted with caution. First, the use of food prices to compute MVD can underestimate total food expenditures [116]. Moreover, MVD may also be measured with error due to lack of specificity of some FNDDS food codes that did not necessarily reflect the exact match of foods as purchased; for example, a “burrito with chicken” can assume that the burritos were purchased refrigerated, frozen, or as a meal kit. Another notable limitation is that foods lacking barcodes, such as those unpackaged and sold by weight, were not found in the food price database; thus, prices of food groups to which those fresh produce and meats corresponded were weighted heavily on frozen and canned items. Furthermore, participants in the Homescan study do not report fast food, restaurants, and other away-from-home food purchases, with food prices differing markedly by source of purchase (i.e., grocery store vs. away-from-home sources). Moreover, imputations were conducted to estimate food price indices for certain FNDDS codes that could not be assigned to the 42 food groups. Finally, both the DASH diet score and the AL have been measured in multiple different ways in past studies. The selection of a method of measurement may have affected some of our key findings. More longitudinal studies are needed to replicate our findings in comparable samples of urban adults.

## 5. Conclusions

In this group of community dwelling urban adults, an energy-adjusted increase in MVD may have a sizeable impact on the DASH diet score which can then reduce the AL at follow-up, in the overall population of urban middle-aged adults, but more strongly among Whites and among women. This highlights the economic barriers behind improving dietary quality of a fixed caloric amount consumed, which in turn could improve metabolic outcomes by reducing the AL. The finding of a mediated effect between MVD and AL through DASH score, highlights the need to investigate this pathway further as well as conduct future randomized trials, while accounting for economic barriers. 

## Figures and Tables

**Table 1:** Study sample characteristics by tertile of mean monetary value of diet (MVD (mean), $/day), HANDLS 2004–2013 ^c^.

	MVD (mean) Tertiles ($/day) ^a^			
	T_1_	T_2_	T_3_	*p* ^b^
	(N = 416)	(N = 407)	(N = 401)	
Range, $/day:	3.83 ± 0.72	5.77 ± 0.56	9.16 ± 2.43	<0.001
Monetary value of diet and energy intakes				
Monetary value of diet at baseline, $/day (X ± SE)	3.8 ± 0.03	5.7 ± 0.03 *	9.2 ± 0.12 *	<0.001
Monetary value of diet at follow-up, $/day (X ± SE)	5.1 ± 0.08	6.0 ± 0.10 *	7.4 ± 0.13 *	<0.001
Monetary value of diet (mean), $/day (X ± SE) ^a^	3.8 ± 0.04	5.8 ± 0.03 *	9.2 ± 0.12 *	<0.001
Energy intake at baseline, kcal/day (X ± SE)	1324 ± 20	1904 ± 24 *	2820 ± 53 *	<0.001
Energy intake at follow-up, kcal/day (X ± SE)	1706 ± 31	2058 ± 36 *	2472 ± 51 *	<0.001
Energy intake (mean), kcal/day (X ± SE) ^a^	1515 ± 20	1981 ± 23 *	2646 ± 44 *	<0.001
Baseline socio-demographic and SES variables				
Sex, % male	23.3	37.1	57.6	<0.001
Age at baseline, yrs. (X ± SE)	48.6 ± 0.4	48.5 ± 0.5	47.0 ± 0.4 *	0.023
Age at first follow-up, yrs. (X ± SE)	53.3 ± 0.4	53.2 ± 0.5	51.8 ± 0.4 *	0.026
Age at second follow-up, yrs. (X ± SE)	57.4 ± 0.4	57.3 ± 0.5	56.1 ± 0.4	0.070
African-American, %	68.5	59.7	48.9	<0.001
Poverty status, % (<125% PIR)	43.8	39.6	37.4	0.170
Education, yrs. Completed, %				
<HS	7.2	6.1	5.7	0.003
HS	64.2	54.6	52.6	
>HS	28.4	39.3	41.7	
Literacy, WRAT-3 score				<0.001
<36, %	26.2	19.9	16.1	
37–40, %	18.5	14.9	12.7	
41–46,%	30.3	27.3	25.7	
≥47, %	25.0	37.8	45.4	
% Unemployed in last month, yes	37.9	29.7	28.2	0.033
% Unemployment in last month, missing	17.1	19.7	19.7	
Baseline drug and tobacco use				
Any drug, current user, %	40.9	42.0	53.9	0.001
Any drug, missing, %	7.5	8.9	7.5	
Tobacco, current user, %	41.6	36.6	41.7	0.529
Tobacco, missing, %	8.9	10.8	9.7	
Baseline body mass index, kg/m^2^ (X ± SE)	30.7 ± 0.4	30.5 ± 0.4	29.1 ± 0.4 *	0.006
Baseline self-rated health				0.873
Poor/Average, %	23.3	20.6	21.2	
Good, %	40.1	43.0	41.4	
Very good/Excellent %	36.5	36.4	37.4	
Baseline energy from grocery stores (X ± SE)	1031 ± 21	1436 ± 27 *	2175 ± 53 *	<0.001
Follow-up energy from grocery stores (X ± SE)	1297 ± 28	1542 ± 33 *	1905 ± 49 *	<0.001
Mean energy from grocery stores (X ± SE)	1164 ± 18	1489 ± 24 *	2040 ± 43 *	<0.001
DASH total score at baseline (X ± SE)	1.70 ± 0.06	1.68 ± 0.06	1.78 ± 0.07	0.62
DASH total score at first follow-up (X ± SE)	1.79 ± 0.06	1.69 ± 0.06	1.81 ± 0.06	0.30
DASH total score (mean) (X ± SE)	1.75 ± 0.04	1.68 ± 0.05	1.79 ± 0.06	0.33
AL at first follow-up (X ± SE)	1.97 ± 0.06	2.06 ± 0.06	1.87 ± 0.07	0.10
AL at second follow-up (X ± SE)	1.96 ± 0.06	2.03 ± 0.06	1.89 ± 0.06	0.26
AL annual rate of change (X ± SE)	0.000 ± 0.02	−0.020 ± 0.020	−0.004 ± 0.16	0.75
AL total (mean)	1.97 ± 0.05	2.04 ± 0.06	1.88 ± 0.06	0.095

Abbreviations: AL = Allostatic load; DASH = Dietary Approaches to Stop Hypertension; HANDLS = Healthy Aging in Neighborhood of Diversity across the Lifespan; HS = High school; MVD = Monetary value of the diet; PIR = Poverty income ratio; SE = standard error; T = tertile; WRAT-3 = Wide Range Achievement Test, 3rd revision; X = mean. ^a^ The monetary value of the diet (MVD) was estimated for each HANDLS visit using the HOMESCAN database at the annual and quarterly level for each food group. This was summed across individual dietary recall and averaged across individual participant in each visit. MVD is measured as mean across visits 1 and 2. This was similarly done for energy intake (kcal/day) and % energy from grocery stores. ^b^ *p*-value from one-way analysis of variance (ANOVA, continuous variables) or from χ^2^ test (categorical variables). * *p* < 0.05, post-hoc Bonferroni corrected *t*-test for null hypothesis of no between-tertile differences, taking T_1_ as the referent. ^c^ Researchers own analyses and calculations based in part on data reported by Nielsen through its Homescan Service for the food and beverage categories for the years 2004–2013, for the US market Nielsen data is licensed from The Nielsen Company, 2016 The conclusions drawn from the Nielsen data are those of the researchers and do not reflect the views of Nielsen. Nielsen is not responsible for and was not involved in analyzing and preparing the results reported herein.

**Table 2:** MVD (mean) tertiles as predictors of DASH (mean) total score, stratifying by sex, race and poverty status: multiple ordinary least square and logistic regression models, HANDLS 2004–2013 ^d^.

	Model 1: MVD (Mean) Tertiles ($/day) ^a^				Model 2: MVD (Mean) Tertiles ($/day) ^a^	
	β ± SE (T_2_ vs. T_1_)	β ± SE (T_3_ vs. T_1_)	P-Trend ^b^	β ± SE (T_2_ vs. T_1_)	β ± SE (T_3_ vs. T_1_)	P-Trend ^b^
DASH total score						
Overall	−0.07 ± 0.07	0.04 ± 0.07	0.57	+0.21 ± 0.07 **	+0.72 ± 0.09 ***	<0.001
Men	−0.13 ± 0.11	−0.11 ± 0.11	0.39	+0.06 ± 0.12	+0.34 ± 0.01 **	0.004
Women	−0.001 ± 0.09	+0.35 ± 0.11 **^,c^	0.003	+0.31 ± 0.09 **	+1.04 ± 0.12 ***	<0.001
Whites	−0.25 ± 0.13	0.09 ± 0.13	0.29	+0.03 ± 0.13	+0.68 ± 0.15 ***	<0.001
AA	−0.002 ± 0.08	−0.16 ± 0.09	0.094	+0.26 ± 0.08 **	+0.54 ± 0.11 ***	<0.001
Above poverty	0.02 ± 0.10	0.05 ± 0.09	0.62	+0.31 ± 0.10 **	+0.76 ± 0.11 ***	<0.001
Below poverty	−0.21 ± 0.11	0.02 ± 0.11	0.99	+0.05 ± 0.11	+0.65 ± 0.13 ***	<0.001

Abbreviations: DASH = Dietary Approaches to Stop Hypertension; HANDLS = Healthy Aging in Neighborhood of Diversity across the Lifespan; MVD = Monetary value of the diet, SE = Standard error. *** *p* < 0.001, ** *p* < 0.010, * *p* < 0.05 for null hypothesis that β = 0 (i.e., T_2_ vs. T_1_ and/or T_3_ vs. T_1_). ^a^ Values are regression coefficients and their standard errors (β ± SE) from a linear regression model with Y = mean DASH total score and the key predictor being tertile of mean MVD, contrasting the middle tertile with the lowest tertile (T_2_ vs. T_1_) and the uppermost tertile with the lowest tertile (T_3_ vs. T_1_). Model 1 is the crude association, while Model 2 further adjusted for energy intake (mean of visits 1 and 2). ^b^ P-trend was derived from a similar model as in a, but with the key predictor MVD tertiles entered as a single ordinal variable rather than two dummy variables. ^c^ P < 0.05 for null hypothesis that the term sex*MVD = 0 in a separate un-stratified regression model in which this interaction term was added. ^d^ Researchers own analyses and calculations based in part on data reported by Nielsen through its Homescan Service for the food and beverage categories for the years 2004–2013, for the US market Nielsen data is licensed from The Nielsen Company, 2016 The conclusions drawn from the Nielsen data are those of the researchers and do not reflect the views of Nielsen. Nielsen is not responsible for and was not involved in analyzing and preparing the results reported herein.

**Table 3:** MVD_(mean)_ and DASH_(mean)_ total score as predictors of first follow-up and rate of change in Allostatic Load (AL and components), overall and stratifying by sex, race and poverty status: multiple linear mixed-effects regression models, HANDLS 2004–2018 ^a,d^.

	Overall	Men	Women	Whites	African-Americans	Below Poverty	Above Poverty
***Allostatic Load* (*AL*)**	**(*n =* 1479; *k =* 1.8)**	**(*n =* 600; *k =* 1.8)**	**(*n =* 879; *k =* 1.8)**	**(*n =* 590; *k =* 1.8)**	**(*n =* 889; *k =* 1.8)**	**(*n =* 605; *k =* 1.8)**	**(*n =* 874; *k* = 1.8)**
Model 1:							
Intercept	+2.73 ± 0.27 ***^,b^	+3.36 ± 0.36 ***^,b^	+2.34 ± 0.34 ***^,b^	+2.38 ± 0.37 ***^,b^	+2.11 ± 0.75 *^,b^	+2.50 ± 0.36 ***^,b^	+2.00 ± 0.73 **^,b^
TIME	+0.00 ± 0.07	−0.19 ± 0.10 *^,b^	−0.01 ± 0.09	+0.06 ± 0.11	+0.16 ± 0.18	+0.09 ± 0.10	+0.16 ± 0.19
MVD_(mean)_	+0.03 ± 0.02 *^,b^	+0.01 ± 0.02 *^c^*	*+0.05 ± 0.02 ^c^*	+0.03 ± 0.02	+0.04 ± 0.02	+0.03 ± 0.03	+0.03 ± 0.02
MVD_(mean)_ × TIME	+0.002 ± 0.004	+0.005 ± 0.006	+0.001 ± 0.006	+0.004 ± 0.006	+0.002 ± 0.006	+0.01 ± 0.01	−0.002 ± 0.05
DASH_(mean)_	−0.083 ± 0.008 **^,b^	−0.090 ± 0.055	−0.069 ± 0.038	−0.095 ± 0.045	−0.070 ± 0.043	−0.08 ± 0.05	−0.074 ± 0.039
DASH_(mean)_ × TIME	+0.002 ± 0.004	+0.002 ± 0.015	−0.001 ± 0.009	−0.012 ± 0.012^c^	+0.010 ± 0.10^c^	−0.01 ± 0.01	+0.003 ± 0.010
Model 2:							
Intercept	+2.74 ± 0.27 ***^,b^	+3.38 ± 0.36 ***^,b^	+2.35 ± 0.34 ***^,b^	+2.40 ± 0.37 ***^,b^	+2.11 ± 0.75 **^,b^	+2.49 ± 0.36 ***^,b^	+2.02 ± 0.73 ***^,b^
TIME	−0.01 ± 0.07	−0.19 ± 0.10 *^,b^	−0.01 ± 0.09	+0.06 ± 0.11	+0.16 ± 0.18	+0.09 ± 0.10	+0.16 ± 0.19
MVD_(mean)_	+0.024 ± 0.017	+0.003 ± 0.024	+0.03 ± 0.02	+0.02 ± 0.02	+0.03 ± 0.02	+0.02 ± 0.03	+0.03 ± 0.02
MVD_(mean)_ × TIME	+0.002 ± 0.004	+0.005 ± 0.006	+0.001 ± 0.006	+0.002 ± 0.006	+0.003 ± 0.006	+0.007 ± 0.007	−0.001 ± 0.005
Model 3:							
Intercept	+2.74 ± 0.27 ***^,b^	+3.36 ± 0.36 **^,b^	+2.37 ± 0.34 ***^,b^	+2.41 ± 0.37 ***^,b^	+2.06 ± 0.75 **^,b^	+2.48 ± 0.36 ***^,b^	+2.03 ± 0.73 ***^,b^
TIME	+0.00 ± 0.07	−0.18 ± 0.10	−0.02 ± 0.09	+0.05 ± 0.11	+0.16 ± 0.18	+0.06 ± 0.10	+0.16 ± 0.19
DASH_(mean)_	−0.068 ± 0.031 *^,b^	−0.08 ± 0.05	−0.05 ± 0.04	−0.08 ± 0.04	−0.06 ± 0.04	−0.06 ± 0.05	−0.06 ± 0.04
DASH_(mean)_ × TIME	+0.001 ± 0.007	0.005 ± 0.014	−0.000 ± 0.009	−0.01 ± 0.01^c^	+0.01 ± 0.01^c^	−0.01 ± 0.01	+0.00 ± 0.01
*Serum Albumin* (*ALB)*	(*n* = 1471; *k* = 1.8)	(*n* = 597; *k* = 1.8)	(*n* = 874; *k* = 1.8)	(*n* = 588; *k* = 1.8)	(*n* = 883; *k* = 1.8)	(*n* = 601; *k* = 1.8)	(*n* = 870; *k* = 1.8)
Intercept	+4.32 ± 0.07 ***^,b^	+4.27 ± 0.10 ***^,b^	+4.26 ± 0.09 ***^,b^	+4.34 ± 0.10 ***^,b^	+4.11 ± 0.20 ***^,b^	+4.43 ± 0.11 ***^,b^	+4.15 ± 0.19 ***^,b^
TIME	−0.03 ± 0.02	−0.00 ± 0.03	−0.01 ± 0.03	−0.05 ± 0.03	−0.002 ± 0.05	−0.062 ± 0.028	−0.014 ± 0.05
MVD_(mean)_	+0.012 ± 0.005 *^,b^	+0.013 ± 0.007	+0.010 ± 0.006	+0.011 ± 0.007	+0.014 ± 0.007 *^,b^	+0.006 ± 0.009	+0.017 ± 0.006 **^,b^
MVD_(mean)_ × TIME	−0.001 ± 0.001	−0.001 ± 0.002	−0.000 ± 0.002	−0.001 ± 0.002	−0.000 ± 0.002	−0.001 ± 0.002	−0.00 ± 0.001
DASH_(mean)_	+0.018 ± 0.009 *^,b^	+0.011 ± 0.016	*+0.018 ± 0.010*	+0.020 ± 0.012	+0.019 ± 0.012	+0.010 ± 0.016	+0.021 ± 0.010 *^,b^
DASH_(mean)_ × TIME	−0.002 ± 0.002	+0.002 ± 0.004	−0.003 ± 0.002	−0.007 ± 0.003 *^,b^	−0.001 ± 0.003	−0.000 ± 0.004	−0.004 ± 0.002
*High-sensitivity C-reactive Protein* (*CRP)*	(*n* = 1465; *k* = 1.8)	(*n* = 595; *k* = 1.8)	(*n* = 870; *k* = 1.8)	(*n* = 586; *k* = 1.8)	(*n* = 879; *k* = 1.8)	(*n* = 600; *k* = 1.8)	(*n* = 865; *k* = 1.8)
Intercept	+12.1 ± 2.4 ***^,b^	+8.6 ± 2.9 ***^,b^	+6.86 ± 3.24 *^,b^	+19.6 ± 3.1 ***^,b^	+6.37 ± 6.81	+16.1 ± 3.7 ***^,b^	−1.27 ± 5.52
TIME	−0.76 ± 0.74	−1.48 ± 0.93	+0.83 ± 0.94	−3.14 ± 1.07 **^,b^	+2.02 ± 1.75	−1.86 ± 1.20	+1.71 ± 1.54
MVD_(mean)_	−0.009 ± 0.151	+0.13 ± 0.20	−0.15 ± 0.23	−0.05 ± 0.21	−0.08 ± 0.22	+0.37 ± 0.29	−0.13 ± 0.16
MVD_(mean)_ × TIME	−0.006 ± 0.040	−0.04 ± 0.06	+0.05 ± 0.06	+0.02 ± 0.06	−0.02 ± 0.06	−0.14 ± 0.08 ^c^	+0.05 ± 0.04 *^c^*
DASH_(mean)_	−0.033 ± 0.274	−0.11 ± 0.44	+0.14 ± 0.36	−0.38 ± 0.38	+0.43 ± 0.39	−0.50 ± 0.53	+0.18 ± 0.30
DASH_(mean)_ × TIME	−0.041 ± 0.073	−0.14 ± 0.14	−0.04 ± 0.09	−0.02 ± 0.11	−0.09 ± 0.10	+0.05 ± 0.14	−0.07 ± 0.08
*Total Serum Cholesterol* (*CHOL)*	(*n* = 1471; *k* = 1.8)	(*n* = 597; *k* = 1.8)	(*n* = 874; *k* = 1.8)	(*n* = 588; *k* = 1.8)	(*n* = 883; *k* = 1.8)	(*n* = 601; *k* = 1.8)	(*n* = 870; *k* = 1.8)
Intercept	+208.1 ± 9.6 ***^,b^	+188.1 ± 13.1 ***^,b^	+205.5 ± 12.1 ***^,b^	212.6 ± 13.6 ***^,b^	+157.2 ± 26.5 ***^,b^	+200.7 ± 12.9 ***^,b^	+216.6 ± 26.6 ***^,b^
TIME	+0.45 ± 2.30	+0.40 ± 2.77	+0.32 ± 3.02	+1.68 ± 3.61	+0.25 ± 5.7	+0.50 ± 3.08	+2.41 ± 5.83
MVD_(mean)_	−0.13 ± 0.62	+0.47 ± 0.90	−0.81 ± 0.87	−0.22 ± 0.92	−0.21 ± 0.86	−0.51 ± 1.04	−0.05 ± 0.79
MVD_(mean)_ × TIME	+0.08 ± 0.13	−0.07 ± 0.18	+0.25 ± 0.18	+0.20 ± 0.20	+0.09 ± 0.17	−0.15 ± 0.21	+0.23 ± 0.16
DASH_(mean)_	−2.05 ± 1.13	−2.98 ± 2.02	−1.90 ± 1.36	−3.58 ± 1.68	−0.83 ± 1.54	−2.94 ± 1.89	−1.24 ± 1.42
DASH_(mean)_ × TIME	+0.17 ± 0.23	+0.43 ± 0.43	+0.09 ± 0.28	−0.26 ± 0.38 ^c^	*+0.50 ± 0.30* ^c^	+0.22 ± 0.38	+0.14 ± 0.29
*High-Density Lipoprotein-Cholesterol* (*HDL-C)*	(*n* = 1471; *k* = 1.8)	(*n* = 597; *k* = 1.8)	(*n* = 874; *k* = 1.8)	(*n* = 588; *k* = 1.8)	(*n* = 883; *k* = 1.8)	(*n* = 601; *k* = 1.8)	(*n* = 870; *k* = 1.8)
Intercept	+57.7 ± 4.00 ***^,b^	+51.4 ± 5.0 ***^,b^	+60.3 ± 5.2 ***^,b^	+58.0 ± 4.7 ***^,b^	+38.4 ± 12.1 ***^,b^	+54.3 ± 5.6 ***^,b^	+46.1 ± 10.6 ***^,b^
TIME	−1.39 ± 0.86	−0.06 ± 1.10	−2.64 ± 1.10 **^,b^	−0.67 ± 1.10	−1.00 ± 2.18	*−1.91 ± 1.23*	+0.99 ± 2.08
MVD_(mean)_	+0.07 ± 0.26	−0.08 ± 0.34	+0.27 ± 0.37	−0.27 ± 0.31	+0.25 ± 0.39	+0.06 ± 0.45	−0.01 ± 0.31
MVD_(mean)_ × TIME	+0.06 ± 0.05	+0.06 ± 0.07	+0.06 ± 0.07	+0.12 ± 0.06	+0.03 ± 0.07	+0.05 ± 0.09	+0.06 ± 0.06
DASH_(mean)_	+0.09 ± 0.47	+0.96 ± 0.78	−0.69 ± 0.59	+0.38 ± 0.57	−0.29 ± 0.70	+0.73 ± 0.82	−0.18 ± 0.57
DASH_(mean)_ × TIME	−0.01 ± 0.09	+0.05 ± 0.17	−0.01 ± 0.11	+0.07 ± 0.12	−0.08 ± 0.12	−0.05 ± 0.15	+0.04 ± 0.10
*Glycated Hemoglobin* *(HBA1C)*	(*n* = 1470; *k* = 1.8)	(*n* = 598; *k* = 1.8)	(*n* = 872; *k* = 1.8)	(*n* = 588; *k* = 1.8)	(*n* = 884; *k* = 1.8)	(*n* = 601; *k* = 1.8)	(*n* = 870; *k* = 1.8)
Intercept	+6.60 ± 0.29 ***^,b^	+7.50 ± 0.44 ***^,b^	+6.42 ± 0.33 ***^,b^	+6.89 ± 0.39 ***^,b^	+5.78 ± 0.81 ***^,b^	+6.84 ± 0.40 ***^,b^	+5.17 ± 0.76***^,b^
TIME	+0.12 ± 0.06	−0.16 ± 0.09	+0.10 ± 0.07	+0.19 ± 0.09 *^,b^	+0.46 ± 0.16 ***^,b^	+0.21 ± 0.08 *^,b^	+0.21 ± 0.17
MVD_(mean)_	+0.05 ± 0.02 *^,b^	*+0.05 ± 0.03*	+0.03 ± 0.02^c^	+0.05 ± 0.03 ^c^	+0.002 ± 0.005	*+0.06 ± 0.03*	+0.05 ± 0.02 *^,b^
MVD_(mean)_ × TIME	−0.001 ± 0.004	−0.009 ± 0.006	+0.009 ± 0.004 *^,b^	−0.002 ± 0.005	−0.010 ± 0.05	+0.006 ± 0.006	−0.003 ± 0.005
DASH_(mean)_	−0.023 ± 0.034	−0.139 ± 0.068 *^,b,c^	+0.035 ± 0.037^c^	−0.025 ± 0.009	+0.010 ± 0.009	−0.056 ± 0.059	−0.009 ± 0.041
DASH_(mean)_ × TIME	+0.000 ± 0.007	+0.025 ± 0.014^c^	−0.014 ± 0.007 *^,b,c^	−0.006 ± 0.009 ^c^	+0.010 ± 0.009 ^c^	+0.003 ± 0.010	+0.000 ± 0.009
*Waist-to-Hip Ratio* (*WHR)*	(*n* = 1475; *k* = 1.8)	(*n* = 598; *k* = 1.8)	(*n* = 877; *k* = 1.8)	(*n* = 590; *k* = 1.8)	(*n* = 885; *k* = 1.8)	(*n* = 601; *k* = 1.8)	(*n* = 874; *k* = 1.8)
Intercept	+0.95 ± 0.02 ***^,b^	+1.04 ± 0.02 ***^,b^	+0.94 ± 0.02 ***^,b^	+0.88 ± 0.02 ***^,b^	+0.97 ± 0.08 ***^,b^	+0.94 ± 0.002 ***^,b^	+0.92 ± 0.08 ***^,b^
TIME	+0.002 ± 0.002	+0.003 ± 0.005	+0.003 ± 0.012	+0.014 ± 0.007*	+0.008 ± 0.023	+0.011 ± 0.007	+0.019 ± 0.025
MVD_(mean)_	+0.003 ± 0.002	+0.002 ± 0.001	+0.003 ± 0.003	+0.002 ± 0.002	+0.004 ± 0.003	+0.003 ± 0.002	+0.003 ± 0.002
MVD_(mean)_ × TIME	−0.000 ± 0.000	−0.000 ± 0.000	+0.000 ± 0.001	+0.001 ± 0.002	−0.001 ± 0.001	+0.000 ± 0.000	−0.000 ± 0.001
DASH_(mean)_	−0.003 ± 0.003	−0.006 ± 0.003 *^,b^	−0.001 ± 0.004	−0.004 ± 0.003	−0.002 ± 0.005	−0.005 ± 0.004	−0.002 ± 0.004
DASH_(mean)_ × TIME	−0.000 ± 0.001	+0.002 ± 0.001 *^,b^	−0.001 ± 0.001	−0.001 ± 0.001	+0.000 ± 0.001	−0.000 ± 0.001	−0.001 ± 0.001
*Systolic Blood Pressure* (*SBP)*	(*n* = 1478; *k* = 1.8)	(*n* = 599; *k* = 1.8)	(*n* = 879; *k* = 1.8)	(*n* = 590; *k* = 1.8)	(*n* = 888; *k* = 1.8)	(*n* = 604; *k* = 1.8)	(*n* = 874; *k* = 1.8)
Intercept	+124.2 ± 3.9 ***^,b^	+124.8 ± 4.9 ***^,b^	+122.5 ± 5.1 ***^,b^	+122.7 ± 5.0 ***^,b^	+113.3 ± 11.2 ***^,b^	+122.1 ± 5.5 ***^,b^	+121.9 ± 10.1 ***^,b^
TIME	−1.23 ± 1.39	−2.45 ± 1.60	−0.34 ± 1.93	−1.67 ± 1.81	+0.22 ± 3.78	−0.59 ± 1.89	−4.84 ± 3.74
MVD_(mean)_	+0.08 ± 0.25	+0.11 ± 0.34	+0.04 ± 0.37	+0.05 ± 0.34	−0.07 ± 0.37	−0.01 ± 0.45	+0.05 ± 0.30
MVD_(mean)_ × TIME	−0.03 ± 0.08	+0.11 ± 0.10	−0.14 ± 0.13	+0.10 ± 0.11	−0.14 ± 0.12	−0.06 ± 0.14	−0.01 ± 0.10
DASH_(mean)_	−0.696 ± 0.454	−0.40 ± 0.76	−0.88 ± 0.58	−0.83 ± 0.62	−0.55 ± 0.65	−0.42 ± 0.81	−0.89 ± 0.54
DASH_(mean)_ × TIME	−0.017 ± 0.153	−0.09 ± 0.25	+0.04 ± 0.20	−0.15 ± 0.21	+0.08 ± 0.21	−0.17 ± 0.25	+0.06 ± 0.19
*Diastolic Blood Pressure* (*DBP)*	(*n* = 1478; *k* = 1.8)	(*n* = 599; *k* = 1.8)	(*n* = 879; *k*= 1.8)	(*n* = 590; *k* = 1.8)	(*n* = 888; *k* = 1.8)	(*n* = 604; *k* = 1.8)	(*n* = 874; *k* = 1.8)
Intercept	+70.6 ± 2.2 ***^,b^	+69.6 ± 3.1 ***^,b^	+73.5 ± 2.8 ***^,b^	+68.3 ± 2.9 ***^,b^	+74.9 ± 6.6 ***^,b^	+69.4 ± 3.1 ***^,b^	+77.0 ± 6.1 ***^,b^
TIME	−1.57 ± 0.78 *^,b^	−0.98 ± 0.93	−1.33 ± 1.07	−2.36 ± 1.03 *^,b^	−0.98 ± 2.10	−1.18 ± 1.08	−4.30 ± 2.10 *^,b^
MVD_(mean)_	+0.06 ± 0.15	+0.07 ± 0.21	+0.12 ± 0.20	−0.10 ± 0.19	+0.19 ± 0.22	−0.07 ± 0.25	+0.07 ± 0.18
MVD_(mean)_ × TIME	−0.05 ± 0.05	−0.02 ± 0.06	−0.06 ± 0.07	+0.04 ± 0.06	*−0.13 ± 0.07*	−0.07 ± 0.08	−0.03 ± 0.06
DASH_(mean)_	−0.05 ± 0.27	−0.27 ± 0.48	−0.08 ± 0.32	+0.17 ± 0.35	−0.20 ± 0.39	−0.01 ± 0.46	−0.08 ± 0.32
DASH_(mean)_ × TIME	+0.05 ± 0.09	+0.004 ± 0.143	+0.09 ± 0.11	−0.13 ± 0.12	+0.18 ± 0.12	+0.08 ± 0.14	+0.05 ± 0.11
*Heart Rate* (*HR)*	(*n =* 1473; *k =* 1.8)	(*n =* 598; *k =* 1.8)	(*n =* 879; *k* = 1.8)	(*n =* 589; *k* = 1.8)	(*n =* 884; *k* = 1.8)	(*n* = 602; *k* = 1.8)	(*n =* 871; *k =* 1.8)
Intercept	+71.9 ± 2.7 ***^,b^	+65.7 ± 3.6 ***^,b^	+72.8 ± 3.4 ***^,b^	+71.0 ± 3.6 ***^,b^	+80.4 ± 7.5 ***^,b^	+72.8 ± 3.7 ***^,b^	+71.2 ± 7.1 ***^,b^
TIME	−0.07 ± 0.67	+0.11 ± 0.87	−0.02 ± 0.87	−0.59 ± 1.02	+1.74 ± 1.66	−0.80 ± 0.90	*+3.32 ± 1.82*
MVD_(mean)_	+0.24 ± 0.17	+0.23 ± 0.25	+0.27 ± 0.24	+0.28 ± 0.24	+0.30 ± 0.24	+0.15 ± 0.30	+0.35 ± 0.21
MVD_(mean)_ × TIME	+0.04 ± 0.04	−0.08 ± 0.06	+0.14 ± 0.06 *^,b^	+0.03 ± 0.06	+0.05 ± 0.05	+0.06 ± 0.07	+0.01 ± 0.05
DASH_(mean)_	−0.12 ± 0.31	−0.83 ± 0.56	+0.10 ± 0.38	+0.02 ± 0.44	−0.09 ± 0.44	+0.50 ± 0.54	−0.48 ± 0.38
DASH_(mean)_ × TIME	−0.06 ± 0.07	+0.29 ± 0.13 *^,b,c^	−0.20 ± 0.09 *^,b,c^	−0.08 ± 0.12	−0.05 ± 0.09	*−0.22 ± 0.12*	+0.05 ± 0.09

Abbreviations: AL = Allostatic load; DASH = Dietary Approaches to Stop Hypertension; HANDLS = Healthy Aging in Neighborhood of Diversity across the Lifespan; MVD = Monetary value of the diet, SE = Standard error. ^a^ Values are fixed effects regression coefficients from mixed-effects linear regression models (γ ± SE). Models were adjusted for baseline age (Agew1), age at first follow-up (Agew3), sex, race, poverty status, educational attainment, literacy, employment status, current smoking status, current drug use, body mass index, self-rated health, mean of total energy intake and in % energy from grocery stores. Agew1 was centered at 48; Agew3 was centered at 53; Body mass index BMI) at baseline was centered at 30; Energy intake was centered at 2030 kcal/day; Energy from stores centered at 1550 kcal/day; DASH_(mean)_ centered at 1.74; MVD_(mean)_ centered at 6.3. ^b^ *p <* 0.05 for null hypothesis that γ = 0. ^c^ *p <* 0.05 for null hypothesis of no difference by sex, race, or poverty status based on 2-way and 3-way interaction terms with MVD/DASH and TIME. ^d^ Researchers own analyses and calculations based in part on data reported by Nielsen through its Homescan Service for the food and beverage categories for the years 2004–2013, for the US market Nielsen data is licensed from The Nielsen Company, 2016 The conclusions drawn from the Nielsen data are those of the Researchers and do not reflect the views of Nielsen. Nielsen is not responsible for and was not involved in analyzing and preparing the results reported herein. * *p* < 0.05, ** *p* < 0.01, *** *p* < 0.001.

**Table 4:** zMVD_(mean)_, zDASH_(mean)_ total score and zAL_(v3)_ (and components) at second follow-up: mediation model relaxing the assumption of no interaction between exposure and mediator and stratifying by sex, race and poverty status, HANDLS 2004–2018 ^a^.

	Controlled Direct Effect			Natural Direct Effect			Natural Indirect Effect			Marginal Total Effect		
	*β*	(SEE)	*P_wald_*	*β*	(SEE)	*P_wald_*	*β*	(SEE)	*P_wald_*	*β*	(SEE)	*P_wald_*
Overall sample, *n* = 1224	+0.068	(0.041)	0.10	+0.068	(0.041)	0.10	−0.018	(0.013)	0.17	+0.050	(0.040)	0.21
Men, *n* = 479	+0.063	(0.058)	0.28	+0.067	(0.060)	0.26	−0.010	(0.013)	0.45	+0.057	(0.057)	0.32
Women, *n* = 745	+0.073	(0.060)	0.22	+0.075	(0.060)	0.21	−0.028	(0.025)	0.26	+0.047	(0.061)	0.44
Whites, *n* = 500	+0.077	(0.06)	0.23	+0.076	(0.063)	0.23	−0.040	(0.019)	0.037	+0.035	(0.061)	0.55
African-Americans, *n* = 724	+0.083	(0.058)	0.15	+0.076	(0.057)	0.19	+0.020	(0.023)	0.39	+0.095	(0.059)	0.11
Below poverty, *n* = 493	+0.081	(0.069)	0.24	+0.084	(0.070)	0.23	−0.043	(0.029)	0.13	+0.042	(0.067)	0.53
Above poverty, *n* = 731	+0.059	(0.053)	0.27	+0.061	(0.053)	0.25	−0.010	(0.013)	0.45	+0.050	(0.052)	0.33

Abbreviations: DASH = Dietary Approaches to Stop Hypertension; HANDLS = Healthy Aging in Neighborhood of Diversity across the Lifespan; MVD = Monetary value of the diet, SEE = Standard error of the estimate. ^a^ Multivariate ordinary least square (OLS) models adjusted for baseline age, sex, race, poverty status, educational attainment, literacy, employment status, current smoking status, current drug use, body mass index, self-rated health, mean of total energy intake and in % energy from grocery stores. Four parameters were estimated with SEE and *p*-values: CDE, NDE NIE, MTE. Those are described in more detail in statistical analysis section. For CDE, zDASH_(mean)_ was set at a value of zero. Only results with significant total effects at type I error of 0.05 were presented.

**Table 5:** Total, direct and indirect effects of zMVD(mean) on zDASH(mean) and zAL_(v3)_ (and components), overall and stratified by sex, race and poverty status based on structural equations modeling (N = 1224): HANDLS 2004–2018.

	zMVD→zAL: Direct Effect			zMVD→zDASH			zDASH→zAL			zMVD→zDASH→zAL: Indirect Effect			zMVD→zAL Total Effect: Direct + Indirect		
	β	(SEE)	P_wald_	β	(SEE)	P_wald_	β	(SEE)	P_wald_	β	(SEE)	P_wald_	β	(SEE)	P_wald_
AL															
Overall sample, *n* = 1224, CD = 0.36	+0.07	(0.04)	0.088	+0.32	(0.04)	<0.001	−0.07	(0.03)	0.028	−0.02	(0.01)	0.033	+0.05	(0.04)	0.22
Men, *n* = 479, CD = 0.40	−0.04	(0.05)	0.50	+0.22	(0.05) ^a^	<0.001	+0.06	(0.06)	0.28	−0.08	(0.01)	0.51	+0.05	(0.06)	0.33
Women, *n* = 745, CD = 0.36	+0.07	(0.06)	0.20	+0.45	(0.06) ^a^	<0.001	−0.07	(0.04)	0.052	−0.03	(0.02)	0.059	+0.04	(0.06)	0.45
Whites, *n* = 500, CD = 0.38	+0.07	(0.06)	0.22	+0.34	(0.06)	<0.001	−0.11	(0.05)	0.013	−0.04	(0.02)	0.022	+0.04	(0.06)	0.55
African-Americans, *n* = 724, CD = 0.34	−0.01	(0.04)	0.74	+0.32	(0.05)	<0.001	+0.07	(0.06)	0.19	−0.00	(0.01)	0.74	−0.01	(0.04)	0.74
Below poverty, *n* = 493, CD = 0.34	−0.08	(0.05)	0.12	+0.39	(0.06)	<0.001	−0.08	(0.05)	0.12	−0.03	(0.02)	0.13	−0.08	(0.05)	0.12
Above poverty, *n* = 731, CD = 0.38	−0.06	(0.04)	0.14	+0.28	(0.05)	<0.001	+0.06	(0.05)	0.23	−0.02	(0.01)	0.15	+0.05	(0.05)	0.35
ALB															
Men, *n* = 466	+0.08	(0.06)	0.20	+0.22	(0.05) ^a^	<0.001	+0.04	(0.06)	0.51	+0.01	(0.01)	0.52	+0.09	(0.06)	0.14
Women, *n* = 718	+0.07	(0.06)	0.28	+0.46	(0.06) ^a^	<0.001	−0.01	(0.04)	0.88	−0.00	(0.02)	0.89	+0.06	(0.06)	0.27
Whites, *n* = 1,184	+0.06	(0.06)	0.36	+0.36	(0.06)	<0.001	−0.04	(0.04)	0.39	−0.01	(0.02)	0.40	+0.04	(0.06)	0.48
African-Americans, *n* = 707	+0.10	(0.06)	0.094	+0.31	(0.05)	<0.001	+0.04	(0.04)	0.32	+0.01	(0.01)	0.32	+0.12	(0.06)	0.051
Below poverty, *n* = 481	+0.03	(0.08)	0.74	+0.40	(0.06)	<0.001	+0.03	(0.06)	0.58	+0.01	(0.02)	0.58	+0.03	(0.06)	0.58
Above poverty, *n* = 703	+0.11	(0.05)	0.034	+0.28	(0.05)	<0.001	−0.01	(0.04)	0.82	−0.00	(0.01)	0.82	+0.11	(0.05)	0.035
CRP															
Men, *n* = 465	−0.01	(0.06)	0.92	+0.22	(0.05) ^a^	<0.001	−0.05	(0.05)	0.32	−0.01	(0.00)	0.33	−0.02	(0.06)	0.75
Women, *n* = 717	+0.03	(0.07)	0.61	+0.46	(0.06) ^a^	<0.001	−0.02	(0.04)	0.57	−0.01	(0.02)	0.61	+0.02	(0.06)	0.72
Whites, *n* = 476	+0.02	(0.05)	0.65	+0.36	(0.06)	<0.001	−0.04	(0.04)	0.28	+0.00	(0.00)	0.38	+0.01	(0.05)	0.87
African-Americans, *n* = 706	−0.03	(0.07)	0.59	+0.31	(0.05)	<0.001	−0.01	(0.05)	0.87	−0.00	(0.02)	0.87	−0.04	(0.07)	0.56
Below poverty, *n* = 480	−0.07	(0.06)	0.45	+0.40	(0.06)	<0.001	−0.04	(0.06)	0.45	−0.02	(0.02)	0.49	−0.08	(0.08)	0.27
Above poverty, *n* = 702	−0.03	(0.05)	0.55	+0.28	(0.05)	<0.001	−0.01	(0.03)	0.72	−0.00	(0.01)	0.72	+0.03	(0.05)	0.59
CHOL															
Men, *n* = 466	−0.07	(0.06)	0.25	+0.22	(0.05) ^a^	<0.001	−0.07	(0.06)	0.25	−0.01	(0.01)	0.26	+0.00	(0.06)	0.97
Whites, *n* = 477	+0.06	(0.06)	0.37	+0.36	(0.06)	<0.001	−0.12	(0.05) ^a^	0.012	−0.04	(0.02)	0.021	+0.02	(0.06)	0.81
African-Americans, *n* = 708	−0.02	(0.06)	0.68	+0.31	(0.05)	<0.001	+0.03	(0.04) ^a^	0.45	+0.01	(0.01)	0.45	−0.01	(0.06)	0.81
Below poverty, *n* = 481	−0.11	(0.07)	0.14	+0.40	(0.06)	<0.001	−0.03	(0.05)	0.58	−0.01	(0.02)	0.58	−0.12	(0.07)	0.088
Above poverty, *n* = 704	+0.06	(0.06)	0.29	+0.28	(0.05)	<0.001	−0.04	(0.04)	0.29	−0.01	(0.01)	0.30	+0.05	(0.05)	0.38
HDL-C															
Men, *n* = 465	+0.03	(0.05)	0.55	+0.22	(0.05) ^a^	<0.001	+0.07	(0.05)	0.18	+0.02	(0.01)	0.20	+0.05	(0.05)	0.37
Women, *n* = 718	+0.04	(0.06)	0.50	+0.46	(0.06) ^a^	<0.001	−0.03	(0.04)	0.48	−0.01	(0.02)	0.48	+0.03	(0.06)	0.62
Whites, *n* = 476	+0.03	(0.05)	0.52	+0.35	(0.06)	<0.001	+0.05	(0.04)	0.20	+0.02	(0.01)	0.21	+0.05	(0.04)	0.20
African-Americans, *n* = 707	+0.03	(0.06)	0.67	+0.31	(0.05)	<0.001	−0.03	(0.04)	0.56	−0.01	(0.01)	0.56	+0.02	(0.06)	0.76
Below poverty, *n* = 480	+0.04	(0.07)	0.62	+0.40	(0.06)	<0.001	+0.04	(0.05)	0.46	+0.02	(0.02)	0.46	+0.05	(0.07)	0.45
Above poverty, *n* = 703	+0.02	(0.05)	0.71	+0.28	(0.05)	<0.001	+0.01	(0.04)	0.81	+0.00	(0.02)	0.81	+0.02	(0.05)	0.67
HBA1C															
Men, *n* = 465	+0.02	(0.07)	0.75	+0.22	(0.05) ^a^	<0.001	−0.00	(0.06)	0.95	−0.00	(0.01)	0.95	+0.02	(0.07)	0.76
Women, *n* = 713	+0.17	(0.05)	0.002	+0.46	(0.06) ^a^	<0.001	−0.01	(0.03)	0.76	−0.00	(0.02)	0.76	+0.16	(0.05)	0.002
Whites, *n* = 479	+0.10	(0.06)	0.080	+0.36	(0.06)	<0.001	−0.04	(0.04)	0.34	−0.01	(0.02)	0.44	+0.09	(0.06)	0.12
African-Americans, *n* = 705	+0.12	(0.06)	0.047	+0.31	(0.05)	<0.001	+0.04	(0.05)	0.31	+0.01	(0.01)	0.32	+0.14	(0.06)	0.023
Below poverty, *n* = 479	+0.17	(0.06)	0.010	+0.40	(0.06)	<0.001	−0.01	(0.05)	0.88	−0.00	(0.02)	0.89	+0.16	(0.06)	0.008
Above poverty, *n* = 699	+0.09	(0.06)	0.12	+0.28	(0.05)	<0.001	+0.00	(0.04)	0.94	+0.00	(0.01)	0.94	+0.09	(0.06)	0.11
WHR															
Men, *n* = 475	+0.02	(0.05)	0.63	+0.22	(0.05)	<0.001	+0.02	(0.05)	0.67	+0.00	(0.01)	0.67	+0.03	(0.05)	0.56
Women, *n* = 734	+0.12	(0.06)	0.051	+0.44	(0.06)	<0.001	−0.08	(0.04)	0.044	−0.03	(0.02)	0.051	+0.08	(0.06)	0.15
Whites, *n* = 495	+0.17	(0.06)	0.004	+0.34	(0.06)	<0.001	−0.10	(0.04)	0.020	−0.04	(0.02)	0.031	+0.13	(0.06)	0.019
African-Americans, *n* = 714	+0.02	(0.06)	0.76	+0.30	(0.05)	<0.001	−0.00	(0.04)	0.95	−0.00	(0.01)	0.95	+0.02	(0.05)	0.76
Below poverty, *n* = 485	+0.10	(0.07)	0.16	+0.37	(0.06)	<0.001	−0.05	(0.05)	0.39	−0.02	(0.02)	0.39	+0.08	(0.07)	0.23
Above poverty, *n* = 724	+0.06	(0.05)	0.22	+0.28	(0.05)	<0.001	−0.06	(0.04)	0.10	−0.02	(0.01)	0.12	+0.04	(0.05)	0.36
SBP															
Men, *n* = 477	+0.08	(0.05) ^a^	0.12	+0.22	(0.05) ^a^	<0.001	−0.05	(0.05)	0.33	−0.01	(0.01)	0.34	+0.07	(0.05)	0.17
Women, *n* = 738	−0.11	(0.07) ^a^	0.11	+0.44	(0.06) ^a^	<0.001	−0.05	(0.04)	0.28	−0.02	(0.02)	0.39	−0.13	(0.06)	0.050
Whites, *n* = 497	+0.03	(0.06)	0.55	+0.34	(0.06)	<0.001	−0.07	(0.04)	0.091	−0.02	(0.02)	0.22	+0.01	(0.05)	0.87
African-Americans, *n* = 718	−0.08	(0.06)	0.21	+0.30	(0.05)	<0.001	−0.01	(0.05)	0.79	−0.00	(0.01)	0.79	−0.09	(0.06)	0.17
Below poverty, *n* = 489	−0.05	(0.07)	0.54	+0.37	(0.06)	<0.001	−0.05	(0.06)	0.33	−0.02	(0.02)	0.34	−0.06	(0.07)	0.37
Above poverty, *n* = 726	−0.00	(0.05)	0.94	+0.28	(0.05)	<0.001	−0.04	(0.04)	0.34	−0.01	(0.01)	0.35	−0.01	(0.05)	0.78
DBP															
Men, *n* = 477	+0.00	(0.06)	0.94	+0.21	(0.05)	<0.001	−0.05	(0.05)	0.36	−0.01	(0.01)	0.37	−0.02	(0.06)	0.78
Women, *n* = 738	−0.07	(0.07)	0.31	+0.44	(0.06)	<0.001	+0.02	(0.04)	0.61	+0.01	(0.02)	0.61	−0.06	(0.06)	0.36
Whites, *n* = 497	−0.00	(0.06)	0.97	+0.34	(0.06)	<0.001	−0.03	(0.04)	0.44	−0.01	(0.01)	0.44	−0.01	(0.05)	0.80
African-Americans, *n* = 718	−0.09	(0.07)	0.17	+0.30	(0.05)	<0.001	+0.05	(0.05)	0.28	+0.02	(0.01)	0.29	−0.08	(0.06)	0.24
Below poverty, *n* = 489	−0.11	(0.08)	0.15	+0.37	(0.06)	<0.001	+0.04	(0.06)	0.44	+0.02	(0.02)	0.44	−0.09	(0.07)	0.20
Above poverty, *n* = 726	−0.02	(0.05)	0.72	+0.28	(0.05)	<0.001	+0.00	(0.04)	0.90	+0.00	(0.01)	0.90	−0.02	(0.05)	0.73
HR															
Men, *n* = 475	+0.01	(0.06) ^a^	0.81	+0.22	(0.05) ^a^	<0.001	+0.06	(0.06)	0.31	+0.01	(0.01)	0.32	+0.03	(0.06)	0.65
Women, *n* = 738	+0.21	(0.06) ^a^	0.001	+0.45	(0.06) ^a^	<0.001	−0.06	(0.04)	0.12	−0.03	(0.02)	0.12	+0.18	(0.06)	0.002
Whites, *n* = 495	+0.11	(0.07)	0.097	+0.34	(0.06)	<0.001	−0.03	(0.05)	0.59	−0.01	(0.02)	0.60	+0.10	(0.06)	0.11
African-Americans, *n* = 718	+0.11	(0.06)	0.057	+0.32	(0.05)	<0.001	+0.01	(0.04)	0.85	+0.00	(0.01)	0.85	+0.11	(0.06)	0.046
Below poverty, *n* = 490	+0.07	(0.07)	0.34	+0.39	(0.06)	<0.001	−0.01	(0.05)	0.80	−0.01	(0.02)	0.80	+0.06	(0.07)	0.36
Above poverty, *n* = 723	+0.10	(0.05)	0.053	+0.27	(0.05)	<0.001	−0.01	(0.04)	0.84	−0.00	(0.01)	0.84	+0.10	(0.05)	0.053

Abbreviation: AL = Allostatic load; CD = Coefficient of determination; DASH = Dietary Approaches to Stop Hypertension; HANDLS = Healthy Aging in Neighborhood of Diversity across the Lifespan; MVD = Monetary value of the diet, SEE = Standard error of the estimate. SEMs were adjusted for baseline age, sex, race, poverty status, educational attainment, literacy, employment status, current smoking status, current drug use, body mass index, self-rated health, mean of total energy intake and in % energy from grocery stores. ^a^ *p* < 0.05 for null hypothesis of model invariance across groups: sex, race and poverty status.

## Supplementary Materials

The following are available online at https://www.mdpi.com/2072-6643/11/10/2360/s1, Figure S1: Participant Flowchart; Method S1: Allostatic load; Method S2: HomeScan data description; Method S3: Food group description; Method S4: Description of mixed-effects regression models. Method S5: Stata syntax of the main analysis.

## Abbreviations

AL	Allostatic Load
DASH	Dietary Approach to Stop Hypertension
DQ	Diet Quality
HEI-2010	Healthy Eating Index, 2010 version
FNDDS	Food and Nutrient Database for Dietary Studies
HANDLS	Healthy Aging in Neighborhood of Diversity across the Life Span
HS	High School
MAR	Mean Adequacy Ratio
MVD	Monetary value of the diet
NAR	Nutrient Adequacy Ratio
NHANES	National Health and Nutrition Examination Surveys
PIR	Poverty Income Ratio
RDA	Recommended Dietary Allowance
SE	Standard Error
USDA	US Department of Agriculture
WRAT-3	Wide Range Achievement Test, 3rd revision
