# Supplementary material for: Longitudinal Associations between Monetary Value of the Diet, DASH Diet Score and the Allostatic Load among Middle-Aged Urban Adults
Source: Nutrients. 2019 Oct 3;11(10):2360. doi: 10.3390/nu11102360 (PMC6835231; doi:10.3390/nu11102360)
Supplement: Supplementary file 1 [file nutrients-11-02360-s001.pdf]

SUPPLEMENTAL FIGURE LEGEND AND OTHER SUPPLEMENTAL MATERIALS

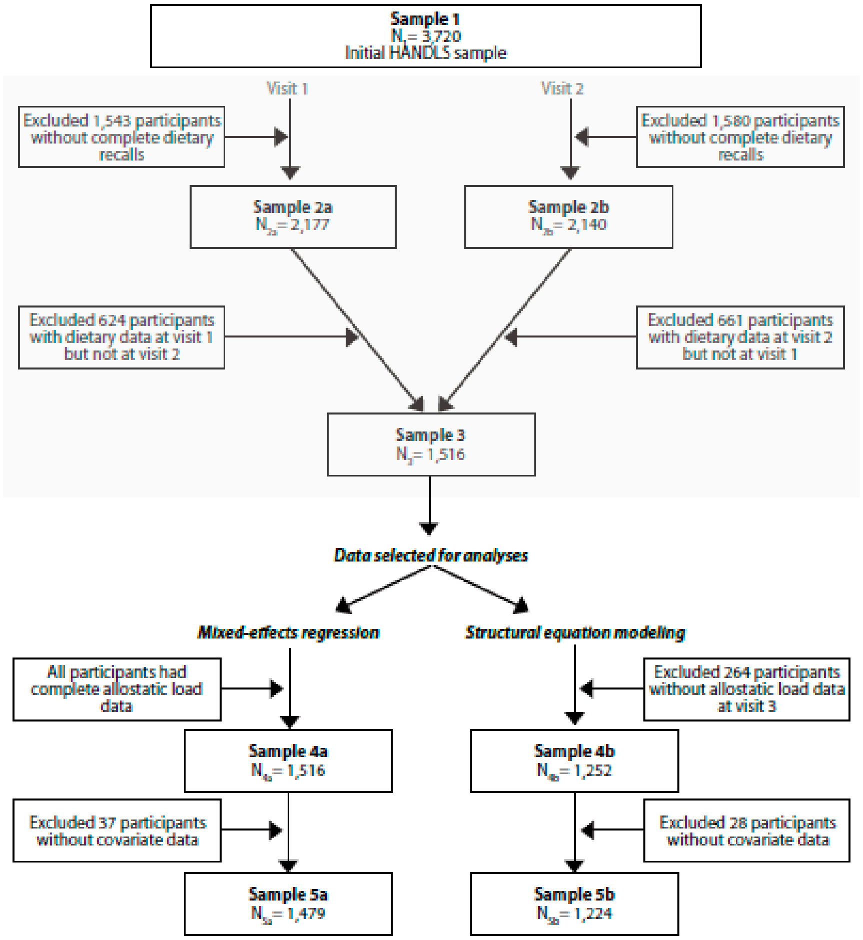

Figure S1. Participant Flowchart[a].

[a] Researchers own analyses and calculations based in part on data reported by Nielsen through its Homescan Service for the food and beverage categories for the years 2004–2013, for the US market Nielsen data is licensed from The Nielsen Company, 2016 The conclusions drawn from the Nielsen data are those of the Researchers and do not reflect the views of Nielsen. Nielsen is not responsible for and was not involved in analyzing and preparing the results reported herein.

Supplemental method S1: Allostatic load

Supplemental method S2: HomeScan data description

Supplemental method S3: Food group description

Supplemental method S4: Description of mixed-effects regression models

Supplemental method S5: Stata do file for main analysis

Supplemental methods S1: Allostatic load

A total AL score was computed using a method described in a previous study.[62] AL total score sums up cardiovascular (systolic and diastolic blood pressure, pulse rate), metabolic (total cholesterol, HDLcholesterol, glycosylated Hb, sex-specific waist-to-hip ratio) and inflammatory (albumin and C-reactive protein (CRP)) risk indicators. Clinical criteria summarized in Table 1 were used to obtain risk indicators which were summed with equal weighting to compute total AL score (range: 0-9).

Total cholesterol (mg/dL), HDL-cholesterol (mg/dL), CRP (mg/dL), albumin (g/dL) and glycosylated hemoglobin (%) were measured by contract laboratories using reference analytical methods (See Laboratory Procedures for NHANES III).[63] Using standard protocols, waist-to-hip ratio, radial pulse (beats/min), and systolic and diastolic blood pressure (mmHg) were measured by trained examiners. Specifically, blood pressure was measured using a mercury sphygmomanometer [63] The arithmetic mean of three systolic and diastolic pressures was used in analysis.

Table 1. Allostatic load indicator criteria[62].

	**High-risk clinical**
Albumin (g/dL)	< 3.8 [121]
C-reactive protein (mg/dL)	≥ 0.3 [122]
Waist:Hip	>0.9 for men; > 0.85 for women [123]
Total cholesterol (mg/dL)	≥240[124]
HDL (mg/dL)	<40[124]
Glycated hemoglobin (%)	≥6.4[125,126]
Resting heart rate (beat/min)	≥90[127]
Systolic BP	≥140[128]
Diastolic BP	≥90[128]

Supplemental Method S2: HomeScan data description

The Homescan panel is a nationwide sample of US households that record all packaged foods and beverages purchased from grocery stores, supermarkets, and other retail food stores continuously throughout the year. Households are followed prospectively and must report purchases for at least 10 months per year. The sample includes approximately 40,000-60,000 US households each year from 76 geographic markets, and Nielsen provides projection factor weights to generate nationally representative estimates.[129] Household members scan the Universal Product Code barcode on each purchased item after each shopping trip using a handheld scanner and report the quantity purchased. Methods for reporting price paid depend on the store where the purchase takes place. For most products, Nielsen imputes the price paid from store-level point-of-sales data ("ScanTrack") as the average price paid for the product from that store for the given week and market.[130] However, for items purchased from stores not covered by ScanTrack, households must manually record the price paid; if the reported price is outside of the typical range, Nielsen replaces the reported value with the median regional price.[130]

Supplemental methods S3: Food group description

**FG UNC**	**UNC Description of FG**	**HANDLS code for closest FG**
1	cheese	24,25,26
2	yogurt	
3	dairy products, other	
4	dairy-based desserts	30,31

5	meat	32,36,38,39
6	meat, breaded	33,35,37
7	processed meat	40
8	Eggs	44
9	Legumes	45
10	Nuts and nut products	57
11	Bread and bread products	1,2
12	TORTILLAS, TACO SHELLS, AND WRAPS	
13	QUICK BREADS	
14	GRAIN-BASED DESSERTS	
15	Grain-based bars	
16	Pasta and rice	4,5,6
17	Cereal	7
18	Fruit	13,14,15,16
19	Fruit dish	17
20	Vegetables	18,19,21
21	Starchy vegetables	20
22	Fried potatoes	
23	Fats and oils	54,55,56
24	Sweeteners	52
25	CANDY AND SWEET SNACKS	51,53
26	Baking products	11
27	Salt and seasoning	60
28	Soups and stews	14,41
29	Salty snacks	8,9
30	SAUCES, DIPS, AND CONDIMENTS	
31	Baby food	12
32	MIXED DISH, REFRIGERATED	
33	MIXED DISH, FROZEN	46
34	MIXED DISH, CANNED/SHELF-STABLE	
35	MIXED DISH, INSTANT/MIX	
36	Water	61 or closest neighbor
37	Coffee and tea	47
38	SSB	48,49
39	FRUIT AND VEGETABLE JUICE	
40	Milk	22,23
41	COCOA AND SWEETENED MILK BEVERAGES	
42	MILK SUBSTITUTES AND MILK BEVERAGES	58
	Use closest neighbor imputation instead if imputed	
	Use HANDLS code if imputed; UNC code otherwise	

HANDLS 61 food groups.

GRAINS

1. (1) Refined breads, (3) Multigrain breads, (5) Low sodium breads, (6) Refined Quick breads

2. (2) 100% whole wheat breads, (4) High fiber, reduced calorie breads, (7) Whole wheat quick breads, (10) Whole wheat pasta without added fat, (11) Whole wheat pasta with added fat, (12) Brown rice without fat added, (13) Brown rice with fat added
3. (18) Whole grain cooked cereals no fat added, (19) Whole grain cooked cereals with fat added, (25) Whole wheat crackers, (21) High fiber cereals
4. (8) Pastas without added fat, (9) Pastas with added fat, (154) Pasta with meat, (155) Pasta without meat
5. (14) Cereals and white rice without added fat, (15) Cereals and white rice with added fat, (156) Rice dishes with meat, (157) Rice dishes without meat
6. (16) Cooked cereals with no fat, (17) Cooked cereals with fat added
7. (20) Ready to eat cereals, (37) Breakfast bars
8. (22) Regular crackers, (26) Salty snacks (chips, pretzels, popcorn, chips)
9 (23) Reduced fat crackers, (27) Low sodium snacks (pretzels, crackers, chips, popcorn), (28) Reduced fat potato chips
10. (24) Sweet crackers (graham, animal), (29) Cakes, (31) Doughnuts, (32) Cookies, (34) Pies (excludes fruit pies), (36) Pastries
11. (30) Diet cakes and pastries, (33) Diet cookies
12. (39) Baby foods, (129) Infant formulas, (230) Baby foods

FRUITS

13. (50) Raw, canned, frozen fruit without added sugar, (62) Unsweetened fruit juices,
(51) Canned sweetened fruit, (63) Sweetened fruit juices,
(52) Citrus fruits without added sugar, (53) Citrus fruits with added sugar, (60) Unsweetened citrus fruit juices, (61) Sweetened citrus fruit juices,
14. (54) Berries without added sugar, (55) Berries with added sugar
15. (56) Dried fruit, dried fruit cooked without added sugar, (57) Dried fruit cooked with added sugar
16. (58) Fruit desserts, (59) Fruit with added fat, (35) Fruit pies

VEGETABLES

17. (200) Raw and cooked without fat dark green vegetables, (204) Low sodium canned dark green vegetables, (202) Canned dark green vegetables with or without added fat, (201) Cooked with added fat dark green vegetables
18. (205) Raw and cooked without fat orange vegetables, (207) Canned orange vegetables with or without added fat, (209) Low sodium canned orange vegetables, (210) Cooked orange vegetables with sugar added, (206) Cooked with added fat orange vegetables, carrot juice
19. (211) Raw and cooked without fat starchy vegetables, (213) Canned starchy vegetables without added fat, (215) Low sodium canned starchy vegetables, (224) Canned vegetable combinations cooked with or without fat, (212) Cooked with added fat starchy vegetables, (214) Canned starchy vegetables with added fat, (216) French fried potatoes
20. (217) Raw and cooked without fat other vegetables, (219) Canned other vegetables without added fat, (221) Low sodium canned other vegetables, (222) Vegetable combinations cooked without fat, (218) Cooked with added fat other vegetables, (220) Canned other vegetables with added fat, (223) Vegetable combinations cooked with fat, (229) Vegetable salads with added fat, (228) Pickled vegetables, (226) Vegetable juices, (227) Low sodium vegetable juices, (232) Low sodium veg combinations

MILK and MILK PRODUCTS

21. (100) Regular milk
22. (101) Reduced fat milk, (102) Fat free milk
23. (112) Natural, regular cheese, (116) Low sodium cheese
24. (113) Natural, reduced fat and fat free cheese
25. (114) Processed, regular cheese
26. (115) Processed, reduced fat and fat free cheese
27. Regular dairy products:

(120) Regular cheese products (cottage, cream), (106) Regular cream, (108) Regular milk based beverages, (117) Regular cheese sauce, (122) Cheese based soups, (110) Regular yogurt (includes frozen)

28. Low fat dairy products:
(121) Lowfat or fat free cheese products (cottage, cream), (107) Reduced fat cream, (109) Reduced fat milk based beverages, (118) Low fat cheese sauce, (111) Lowfat yogurt (includes frozen)
29. Dairy desserts- regular: (123) Regular ice cream, (126) Regular pudding, (103) Condensed milk
30. Dairy desserts – low or ff desserts: (124) Light ice cream, (125) Fat free ice cream, (127) Reduced fat pudding, (162) nondairy frozen desserts

MEATS

31. (300) Lean red meats no added fat, (309) Veal, (311) Game
32. (301) Red meats with fat, (308) Lamb
33. (302) Chicken/poultry no added fat
34. (303) Chicken/poultry with added fat
35. (304) Fin fish no fat
36. (305) Fin fish with added fat
37. (306) Shellfish
38. Sandwich: (119) Cheese sandwich, (320) Beef/pork sandwiches, (321) Poultry sandwiches, (322) Fish sandwiches, (323) Bacon/sausage hot dog sandwiches, (324) Submarine sandwiches and luncheon meat sandwiches, (38) Sandwiches (croissant, turnover)
39. (307) Sausage/bacon/luncheon meats, (310) Organ meats
40. (312) Meat dishes, (325) Frozen meat meals, (329) Frozen veal meals,
(313) Chicken dishes, (327) Frozen chicken meals,
(314) Seafood dishes, (330) Frozen fish meals, (150) Hispanic dishes with meat, (160) Dumplings and egg rolls
41. Soups: (315) Mixed meats (stews, gumbo), (316) Beef/pork soups, (317) Poultry soups, (318) Seafood soups, (161) Grain-based soups with meat,(231)Vegetable soups
42. Diet Frozen meals: (326) meat, (328) chicken, (331) fish

EGGS

43. Egg dishes: (140) Egg dishes without fat, (141) Egg dishes with added fat, (142) Egg substitutes, (143) Egg sandwiches, (144) Frozen egg meals

LEGUMES

44. (250) Legumes prepared with fat, (251) Legumes prepared without fat, (252) Canned legumes prepared with fat, (253) Canned legumes prepared without fat, (254) Low sodium canned legumes, (255) Legume prepared dishes with meat, (256) Legume prepared dishes without meat, (257) Legume based soups, (258) Low sodium legume based soups, (104) Soy milk, (151) Hispanic dishes without meat

MIXED DISHES

45. Pizza: (152) Pizza with meat, (153) Pizza without meat

BEVERAGES

46. Coffee/ Tea: (80) Coffee, (83) Coffee substitutes, (84) Tea
47. Sweetened drinks: (89) Regular soft drinks, (81) Presweetened coffee, (85) Presweetened tea, (87) Fruit drinks
48. Diet drinks: (90) Diet soft drinks, (82) Coffee with low calorie sweeteners, (86) Tea with low calorie sweetener, (88) Low calories fruit drinks
49. (91) Alcoholic beverages

SUGARS

50. Sugar: (400) Added sugars, (402) Regular gelatin dessert
51. Sugar substitute: (401) Sugar substitutes, (403) Sugar free gelatin desserts, (405) Dietetic free/low calorie candy
52. (404) Candy

FATS

53. (420) Animal fats and salad dressings, (319) Meat gravy
54. (421) Vegetable fats and salad dressings
55. (422) Reduced calorie spreads and salad dressings

NUTS

56. (450) Nuts and nut butters, (451) Low sodium nuts and nut butters, (452) Peanut butter sandwiches

OTHERS

57. (470) Protein powders and meal replacements, (128) Milk based powders, Milk substitutes, nutritional beverage.
58. (480) Yeast
59. (490) Condiments
60. Water

Harmonized food group among **non-imputed** food codes, visit 1, day 1

Food_group_ final	Freq.	Percent	Cum.
**1**	**796**	**5.98**	**5.98**
2	74	0.56	6.53
3	540	4.06	10.59
4	215	1.61	12.20
5	288	2.16	14.37
6	155	1.16	15.53
**7**	**1,168**	**8.77**	**24.30**
8	479	3.60	27.90
9	45	0.34	28.24
10	190	1.43	29.66
**11**	**1,029**	**7.73**	**37.39**
12	22	0.17	37.56
13	217	1.63	39.19
14	587	4.41	43.59
15	46	0.35	43.94
16	71	0.53	44.47
17	380	2.85	47.33
18	248	1.86	49.19
19	2	0.02	49.20
**20**	**1,036**	**7.78**	**56.98**
21	33	0.25	57.23
22	118	0.89	58.12
23	300	2.25	60.37
24	301	2.26	62.63
25	104	0.78	63.41
26	4	0.03	63.44
27	1	0.01	63.45
28	113	0.85	64.30
29	383	2.88	67.17
30	654	4.91	72.09

32	321	2.41	74.50
33	532	4.00	78.49
34	147	1.10	79.60
35	84	0.63	80.23
**37**	**1,249**	**9.38**	**89.61**
38	325	2.44	92.05
39	281	2.11	94.16
**40**	**706**	**5.30**	**99.46**
41	41	0.31	99.77
42	31	0.23	100.00
Total	13,316		100.00

Harmonized food group among **imputed** food codes, visit 1, day 1

Food_group_ final	Freq.	Percent	Cum.
1	8	0.06	0.06
3	5	0.04	0.09
4	90	0.64	0.73
5	559	3.95	4.68
**6**	**804**	**5.68**	**10.36**
7	70	0.49	10.86
8	68	0.48	11.34
9	61	0.43	11.77
10	32	0.23	12.00
11	622	4.40	16.39
12	4	0.03	16.42
13	26	0.18	16.60
14	337	2.38	18.99
16	510	3.61	22.59
17	124	0.88	23.47
18	517	3.65	27.12
19	19	0.13	27.26
**20**	**1,243**	**8.79**	**36.04**
21	338	2.39	38.43
22	406	2.87	41.30
23	256	1.81	43.11
24	107	0.76	43.87
**25**	**1,192**	**8.43**	**52.29**
26	5	0.04	52.33
27	237	1.68	54.00
28	266	1.88	55.88
29	547	3.87	59.75
30	158	1.12	60.87
31	2	0.01	60.88
32	143	1.01	61.89
33	157	1.11	63.00
34	79	0.56	63.56
35	76	0.54	64.10
**37**	**1,970**	**13.93**	**78.02**
38	2,512	17.76	95.78
39	530	3.75	99.53
40	29	0.20	99.73

41	10	0.07	99.80
42	28	0.20	100.00
Total	14,147		100.00

Supplemental Method S4: Description of mixed-effects regression models

The main multiple mixed-effects regression models can be summarized as follows:

	**Multi-level models**	vs. **Composite models**	
**Eq. 1.1-1.4**	$Y_{ij} = \pi_{0i} + \pi_{1i}Time_{ij} + \varepsilon_{ij}$	$\pi_{0i} = \gamma_{00} + \gamma_{0a}X_{aij} + \sum_{k=1}^{l}\gamma_{0k}Z_{ik} + \zeta_{0i}$<br>$\pi_{1i} = \gamma_{10} + \gamma_{1a}X_{aij} + \sum_{m=1}^{n}\gamma_{1m}Z_{im} + \zeta_{1i}$	$Y_{ij} = \gamma_{00} + \gamma_{0a}X_{aij} + \sum_{k=1}^{l}\gamma_{0k}Z_{ik}$<br>$+ \gamma_{10}Time_{ij} + \gamma_{1a}X_{aij}Time_{ij}$<br>$+ \sum_{m=1}^{n}\gamma_{1m}Z_{im}Time_{ij}$<br>$+ (\zeta_{0i} + \zeta_{1i}Time_{ij} + \varepsilon_{ij})$

Where Y_{ij} is the outcome (AL and components) for each individual "i" and visit "j"; π_{0i} is the level-1 intercept for individual i; π_{1i} is the level-1 slope for individual i; γ_{00} is the level-2 intercept of the random intercept π_{0i} ; γ_{10} is the level-2 intercept of the slope π_{1i} ; Z_{ik} is a vector of fixed covariates for each individual *i* that are used to predict level-1 intercepts and slopes and included baseline age (Age_{base}) among other covariates. $X_{ija,}$ represents the main predictor variables (MVD and/or DASH mean across first two visits); ζ_{0i} and ζ_{1i} are level-2 disturbances; ε_{ij} is the within-person level-1 disturbance. Of primary interest are the main effects of each exposure X_a (γ_{0a}) and their interaction with *TIME* (γ_{1a}), as described in a previous methodolgical paper.[131]

Supplemental method S5: Stata do file for main analysis

**PRELIMINARY DATA MANAGEMENT NOT SHOWN FOR SIMPLICITY, GENERAL STEPS:

STEP 0: ESTIMATE FOOD COST/MVD FOR WAVES 1 AND 3 (ALREADY COMPLETED FOR ANOTHER PROJECT).

STEP 1: PREPARE COVARIATES DATASET + AGE VARIABLES

STEP 2: MERGE BASELINE COVARIATES DATASET + AGE VARIBLES WITH DIETARY COVARIATES

STEP 3: MERGE DIETARY DASH DATA WITH DEMOGRAPHIC DATA FOR EACH OF WAVES 1 AND 3

STEP 4: MERGE FOOD COST DATA WITH DEMOGRAPHIC DATA FOR EACH OF WAVES 1 AND 3

STEP 5: MERGE FOOD COST WITH DASH DIET VARIABLES AT EACH WAVE, with demo variables

STEP 6A: MERGE WAVES 1 AND 3 (WIDE) FOR FOOD COST AND DASH DIET + DEMOGRAPHICS

STEP 6B: APPEND WAVES 1 AND 3 (LONG) FOR FOOD COST AND DASH DIET + DEMOGRAPHICS

STEP 6C: MERGE WIDE DATASET WITH REMAINING COVARIATES + AGE VARIABLES

STEP 6D: MERGE LONG DATASET WITH REMAINING COVARIATES + AGE VARIABLES

STEP 7A: DISASSEMBLE WAVES 3 AND 4 FOR ALLOSTATIC LOAD AND COMPONENTS

STEP 7B: RENAME VARIABLES OF ALLOSTATIC LOAD FILES FOR EACH WAVE (W3 AND W4)

STEP 8: MERGE LONG ALLOSTATIC LOAD DATASET WAVE 3 WITH W3 LONG DIETARY/FCOST DATA + demographics

STEP 9: MERGE DEMO FILE WITH LONG ALLOSTATIC LOAD WAVE 4

STEP 10: APPEND FILES FROM STEPS 8 AND 9

STEP 11: APPEND WAVE 1 DIET/FC LONG + DEMO on STEP 10 FILE

STEP 12: MERGE FILE IN STEP 11 with Covariates+Age dataset, Wide diet+FC files and Wide Allostatic load files

STEP 13: Re-merge with Covariates file

```
***********MAIN DATA MANAGEMENT*****************

////////////////////////////////SAMPLE SELECTIVITY///////////////////////////
cd "G:\...\DATA"

use HANDLS_Allostaticload_dietfcfinal, clear

//SAMPLE WITH COMPLETE DATA ON DEMOGRAPHICS//

capture drop sample1
gen sample1=1 if Agew1~=.
replace sample1=0 if sample1~=1

tab sample1
tab sample1 if HNDwave==1
tab sample1 if HNDwave==3
tab sample1 if HNDwave==4

//SAMPLE WITH COMPLETE DATA ON DEMOGRAPHICS + DIET+FC//


capture drop sample2
gen sample2=1 if DASH_score~=.
replace sample2=0 if sample2~=1

tab sample2
tab sample2 if HNDwave==1
tab sample2 if HNDwave==3
tab sample2 if HNDwave==4


reg Agew1 i.sample2 if HNDwave==1
tab sample2 sex if HNDwave==1, row col chi
tab sample2 race if HNDwave==1, row col chi
tab sample2 pir if HNDwave==1, row col chi
tab sample2 edubr if HNDwave==1, row col chi

//SAMPLE WITH COMPLETE DIETARY DATA AT VISITS 1 AND 2//

capture drop sample2b
gen sample2b=.
replace sample2b=1 if DASH_scorew1~=. & DASH_scorew3~=.
```

replace sample2b=0 if sample2b~=1

tab sample2b if HNDwave==1

//SAMPLE WITH COMPLETE DATA ON DEMOGRAPHICS + Allostatic load//

capture drop sample3
gen sample3=1 if allostatic_prop~=.
replace sample3=0 if sample3~=1

tab sample3
tab sample3 if HNDwave==1
tab sample3 if HNDwave==3
tab sample3 if HNDwave==4

//SAMPLE WITH COMPLETE DATA ON DEMOGRAPHICS + Allostatic load at eithe wave or Diet at either wave//

capture drop sample4
gen sample4=1 if allostatic_propw3~=. | DASH_scorew1~=. | DASH_scorew3~=. | allostatic_propw4~=.
replace sample4=0 if sample4~=1

tab sample4
tab sample4 if HNDwave==1
tab sample4 if HNDwave==3
tab sample4 if HNDwave==4

//SAMPLE WITH COMPLETE DATA ON DEMOGRAPHICS + Allostatic load at eithe wave and Diet at either wave//
capture drop sample4b
gen sample4b=1 if allostatic_propw3~=. & DASH_scorew1~=. | allostatic_propw3~=. & DASH_scorew3~=. | allostatic_propw4~=. & DASH_scorew1~=. | allostatic_propw4~=. & DASH_scorew3~=.
replace sample4b=0 if sample4b~=1

tab sample4b
tab sample4b if HNDwave==1
tab sample4b if HNDwave==3
tab sample4b if HNDwave==4

//SAMPLE WITH COMPLETE DATA ON DEMOGRAPHICS + Allostatic load at wave 3 or 4 + Diet at wave at wave 1 & 3 //
capture drop sample5

```
gen sample5=1 if allostatic_propw3~=. & DASH_scorew1~=. & DASH_scorew3~=. | allostatic_propw4~=. & DASH_scorew1~=. & DASH_scorew3~=.
replace sample5=0 if sample5~=1

tab sample5
tab sample5 if HNDwave==1
tab sample5 if HNDwave==3
tab sample5 if HNDwave==4

//SAMPLE WITH COMPLETE DATA ON DEMOGRAPHICS + Allostatic load at wave 4 + Diet at wave at wave 1 & 3 //
capture drop sample6
gen sample6=1 if allostatic_propw4~=. & DASH_scorew1~=. & DASH_scorew3~=.
replace sample6=0 if sample6~=1

tab sample6
tab sample6 if HNDwave==1
tab sample6 if HNDwave==3
tab sample6 if HNDwave==4


//SAMPLE WITH COMPLETE DATA ON DEMOGRAPHICS + Allostatic load at waves 3 and 4 + Diet at wave at wave 1 & 3 + Covariates: Agew1 sex race pir edubr employed wrattbr smoke currdrugs bmi SRHbr //
capture drop sample7
gen sample7=1 if allostatic_propw3~=. & allostatic_propw4~=. & DASH_scorew1~=. & DASH_scorew3~=.
replace sample7=0 if sample7~=1

tab sample7
tab sample7 if HNDwave==1
tab sample7 if HNDwave==3
tab sample7 if HNDwave==4


//FINAL SAMPLE for MIXED MODELS: complete on demographics, allostatic load at waves 3 or 4, diet at waves 1 and 3 + Covariates ///

**Covariates: edubr employed wrattbr smoke currdrugs bmi SRHbr
**Mean energy intake, engery from grocery stores (w1 and w3): energystoresw1 energystoresw3 kcalw1 kcalw3

capture drop kcal_w1w3mean
gen kcal_w1w3mean=(kcalw1+kcalw3)/2
su kcal_w1w3mean

capture drop energystoresw1w3mean
gen energystoresw1w3mean=(energystoresw1+energystoresw3)/2
```

```
su energystoresw1w3mean

capture drop sample_cov
gen sample_cov=.
replace sample_cov=1 if edubr~=. & employed~=. & wrattbr~=. & smoke~=. & currdrugs~=. & bmi~=. & SRHbr~=.
replace sample_cov=0 if sample_cov~=1

tab sample_cov

capture drop sample8
gen sample8=.
replace sample8=1 if (allostatic_propw3~=. & DASH_scorew1~=. & DASH_scorew3~=. & HNDwave==3 & sample_cov==1 | allostatic_propw3~=. & DASH_scorew1~=. & DASH_scorew3~=. & HNDwave==4 & sample_cov==1) | (allostatic_propw4~=. & DASH_scorew1~=. & DASH_scorew3~=. & HNDwave==3 & sample_cov==1 | allostatic_propw4~=. & DASH_scorew1~=. & DASH_scorew3~=. & HNDwave==4 & sample_cov==1 )
replace sample8=0 if sample8~=1 & HNDwave==3 | sample8~=1 & HNDwave==4

tab sample8
tab sample8 if HNDwave==1
tab sample8 if HNDwave==3
tab sample8 if HNDwave==4

reg Agew1 i.sample8 if HNDwave==3
tab sample8 sex if HNDwave==3, row col chi
tab sample8 race if HNDwave==3, row col chi
tab sample8 pir if HNDwave==3, row col chi
tab sample8 edubr if HNDwave==3, row col chi

//FINAL SAMPLE for SEM MODELS: complete on demographics, allostatic load at wave 4, diet at waves 1 and 3 + Covariates ///

capture drop sample9
gen sample9=.
replace sample9=1 if allostatic_propw4~=. & DASH_scorew1~=. & DASH_scorew3~=. & HNDwave==4 & sample_cov==1
replace sample9=0 if sample9~=1 & HNDwave==4

tab sample9
tab sample9 if HNDwave==1
tab sample9 if HNDwave==3
```

```
tab sample9 if HNDwave==4

reg Agew1 i.sample9 if HNDwave==4
tab sample9 sex if HNDwave==4, row col chi
tab sample9 race if HNDwave==4, row col chi
tab sample9 pir if HNDwave==4, row col chi
tab sample9 edubr if HNDwave==4, row col chi

save, replace

/////GENERATE THE TWO INVERSE MILLS RATIOS//
cd "G:\...\DATA"

use HANDLS_Allostaticload_dietfcfinal, clear

**MIXED MODELS**
xi:probit sample8 Agew1 i.race pir sex if HNDwave==3 | HNDwave==4

capture drop p1mixed
predict p1mixed, xb

capture drop phimixed
capture drop caphimixed
capture drop invmillsmixed

gen phimixed=(1/sqrt(2*_pi))*exp(-(p1mixed^2/2))

egen caphimixed=std(p1mixed)

capture drop invmillsmixed
gen invmillsmixed=phimixed/caphimixed



**SEM MODELS**
xi:probit sample9 Agew1 i.race pir sex



capture drop p1sem
predict p1sem, xb

capture drop phisem
capture drop caphisem
capture drop invmillssem
```

```
gen phisem=(1/sqrt(2*_pi))*exp(-(p1sem^2/2))

egen caphisem=std(p1sem)

capture drop invmillssem
gen invmillssem=phisem/caphisem


save, replace

//////CREATE A PARTICIPANT FLOWCHART///

**Initial sample --> Sample with dietary data at wave 1 --> Sample with dietary data at wave 3 --> Sample with allostatic load at waves 3 or 4 --> Sample with both dietary data and allostatic load data at wave 3 or 4 -->
**--> Sample with both dietary data and allostatic load data at wave 3 or 4 + Covariates (Mixed models) --> Sample with both dietary data and allostatic load data at wave 4 + Covariates (SEM)


///Create TIME variable////

capture drop timew3w4
gen timew3w4=.
replace timew3w4=(Agew4-Agew3) if HNDwave==4
replace timew3w4=0 if HNDwave==3

su timew3w4 if HNDwave==4
su timew3w4 if HNDwave==3

su timew3w4 if sample8==1
su timew3w4 if sample8==1 & HNDwave==3
su timew3w4 if sample8==1 & HNDwave==4



su timew3w4 if sample9==1

save, replace

///////////////////MIXED MODELS: Means of DASH diet and MVD vs. baseline and change in Allostatic load////////////////////
cd "G:\...\DATA"

use HANDLS_Allostaticload_dietfcfinal, clear
```

```
**Main exposures and centering of continuous predictors**
capture drop food_price_USAfinW1W3mean
gen food_price_BALTfinW1W3mean=(food_price_BALTfinWave1+food_price_BALTfinWave1)/2

su food_price_BALTfinW1W3mean if sample8==1
su food_price_BALTfinW1W3mean if sample9==1

capture drop food_price_BALTfinW1W3mean_C
gen food_price_BALTfinW1W3mean_C=food_price_BALTfinW1W3mean-6.3

capture drop food_price_USAfinW1W3meantert
xtile food_price_BALTfinW1W3meantert=food_price_BALTfinW1W3mean if sample8==1, nq(3)



capture drop DASH_scorew1w3mean
gen DASH_scorew1w3mean=(DASH_scorew1+DASH_scorew3)/2

su DASH_scorew1w3mean if sample8==1
su DASH_scorew1w3mean if sample9==1

capture drop DASH_scorew1w3mean_C
gen DASH_scorew1w3mean_C=DASH_scorew1w3mean-1.74



capture drop kcalw1w3mean
gen kcalw1w3mean=(kcalw1+kcalw3)/2

su kcalw1w3mean if sample8==1
su kcalw1w3mean if sample9==1

capture drop kcalw1w3mean_C
gen kcalw1w3mean_C=kcalw1w3mean-2030



su energystoresw1w3mean if sample8==1
su energystoresw1w3mean if sample9==1



capture drop energystoresw1w3mean_C
gen energystoresw1w3mean_C=energystoresw1w3mean-1550



save, replace
```

```
su bmi if sample8==1
su bmi if sample9==1

capture drop bmi_C
gen bmi_C=bmi-30


su Agew1 if sample8==1
su Agew1 if sample9==1

capture drop Agew1_C
gen Agew1_C=Agew1-48


su Agew3 if sample8==1
su Agew3 if sample9==1

capture drop Agew3_C
gen Agew3_C=Agew3-53


save, replace






//////////////////////////////////TABLE 1/////////////////////////////////////////////

cd "G:\...\DATA"

use HANDLS_Allostaticload_dietfcfinal, clear

capture drop finalsample
gen finalsample=.
replace finalsample=sample9


capture drop allostatic_propdelta
gen allostatic_propdelta=(allostatic_propw4-allostatic_propw3)/(Agew4-Agew3)
```

```
su allostatic_propdelta


capture drop allostatic_propmean
gen allostatic_propmean=(allostatic_propw4+allostatic_propw3)/2

su allostatic_propmean


save, replace

**Overall**

mean Agew1 if finalsample==1
mean Agew3 if finalsample==1
mean Agew4 if finalsample==1
tab sex if finalsample==1
tab race if finalsample==1
tab pir if finalsample==1
tab edubr if finalsample==1
tab employed if finalsample==1
tab wrattbr if finalsample==1
tab smoke if finalsample==1
tab currdrugs if finalsample==1
mean bmi if finalsample==1
mean SRHbr if finalsample==1
mean food_price_BALTfinWave1 if finalsample==1
mean food_price_BALTfinWave3 if finalsample==1
mean food_price_BALTfinW1W3mean if finalsample==1
mean kcalw1 if finalsample==1
mean kcalw3 if finalsample==1
mean kcalw1w3mean if finalsample==1
mean energystoresw1 if finalsample==1
mean energystoresw3 if finalsample==1
mean energystoresw1w3mean if finalsample==1

mean DASH_scorew1 if finalsample==1
mean DASH_scorew3 if finalsample==1
mean DASH_scorew1w3mean if finalsample==1

mean allostatic_propw3 if finalsample==1
mean allostatic_propw4 if finalsample==1
mean allostatic_propdelta if finalsample==1
mean allostatic_propmean if finalsample==1
```

//MEAN FOOD COST TERTILES//

su food_price_BALTfinW1W3mean if finalsample==1 & food_price_BALTfinW1W3meantert==1
su food_price_BALTfinW1W3mean if finalsample==1 & food_price_BALTfinW1W3meantert==2
su food_price_BALTfinW1W3mean if finalsample==1 & food_price_BALTfinW1W3meantert==3

**Lowest mean in FC/Baltimore tertile:
mean Agew1 if finalsample==1 & food_price_BALTfinW1W3meantert==1
mean Agew3 if finalsample==1 & food_price_BALTfinW1W3meantert==1
mean Agew4 if finalsample==1 & food_price_BALTfinW1W3meantert==1
tab sex if finalsample==1 & food_price_BALTfinW1W3meantert==1
tab race if finalsample==1 & food_price_BALTfinW1W3meantert==1
tab pir if finalsample==1 & food_price_BALTfinW1W3meantert==1
tab edubr if finalsample==1 & food_price_BALTfinW1W3meantert==1
tab employed if finalsample==1 & food_price_BALTfinW1W3meantert==1
tab wrattbr if finalsample==1 & food_price_BALTfinW1W3meantert==1
tab smoke if finalsample==1 & food_price_BALTfinW1W3meantert==1
tab currdrugs if finalsample==1 & food_price_BALTfinW1W3meantert==1
mean bmi if finalsample==1 & food_price_BALTfinW1W3meantert==1
prop SRHbr if finalsample==1 & food_price_BALTfinW1W3meantert==1
mean food_price_BALTfinWave1 if finalsample==1 & food_price_BALTfinW1W3meantert==1
mean food_price_BALTfinWave3 if finalsample==1 & food_price_BALTfinW1W3meantert==1
mean food_price_BALTfinW1W3mean if finalsample==1 & food_price_BALTfinW1W3meantert==1
mean kcalw1 if finalsample==1 & food_price_BALTfinW1W3meantert==1
mean kcalw3 if finalsample==1 & food_price_BALTfinW1W3meantert==1
mean kcalw1w3mean if finalsample==1 & food_price_BALTfinW1W3meantert==1
mean energystoresw1 if finalsample==1 & food_price_BALTfinW1W3meantert==1
mean energystoresw3 if finalsample==1 & food_price_BALTfinW1W3meantert==1
mean energystoresw1w3mean if finalsample==1 & food_price_BALTfinW1W3meantert==1
** DASH_scorew1 DASH_scorew3 DASH_scorew1w3 allostatic_propw3 allostatic_propw4 allostatic_propdelta

mean DASH_scorew1 if finalsample==1 & food_price_BALTfinW1W3meantert==1
mean DASH_scorew3 if finalsample==1 & food_price_BALTfinW1W3meantert==1
mean DASH_scorew1w3mean if finalsample==1 & food_price_BALTfinW1W3meantert==1

mean allostatic_propw3 if finalsample==1 & food_price_BALTfinW1W3meantert==1
mean allostatic_propw4 if finalsample==1 & food_price_BALTfinW1W3meantert==1
mean allostatic_propdelta if finalsample==1 & food_price_BALTfinW1W3meantert==1
mean allostatic_propmean if finalsample==1 & food_price_BALTfinW1W3meantert==1

**Middle mean in FC/Baltimore tertile:

mean Agew1 if finalsample==1 & food_price_BALTfinW1W3meantert==2

mean Agew3 if finalsample==1 & food_price_BALTfinW1W3meantert==2

mean Agew4 if finalsample==1 & food_price_BALTfinW1W3meantert==2

tab sex if finalsample==1 & food_price_BALTfinW1W3meantert==2

tab race if finalsample==1 & food_price_BALTfinW1W3meantert==2

tab pir if finalsample==1 & food_price_BALTfinW1W3meantert==2

tab edubr if finalsample==1 & food_price_BALTfinW1W3meantert==2

tab employed if finalsample==1 & food_price_BALTfinW1W3meantert==2

tab wrattbr if finalsample==1 & food_price_BALTfinW1W3meantert==2

tab smoke if finalsample==1 & food_price_BALTfinW1W3meantert==2

tab currdrugs if finalsample==1 & food_price_BALTfinW1W3meantert==2

mean bmi if finalsample==1 & food_price_BALTfinW1W3meantert==2

prop SRHbr if finalsample==1 & food_price_BALTfinW1W3meantert==2

mean food_price_BALTfinWave1 if finalsample==1 & food_price_BALTfinW1W3meantert==2

mean food_price_BALTfinWave3 if finalsample==1 & food_price_BALTfinW1W3meantert==2

mean food_price_BALTfinW1W3mean if finalsample==1 & food_price_BALTfinW1W3meantert==2

mean kcalw1 if finalsample==1 & food_price_BALTfinW1W3meantert==2

mean kcalw3 if finalsample==1 & food_price_BALTfinW1W3meantert==2

mean kcalw1w3mean if finalsample==1 & food_price_BALTfinW1W3meantert==2

mean energystoresw1 if finalsample==1 & food_price_BALTfinW1W3meantert==2

mean energystoresw3 if finalsample==1 & food_price_BALTfinW1W3meantert==2

mean energystoresw1w3mean if finalsample==1 & food_price_BALTfinW1W3meantert==2

mean DASH_scorew1 if finalsample==1 & food_price_BALTfinW1W3meantert==2

mean DASH_scorew3 if finalsample==1 & food_price_BALTfinW1W3meantert==2

mean DASH_scorew1w3mean if finalsample==1 & food_price_BALTfinW1W3meantert==2

mean allostatic_propw3 if finalsample==1 & food_price_BALTfinW1W3meantert==2

mean allostatic_propw4 if finalsample==1 & food_price_BALTfinW1W3meantert==2

mean allostatic_propdelta if finalsample==1 & food_price_BALTfinW1W3meantert==2

mean allostatic_propmean if finalsample==1 & food_price_BALTfinW1W3meantert==2

**Upper mean in FC/Baltimore tertile:

mean Agew1 if finalsample==1 & food_price_BALTfinW1W3meantert==3

mean Agew3 if finalsample==1 & food_price_BALTfinW1W3meantert==3

mean Agew4 if finalsample==1 & food_price_BALTfinW1W3meantert==3

tab sex if finalsample==1 & food_price_BALTfinW1W3meantert==3

tab race if finalsample==1 & food_price_BALTfinW1W3meantert==3

tab pir if finalsample==1 & food_price_BALTfinW1W3meantert==3

```
tab edubr if finalsample==1 & food_price_BALTfinW1W3meantert==3
tab employed if finalsample==1 & food_price_BALTfinW1W3meantert==3
tab wrattbr if finalsample==1 & food_price_BALTfinW1W3meantert==3
tab smoke if finalsample==1 & food_price_BALTfinW1W3meantert==3
tab currdrugs if finalsample==1 & food_price_BALTfinW1W3meantert==3
mean bmi if finalsample==1 & food_price_BALTfinW1W3meantert==3
prop SRHbr if finalsample==1 & food_price_BALTfinW1W3meantert==3
mean food_price_BALTfinWave1 if finalsample==1 & food_price_BALTfinW1W3meantert==3
mean food_price_BALTfinWave3 if finalsample==1 & food_price_BALTfinW1W3meantert==3
mean food_price_BALTfinW1W3mean if finalsample==1 & food_price_BALTfinW1W3meantert==3
mean kcalw1 if finalsample==1 & food_price_BALTfinW1W3meantert==3
mean kcalw3 if finalsample==1 & food_price_BALTfinW1W3meantert==3
mean kcalw1w3mean if finalsample==1 & food_price_BALTfinW1W3meantert==3
mean energystoresw1 if finalsample==1 & food_price_BALTfinW1W3meantert==3
mean energystoresw3 if finalsample==1 & food_price_BALTfinW1W3meantert==3
mean energystoresw1w3mean if finalsample==1 & food_price_BALTfinW1W3meantert==3

mean DASH_scorew1 if finalsample==1 & food_price_BALTfinW1W3meantert==3
mean DASH_scorew3 if finalsample==1 & food_price_BALTfinW1W3meantert==3
mean DASH_scorew1w3mean if finalsample==1 & food_price_BALTfinW1W3meantert==3

mean allostatic_propw3 if finalsample==1 & food_price_BALTfinW1W3meantert==3
mean allostatic_propw4 if finalsample==1 & food_price_BALTfinW1W3meantert==3
mean allostatic_propdelta if finalsample==1 & food_price_BALTfinW1W3meantert==3
mean allostatic_propmean if finalsample==1 & food_price_BALTfinW1W3meantert==3


**Difference in characterestics by tertile of food cost/Baltimore**

oneway Agew1 food_price_BALTfinW1W3meantert if finalsample==1, bon
oneway Agew3 food_price_BALTfinW1W3meantert if finalsample==1, bon
oneway Agew4 food_price_BALTfinW1W3meantert if finalsample==1, bon
tab sex food_price_BALTfinW1W3meantert if finalsample==1, row col chi
tab race food_price_BALTfinW1W3meantert if finalsample==1, row col chi
tab pir food_price_BALTfinW1W3meantert if finalsample==1, row col chi
tab edubr food_price_BALTfinW1W3meantert if finalsample==1, row col chi
tab employed food_price_BALTfinW1W3meantert if finalsample==1, row col chi
tab wrattbr food_price_BALTfinW1W3meantert if finalsample==1, row col chi
tab smoke food_price_BALTfinW1W3meantert if finalsample==1, row col chi
tab currdrugs food_price_BALTfinW1W3meantert if finalsample==1, row col chi
tab SRHbr food_price_BALTfinW1W3meantert if finalsample==1, row col chi
oneway bmi food_price_BALTfinW1W3meantert if finalsample==1, bon
oneway kcalw1 food_price_BALTfinW1W3meantert if finalsample==1 , bon
oneway kcalw3 food_price_BALTfinW1W3meantert if finalsample==1 , bon
```

```
oneway kcalw1w3mean food_price_BALTfinW1W3meantert if finalsample==1 , bon
oneway energystoresw1 food_price_BALTfinW1W3meantert if finalsample==1 , bon
oneway energystoresw3 food_price_BALTfinW1W3meantert if finalsample==1 , bon
oneway energystoresw1w3mean food_price_BALTfinW1W3meantert if finalsample==1 , bon
oneway food_price_BALTfinWave1 food_price_BALTfinW1W3meantert if finalsample==1, bon
oneway food_price_BALTfinWave3 food_price_BALTfinW1W3meantert if finalsample==1, bon
oneway food_price_BALTfinW1W3mean food_price_BALTfinW1W3meantert if finalsample==1, bon


save, replace

**DASH_scorew1w3 DASH_scorew1 DASH_scorew3 allostatic_propw3 allostatic_propw4 allostatic_propdelta


oneway DASH_scorew1 food_price_BALTfinW1W3meantert if finalsample==1, bon
oneway DASH_scorew3 food_price_BALTfinW1W3meantert if finalsample==1, bon
oneway DASH_scorew1w3mean food_price_BALTfinW1W3meantert if finalsample==1, bon


oneway allostatic_propw3 food_price_BALTfinW1W3meantert if finalsample==1, bon
oneway allostatic_propw4 food_price_BALTfinW1W3meantert if finalsample==1, bon
oneway allostatic_propdelta food_price_BALTfinW1W3meantert if finalsample==1, bon
oneway allostatic_propmean food_price_BALTfinW1W3meantert if finalsample==1, bon



////////////////////////////////////////TABLE 2/////////////////////////////////


cd "G:\...\DATA"

use HANDLS_Allostaticload_dietfcfinal, clear

////////////////////////////////////////////////CRUDE MODEL///////////////////////////////////

**Compute means of DASH components**
//DASH_SatFatw1 DASH_Fatw1 DASH_proteinw1 DASH_cholesterolw1 DASH_fiberw1 DASH_Magnesiumw1 DASH_calciumw1 DASH_potassiumw1 DASH_Sodiumw1//
//DASH_SatFatw3 DASH_Fatw3 Dash_protein_W3 DASH_cholesterolw3 DASH_fiberw3 DASH_Magnesiumw3 DASH_calciumw3 DASH_potassiumw3 DASH_Sodiumw3//


capture drop DASH_SatFatw1w3mean
gen DASH_SatFatw1w3mean=(DASH_SatFatw1+DASH_SatFatw3)/2
```

```
capture drop DASH_Fatw1w3mean
gen DASH_Fatw1w3mean=(DASH_Fatw1+DASH_Fatw3)/2

capture drop Dash_protein_W1W3mean
gen Dash_protein_W1W3mean=(DASH_proteinw1+Dash_protein_W3)/2

capture drop DASH_cholesterolw1w3
gen DASH_cholesterolw1w3=(DASH_cholesterolw1+DASH_cholesterolw3)/2

capture drop DASH_fiberw1w3
gen DASH_fiberw1w3=(DASH_fiberw1+DASH_fiberw3)/2


capture drop DASH_Magnesiumw1w3mean
gen DASH_Magnesiumw1w3mean=(DASH_Magnesiumw1+DASH_Magnesiumw3)/2

capture drop DASH_calciumw1w3mean
gen DASH_calciumw1w3mean=(DASH_calciumw1+DASH_calciumw3)/2

capture drop DASH_potassiumw1w3mean
gen DASH_potassiumw1w3mean=(DASH_potassiumw1+DASH_potassiumw3)/2

capture drop DASH_Sodiumw1w3mean
gen DASH_Sodiumw1w3mean=(DASH_Sodiumw1+DASH_Sodiumw3)/2


save, replace

*******P-value comparing 2nd to 1st and 3rd to 1st*****

reg DASH_scorew1w3mean i.food_price_BALTfinW1W3meantert if finalsample==1
reg DASH_scorew1w3mean i.food_price_BALTfinW1W3meantert if finalsample==1 & sex==1
reg DASH_scorew1w3mean i.food_price_BALTfinW1W3meantert if finalsample==1 & sex==0
reg DASH_scorew1w3mean i.food_price_BALTfinW1W3meantert if finalsample==1 & race==0
reg DASH_scorew1w3mean i.food_price_BALTfinW1W3meantert if finalsample==1 & race==1
reg DASH_scorew1w3mean i.food_price_BALTfinW1W3meantert if finalsample==1 & pir==0
reg DASH_scorew1w3mean i.food_price_BALTfinW1W3meantert if finalsample==1 & pir==1


reg DASH_SatFatw1w3mean i.food_price_BALTfinW1W3meantert if finalsample==1
reg DASH_SatFatw1w3mean i.food_price_BALTfinW1W3meantert if finalsample==1 & sex==1
```

```
reg DASH_SatFatw1w3mean i.food_price_BALTfinW1W3meantert if finalsample==1 & sex==0
reg DASH_SatFatw1w3mean i.food_price_BALTfinW1W3meantert if finalsample==1 & race==0
reg DASH_SatFatw1w3mean i.food_price_BALTfinW1W3meantert if finalsample==1 & race==1
reg DASH_SatFatw1w3mean i.food_price_BALTfinW1W3meantert if finalsample==1 & pir==0
reg DASH_SatFatw1w3mean i.food_price_BALTfinW1W3meantert if finalsample==1 & pir==1


reg DASH_Fatw1w3mean i.food_price_BALTfinW1W3meantert if finalsample==1
reg DASH_Fatw1w3mean i.food_price_BALTfinW1W3meantert if finalsample==1 & sex==1
reg DASH_Fatw1w3mean i.food_price_BALTfinW1W3meantert if finalsample==1 & sex==0
reg DASH_Fatw1w3mean i.food_price_BALTfinW1W3meantert if finalsample==1 & race==0
reg DASH_Fatw1w3mean i.food_price_BALTfinW1W3meantert if finalsample==1 & race==1
reg DASH_Fatw1w3mean i.food_price_BALTfinW1W3meantert if finalsample==1 & pir==0
reg DASH_Fatw1w3mean i.food_price_BALTfinW1W3meantert if finalsample==1 & pir==1


reg Dash_protein_W1W3mean i.food_price_BALTfinW1W3meantert if finalsample==1
reg Dash_protein_W1W3mean i.food_price_BALTfinW1W3meantert if finalsample==1 & sex==1
reg Dash_protein_W1W3mean i.food_price_BALTfinW1W3meantert if finalsample==1 & sex==0
reg Dash_protein_W1W3mean i.food_price_BALTfinW1W3meantert if finalsample==1 & race==0
reg Dash_protein_W1W3mean i.food_price_BALTfinW1W3meantert if finalsample==1 & race==1
reg Dash_protein_W1W3mean i.food_price_BALTfinW1W3meantert if finalsample==1 & pir==0
reg Dash_protein_W1W3mean i.food_price_BALTfinW1W3meantert if finalsample==1 & pir==1


reg DASH_cholesterolw1w3 i.food_price_BALTfinW1W3meantert if finalsample==1
reg DASH_cholesterolw1w3 i.food_price_BALTfinW1W3meantert if finalsample==1 & sex==1
reg DASH_cholesterolw1w3 i.food_price_BALTfinW1W3meantert if finalsample==1 & sex==0
reg DASH_cholesterolw1w3 i.food_price_BALTfinW1W3meantert if finalsample==1 & race==0
reg DASH_cholesterolw1w3 i.food_price_BALTfinW1W3meantert if finalsample==1 & race==1
reg DASH_cholesterolw1w3 i.food_price_BALTfinW1W3meantert if finalsample==1 & pir==0
reg DASH_cholesterolw1w3 i.food_price_BALTfinW1W3meantert if finalsample==1 & pir==1


reg DASH_fiberw1w3 i.food_price_BALTfinW1W3meantert if finalsample==1
reg DASH_fiberw1w3 i.food_price_BALTfinW1W3meantert if finalsample==1 & sex==1
reg DASH_fiberw1w3 i.food_price_BALTfinW1W3meantert if finalsample==1 & sex==0
reg DASH_fiberw1w3 i.food_price_BALTfinW1W3meantert if finalsample==1 & race==0
reg DASH_fiberw1w3 i.food_price_BALTfinW1W3meantert if finalsample==1 & race==1
```

```
reg DASH_fiberw1w3 i.food_price_BALTfinW1W3meantert if finalsample==1 & pir==0
reg DASH_fiberw1w3 i.food_price_BALTfinW1W3meantert if finalsample==1 & pir==1


reg DASH_Magnesiumw1w3mean i.food_price_BALTfinW1W3meantert if finalsample==1
reg DASH_Magnesiumw1w3mean i.food_price_BALTfinW1W3meantert if finalsample==1 & sex==1
reg DASH_Magnesiumw1w3mean i.food_price_BALTfinW1W3meantert if finalsample==1 & sex==0
reg DASH_Magnesiumw1w3mean i.food_price_BALTfinW1W3meantert if finalsample==1 & race==0
reg DASH_Magnesiumw1w3mean i.food_price_BALTfinW1W3meantert if finalsample==1 & race==1
reg DASH_Magnesiumw1w3mean i.food_price_BALTfinW1W3meantert if finalsample==1 & pir==0
reg DASH_Magnesiumw1w3mean i.food_price_BALTfinW1W3meantert if finalsample==1 & pir==1


reg DASH_calciumw1w3mean i.food_price_BALTfinW1W3meantert if finalsample==1
reg DASH_calciumw1w3mean i.food_price_BALTfinW1W3meantert if finalsample==1 & sex==1
reg DASH_calciumw1w3mean i.food_price_BALTfinW1W3meantert if finalsample==1 & sex==0
reg DASH_calciumw1w3mean i.food_price_BALTfinW1W3meantert if finalsample==1 & race==0
reg DASH_calciumw1w3mean i.food_price_BALTfinW1W3meantert if finalsample==1 & race==1
reg DASH_calciumw1w3mean i.food_price_BALTfinW1W3meantert if finalsample==1 & pir==0
reg DASH_calciumw1w3mean i.food_price_BALTfinW1W3meantert if finalsample==1 & pir==1


reg DASH_potassiumw1w3mean i.food_price_BALTfinW1W3meantert if finalsample==1
reg DASH_potassiumw1w3mean i.food_price_BALTfinW1W3meantert if finalsample==1 & sex==1
reg DASH_potassiumw1w3mean i.food_price_BALTfinW1W3meantert if finalsample==1 & sex==0
reg DASH_potassiumw1w3mean i.food_price_BALTfinW1W3meantert if finalsample==1 & race==0
reg DASH_potassiumw1w3mean i.food_price_BALTfinW1W3meantert if finalsample==1 & race==1
reg DASH_potassiumw1w3mean i.food_price_BALTfinW1W3meantert if finalsample==1 & pir==0
reg DASH_potassiumw1w3mean i.food_price_BALTfinW1W3meantert if finalsample==1 & pir==1


reg DASH_Sodiumw1w3mean i.food_price_BALTfinW1W3meantert if finalsample==1
reg DASH_Sodiumw1w3mean i.food_price_BALTfinW1W3meantert if finalsample==1 & sex==1
reg DASH_Sodiumw1w3mean i.food_price_BALTfinW1W3meantert if finalsample==1 & sex==0
reg DASH_Sodiumw1w3mean i.food_price_BALTfinW1W3meantert if finalsample==1 & race==0
reg DASH_Sodiumw1w3mean i.food_price_BALTfinW1W3meantert if finalsample==1 & race==1
reg DASH_Sodiumw1w3mean i.food_price_BALTfinW1W3meantert if finalsample==1 & pir==0
reg DASH_Sodiumw1w3mean i.food_price_BALTfinW1W3meantert if finalsample==1 & pir==1

*******P-trend*****
```

```
reg DASH_scorew1w3mean food_price_BALTfinW1W3meantert if finalsample==1
reg DASH_scorew1w3mean food_price_BALTfinW1W3meantert if finalsample==1 & sex==1
reg DASH_scorew1w3mean food_price_BALTfinW1W3meantert if finalsample==1 & sex==0
reg DASH_scorew1w3mean food_price_BALTfinW1W3meantert if finalsample==1 & race==0
reg DASH_scorew1w3mean food_price_BALTfinW1W3meantert if finalsample==1 & race==1
reg DASH_scorew1w3mean food_price_BALTfinW1W3meantert if finalsample==1 & pir==0
reg DASH_scorew1w3mean food_price_BALTfinW1W3meantert if finalsample==1 & pir==1


reg DASH_SatFatw1w3mean food_price_BALTfinW1W3meantert if finalsample==1
reg DASH_SatFatw1w3mean food_price_BALTfinW1W3meantert if finalsample==1 & sex==1
reg DASH_SatFatw1w3mean food_price_BALTfinW1W3meantert if finalsample==1 & sex==0
reg DASH_SatFatw1w3mean food_price_BALTfinW1W3meantert if finalsample==1 & race==0
reg DASH_SatFatw1w3mean food_price_BALTfinW1W3meantert if finalsample==1 & race==1
reg DASH_SatFatw1w3mean food_price_BALTfinW1W3meantert if finalsample==1 & pir==0
reg DASH_SatFatw1w3mean food_price_BALTfinW1W3meantert if finalsample==1 & pir==1


reg DASH_Fatw1w3mean food_price_BALTfinW1W3meantert if finalsample==1
reg DASH_Fatw1w3mean food_price_BALTfinW1W3meantert if finalsample==1 & sex==1
reg DASH_Fatw1w3mean food_price_BALTfinW1W3meantert if finalsample==1 & sex==0
reg DASH_Fatw1w3mean food_price_BALTfinW1W3meantert if finalsample==1 & race==0
reg DASH_Fatw1w3mean food_price_BALTfinW1W3meantert if finalsample==1 & race==1
reg DASH_Fatw1w3mean food_price_BALTfinW1W3meantert if finalsample==1 & pir==0
reg DASH_Fatw1w3mean food_price_BALTfinW1W3meantert if finalsample==1 & pir==1


reg Dash_protein_W1W3mean food_price_BALTfinW1W3meantert if finalsample==1
reg Dash_protein_W1W3mean food_price_BALTfinW1W3meantert if finalsample==1 & sex==1
reg Dash_protein_W1W3mean food_price_BALTfinW1W3meantert if finalsample==1 & sex==0
reg Dash_protein_W1W3mean food_price_BALTfinW1W3meantert if finalsample==1 & race==0
reg Dash_protein_W1W3mean food_price_BALTfinW1W3meantert if finalsample==1 & race==1
reg Dash_protein_W1W3mean food_price_BALTfinW1W3meantert if finalsample==1 & pir==0
reg Dash_protein_W1W3mean food_price_BALTfinW1W3meantert if finalsample==1 & pir==1


reg DASH_cholesterolw1w3 food_price_BALTfinW1W3meantert if finalsample==1
reg DASH_cholesterolw1w3 food_price_BALTfinW1W3meantert if finalsample==1 & sex==1
reg DASH_cholesterolw1w3 food_price_BALTfinW1W3meantert if finalsample==1 & sex==0
```

```
reg DASH_cholesterolw1w3 food_price_BALTfinW1W3meantert if finalsample==1 & race==0
reg DASH_cholesterolw1w3 food_price_BALTfinW1W3meantert if finalsample==1 & race==1
reg DASH_cholesterolw1w3 food_price_BALTfinW1W3meantert if finalsample==1 & pir==0
reg DASH_cholesterolw1w3 food_price_BALTfinW1W3meantert if finalsample==1 & pir==1



reg DASH_fiberw1w3 food_price_BALTfinW1W3meantert if finalsample==1
reg DASH_fiberw1w3 food_price_BALTfinW1W3meantert if finalsample==1 & sex==1
reg DASH_fiberw1w3 food_price_BALTfinW1W3meantert if finalsample==1 & sex==0
reg DASH_fiberw1w3 food_price_BALTfinW1W3meantert if finalsample==1 & race==0
reg DASH_fiberw1w3 food_price_BALTfinW1W3meantert if finalsample==1 & race==1
reg DASH_fiberw1w3 food_price_BALTfinW1W3meantert if finalsample==1 & pir==0
reg DASH_fiberw1w3 food_price_BALTfinW1W3meantert if finalsample==1 & pir==1


reg DASH_Magnesiumw1w3mean food_price_BALTfinW1W3meantert if finalsample==1
reg DASH_Magnesiumw1w3mean food_price_BALTfinW1W3meantert if finalsample==1 & sex==1
reg DASH_Magnesiumw1w3mean food_price_BALTfinW1W3meantert if finalsample==1 & sex==0
reg DASH_Magnesiumw1w3mean food_price_BALTfinW1W3meantert if finalsample==1 & race==0
reg DASH_Magnesiumw1w3mean food_price_BALTfinW1W3meantert if finalsample==1 & race==1
reg DASH_Magnesiumw1w3mean food_price_BALTfinW1W3meantert if finalsample==1 & pir==0
reg DASH_Magnesiumw1w3mean food_price_BALTfinW1W3meantert if finalsample==1 & pir==1



reg DASH_calciumw1w3mean food_price_BALTfinW1W3meantert if finalsample==1
reg DASH_calciumw1w3mean food_price_BALTfinW1W3meantert if finalsample==1 & sex==1
reg DASH_calciumw1w3mean food_price_BALTfinW1W3meantert if finalsample==1 & sex==0
reg DASH_calciumw1w3mean food_price_BALTfinW1W3meantert if finalsample==1 & race==0
reg DASH_calciumw1w3mean food_price_BALTfinW1W3meantert if finalsample==1 & race==1
reg DASH_calciumw1w3mean food_price_BALTfinW1W3meantert if finalsample==1 & pir==0
reg DASH_calciumw1w3mean food_price_BALTfinW1W3meantert if finalsample==1 & pir==1



reg DASH_potassiumw1w3mean food_price_BALTfinW1W3meantert if finalsample==1
reg DASH_potassiumw1w3mean food_price_BALTfinW1W3meantert if finalsample==1 & sex==1
reg DASH_potassiumw1w3mean food_price_BALTfinW1W3meantert if finalsample==1 & sex==0
reg DASH_potassiumw1w3mean food_price_BALTfinW1W3meantert if finalsample==1 & race==0
reg DASH_potassiumw1w3mean food_price_BALTfinW1W3meantert if finalsample==1 & race==1
reg DASH_potassiumw1w3mean food_price_BALTfinW1W3meantert if finalsample==1 & pir==0
reg DASH_potassiumw1w3mean food_price_BALTfinW1W3meantert if finalsample==1 & pir==1
```

```
reg DASH_Sodiumw1w3mean food_price_BALTfinW1W3meantert if finalsample==1
reg DASH_Sodiumw1w3mean food_price_BALTfinW1W3meantert if finalsample==1 & sex==1
reg DASH_Sodiumw1w3mean food_price_BALTfinW1W3meantert if finalsample==1 & sex==0
reg DASH_Sodiumw1w3mean food_price_BALTfinW1W3meantert if finalsample==1 & race==0
reg DASH_Sodiumw1w3mean food_price_BALTfinW1W3meantert if finalsample==1 & race==1
reg DASH_Sodiumw1w3mean food_price_BALTfinW1W3meantert if finalsample==1 & pir==0
reg DASH_Sodiumw1w3mean food_price_BALTfinW1W3meantert if finalsample==1 & pir==1

/////////////////P-trend heterogeneity by sex, race and pir/////////////////

reg DASH_scorew1w3mean food_price_BALTfinW1W3meantert##sex if finalsample==1
reg DASH_scorew1w3mean food_price_BALTfinW1W3meantert##race if finalsample==1
reg DASH_scorew1w3mean food_price_BALTfinW1W3meantert##pir if finalsample==1

reg DASH_SatFatw1w3mean food_price_BALTfinW1W3meantert##sex if finalsample==1
reg DASH_SatFatw1w3mean food_price_BALTfinW1W3meantert##race if finalsample==1
reg DASH_SatFatw1w3mean food_price_BALTfinW1W3meantert##pir if finalsample==1

reg DASH_Fatw1w3mean food_price_BALTfinW1W3meantert##sex if finalsample==1
reg DASH_Fatw1w3mean food_price_BALTfinW1W3meantert##race if finalsample==1
reg DASH_Fatw1w3mean food_price_BALTfinW1W3meantert##pir if finalsample==1

reg Dash_protein_W1W3mean food_price_BALTfinW1W3meantert##sex if finalsample==1
reg Dash_protein_W1W3mean food_price_BALTfinW1W3meantert##race if finalsample==1
reg Dash_protein_W1W3mean food_price_BALTfinW1W3meantert##pir if finalsample==1

reg DASH_cholesterolw1w3 food_price_BALTfinW1W3meantert##sex if finalsample==1
reg DASH_cholesterolw1w3 food_price_BALTfinW1W3meantert##race if finalsample==1
reg DASH_cholesterolw1w3 food_price_BALTfinW1W3meantert##pir if finalsample==1
```

```
reg DASH_fiberw1w3 food_price_BALTfinW1W3meantert##sex if finalsample==1
reg DASH_fiberw1w3 food_price_BALTfinW1W3meantert##race if finalsample==1
reg DASH_fiberw1w3 food_price_BALTfinW1W3meantert##pir if finalsample==1


reg DASH_Magnesiumw1w3mean food_price_BALTfinW1W3meantert##sex if finalsample==1
reg DASH_Magnesiumw1w3mean food_price_BALTfinW1W3meantert##race if finalsample==1
reg DASH_Magnesiumw1w3mean food_price_BALTfinW1W3meantert##pir if finalsample==1


reg DASH_calciumw1w3mean food_price_BALTfinW1W3meantert##sex if finalsample==1
reg DASH_calciumw1w3mean food_price_BALTfinW1W3meantert##race if finalsample==1
reg DASH_calciumw1w3mean food_price_BALTfinW1W3meantert##pir if finalsample==1


reg DASH_potassiumw1w3mean food_price_BALTfinW1W3meantert##sex if finalsample==1
reg DASH_potassiumw1w3mean food_price_BALTfinW1W3meantert##race if finalsample==1
reg DASH_potassiumw1w3mean food_price_BALTfinW1W3meantert##pir if finalsample==1


reg DASH_Sodiumw1w3mean food_price_BALTfinW1W3meantert##sex if finalsample==1
reg DASH_Sodiumw1w3mean food_price_BALTfinW1W3meantert##race if finalsample==1
reg DASH_Sodiumw1w3mean food_price_BALTfinW1W3meantert##pir if finalsample==1

/////////////////////////////MODEL ADJUSTED FOR ENERGY INTAKE (MEAN)//////////////////////////////////

**Compute means of DASH components**
//DASH_SatFatw1 DASH_Fatw1 DASH_proteinw1 DASH_cholesterolw1 DASH_fiberw1 DASH_Magnesiumw1
DASH_calciumw1 DASH_potassiumw1 DASH_Sodiumw1//
//DASH_SatFatw3 DASH_Fatw3 Dash_protein_W3 DASH_cholesterolw3 DASH_fiberw3 DASH_Magnesiumw3
DASH_calciumw3 DASH_potassiumw3 DASH_Sodiumw3//


capture drop DASH_SatFatw1w3mean
gen DASH_SatFatw1w3mean=(DASH_SatFatw1+DASH_SatFatw3)/2


capture drop DASH_Fatw1w3mean
gen DASH_Fatw1w3mean=(DASH_Fatw1+DASH_Fatw3)/2

capture drop Dash_protein_W1W3mean
gen Dash_protein_W1W3mean=(DASH_proteinw1+Dash_protein_W3)/2
```

```
capture drop DASH_cholesterolw1w3
gen DASH_cholesterolw1w3=(DASH_cholesterolw1+DASH_cholesterolw3)/2

capture drop DASH_fiberw1w3
gen DASH_fiberw1w3=(DASH_fiberw1+DASH_fiberw3)/2



capture drop DASH_Magnesiumw1w3mean
gen DASH_Magnesiumw1w3mean=(DASH_Magnesiumw1+DASH_Magnesiumw3)/2

capture drop DASH_calciumw1w3mean
gen DASH_calciumw1w3mean=(DASH_calciumw1+DASH_calciumw3)/2

capture drop DASH_potassiumw1w3mean
gen DASH_potassiumw1w3mean=(DASH_potassiumw1+DASH_potassiumw3)/2

capture drop DASH_Sodiumw1w3mean
gen DASH_Sodiumw1w3mean=(DASH_Sodiumw1+DASH_Sodiumw3)/2



save, replace

*******P-value comparing 2nd to 1st and 3rd to 1st*****

reg DASH_scorew1w3mean i.food_price_BALTfinW1W3meantert kcalw1w3mean_C if finalsample==1
reg DASH_scorew1w3mean i.food_price_BALTfinW1W3meantert kcalw1w3mean_C if finalsample==1 & sex==1
reg DASH_scorew1w3mean i.food_price_BALTfinW1W3meantert kcalw1w3mean_C if finalsample==1 & sex==0
reg DASH_scorew1w3mean i.food_price_BALTfinW1W3meantert kcalw1w3mean_C if finalsample==1 & race==0
reg DASH_scorew1w3mean i.food_price_BALTfinW1W3meantert kcalw1w3mean_C if finalsample==1 & race==1
reg DASH_scorew1w3mean i.food_price_BALTfinW1W3meantert kcalw1w3mean_C if finalsample==1 & pir==0
reg DASH_scorew1w3mean i.food_price_BALTfinW1W3meantert kcalw1w3mean_C if finalsample==1 & pir==1




reg DASH_SatFatw1w3mean i.food_price_BALTfinW1W3meantert kcalw1w3mean_C if finalsample==1
reg DASH_SatFatw1w3mean i.food_price_BALTfinW1W3meantert kcalw1w3mean_C if finalsample==1 & sex==1
reg DASH_SatFatw1w3mean i.food_price_BALTfinW1W3meantert kcalw1w3mean_C if finalsample==1 & sex==0
reg DASH_SatFatw1w3mean i.food_price_BALTfinW1W3meantert kcalw1w3mean_C if finalsample==1 & race==0
reg DASH_SatFatw1w3mean i.food_price_BALTfinW1W3meantert kcalw1w3mean_C if finalsample==1 & race==1
reg DASH_SatFatw1w3mean i.food_price_BALTfinW1W3meantert kcalw1w3mean_C if finalsample==1 & pir==0
reg DASH_SatFatw1w3mean i.food_price_BALTfinW1W3meantert kcalw1w3mean_C if finalsample==1 & pir==1
```

```
reg DASH_Fatw1w3mean i.food_price_BALTfinW1W3meantert kcalw1w3mean_C if finalsample==1
reg DASH_Fatw1w3mean i.food_price_BALTfinW1W3meantert kcalw1w3mean_C if finalsample==1 & sex==1
reg DASH_Fatw1w3mean i.food_price_BALTfinW1W3meantert kcalw1w3mean_C if finalsample==1 & sex==0
reg DASH_Fatw1w3mean i.food_price_BALTfinW1W3meantert kcalw1w3mean_C if finalsample==1 & race==0
reg DASH_Fatw1w3mean i.food_price_BALTfinW1W3meantert kcalw1w3mean_C if finalsample==1 & race==1
reg DASH_Fatw1w3mean i.food_price_BALTfinW1W3meantert kcalw1w3mean_C if finalsample==1 & pir==0
reg DASH_Fatw1w3mean i.food_price_BALTfinW1W3meantert kcalw1w3mean_C if finalsample==1 & pir==1

reg Dash_protein_W1W3mean i.food_price_BALTfinW1W3meantert kcalw1w3mean_C if finalsample==1
reg Dash_protein_W1W3mean i.food_price_BALTfinW1W3meantert kcalw1w3mean_C if finalsample==1 & sex==1
reg Dash_protein_W1W3mean i.food_price_BALTfinW1W3meantert kcalw1w3mean_C if finalsample==1 & sex==0
reg Dash_protein_W1W3mean i.food_price_BALTfinW1W3meantert kcalw1w3mean_C if finalsample==1 & race==0
reg Dash_protein_W1W3mean i.food_price_BALTfinW1W3meantert kcalw1w3mean_C if finalsample==1 & race==1
reg Dash_protein_W1W3mean i.food_price_BALTfinW1W3meantert kcalw1w3mean_C if finalsample==1 & pir==0
reg Dash_protein_W1W3mean i.food_price_BALTfinW1W3meantert kcalw1w3mean_C if finalsample==1 & pir==1

reg DASH_cholesterolw1w3 i.food_price_BALTfinW1W3meantert kcalw1w3mean_C if finalsample==1
reg DASH_cholesterolw1w3 i.food_price_BALTfinW1W3meantert kcalw1w3mean_C if finalsample==1 & sex==1
reg DASH_cholesterolw1w3 i.food_price_BALTfinW1W3meantert kcalw1w3mean_C if finalsample==1 & sex==0
reg DASH_cholesterolw1w3 i.food_price_BALTfinW1W3meantert kcalw1w3mean_C if finalsample==1 & race==0
reg DASH_cholesterolw1w3 i.food_price_BALTfinW1W3meantert kcalw1w3mean_C if finalsample==1 & race==1
reg DASH_cholesterolw1w3 i.food_price_BALTfinW1W3meantert kcalw1w3mean_C if finalsample==1 & pir==0
reg DASH_cholesterolw1w3 i.food_price_BALTfinW1W3meantert kcalw1w3mean_C if finalsample==1 & pir==1

reg DASH_fiberw1w3 i.food_price_BALTfinW1W3meantert kcalw1w3mean_C if finalsample==1
reg DASH_fiberw1w3 i.food_price_BALTfinW1W3meantert kcalw1w3mean_C if finalsample==1 & sex==1
reg DASH_fiberw1w3 i.food_price_BALTfinW1W3meantert kcalw1w3mean_C if finalsample==1 & sex==0
reg DASH_fiberw1w3 i.food_price_BALTfinW1W3meantert kcalw1w3mean_C if finalsample==1 & race==0
reg DASH_fiberw1w3 i.food_price_BALTfinW1W3meantert kcalw1w3mean_C if finalsample==1 & race==1
reg DASH_fiberw1w3 i.food_price_BALTfinW1W3meantert kcalw1w3mean_C if finalsample==1 & pir==0
reg DASH_fiberw1w3 i.food_price_BALTfinW1W3meantert kcalw1w3mean_C if finalsample==1 & pir==1

reg DASH_Magnesiumw1w3mean i.food_price_BALTfinW1W3meantert kcalw1w3mean_C if finalsample==1
reg DASH_Magnesiumw1w3mean i.food_price_BALTfinW1W3meantert kcalw1w3mean_C if finalsample==1 & sex==1
reg DASH_Magnesiumw1w3mean i.food_price_BALTfinW1W3meantert kcalw1w3mean_C if finalsample==1 & sex==0
```

```
reg DASH_Magnesiumw1w3mean i.food_price_BALTfinW1W3meantert kcalw1w3mean_C if finalsample==1 & race==0
reg DASH_Magnesiumw1w3mean i.food_price_BALTfinW1W3meantert kcalw1w3mean_C if finalsample==1 & race==1
reg DASH_Magnesiumw1w3mean i.food_price_BALTfinW1W3meantert kcalw1w3mean_C if finalsample==1 & pir==0
reg DASH_Magnesiumw1w3mean i.food_price_BALTfinW1W3meantert kcalw1w3mean_C if finalsample==1 & pir==1

reg DASH_calciumw1w3mean i.food_price_BALTfinW1W3meantert kcalw1w3mean_C if finalsample==1
reg DASH_calciumw1w3mean i.food_price_BALTfinW1W3meantert kcalw1w3mean_C if finalsample==1 & sex==1
reg DASH_calciumw1w3mean i.food_price_BALTfinW1W3meantert kcalw1w3mean_C if finalsample==1 & sex==0
reg DASH_calciumw1w3mean i.food_price_BALTfinW1W3meantert kcalw1w3mean_C if finalsample==1 & race==0
reg DASH_calciumw1w3mean i.food_price_BALTfinW1W3meantert kcalw1w3mean_C if finalsample==1 & race==1
reg DASH_calciumw1w3mean i.food_price_BALTfinW1W3meantert kcalw1w3mean_C if finalsample==1 & pir==0
reg DASH_calciumw1w3mean i.food_price_BALTfinW1W3meantert kcalw1w3mean_C if finalsample==1 & pir==1

reg DASH_potassiumw1w3mean i.food_price_BALTfinW1W3meantert kcalw1w3mean_C if finalsample==1
reg DASH_potassiumw1w3mean i.food_price_BALTfinW1W3meantert kcalw1w3mean_C if finalsample==1 & sex==1
reg DASH_potassiumw1w3mean i.food_price_BALTfinW1W3meantert kcalw1w3mean_C if finalsample==1 & sex==0
reg DASH_potassiumw1w3mean i.food_price_BALTfinW1W3meantert kcalw1w3mean_C if finalsample==1 & race==0
reg DASH_potassiumw1w3mean i.food_price_BALTfinW1W3meantert kcalw1w3mean_C if finalsample==1 & race==1
reg DASH_potassiumw1w3mean i.food_price_BALTfinW1W3meantert kcalw1w3mean_C if finalsample==1 & pir==0
reg DASH_potassiumw1w3mean i.food_price_BALTfinW1W3meantert kcalw1w3mean_C if finalsample==1 & pir==1

reg DASH_Sodiumw1w3mean i.food_price_BALTfinW1W3meantert kcalw1w3mean_C if finalsample==1
reg DASH_Sodiumw1w3mean i.food_price_BALTfinW1W3meantert kcalw1w3mean_C if finalsample==1 & sex==1
reg DASH_Sodiumw1w3mean i.food_price_BALTfinW1W3meantert kcalw1w3mean_C if finalsample==1 & sex==0
reg DASH_Sodiumw1w3mean i.food_price_BALTfinW1W3meantert kcalw1w3mean_C if finalsample==1 & race==0
reg DASH_Sodiumw1w3mean i.food_price_BALTfinW1W3meantert kcalw1w3mean_C if finalsample==1 & race==1
reg DASH_Sodiumw1w3mean i.food_price_BALTfinW1W3meantert kcalw1w3mean_C if finalsample==1 & pir==0
reg DASH_Sodiumw1w3mean i.food_price_BALTfinW1W3meantert kcalw1w3mean_C if finalsample==1 & pir==1

*******P-trend*****

reg DASH_scorew1w3mean food_price_BALTfinW1W3meantert kcalw1w3mean_C if finalsample==1
reg DASH_scorew1w3mean food_price_BALTfinW1W3meantert kcalw1w3mean_C if finalsample==1 & sex==1
reg DASH_scorew1w3mean food_price_BALTfinW1W3meantert kcalw1w3mean_C if finalsample==1 & sex==0
reg DASH_scorew1w3mean food_price_BALTfinW1W3meantert kcalw1w3mean_C if finalsample==1 & race==0
reg DASH_scorew1w3mean food_price_BALTfinW1W3meantert kcalw1w3mean_C if finalsample==1 & race==1
reg DASH_scorew1w3mean food_price_BALTfinW1W3meantert kcalw1w3mean_C if finalsample==1 & pir==0
reg DASH_scorew1w3mean food_price_BALTfinW1W3meantert kcalw1w3mean_C if finalsample==1 & pir==1
```

```
reg DASH_SatFatw1w3mean food_price_BALTfinW1W3meantert kcalw1w3mean_C if finalsample==1
reg DASH_SatFatw1w3mean food_price_BALTfinW1W3meantert kcalw1w3mean_C if finalsample==1 & sex==1
reg DASH_SatFatw1w3mean food_price_BALTfinW1W3meantert kcalw1w3mean_C if finalsample==1 & sex==0
reg DASH_SatFatw1w3mean food_price_BALTfinW1W3meantert kcalw1w3mean_C if finalsample==1 & race==0
reg DASH_SatFatw1w3mean food_price_BALTfinW1W3meantert kcalw1w3mean_C if finalsample==1 & race==1
reg DASH_SatFatw1w3mean food_price_BALTfinW1W3meantert kcalw1w3mean_C if finalsample==1 & pir==0
reg DASH_SatFatw1w3mean food_price_BALTfinW1W3meantert kcalw1w3mean_C if finalsample==1 & pir==1

reg DASH_Fatw1w3mean food_price_BALTfinW1W3meantert kcalw1w3mean_C if finalsample==1
reg DASH_Fatw1w3mean food_price_BALTfinW1W3meantert kcalw1w3mean_C if finalsample==1 & sex==1
reg DASH_Fatw1w3mean food_price_BALTfinW1W3meantert kcalw1w3mean_C if finalsample==1 & sex==0
reg DASH_Fatw1w3mean food_price_BALTfinW1W3meantert kcalw1w3mean_C if finalsample==1 & race==0
reg DASH_Fatw1w3mean food_price_BALTfinW1W3meantert kcalw1w3mean_C if finalsample==1 & race==1
reg DASH_Fatw1w3mean food_price_BALTfinW1W3meantert kcalw1w3mean_C if finalsample==1 & pir==0
reg DASH_Fatw1w3mean food_price_BALTfinW1W3meantert kcalw1w3mean_C if finalsample==1 & pir==1

reg Dash_protein_W1W3mean food_price_BALTfinW1W3meantert kcalw1w3mean_C if finalsample==1
reg Dash_protein_W1W3mean food_price_BALTfinW1W3meantert kcalw1w3mean_C if finalsample==1 & sex==1
reg Dash_protein_W1W3mean food_price_BALTfinW1W3meantert kcalw1w3mean_C if finalsample==1 & sex==0
reg Dash_protein_W1W3mean food_price_BALTfinW1W3meantert kcalw1w3mean_C if finalsample==1 & race==0
reg Dash_protein_W1W3mean food_price_BALTfinW1W3meantert kcalw1w3mean_C if finalsample==1 & race==1
reg Dash_protein_W1W3mean food_price_BALTfinW1W3meantert kcalw1w3mean_C if finalsample==1 & pir==0
reg Dash_protein_W1W3mean food_price_BALTfinW1W3meantert kcalw1w3mean_C if finalsample==1 & pir==1

reg DASH_cholesterolw1w3 food_price_BALTfinW1W3meantert kcalw1w3mean_C if finalsample==1
reg DASH_cholesterolw1w3 food_price_BALTfinW1W3meantert kcalw1w3mean_C if finalsample==1 & sex==1
reg DASH_cholesterolw1w3 food_price_BALTfinW1W3meantert kcalw1w3mean_C if finalsample==1 & sex==0
reg DASH_cholesterolw1w3 food_price_BALTfinW1W3meantert kcalw1w3mean_C if finalsample==1 & race==0
reg DASH_cholesterolw1w3 food_price_BALTfinW1W3meantert kcalw1w3mean_C if finalsample==1 & race==1
reg DASH_cholesterolw1w3 food_price_BALTfinW1W3meantert kcalw1w3mean_C if finalsample==1 & pir==0
reg DASH_cholesterolw1w3 food_price_BALTfinW1W3meantert kcalw1w3mean_C if finalsample==1 & pir==1
```

```
reg DASH_fiberw1w3 food_price_BALTfinW1W3meantert kcalw1w3mean_C if finalsample==1
reg DASH_fiberw1w3 food_price_BALTfinW1W3meantert kcalw1w3mean_C if finalsample==1 & sex==1
reg DASH_fiberw1w3 food_price_BALTfinW1W3meantert kcalw1w3mean_C if finalsample==1 & sex==0
reg DASH_fiberw1w3 food_price_BALTfinW1W3meantert kcalw1w3mean_C if finalsample==1 & race==0
reg DASH_fiberw1w3 food_price_BALTfinW1W3meantert kcalw1w3mean_C if finalsample==1 & race==1
reg DASH_fiberw1w3 food_price_BALTfinW1W3meantert kcalw1w3mean_C if finalsample==1 & pir==0
reg DASH_fiberw1w3 food_price_BALTfinW1W3meantert kcalw1w3mean_C if finalsample==1 & pir==1


reg DASH_Magnesiumw1w3mean food_price_BALTfinW1W3meantert kcalw1w3mean_C if finalsample==1
reg DASH_Magnesiumw1w3mean food_price_BALTfinW1W3meantert kcalw1w3mean_C if finalsample==1 & sex==1
reg DASH_Magnesiumw1w3mean food_price_BALTfinW1W3meantert kcalw1w3mean_C if finalsample==1 & sex==0
reg DASH_Magnesiumw1w3mean food_price_BALTfinW1W3meantert kcalw1w3mean_C if finalsample==1 & race==0
reg DASH_Magnesiumw1w3mean food_price_BALTfinW1W3meantert kcalw1w3mean_C if finalsample==1 & race==1
reg DASH_Magnesiumw1w3mean food_price_BALTfinW1W3meantert kcalw1w3mean_C if finalsample==1 & pir==0
reg DASH_Magnesiumw1w3mean food_price_BALTfinW1W3meantert kcalw1w3mean_C if finalsample==1 & pir==1


reg DASH_calciumw1w3mean food_price_BALTfinW1W3meantert kcalw1w3mean_C if finalsample==1
reg DASH_calciumw1w3mean food_price_BALTfinW1W3meantert kcalw1w3mean_C if finalsample==1 & sex==1
reg DASH_calciumw1w3mean food_price_BALTfinW1W3meantert kcalw1w3mean_C if finalsample==1 & sex==0
reg DASH_calciumw1w3mean food_price_BALTfinW1W3meantert kcalw1w3mean_C if finalsample==1 & race==0
reg DASH_calciumw1w3mean food_price_BALTfinW1W3meantert kcalw1w3mean_C if finalsample==1 & race==1
reg DASH_calciumw1w3mean food_price_BALTfinW1W3meantert kcalw1w3mean_C if finalsample==1 & pir==0
reg DASH_calciumw1w3mean food_price_BALTfinW1W3meantert kcalw1w3mean_C if finalsample==1 & pir==1


reg DASH_potassiumw1w3mean food_price_BALTfinW1W3meantert kcalw1w3mean_C if finalsample==1
reg DASH_potassiumw1w3mean food_price_BALTfinW1W3meantert kcalw1w3mean_C if finalsample==1 & sex==1
reg DASH_potassiumw1w3mean food_price_BALTfinW1W3meantert kcalw1w3mean_C if finalsample==1 & sex==0
reg DASH_potassiumw1w3mean food_price_BALTfinW1W3meantert kcalw1w3mean_C if finalsample==1 & race==0
reg DASH_potassiumw1w3mean food_price_BALTfinW1W3meantert kcalw1w3mean_C if finalsample==1 & race==1
reg DASH_potassiumw1w3mean food_price_BALTfinW1W3meantert kcalw1w3mean_C if finalsample==1 & pir==0
reg DASH_potassiumw1w3mean food_price_BALTfinW1W3meantert kcalw1w3mean_C if finalsample==1 & pir==1


reg DASH_Sodiumw1w3mean food_price_BALTfinW1W3meantert kcalw1w3mean_C if finalsample==1
reg DASH_Sodiumw1w3mean food_price_BALTfinW1W3meantert kcalw1w3mean_C if finalsample==1 & sex==1
reg DASH_Sodiumw1w3mean food_price_BALTfinW1W3meantert kcalw1w3mean_C if finalsample==1 & sex==0
reg DASH_Sodiumw1w3mean food_price_BALTfinW1W3meantert kcalw1w3mean_C if finalsample==1 & race==0
reg DASH_Sodiumw1w3mean food_price_BALTfinW1W3meantert kcalw1w3mean_C if finalsample==1 & race==1
```

```
reg DASH_Sodiumw1w3mean food_price_BALTfinW1W3meantert kcalw1w3mean_C if finalsample==1 & pir==0
reg DASH_Sodiumw1w3mean food_price_BALTfinW1W3meantert kcalw1w3mean_C if finalsample==1 & pir==1

//////////////////P-trend heterogeneity by sex, race and pir//////////////////

reg DASH_scorew1w3mean food_price_BALTfinW1W3meantert##sex kcalw1w3mean_C if finalsample==1
reg DASH_scorew1w3mean food_price_BALTfinW1W3meantert##race kcalw1w3mean_C if finalsample==1
reg DASH_scorew1w3mean food_price_BALTfinW1W3meantert##pir kcalw1w3mean_C if finalsample==1

reg DASH_SatFatw1w3mean food_price_BALTfinW1W3meantert##sex kcalw1w3mean_C if finalsample==1
reg DASH_SatFatw1w3mean food_price_BALTfinW1W3meantert##race kcalw1w3mean_C if finalsample==1
reg DASH_SatFatw1w3mean food_price_BALTfinW1W3meantert##pir kcalw1w3mean_C if finalsample==1

reg DASH_Fatw1w3mean food_price_BALTfinW1W3meantert##sex kcalw1w3mean_C if finalsample==1
reg DASH_Fatw1w3mean food_price_BALTfinW1W3meantert##race kcalw1w3mean_C if finalsample==1
reg DASH_Fatw1w3mean food_price_BALTfinW1W3meantert##pir kcalw1w3mean_C if finalsample==1

reg Dash_protein_W1W3mean food_price_BALTfinW1W3meantert##sex kcalw1w3mean_C if finalsample==1
reg Dash_protein_W1W3mean food_price_BALTfinW1W3meantert##race kcalw1w3mean_C if finalsample==1
reg Dash_protein_W1W3mean food_price_BALTfinW1W3meantert##pir kcalw1w3mean_C if finalsample==1

reg DASH_cholesterolw1w3 food_price_BALTfinW1W3meantert##sex kcalw1w3mean_C if finalsample==1
reg DASH_cholesterolw1w3 food_price_BALTfinW1W3meantert##race kcalw1w3mean_C if finalsample==1
reg DASH_cholesterolw1w3 food_price_BALTfinW1W3meantert##pir kcalw1w3mean_C if finalsample==1

reg DASH_fiberw1w3 food_price_BALTfinW1W3meantert##sex kcalw1w3mean_C if finalsample==1
reg DASH_fiberw1w3 food_price_BALTfinW1W3meantert##race kcalw1w3mean_C if finalsample==1
reg DASH_fiberw1w3 food_price_BALTfinW1W3meantert##pir kcalw1w3mean_C if finalsample==1

reg DASH_Magnesiumw1w3mean food_price_BALTfinW1W3meantert##sex kcalw1w3mean_C if finalsample==1
reg DASH_Magnesiumw1w3mean food_price_BALTfinW1W3meantert##race kcalw1w3mean_C if finalsample==1
```

```
reg DASH_Magnesiumw1w3mean food_price_BALTfinW1W3meantert##pir kcalw1w3mean_C if finalsample==1



reg DASH_calciumw1w3mean food_price_BALTfinW1W3meantert##sex kcalw1w3mean_C if finalsample==1
reg DASH_calciumw1w3mean food_price_BALTfinW1W3meantert##race kcalw1w3mean_C if finalsample==1
reg DASH_calciumw1w3mean food_price_BALTfinW1W3meantert##pir kcalw1w3mean_C if finalsample==1


reg DASH_potassiumw1w3mean food_price_BALTfinW1W3meantert##sex kcalw1w3mean_C if finalsample==1
reg DASH_potassiumw1w3mean food_price_BALTfinW1W3meantert##race kcalw1w3mean_C if finalsample==1
reg DASH_potassiumw1w3mean food_price_BALTfinW1W3meantert##pir kcalw1w3mean_C if finalsample==1



reg DASH_Sodiumw1w3mean food_price_BALTfinW1W3meantert##sex kcalw1w3mean_C if finalsample==1
reg DASH_Sodiumw1w3mean food_price_BALTfinW1W3meantert##race kcalw1w3mean_C if finalsample==1
reg DASH_Sodiumw1w3mean food_price_BALTfinW1W3meantert##pir kcalw1w3mean_C if finalsample==1

save, replace

////////////////////////////////////////TABLE 3///////////////////////

cd "G:\...\DATA"

use HANDLS_Allostaticload_dietfcfinal, clear

** Main predictors: food_price_BALTfinW1W3mean_C DASH_scorew1w3mean_C
**Covariates: Agew1_C Agew3_C sex race pir edubr employed wrattbr smoke currdrugs bmi_C SRHbr kcalw1w3mean_C energystoresw1w3mean_C

///////////////////////////////////BOTH FOOD COST AND DASH DIET TOGETHER IN MODEL: MODEL 1/////////////////////////

//TOTAL SAMPLE//
xtmixed allostatic_prop c.timew3w4##c.food_price_BALTfinW1W3mean_C c.timew3w4##c.DASH_scorew1w3mean_C c.timew3w4##c.Agew1_C c.timew3w4##c.Agew3_C c.timew3w4##sex c.timew3w4##race c.timew3w4##pir c.timew3w4##edubr c.timew3w4##employed c.timew3w4##wrattbr c.timew3w4##smoke c.timew3w4##currdrugs c.timew3w4##c.bmi_C c.timew3w4##SRHbr c.timew3w4##c.kcalw1w3mean_C c.timew3w4##c.energystoresw1w3mean_C c.timew3w4##c.invmillsmixed if sample8==1 || HNDID: timew3w4

su DASH_scorew1w3mean_C if sample8==1
su DASH_scorew1w3mean if sample8==1

set matsize 400
```

```
margins, at(c.time=(0(1)5) DASH_scorew1w3mean_C=(-1(1)1))
marginsplot, noci legend(rows(2)) recast(line) scheme(s1mono)

//MEN//
xtmixed allostatic_prop c.timew3w4##c.food_price_BALTfinW1W3mean_C c.timew3w4##c.DASH_scorew1w3mean_C c.timew3w4##c.Agew1_C c.timew3w4##c.Agew3_C c.timew3w4##sex c.timew3w4##race c.timew3w4##pir c.timew3w4##edubr c.timew3w4##employed c.timew3w4##wrattbr c.timew3w4##smoke c.timew3w4##currdrugs c.timew3w4##c.bmi_C c.timew3w4##SRHbr c.timew3w4##c.kcalw1w3mean_C c.timew3w4##c.energystoresw1w3mean_C c.timew3w4##c.invmillsmixed if sample8==1 & sex==1 || HNDID: timew3w4

//WOMEN//
xtmixed allostatic_prop c.timew3w4##c.food_price_BALTfinW1W3mean_C c.timew3w4##c.DASH_scorew1w3mean_C c.timew3w4##c.Agew1_C c.timew3w4##c.Agew3_C c.timew3w4##sex c.timew3w4##race c.timew3w4##pir c.timew3w4##edubr c.timew3w4##employed c.timew3w4##wrattbr c.timew3w4##smoke c.timew3w4##currdrugs c.timew3w4##c.bmi_C c.timew3w4##SRHbr c.timew3w4##c.kcalw1w3mean_C c.timew3w4##c.energystoresw1w3mean_C c.timew3w4##c.invmillsmixed if sample8==1 & sex==0 || HNDID: timew3w4

//WHITES//
xtmixed allostatic_prop c.timew3w4##c.food_price_BALTfinW1W3mean_C c.timew3w4##c.DASH_scorew1w3mean_C c.timew3w4##c.Agew1_C c.timew3w4##c.Agew3_C c.timew3w4##sex c.timew3w4##race c.timew3w4##pir c.timew3w4##edubr c.timew3w4##employed c.timew3w4##wrattbr c.timew3w4##smoke c.timew3w4##currdrugs c.timew3w4##c.bmi_C c.timew3w4##SRHbr c.timew3w4##c.kcalw1w3mean_C c.timew3w4##c.energystoresw1w3mean_C c.timew3w4##c.invmillsmixed if sample8==1 & race==0 || HNDID: timew3w4

//AA//
xtmixed allostatic_prop c.timew3w4##c.food_price_BALTfinW1W3mean_C c.timew3w4##c.DASH_scorew1w3mean_C c.timew3w4##c.Agew1_C c.timew3w4##c.Agew3_C c.timew3w4##sex c.timew3w4##race c.timew3w4##pir c.timew3w4##edubr c.timew3w4##employed c.timew3w4##wrattbr c.timew3w4##smoke c.timew3w4##currdrugs c.timew3w4##c.bmi_C c.timew3w4##SRHbr c.timew3w4##c.kcalw1w3mean_C c.timew3w4##c.energystoresw1w3mean_C c.timew3w4##c.invmillsmixed if sample8==1 & race==1 || HNDID: timew3w4

//BELOW POVERTY//
xtmixed allostatic_prop c.timew3w4##c.food_price_BALTfinW1W3mean_C c.timew3w4##c.DASH_scorew1w3mean_C c.timew3w4##c.Agew1_C c.timew3w4##c.Agew3_C c.timew3w4##sex c.timew3w4##race c.timew3w4##pir c.timew3w4##edubr c.timew3w4##employed c.timew3w4##wrattbr c.timew3w4##smoke c.timew3w4##currdrugs c.timew3w4##c.bmi_C c.timew3w4##SRHbr c.timew3w4##c.kcalw1w3mean_C c.timew3w4##c.energystoresw1w3mean_C c.timew3w4##c.invmillsmixed if sample8==1 & pir==0 || HNDID: timew3w4

//ABOVE POVERTY//
xtmixed allostatic_prop c.timew3w4##c.food_price_BALTfinW1W3mean_C c.timew3w4##c.DASH_scorew1w3mean_C c.timew3w4##c.Agew1_C c.timew3w4##c.Agew3_C c.timew3w4##sex c.timew3w4##race c.timew3w4##pir c.timew3w4##edubr c.timew3w4##employed c.timew3w4##wrattbr c.timew3w4##smoke c.timew3w4##currdrugs c.timew3w4##c.bmi_C c.timew3w4##SRHbr c.timew3w4##c.kcalw1w3mean_C c.timew3w4##c.energystoresw1w3mean_C c.timew3w4##c.invmillsmixed if sample8==1 & pir==1 || HNDID: timew3w4
```

Components of Allostatic load

//albumin crp chol hdl hgba1c whr bpsys bpdia hr//

****Albumin**********

//TOTAL SAMPLE//

xtmixed albumin c.timew3w4##c.food_price_BALTfinW1W3mean_C c.timew3w4##c.DASH_scorew1w3mean_C c.timew3w4##c.Agew1_C c.timew3w4##c.Agew3_C c.timew3w4##sex c.timew3w4##race c.timew3w4##pir c.timew3w4##edubr c.timew3w4##employed c.timew3w4##wrattbr c.timew3w4##smoke c.timew3w4##currdrugs c.timew3w4##c.bmi_C c.timew3w4##SRHbr c.timew3w4##c.kcalw1w3mean_C c.timew3w4##c.energystoresw1w3mean_C c.timew3w4##c.invmillsmixed if sample8==1 || HNDID: timew3w4

//MEN//

xtmixed albumin c.timew3w4##c.food_price_BALTfinW1W3mean_C c.timew3w4##c.DASH_scorew1w3mean_C c.timew3w4##c.Agew1_C c.timew3w4##c.Agew3_C c.timew3w4##sex c.timew3w4##race c.timew3w4##pir c.timew3w4##edubr c.timew3w4##employed c.timew3w4##wrattbr c.timew3w4##smoke c.timew3w4##currdrugs c.timew3w4##c.bmi_C c.timew3w4##SRHbr c.timew3w4##c.kcalw1w3mean_C c.timew3w4##c.energystoresw1w3mean_C c.timew3w4##c.invmillsmixed if sample8==1 & sex==1 || HNDID: timew3w4

//WOMEN//

xtmixed albumin c.timew3w4##c.food_price_BALTfinW1W3mean_C c.timew3w4##c.DASH_scorew1w3mean_C c.timew3w4##c.Agew1_C c.timew3w4##c.Agew3_C c.timew3w4##sex c.timew3w4##race c.timew3w4##pir c.timew3w4##edubr c.timew3w4##employed c.timew3w4##wrattbr c.timew3w4##smoke c.timew3w4##currdrugs c.timew3w4##c.bmi_C c.timew3w4##SRHbr c.timew3w4##c.kcalw1w3mean_C c.timew3w4##c.energystoresw1w3mean_C c.timew3w4##c.invmillsmixed if sample8==1 & sex==0 || HNDID: timew3w4

//WHITES//

xtmixed albumin c.timew3w4##c.food_price_BALTfinW1W3mean_C c.timew3w4##c.DASH_scorew1w3mean_C c.timew3w4##c.Agew1_C c.timew3w4##c.Agew3_C c.timew3w4##sex c.timew3w4##race c.timew3w4##pir c.timew3w4##edubr c.timew3w4##employed c.timew3w4##wrattbr c.timew3w4##smoke c.timew3w4##currdrugs c.timew3w4##c.bmi_C c.timew3w4##SRHbr c.timew3w4##c.kcalw1w3mean_C c.timew3w4##c.energystoresw1w3mean_C c.timew3w4##c.invmillsmixed if sample8==1 & race==0 || HNDID: timew3w4

//AA//

xtmixed albumin c.timew3w4##c.food_price_BALTfinW1W3mean_C c.timew3w4##c.DASH_scorew1w3mean_C c.timew3w4##c.Agew1_C c.timew3w4##c.Agew3_C c.timew3w4##sex c.timew3w4##race c.timew3w4##pir c.timew3w4##edubr c.timew3w4##employed c.timew3w4##wrattbr c.timew3w4##smoke c.timew3w4##currdrugs c.timew3w4##c.bmi_C c.timew3w4##SRHbr c.timew3w4##c.kcalw1w3mean_C c.timew3w4##c.energystoresw1w3mean_C c.timew3w4##c.invmillsmixed if sample8==1 & race==1 || HNDID: timew3w4

//BELOW POVERTY//

xtmixed albumin c.timew3w4##c.food_price_BALTfinW1W3mean_C c.timew3w4##c.DASH_scorew1w3mean_C c.timew3w4##c.Agew1_C c.timew3w4##c.Agew3_C c.timew3w4##sex c.timew3w4##race c.timew3w4##pir c.timew3w4##edubr c.timew3w4##employed c.timew3w4##wrattbr c.timew3w4##smoke c.timew3w4##currdrugs c.timew3w4##c.bmi_C c.timew3w4##SRHbr c.timew3w4##c.kcalw1w3mean_C c.timew3w4##c.energystoresw1w3mean_C c.timew3w4##c.invmillsmixed if sample8==1 & pir==0 || HNDID: timew3w4

//ABOVE POVERTY//

xtmixed albumin c.timew3w4##c.food_price_BALTfinW1W3mean_C c.timew3w4##c.DASH_scorew1w3mean_C c.timew3w4##c.Agew1_C c.timew3w4##c.Agew3_C c.timew3w4##sex c.timew3w4##race c.timew3w4##pir c.timew3w4##edubr c.timew3w4##employed c.timew3w4##wrattbr c.timew3w4##smoke c.timew3w4##currdrugs c.timew3w4##c.bmi_C c.timew3w4##SRHbr c.timew3w4##c.kcalw1w3mean_C c.timew3w4##c.energystoresw1w3mean_C c.timew3w4##c.invmillsmixed if sample8==1 & pir==1 || HNDID: timew3w4

*****C-reactive protein*******

//TOTAL SAMPLE//

xtmixed crp c.timew3w4##c.food_price_BALTfinW1W3mean_C c.timew3w4##c.DASH_scorew1w3mean_C c.timew3w4##c.Agew1_C c.timew3w4##c.Agew3_C c.timew3w4##sex c.timew3w4##race c.timew3w4##pir c.timew3w4##edubr c.timew3w4##employed c.timew3w4##wrattbr c.timew3w4##smoke c.timew3w4##currdrugs c.timew3w4##c.bmi_C c.timew3w4##SRHbr c.timew3w4##c.kcalw1w3mean_C c.timew3w4##c.energystoresw1w3mean_C c.timew3w4##c.invmillsmixed if sample8==1 || HNDID: timew3w4

//MEN//

xtmixed crp c.timew3w4##c.food_price_BALTfinW1W3mean_C c.timew3w4##c.DASH_scorew1w3mean_C c.timew3w4##c.Agew1_C c.timew3w4##c.Agew3_C c.timew3w4##sex c.timew3w4##race c.timew3w4##pir c.timew3w4##edubr c.timew3w4##employed c.timew3w4##wrattbr c.timew3w4##smoke c.timew3w4##currdrugs c.timew3w4##c.bmi_C c.timew3w4##SRHbr c.timew3w4##c.kcalw1w3mean_C c.timew3w4##c.energystoresw1w3mean_C c.timew3w4##c.invmillsmixed if sample8==1 & sex==1 || HNDID: timew3w4

//WOMEN//

xtmixed crp c.timew3w4##c.food_price_BALTfinW1W3mean_C c.timew3w4##c.DASH_scorew1w3mean_C c.timew3w4##c.Agew1_C c.timew3w4##c.Agew3_C c.timew3w4##sex c.timew3w4##race c.timew3w4##pir c.timew3w4##edubr c.timew3w4##employed c.timew3w4##wrattbr c.timew3w4##smoke c.timew3w4##currdrugs c.timew3w4##c.bmi_C c.timew3w4##SRHbr c.timew3w4##c.kcalw1w3mean_C c.timew3w4##c.energystoresw1w3mean_C c.timew3w4##c.invmillsmixed if sample8==1 & sex==0 || HNDID: timew3w4

//WHITES//

xtmixed crp c.timew3w4##c.food_price_BALTfinW1W3mean_C c.timew3w4##c.DASH_scorew1w3mean_C c.timew3w4##c.Agew1_C c.timew3w4##c.Agew3_C c.timew3w4##sex c.timew3w4##race c.timew3w4##pir c.timew3w4##edubr c.timew3w4##employed c.timew3w4##wrattbr c.timew3w4##smoke c.timew3w4##currdrugs c.timew3w4##c.bmi_C c.timew3w4##SRHbr c.timew3w4##c.kcalw1w3mean_C c.timew3w4##c.energystoresw1w3mean_C c.timew3w4##c.invmillsmixed if sample8==1 & race==0 || HNDID: timew3w4

//AA//

xtmixed crp c.timew3w4##c.food_price_BALTfinW1W3mean_C c.timew3w4##c.DASH_scorew1w3mean_C c.timew3w4##c.Agew1_C c.timew3w4##c.Agew3_C c.timew3w4##sex c.timew3w4##race c.timew3w4##pir c.timew3w4##edubr c.timew3w4##employed c.timew3w4##wrattbr c.timew3w4##smoke c.timew3w4##currdrugs c.timew3w4##c.bmi_C c.timew3w4##SRHbr c.timew3w4##c.kcalw1w3mean_C c.timew3w4##c.energystoresw1w3mean_C c.timew3w4##c.invmillsmixed if sample8==1 & race==1 || HNDID: timew3w4

//BELOW POVERTY//

xtmixed crp c.timew3w4##c.food_price_BALTfinW1W3mean_C c.timew3w4##c.DASH_scorew1w3mean_C c.timew3w4##c.Agew1_C c.timew3w4##c.Agew3_C c.timew3w4##sex c.timew3w4##race c.timew3w4##pir c.timew3w4##edubr c.timew3w4##employed c.timew3w4##wrattbr c.timew3w4##smoke c.timew3w4##currdrugs c.timew3w4##c.bmi_C c.timew3w4##SRHbr c.timew3w4##c.kcalw1w3mean_C c.timew3w4##c.energystoresw1w3mean_C c.timew3w4##c.invmillsmixed if sample8==1 & pir==0 || HNDID: timew3w4

//ABOVE POVERTY//

xtmixed crp c.timew3w4##c.food_price_BALTfinW1W3mean_C c.timew3w4##c.DASH_scorew1w3mean_C c.timew3w4##c.Agew1_C c.timew3w4##c.Agew3_C c.timew3w4##sex c.timew3w4##race c.timew3w4##pir c.timew3w4##edubr c.timew3w4##employed c.timew3w4##wrattbr c.timew3w4##smoke c.timew3w4##currdrugs c.timew3w4##c.bmi_C c.timew3w4##SRHbr c.timew3w4##c.kcalw1w3mean_C c.timew3w4##c.energystoresw1w3mean_C c.timew3w4##c.invmillsmixed if sample8==1 & pir==1 || HNDID: timew3w4

***********Cholesterol**************

//TOTAL SAMPLE//

xtmixed chol c.timew3w4##c.food_price_BALTfinW1W3mean_C c.timew3w4##c.DASH_scorew1w3mean_C c.timew3w4##c.Agew1_C c.timew3w4##c.Agew3_C c.timew3w4##sex c.timew3w4##race c.timew3w4##pir c.timew3w4##edubr c.timew3w4##employed c.timew3w4##wrattbr c.timew3w4##smoke c.timew3w4##currdrugs c.timew3w4##c.bmi_C c.timew3w4##SRHbr c.timew3w4##c.kcalw1w3mean_C c.timew3w4##c.energystoresw1w3mean_C c.timew3w4##c.invmillsmixed if sample8==1 || HNDID: timew3w4

//MEN//

xtmixed chol c.timew3w4##c.food_price_BALTfinW1W3mean_C c.timew3w4##c.DASH_scorew1w3mean_C c.timew3w4##c.Agew1_C c.timew3w4##c.Agew3_C c.timew3w4##sex c.timew3w4##race c.timew3w4##pir c.timew3w4##edubr c.timew3w4##employed c.timew3w4##wrattbr c.timew3w4##smoke c.timew3w4##currdrugs c.timew3w4##c.bmi_C c.timew3w4##SRHbr c.timew3w4##c.kcalw1w3mean_C c.timew3w4##c.energystoresw1w3mean_C c.timew3w4##c.invmillsmixed if sample8==1 & sex==1 || HNDID: timew3w4

//WOMEN//

xtmixed chol c.timew3w4##c.food_price_BALTfinW1W3mean_C c.timew3w4##c.DASH_scorew1w3mean_C c.timew3w4##c.Agew1_C c.timew3w4##c.Agew3_C c.timew3w4##sex c.timew3w4##race c.timew3w4##pir c.timew3w4##edubr c.timew3w4##employed c.timew3w4##wrattbr c.timew3w4##smoke c.timew3w4##currdrugs

c.timew3w4##c.bmi_C c.timew3w4##SRHbr c.timew3w4##c.kcalw1w3mean_C c.timew3w4##c.energystoresw1w3mean_C c.timew3w4##c.invmillsmixed if sample8==1 & sex==0 || HNDID: timew3w4

//WHITES//

xtmixed chol c.timew3w4##c.food_price_BALTfinW1W3mean_C c.timew3w4##c.DASH_scorew1w3mean_C c.timew3w4##c.Agew1_C c.timew3w4##c.Agew3_C c.timew3w4##sex c.timew3w4##race c.timew3w4##pir c.timew3w4##edubr c.timew3w4##employed c.timew3w4##wrattbr c.timew3w4##smoke c.timew3w4##currdrugs c.timew3w4##c.bmi_C c.timew3w4##SRHbr c.timew3w4##c.kcalw1w3mean_C c.timew3w4##c.energystoresw1w3mean_C c.timew3w4##c.invmillsmixed if sample8==1 & race==0 || HNDID: timew3w4

//AA//

xtmixed chol c.timew3w4##c.food_price_BALTfinW1W3mean_C c.timew3w4##c.DASH_scorew1w3mean_C c.timew3w4##c.Agew1_C c.timew3w4##c.Agew3_C c.timew3w4##sex c.timew3w4##race c.timew3w4##pir c.timew3w4##edubr c.timew3w4##employed c.timew3w4##wrattbr c.timew3w4##smoke c.timew3w4##currdrugs c.timew3w4##c.bmi_C c.timew3w4##SRHbr c.timew3w4##c.kcalw1w3mean_C c.timew3w4##c.energystoresw1w3mean_C c.timew3w4##c.invmillsmixed if sample8==1 & race==1 || HNDID: timew3w4

//BELOW POVERTY//

xtmixed chol c.timew3w4##c.food_price_BALTfinW1W3mean_C c.timew3w4##c.DASH_scorew1w3mean_C c.timew3w4##c.Agew1_C c.timew3w4##c.Agew3_C c.timew3w4##sex c.timew3w4##race c.timew3w4##pir c.timew3w4##edubr c.timew3w4##employed c.timew3w4##wrattbr c.timew3w4##smoke c.timew3w4##currdrugs c.timew3w4##c.bmi_C c.timew3w4##SRHbr c.timew3w4##c.kcalw1w3mean_C c.timew3w4##c.energystoresw1w3mean_C c.timew3w4##c.invmillsmixed if sample8==1 & pir==0 || HNDID: timew3w4

//ABOVE POVERTY//

xtmixed chol c.timew3w4##c.food_price_BALTfinW1W3mean_C c.timew3w4##c.DASH_scorew1w3mean_C c.timew3w4##c.Agew1_C c.timew3w4##c.Agew3_C c.timew3w4##sex c.timew3w4##race c.timew3w4##pir c.timew3w4##edubr c.timew3w4##employed c.timew3w4##wrattbr c.timew3w4##smoke c.timew3w4##currdrugs c.timew3w4##c.bmi_C c.timew3w4##SRHbr c.timew3w4##c.kcalw1w3mean_C c.timew3w4##c.energystoresw1w3mean_C c.timew3w4##c.invmillsmixed if sample8==1 & pir==1 || HNDID: timew3w4

***************HDL-C******************

//TOTAL SAMPLE//

xtmixed hdl c.timew3w4##c.food_price_BALTfinW1W3mean_C c.timew3w4##c.DASH_scorew1w3mean_C c.timew3w4##c.Agew1_C c.timew3w4##c.Agew3_C c.timew3w4##sex c.timew3w4##race c.timew3w4##pir c.timew3w4##edubr c.timew3w4##employed c.timew3w4##wrattbr c.timew3w4##smoke c.timew3w4##currdrugs c.timew3w4##c.bmi_C c.timew3w4##SRHbr c.timew3w4##c.kcalw1w3mean_C c.timew3w4##c.energystoresw1w3mean_C c.timew3w4##c.invmillsmixed if sample8==1 || HNDID: timew3w4

//MEN//

xtmixed hdl c.timew3w4##c.food_price_BALTfinW1W3mean_C c.timew3w4##c.DASH_scorew1w3mean_C c.timew3w4##c.Agew1_C c.timew3w4##c.Agew3_C c.timew3w4##sex c.timew3w4##race c.timew3w4##pir

c.timew3w4##edubr c.timew3w4##employed c.timew3w4##wrattbr c.timew3w4##smoke c.timew3w4##currdrugs c.timew3w4##c.bmi_C c.timew3w4##SRHbr c.timew3w4##c.kcalw1w3mean_C c.timew3w4##c.energystoresw1w3mean_C c.timew3w4##c.invmillsmixed if sample8==1 & sex==1 || HNDID: timew3w4

//WOMEN//

xtmixed hdl c.timew3w4##c.food_price_BALTfinW1W3mean_C c.timew3w4##c.DASH_scorew1w3mean_C c.timew3w4##c.Agew1_C c.timew3w4##c.Agew3_C c.timew3w4##sex c.timew3w4##race c.timew3w4##pir c.timew3w4##edubr c.timew3w4##employed c.timew3w4##wrattbr c.timew3w4##smoke c.timew3w4##currdrugs c.timew3w4##c.bmi_C c.timew3w4##SRHbr c.timew3w4##c.kcalw1w3mean_C c.timew3w4##c.energystoresw1w3mean_C c.timew3w4##c.invmillsmixed if sample8==1 & sex==0 || HNDID: timew3w4

//WHITES//

xtmixed hdl c.timew3w4##c.food_price_BALTfinW1W3mean_C c.timew3w4##c.DASH_scorew1w3mean_C c.timew3w4##c.Agew1_C c.timew3w4##c.Agew3_C c.timew3w4##sex c.timew3w4##race c.timew3w4##pir c.timew3w4##edubr c.timew3w4##employed c.timew3w4##wrattbr c.timew3w4##smoke c.timew3w4##currdrugs c.timew3w4##c.bmi_C c.timew3w4##SRHbr c.timew3w4##c.kcalw1w3mean_C c.timew3w4##c.energystoresw1w3mean_C c.timew3w4##c.invmillsmixed if sample8==1 & race==0 || HNDID: timew3w4

//AA//

xtmixed hdl c.timew3w4##c.food_price_BALTfinW1W3mean_C c.timew3w4##c.DASH_scorew1w3mean_C c.timew3w4##c.Agew1_C c.timew3w4##c.Agew3_C c.timew3w4##sex c.timew3w4##race c.timew3w4##pir c.timew3w4##edubr c.timew3w4##employed c.timew3w4##wrattbr c.timew3w4##smoke c.timew3w4##currdrugs c.timew3w4##c.bmi_C c.timew3w4##SRHbr c.timew3w4##c.kcalw1w3mean_C c.timew3w4##c.energystoresw1w3mean_C c.timew3w4##c.invmillsmixed if sample8==1 & race==1 || HNDID: timew3w4

//BELOW POVERTY//

xtmixed hdl c.timew3w4##c.food_price_BALTfinW1W3mean_C c.timew3w4##c.DASH_scorew1w3mean_C c.timew3w4##c.Agew1_C c.timew3w4##c.Agew3_C c.timew3w4##sex c.timew3w4##race c.timew3w4##pir c.timew3w4##edubr c.timew3w4##employed c.timew3w4##wrattbr c.timew3w4##smoke c.timew3w4##currdrugs c.timew3w4##c.bmi_C c.timew3w4##SRHbr c.timew3w4##c.kcalw1w3mean_C c.timew3w4##c.energystoresw1w3mean_C c.timew3w4##c.invmillsmixed if sample8==1 & pir==0 || HNDID: timew3w4

//ABOVE POVERTY//

xtmixed hdl c.timew3w4##c.food_price_BALTfinW1W3mean_C c.timew3w4##c.DASH_scorew1w3mean_C c.timew3w4##c.Agew1_C c.timew3w4##c.Agew3_C c.timew3w4##sex c.timew3w4##race c.timew3w4##pir c.timew3w4##edubr c.timew3w4##employed c.timew3w4##wrattbr c.timew3w4##smoke c.timew3w4##currdrugs c.timew3w4##c.bmi_C c.timew3w4##SRHbr c.timew3w4##c.kcalw1w3mean_C c.timew3w4##c.energystoresw1w3mean_C c.timew3w4##c.invmillsmixed if sample8==1 & pir==1 || HNDID: timew3w4

/////////////GLYCATED HEMOGLOBIN/////////////

//TOTAL SAMPLE//

```
xtmixed hgba1c c.timew3w4##c.food_price_BALTfinW1W3mean_C c.timew3w4##c.DASH_scorew1w3mean_C c.timew3w4##c.Agew1_C c.timew3w4##c.Agew3_C c.timew3w4##sex c.timew3w4##race c.timew3w4##pir c.timew3w4##edubr c.timew3w4##employed c.timew3w4##wrattbr c.timew3w4##smoke c.timew3w4##currdrugs c.timew3w4##c.bmi_C c.timew3w4##SRHbr c.timew3w4##c.kcalw1w3mean_C c.timew3w4##c.energystoresw1w3mean_C c.timew3w4##c.invmillsmixed if sample8==1 || HNDID: timew3w4
```

```
//MEN//
xtmixed hgba1c c.timew3w4##c.food_price_BALTfinW1W3mean_C c.timew3w4##c.DASH_scorew1w3mean_C c.timew3w4##c.Agew1_C c.timew3w4##c.Agew3_C c.timew3w4##sex c.timew3w4##race c.timew3w4##pir c.timew3w4##edubr c.timew3w4##employed c.timew3w4##wrattbr c.timew3w4##smoke c.timew3w4##currdrugs c.timew3w4##c.bmi_C c.timew3w4##SRHbr c.timew3w4##c.kcalw1w3mean_C c.timew3w4##c.energystoresw1w3mean_C c.timew3w4##c.invmillsmixed if sample8==1 & sex==1 || HNDID: timew3w4

//WOMEN//
xtmixed hgba1c c.timew3w4##c.food_price_BALTfinW1W3mean_C c.timew3w4##c.DASH_scorew1w3mean_C c.timew3w4##c.Agew1_C c.timew3w4##c.Agew3_C c.timew3w4##sex c.timew3w4##race c.timew3w4##pir c.timew3w4##edubr c.timew3w4##employed c.timew3w4##wrattbr c.timew3w4##smoke c.timew3w4##currdrugs c.timew3w4##c.bmi_C c.timew3w4##SRHbr c.timew3w4##c.kcalw1w3mean_C c.timew3w4##c.energystoresw1w3mean_C c.timew3w4##c.invmillsmixed if sample8==1 & sex==0 || HNDID: timew3w4

//WHITES//
xtmixed hgba1c c.timew3w4##c.food_price_BALTfinW1W3mean_C c.timew3w4##c.DASH_scorew1w3mean_C c.timew3w4##c.Agew1_C c.timew3w4##c.Agew3_C c.timew3w4##sex c.timew3w4##race c.timew3w4##pir c.timew3w4##edubr c.timew3w4##employed c.timew3w4##wrattbr c.timew3w4##smoke c.timew3w4##currdrugs c.timew3w4##c.bmi_C c.timew3w4##SRHbr c.timew3w4##c.kcalw1w3mean_C c.timew3w4##c.energystoresw1w3mean_C c.timew3w4##c.invmillsmixed if sample8==1 & race==0 || HNDID: timew3w4

//AA//
xtmixed hgba1c c.timew3w4##c.food_price_BALTfinW1W3mean_C c.timew3w4##c.DASH_scorew1w3mean_C c.timew3w4##c.Agew1_C c.timew3w4##c.Agew3_C c.timew3w4##sex c.timew3w4##race c.timew3w4##pir c.timew3w4##edubr c.timew3w4##employed c.timew3w4##wrattbr c.timew3w4##smoke c.timew3w4##currdrugs c.timew3w4##c.bmi_C c.timew3w4##SRHbr c.timew3w4##c.kcalw1w3mean_C c.timew3w4##c.energystoresw1w3mean_C c.timew3w4##c.invmillsmixed if sample8==1 & race==1 || HNDID: timew3w4

//BELOW POVERTY//
xtmixed hgba1c c.timew3w4##c.food_price_BALTfinW1W3mean_C c.timew3w4##c.DASH_scorew1w3mean_C c.timew3w4##c.Agew1_C c.timew3w4##c.Agew3_C c.timew3w4##sex c.timew3w4##race c.timew3w4##pir c.timew3w4##edubr c.timew3w4##employed c.timew3w4##wrattbr c.timew3w4##smoke c.timew3w4##currdrugs c.timew3w4##c.bmi_C c.timew3w4##SRHbr c.timew3w4##c.kcalw1w3mean_C c.timew3w4##c.energystoresw1w3mean_C c.timew3w4##c.invmillsmixed if sample8==1 & pir==0 || HNDID: timew3w4

//ABOVE POVERTY//
```

xtmixed hgba1c c.timew3w4##c.food_price_BALTfinW1W3mean_C c.timew3w4##c.DASH_scorew1w3mean_C c.timew3w4##c.Agew1_C c.timew3w4##c.Agew3_C c.timew3w4##sex c.timew3w4##race c.timew3w4##pir c.timew3w4##edubr c.timew3w4##employed c.timew3w4##wrattbr c.timew3w4##smoke c.timew3w4##currdrugs c.timew3w4##c.bmi_C c.timew3w4##SRHbr c.timew3w4##c.kcalw1w3mean_C c.timew3w4##c.energystoresw1w3mean_C c.timew3w4##c.invmillsmixed if sample8==1 & pir==1 || HNDID: timew3w4

////////////////WHR////////////////

//TOTAL SAMPLE//

xtmixed whr c.timew3w4##c.food_price_BALTfinW1W3mean_C c.timew3w4##c.DASH_scorew1w3mean_C c.timew3w4##c.Agew1_C c.timew3w4##c.Agew3_C c.timew3w4##sex c.timew3w4##race c.timew3w4##pir c.timew3w4##edubr c.timew3w4##employed c.timew3w4##wrattbr c.timew3w4##smoke c.timew3w4##currdrugs c.timew3w4##c.bmi_C c.timew3w4##SRHbr c.timew3w4##c.kcalw1w3mean_C c.timew3w4##c.energystoresw1w3mean_C c.timew3w4##c.invmillsmixed if sample8==1 || HNDID: timew3w4

//MEN//

xtmixed whr c.timew3w4##c.food_price_BALTfinW1W3mean_C c.timew3w4##c.DASH_scorew1w3mean_C c.timew3w4##c.Agew1_C c.timew3w4##c.Agew3_C c.timew3w4##sex c.timew3w4##race c.timew3w4##pir c.timew3w4##edubr c.timew3w4##employed c.timew3w4##wrattbr c.timew3w4##smoke c.timew3w4##currdrugs c.timew3w4##c.bmi_C c.timew3w4##SRHbr c.timew3w4##c.kcalw1w3mean_C c.timew3w4##c.energystoresw1w3mean_C c.timew3w4##c.invmillsmixed if sample8==1 & sex==1 || HNDID: timew3w4

//WOMEN//

xtmixed whr c.timew3w4##c.food_price_BALTfinW1W3mean_C c.timew3w4##c.DASH_scorew1w3mean_C c.timew3w4##c.Agew1_C c.timew3w4##c.Agew3_C c.timew3w4##sex c.timew3w4##race c.timew3w4##pir c.timew3w4##edubr c.timew3w4##employed c.timew3w4##wrattbr c.timew3w4##smoke c.timew3w4##currdrugs c.timew3w4##c.bmi_C c.timew3w4##SRHbr c.timew3w4##c.kcalw1w3mean_C c.timew3w4##c.energystoresw1w3mean_C c.timew3w4##c.invmillsmixed if sample8==1 & sex==0 || HNDID: timew3w4

//WHITES//

xtmixed whr c.timew3w4##c.food_price_BALTfinW1W3mean_C c.timew3w4##c.DASH_scorew1w3mean_C c.timew3w4##c.Agew1_C c.timew3w4##c.Agew3_C c.timew3w4##sex c.timew3w4##race c.timew3w4##pir c.timew3w4##edubr c.timew3w4##employed c.timew3w4##wrattbr c.timew3w4##smoke c.timew3w4##currdrugs c.timew3w4##c.bmi_C c.timew3w4##SRHbr c.timew3w4##c.kcalw1w3mean_C c.timew3w4##c.energystoresw1w3mean_C c.timew3w4##c.invmillsmixed if sample8==1 & race==0 || HNDID: timew3w4

//AA//

xtmixed whr c.timew3w4##c.food_price_BALTfinW1W3mean_C c.timew3w4##c.DASH_scorew1w3mean_C c.timew3w4##c.Agew1_C c.timew3w4##c.Agew3_C c.timew3w4##sex c.timew3w4##race c.timew3w4##pir c.timew3w4##edubr c.timew3w4##employed c.timew3w4##wrattbr c.timew3w4##smoke c.timew3w4##currdrugs c.timew3w4##c.bmi_C c.timew3w4##SRHbr c.timew3w4##c.kcalw1w3mean_C c.timew3w4##c.energystoresw1w3mean_C c.timew3w4##c.invmillsmixed if sample8==1 & race==1 || HNDID: timew3w4

```
//BELOW POVERTY//
xtmixed whr c.timew3w4##c.food_price_BALTfinW1W3mean_C c.timew3w4##c.DASH_scorew1w3mean_C c.timew3w4##c.Agew1_C c.timew3w4##c.Agew3_C c.timew3w4##sex c.timew3w4##race c.timew3w4##pir c.timew3w4##edubr c.timew3w4##employed c.timew3w4##wrattbr c.timew3w4##smoke c.timew3w4##currdrugs c.timew3w4##c.bmi_C c.timew3w4##SRHbr c.timew3w4##c.kcalw1w3mean_C c.timew3w4##c.energystoresw1w3mean_C c.timew3w4##c.invmillsmixed if sample8==1 & pir==0 || HNDID: timew3w4

//ABOVE POVERTY//
xtmixed whr c.timew3w4##c.food_price_BALTfinW1W3mean_C c.timew3w4##c.DASH_scorew1w3mean_C c.timew3w4##c.Agew1_C c.timew3w4##c.Agew3_C c.timew3w4##sex c.timew3w4##race c.timew3w4##pir c.timew3w4##edubr c.timew3w4##employed c.timew3w4##wrattbr c.timew3w4##smoke c.timew3w4##currdrugs c.timew3w4##c.bmi_C c.timew3w4##SRHbr c.timew3w4##c.kcalw1w3mean_C c.timew3w4##c.energystoresw1w3mean_C c.timew3w4##c.invmillsmixed if sample8==1 & pir==1 || HNDID: timew3w4

///////////////////////////SYSTOLIC BLOOD PRESSURE/////////////////////

//TOTAL SAMPLE//
xtmixed bpsys c.timew3w4##c.food_price_BALTfinW1W3mean_C c.timew3w4##c.DASH_scorew1w3mean_C c.timew3w4##c.Agew1_C c.timew3w4##c.Agew3_C c.timew3w4##sex c.timew3w4##race c.timew3w4##pir c.timew3w4##edubr c.timew3w4##employed c.timew3w4##wrattbr c.timew3w4##smoke c.timew3w4##currdrugs c.timew3w4##c.bmi_C c.timew3w4##SRHbr c.timew3w4##c.kcalw1w3mean_C c.timew3w4##c.energystoresw1w3mean_C c.timew3w4##c.invmillsmixed if sample8==1 || HNDID: timew3w4


//MEN//
xtmixed bpsys c.timew3w4##c.food_price_BALTfinW1W3mean_C c.timew3w4##c.DASH_scorew1w3mean_C c.timew3w4##c.Agew1_C c.timew3w4##c.Agew3_C c.timew3w4##sex c.timew3w4##race c.timew3w4##pir c.timew3w4##edubr c.timew3w4##employed c.timew3w4##wrattbr c.timew3w4##smoke c.timew3w4##currdrugs c.timew3w4##c.bmi_C c.timew3w4##SRHbr c.timew3w4##c.kcalw1w3mean_C c.timew3w4##c.energystoresw1w3mean_C c.timew3w4##c.invmillsmixed if sample8==1 & sex==1 || HNDID: timew3w4

//WOMEN//
xtmixed bpsys c.timew3w4##c.food_price_BALTfinW1W3mean_C c.timew3w4##c.DASH_scorew1w3mean_C c.timew3w4##c.Agew1_C c.timew3w4##c.Agew3_C c.timew3w4##sex c.timew3w4##race c.timew3w4##pir c.timew3w4##edubr c.timew3w4##employed c.timew3w4##wrattbr c.timew3w4##smoke c.timew3w4##currdrugs c.timew3w4##c.bmi_C c.timew3w4##SRHbr c.timew3w4##c.kcalw1w3mean_C c.timew3w4##c.energystoresw1w3mean_C c.timew3w4##c.invmillsmixed if sample8==1 & sex==0 || HNDID: timew3w4

//WHITES//
xtmixed bpsys c.timew3w4##c.food_price_BALTfinW1W3mean_C c.timew3w4##c.DASH_scorew1w3mean_C c.timew3w4##c.Agew1_C c.timew3w4##c.Agew3_C c.timew3w4##sex c.timew3w4##race c.timew3w4##pir c.timew3w4##edubr c.timew3w4##employed c.timew3w4##wrattbr c.timew3w4##smoke c.timew3w4##currdrugs
```

c.timew3w4##c.bmi_C c.timew3w4##SRHbr c.timew3w4##c.kcalw1w3mean_C c.timew3w4##c.energystoresw1w3mean_C c.timew3w4##c.invmillsmixed if sample8==1 & race==0 || HNDID: timew3w4

//AA//

xtmixed bpsys c.timew3w4##c.food_price_BALTfinW1W3mean_C c.timew3w4##c.DASH_scorew1w3mean_C c.timew3w4##c.Agew1_C c.timew3w4##c.Agew3_C c.timew3w4##sex c.timew3w4##race c.timew3w4##pir c.timew3w4##edubr c.timew3w4##employed c.timew3w4##wrattbr c.timew3w4##smoke c.timew3w4##currdrugs c.timew3w4##c.bmi_C c.timew3w4##SRHbr c.timew3w4##c.kcalw1w3mean_C c.timew3w4##c.energystoresw1w3mean_C c.timew3w4##c.invmillsmixed if sample8==1 & race==1 || HNDID: timew3w4

//BELOW POVERTY//

xtmixed bpsys c.timew3w4##c.food_price_BALTfinW1W3mean_C c.timew3w4##c.DASH_scorew1w3mean_C c.timew3w4##c.Agew1_C c.timew3w4##c.Agew3_C c.timew3w4##sex c.timew3w4##race c.timew3w4##pir c.timew3w4##edubr c.timew3w4##employed c.timew3w4##wrattbr c.timew3w4##smoke c.timew3w4##currdrugs c.timew3w4##c.bmi_C c.timew3w4##SRHbr c.timew3w4##c.kcalw1w3mean_C c.timew3w4##c.energystoresw1w3mean_C c.timew3w4##c.invmillsmixed if sample8==1 & pir==0 || HNDID: timew3w4

//ABOVE POVERTY//

xtmixed bpsys c.timew3w4##c.food_price_BALTfinW1W3mean_C c.timew3w4##c.DASH_scorew1w3mean_C c.timew3w4##c.Agew1_C c.timew3w4##c.Agew3_C c.timew3w4##sex c.timew3w4##race c.timew3w4##pir c.timew3w4##edubr c.timew3w4##employed c.timew3w4##wrattbr c.timew3w4##smoke c.timew3w4##currdrugs c.timew3w4##c.bmi_C c.timew3w4##SRHbr c.timew3w4##c.kcalw1w3mean_C c.timew3w4##c.energystoresw1w3mean_C c.timew3w4##c.invmillsmixed if sample8==1 & pir==1 || HNDID: timew3w4

/////////////////////////DIASTOLIC BLOOD PRESSURE/////////////

//TOTAL SAMPLE//

xtmixed bpdia c.timew3w4##c.food_price_BALTfinW1W3mean_C c.timew3w4##c.DASH_scorew1w3mean_C c.timew3w4##c.Agew1_C c.timew3w4##c.Agew3_C c.timew3w4##sex c.timew3w4##race c.timew3w4##pir c.timew3w4##edubr c.timew3w4##employed c.timew3w4##wrattbr c.timew3w4##smoke c.timew3w4##currdrugs c.timew3w4##c.bmi_C c.timew3w4##SRHbr c.timew3w4##c.kcalw1w3mean_C c.timew3w4##c.energystoresw1w3mean_C c.timew3w4##c.invmillsmixed if sample8==1 || HNDID: timew3w4

//MEN//

xtmixed bpdia c.timew3w4##c.food_price_BALTfinW1W3mean_C c.timew3w4##c.DASH_scorew1w3mean_C c.timew3w4##c.Agew1_C c.timew3w4##c.Agew3_C c.timew3w4##sex c.timew3w4##race c.timew3w4##pir c.timew3w4##edubr c.timew3w4##employed c.timew3w4##wrattbr c.timew3w4##smoke c.timew3w4##currdrugs c.timew3w4##c.bmi_C c.timew3w4##SRHbr c.timew3w4##c.kcalw1w3mean_C c.timew3w4##c.energystoresw1w3mean_C c.timew3w4##c.invmillsmixed if sample8==1 & sex==1 || HNDID: timew3w4

//WOMEN//

xtmixed bpdia c.timew3w4##c.food_price_BALTfinW1W3mean_C c.timew3w4##c.DASH_scorew1w3mean_C c.timew3w4##c.Agew1_C c.timew3w4##c.Agew3_C c.timew3w4##sex c.timew3w4##race c.timew3w4##pir

c.timew3w4##edubr c.timew3w4##employed c.timew3w4##wrattbr c.timew3w4##smoke c.timew3w4##currdrugs c.timew3w4##c.bmi_C c.timew3w4##SRHbr c.timew3w4##c.kcalw1w3mean_C c.timew3w4##c.energystoresw1w3mean_C c.timew3w4##c.invmillsmixed if sample8==1 & sex==0 || HNDID: timew3w4

//WHITES//

xtmixed bpdia c.timew3w4##c.food_price_BALTfinW1W3mean_C c.timew3w4##c.DASH_scorew1w3mean_C c.timew3w4##c.Agew1_C c.timew3w4##c.Agew3_C c.timew3w4##sex c.timew3w4##race c.timew3w4##pir c.timew3w4##edubr c.timew3w4##employed c.timew3w4##wrattbr c.timew3w4##smoke c.timew3w4##currdrugs c.timew3w4##c.bmi_C c.timew3w4##SRHbr c.timew3w4##c.kcalw1w3mean_C c.timew3w4##c.energystoresw1w3mean_C c.timew3w4##c.invmillsmixed if sample8==1 & race==0 || HNDID: timew3w4

//AA//

xtmixed bpdia c.timew3w4##c.food_price_BALTfinW1W3mean_C c.timew3w4##c.DASH_scorew1w3mean_C c.timew3w4##c.Agew1_C c.timew3w4##c.Agew3_C c.timew3w4##sex c.timew3w4##race c.timew3w4##pir c.timew3w4##edubr c.timew3w4##employed c.timew3w4##wrattbr c.timew3w4##smoke c.timew3w4##currdrugs c.timew3w4##c.bmi_C c.timew3w4##SRHbr c.timew3w4##c.kcalw1w3mean_C c.timew3w4##c.energystoresw1w3mean_C c.timew3w4##c.invmillsmixed if sample8==1 & race==1 || HNDID: timew3w4

//BELOW POVERTY//

xtmixed bpdia c.timew3w4##c.food_price_BALTfinW1W3mean_C c.timew3w4##c.DASH_scorew1w3mean_C c.timew3w4##c.Agew1_C c.timew3w4##c.Agew3_C c.timew3w4##sex c.timew3w4##race c.timew3w4##pir c.timew3w4##edubr c.timew3w4##employed c.timew3w4##wrattbr c.timew3w4##smoke c.timew3w4##currdrugs c.timew3w4##c.bmi_C c.timew3w4##SRHbr c.timew3w4##c.kcalw1w3mean_C c.timew3w4##c.energystoresw1w3mean_C c.timew3w4##c.invmillsmixed if sample8==1 & pir==0 || HNDID: timew3w4

//ABOVE POVERTY//

xtmixed bpdia c.timew3w4##c.food_price_BALTfinW1W3mean_C c.timew3w4##c.DASH_scorew1w3mean_C c.timew3w4##c.Agew1_C c.timew3w4##c.Agew3_C c.timew3w4##sex c.timew3w4##race c.timew3w4##pir c.timew3w4##edubr c.timew3w4##employed c.timew3w4##wrattbr c.timew3w4##smoke c.timew3w4##currdrugs c.timew3w4##c.bmi_C c.timew3w4##SRHbr c.timew3w4##c.kcalw1w3mean_C c.timew3w4##c.energystoresw1w3mean_C c.timew3w4##c.invmillsmixed if sample8==1 & pir==1 || HNDID: timew3w4

////////////////////HEART RATE//////////////////

//TOTAL SAMPLE//

xtmixed hr c.timew3w4##c.food_price_BALTfinW1W3mean_C c.timew3w4##c.DASH_scorew1w3mean_C c.timew3w4##c.Agew1_C c.timew3w4##c.Agew3_C c.timew3w4##sex c.timew3w4##race c.timew3w4##pir c.timew3w4##edubr c.timew3w4##employed c.timew3w4##wrattbr c.timew3w4##smoke c.timew3w4##currdrugs c.timew3w4##c.bmi_C c.timew3w4##SRHbr c.timew3w4##c.kcalw1w3mean_C c.timew3w4##c.energystoresw1w3mean_C c.timew3w4##c.invmillsmixed if sample8==1 || HNDID: timew3w4

//MEN//

```
xtmixed hr c.timew3w4##c.food_price_BALTfinW1W3mean_C c.timew3w4##c.DASH_scorew1w3mean_C c.timew3w4##c.Agew1_C c.timew3w4##c.Agew3_C c.timew3w4##sex c.timew3w4##race c.timew3w4##pir c.timew3w4##edubr c.timew3w4##employed c.timew3w4##wrattbr c.timew3w4##smoke c.timew3w4##currdrugs c.timew3w4##c.bmi_C c.timew3w4##SRHbr c.timew3w4##c.kcalw1w3mean_C c.timew3w4##c.energystoresw1w3mean_C c.timew3w4##c.invmillsmixed if sample8==1 & sex==1 || HNDID: timew3w4

//WOMEN//
xtmixed hr c.timew3w4##c.food_price_BALTfinW1W3mean_C c.timew3w4##c.DASH_scorew1w3mean_C c.timew3w4##c.Agew1_C c.timew3w4##c.Agew3_C c.timew3w4##sex c.timew3w4##race c.timew3w4##pir c.timew3w4##edubr c.timew3w4##employed c.timew3w4##wrattbr c.timew3w4##smoke c.timew3w4##currdrugs c.timew3w4##c.bmi_C c.timew3w4##SRHbr c.timew3w4##c.kcalw1w3mean_C c.timew3w4##c.energystoresw1w3mean_C c.timew3w4##c.invmillsmixed if sample8==1 & sex==0 || HNDID: timew3w4

//WHITES//
xtmixed hr c.timew3w4##c.food_price_BALTfinW1W3mean_C c.timew3w4##c.DASH_scorew1w3mean_C c.timew3w4##c.Agew1_C c.timew3w4##c.Agew3_C c.timew3w4##sex c.timew3w4##race c.timew3w4##pir c.timew3w4##edubr c.timew3w4##employed c.timew3w4##wrattbr c.timew3w4##smoke c.timew3w4##currdrugs c.timew3w4##c.bmi_C c.timew3w4##SRHbr c.timew3w4##c.kcalw1w3mean_C c.timew3w4##c.energystoresw1w3mean_C c.timew3w4##c.invmillsmixed if sample8==1 & race==0 || HNDID: timew3w4

//AA//
xtmixed hr c.timew3w4##c.food_price_BALTfinW1W3mean_C c.timew3w4##c.DASH_scorew1w3mean_C c.timew3w4##c.Agew1_C c.timew3w4##c.Agew3_C c.timew3w4##sex c.timew3w4##race c.timew3w4##pir c.timew3w4##edubr c.timew3w4##employed c.timew3w4##wrattbr c.timew3w4##smoke c.timew3w4##currdrugs c.timew3w4##c.bmi_C c.timew3w4##SRHbr c.timew3w4##c.kcalw1w3mean_C c.timew3w4##c.energystoresw1w3mean_C c.timew3w4##c.invmillsmixed if sample8==1 & race==1 || HNDID: timew3w4

//BELOW POVERTY//
xtmixed hr c.timew3w4##c.food_price_BALTfinW1W3mean_C c.timew3w4##c.DASH_scorew1w3mean_C c.timew3w4##c.Agew1_C c.timew3w4##c.Agew3_C c.timew3w4##sex c.timew3w4##race c.timew3w4##pir c.timew3w4##edubr c.timew3w4##employed c.timew3w4##wrattbr c.timew3w4##smoke c.timew3w4##currdrugs c.timew3w4##c.bmi_C c.timew3w4##SRHbr c.timew3w4##c.kcalw1w3mean_C c.timew3w4##c.energystoresw1w3mean_C c.timew3w4##c.invmillsmixed if sample8==1 & pir==0 || HNDID: timew3w4

//ABOVE POVERTY//
xtmixed hr c.timew3w4##c.food_price_BALTfinW1W3mean_C c.timew3w4##c.DASH_scorew1w3mean_C c.timew3w4##c.Agew1_C c.timew3w4##c.Agew3_C c.timew3w4##sex c.timew3w4##race c.timew3w4##pir c.timew3w4##edubr c.timew3w4##employed c.timew3w4##wrattbr c.timew3w4##smoke c.timew3w4##currdrugs c.timew3w4##c.bmi_C c.timew3w4##SRHbr c.timew3w4##c.kcalw1w3mean_C c.timew3w4##c.energystoresw1w3mean_C c.timew3w4##c.invmillsmixed if sample8==1 & pir==1 || HNDID: timew3w4


save, replace
```

////////////////////////FOOD COST ALONE: MODEL 2////////////////

//TOTAL SAMPLE//

xtmixed allostatic_prop c.timew3w4##c.food_price_BALTfinW1W3mean_C c.timew3w4##c.Agew1_C c.timew3w4##c.Agew3_C c.timew3w4##sex c.timew3w4##race c.timew3w4##pir c.timew3w4##edubr c.timew3w4##employed c.timew3w4##wrattbr c.timew3w4##smoke c.timew3w4##currdrugs c.timew3w4##c.bmi_C c.timew3w4##SRHbr c.timew3w4##c.kcalw1w3mean_C c.timew3w4##c.energystoresw1w3mean_C c.timew3w4##c.invmillsmixed if sample8==1 || HNDID: timew3w4

//MEN//

xtmixed allostatic_prop c.timew3w4##c.food_price_BALTfinW1W3mean_C c.timew3w4##c.Agew1_C c.timew3w4##c.Agew3_C c.timew3w4##sex c.timew3w4##race c.timew3w4##pir c.timew3w4##edubr c.timew3w4##employed c.timew3w4##wrattbr c.timew3w4##smoke c.timew3w4##currdrugs c.timew3w4##c.bmi_C c.timew3w4##SRHbr c.timew3w4##c.kcalw1w3mean_C c.timew3w4##c.energystoresw1w3mean_C c.timew3w4##c.invmillsmixed if sample8==1 & sex==1 || HNDID: timew3w4

//WOMEN//

xtmixed allostatic_prop c.timew3w4##c.food_price_BALTfinW1W3mean_C c.timew3w4##c.Agew1_C c.timew3w4##c.Agew3_C c.timew3w4##sex c.timew3w4##race c.timew3w4##pir c.timew3w4##edubr c.timew3w4##employed c.timew3w4##wrattbr c.timew3w4##smoke c.timew3w4##currdrugs c.timew3w4##c.bmi_C c.timew3w4##SRHbr c.timew3w4##c.kcalw1w3mean_C c.timew3w4##c.energystoresw1w3mean_C c.timew3w4##c.invmillsmixed if sample8==1 & sex==0 || HNDID: timew3w4

//WHITES//

xtmixed allostatic_prop c.timew3w4##c.food_price_BALTfinW1W3mean_C c.timew3w4##c.Agew1_C c.timew3w4##c.Agew3_C c.timew3w4##sex c.timew3w4##race c.timew3w4##pir c.timew3w4##edubr c.timew3w4##employed c.timew3w4##wrattbr c.timew3w4##smoke c.timew3w4##currdrugs c.timew3w4##c.bmi_C c.timew3w4##SRHbr c.timew3w4##c.kcalw1w3mean_C c.timew3w4##c.energystoresw1w3mean_C c.timew3w4##c.invmillsmixed if sample8==1 & race==0 || HNDID: timew3w4

//AA//

xtmixed allostatic_prop c.timew3w4##c.food_price_BALTfinW1W3mean_C c.timew3w4##c.Agew1_C c.timew3w4##c.Agew3_C c.timew3w4##sex c.timew3w4##race c.timew3w4##pir c.timew3w4##edubr c.timew3w4##employed c.timew3w4##wrattbr c.timew3w4##smoke c.timew3w4##currdrugs c.timew3w4##c.bmi_C c.timew3w4##SRHbr c.timew3w4##c.kcalw1w3mean_C c.timew3w4##c.energystoresw1w3mean_C c.timew3w4##c.invmillsmixed if sample8==1 & race==1 || HNDID: timew3w4

//BELOW POVERTY//

xtmixed allostatic_prop c.timew3w4##c.food_price_BALTfinW1W3mean_C c.timew3w4##c.Agew1_C c.timew3w4##c.Agew3_C c.timew3w4##sex c.timew3w4##race c.timew3w4##pir c.timew3w4##edubr c.timew3w4##employed c.timew3w4##wrattbr c.timew3w4##smoke c.timew3w4##currdrugs c.timew3w4##c.bmi_C

c.timew3w4##SRHbr c.timew3w4##c.kcalw1w3mean_C c.timew3w4##c.energystoresw1w3mean_C c.timew3w4##c.invmillsmixed if sample8==1 & pir==0 || HNDID: timew3w4

//ABOVE POVERTY//

xtmixed allostatic_prop c.timew3w4##c.food_price_BALTfinW1W3mean_C c.timew3w4##c.Agew1_C c.timew3w4##c.Agew3_C c.timew3w4##sex c.timew3w4##race c.timew3w4##pir c.timew3w4##edubr c.timew3w4##employed c.timew3w4##wrattbr c.timew3w4##smoke c.timew3w4##currdrugs c.timew3w4##c.bmi_C c.timew3w4##SRHbr c.timew3w4##c.kcalw1w3mean_C c.timew3w4##c.energystoresw1w3mean_C c.timew3w4##c.invmillsmixed if sample8==1 & pir==1 || HNDID: timew3w4

Components of Allostatic load

//albumin crp chol hdl hgba1c whr bpsys bpdia hr//

****Albumin**********

//TOTAL SAMPLE//

xtmixed albumin c.timew3w4##c.food_price_BALTfinW1W3mean_C c.timew3w4##c.Agew1_C c.timew3w4##c.Agew3_C c.timew3w4##sex c.timew3w4##race c.timew3w4##pir c.timew3w4##edubr c.timew3w4##employed c.timew3w4##wrattbr c.timew3w4##smoke c.timew3w4##currdrugs c.timew3w4##c.bmi_C c.timew3w4##SRHbr c.timew3w4##c.kcalw1w3mean_C c.timew3w4##c.energystoresw1w3mean_C c.timew3w4##c.invmillsmixed if sample8==1 || HNDID: timew3w4

//MEN//

xtmixed albumin c.timew3w4##c.food_price_BALTfinW1W3mean_C c.timew3w4##c.Agew1_C c.timew3w4##c.Agew3_C c.timew3w4##sex c.timew3w4##race c.timew3w4##pir c.timew3w4##edubr c.timew3w4##employed c.timew3w4##wrattbr c.timew3w4##smoke c.timew3w4##currdrugs c.timew3w4##c.bmi_C c.timew3w4##SRHbr c.timew3w4##c.kcalw1w3mean_C c.timew3w4##c.energystoresw1w3mean_C c.timew3w4##c.invmillsmixed if sample8==1 & sex==1 || HNDID: timew3w4

//WOMEN//

xtmixed albumin c.timew3w4##c.food_price_BALTfinW1W3mean_C c.timew3w4##c.Agew1_C c.timew3w4##c.Agew3_C c.timew3w4##sex c.timew3w4##race c.timew3w4##pir c.timew3w4##edubr c.timew3w4##employed c.timew3w4##wrattbr c.timew3w4##smoke c.timew3w4##currdrugs c.timew3w4##c.bmi_C c.timew3w4##SRHbr c.timew3w4##c.kcalw1w3mean_C c.timew3w4##c.energystoresw1w3mean_C c.timew3w4##c.invmillsmixed if sample8==1 & sex==0 || HNDID: timew3w4

//WHITES//

xtmixed albumin c.timew3w4##c.food_price_BALTfinW1W3mean_C c.timew3w4##c.Agew1_C c.timew3w4##c.Agew3_C c.timew3w4##sex c.timew3w4##race c.timew3w4##pir c.timew3w4##edubr c.timew3w4##employed c.timew3w4##wrattbr c.timew3w4##smoke c.timew3w4##currdrugs c.timew3w4##c.bmi_C c.timew3w4##SRHbr c.timew3w4##c.kcalw1w3mean_C c.timew3w4##c.energystoresw1w3mean_C c.timew3w4##c.invmillsmixed if sample8==1 & race==0 || HNDID: timew3w4

//AA//

xtmixed albumin c.timew3w4##c.food_price_BALTfinW1W3mean_C c.timew3w4##c.Agew1_C c.timew3w4##c.Agew3_C c.timew3w4##sex c.timew3w4##race c.timew3w4##pir c.timew3w4##edubr c.timew3w4##employed c.timew3w4##wrattbr

c.timew3w4##smoke c.timew3w4##currdrugs c.timew3w4##c.bmi_C c.timew3w4##SRHbr c.timew3w4##c.kcalw1w3mean_C c.timew3w4##c.energystoresw1w3mean_C c.timew3w4##c.invmillsmixed if sample8==1 & race==1 || HNDID: timew3w4

//BELOW POVERTY//

xtmixed albumin c.timew3w4##c.food_price_BALTfinW1W3mean_C c.timew3w4##c.Agew1_C c.timew3w4##c.Agew3_C c.timew3w4##sex c.timew3w4##race c.timew3w4##pir c.timew3w4##edubr c.timew3w4##employed c.timew3w4##wrattbr c.timew3w4##smoke c.timew3w4##currdrugs c.timew3w4##c.bmi_C c.timew3w4##SRHbr c.timew3w4##c.kcalw1w3mean_C c.timew3w4##c.energystoresw1w3mean_C c.timew3w4##c.invmillsmixed if sample8==1 & pir==0 || HNDID: timew3w4

//ABOVE POVERTY//

xtmixed albumin c.timew3w4##c.food_price_BALTfinW1W3mean_C c.timew3w4##c.Agew1_C c.timew3w4##c.Agew3_C c.timew3w4##sex c.timew3w4##race c.timew3w4##pir c.timew3w4##edubr c.timew3w4##employed c.timew3w4##wrattbr c.timew3w4##smoke c.timew3w4##currdrugs c.timew3w4##c.bmi_C c.timew3w4##SRHbr c.timew3w4##c.kcalw1w3mean_C c.timew3w4##c.energystoresw1w3mean_C c.timew3w4##c.invmillsmixed if sample8==1 & pir==1 || HNDID: timew3w4

*****C-reactive protein*******

//TOTAL SAMPLE//

xtmixed crp c.timew3w4##c.food_price_BALTfinW1W3mean_C c.timew3w4##c.Agew1_C c.timew3w4##c.Agew3_C c.timew3w4##sex c.timew3w4##race c.timew3w4##pir c.timew3w4##edubr c.timew3w4##employed c.timew3w4##wrattbr c.timew3w4##smoke c.timew3w4##currdrugs c.timew3w4##c.bmi_C c.timew3w4##SRHbr c.timew3w4##c.kcalw1w3mean_C c.timew3w4##c.energystoresw1w3mean_C c.timew3w4##c.invmillsmixed if sample8==1 || HNDID: timew3w4

//MEN//

xtmixed crp c.timew3w4##c.food_price_BALTfinW1W3mean_C c.timew3w4##c.Agew1_C c.timew3w4##c.Agew3_C c.timew3w4##sex c.timew3w4##race c.timew3w4##pir c.timew3w4##edubr c.timew3w4##employed c.timew3w4##wrattbr c.timew3w4##smoke c.timew3w4##currdrugs c.timew3w4##c.bmi_C c.timew3w4##SRHbr c.timew3w4##c.kcalw1w3mean_C c.timew3w4##c.energystoresw1w3mean_C c.timew3w4##c.invmillsmixed if sample8==1 & sex==1 || HNDID: timew3w4

//WOMEN//

xtmixed crp c.timew3w4##c.food_price_BALTfinW1W3mean_C c.timew3w4##c.Agew1_C c.timew3w4##c.Agew3_C c.timew3w4##sex c.timew3w4##race c.timew3w4##pir c.timew3w4##edubr c.timew3w4##employed c.timew3w4##wrattbr c.timew3w4##smoke c.timew3w4##currdrugs c.timew3w4##c.bmi_C c.timew3w4##SRHbr c.timew3w4##c.kcalw1w3mean_C c.timew3w4##c.energystoresw1w3mean_C c.timew3w4##c.invmillsmixed if sample8==1 & sex==0 || HNDID: timew3w4

//WHITES//

xtmixed crp c.timew3w4##c.food_price_BALTfinW1W3mean_C c.timew3w4##c.Agew1_C c.timew3w4##c.Agew3_C c.timew3w4##sex c.timew3w4##race c.timew3w4##pir c.timew3w4##edubr c.timew3w4##employed c.timew3w4##wrattbr c.timew3w4##smoke c.timew3w4##currdrugs c.timew3w4##c.bmi_C c.timew3w4##SRHbr c.timew3w4##c.kcalw1w3mean_C c.timew3w4##c.energystoresw1w3mean_C c.timew3w4##c.invmillsmixed if sample8==1 & race==0 || HNDID: timew3w4

//AA//

xtmixed crp c.timew3w4##c.food_price_BALTfinW1W3mean_C c.timew3w4##c.Agew1_C c.timew3w4##c.Agew3_C c.timew3w4##sex c.timew3w4##race c.timew3w4##pir c.timew3w4##edubr c.timew3w4##employed c.timew3w4##wrattbr c.timew3w4##smoke c.timew3w4##currdrugs c.timew3w4##c.bmi_C c.timew3w4##SRHbr c.timew3w4##c.kcalw1w3mean_C c.timew3w4##c.energystoresw1w3mean_C c.timew3w4##c.invmillsmixed if sample8==1 & race==1 || HNDID: timew3w4

//BELOW POVERTY//

xtmixed crp c.timew3w4##c.food_price_BALTfinW1W3mean_C c.timew3w4##c.Agew1_C c.timew3w4##c.Agew3_C c.timew3w4##sex c.timew3w4##race c.timew3w4##pir c.timew3w4##edubr c.timew3w4##employed c.timew3w4##wrattbr c.timew3w4##smoke c.timew3w4##currdrugs c.timew3w4##c.bmi_C c.timew3w4##SRHbr c.timew3w4##c.kcalw1w3mean_C c.timew3w4##c.energystoresw1w3mean_C c.timew3w4##c.invmillsmixed if sample8==1 & pir==0 || HNDID: timew3w4

//ABOVE POVERTY//

xtmixed crp c.timew3w4##c.food_price_BALTfinW1W3mean_C c.timew3w4##c.Agew1_C c.timew3w4##c.Agew3_C c.timew3w4##sex c.timew3w4##race c.timew3w4##pir c.timew3w4##edubr c.timew3w4##employed c.timew3w4##wrattbr c.timew3w4##smoke c.timew3w4##currdrugs c.timew3w4##c.bmi_C c.timew3w4##SRHbr c.timew3w4##c.kcalw1w3mean_C c.timew3w4##c.energystoresw1w3mean_C c.timew3w4##c.invmillsmixed if sample8==1 & pir==1 || HNDID: timew3w4

***********Cholesterol***************

//TOTAL SAMPLE//

xtmixed chol c.timew3w4##c.food_price_BALTfinW1W3mean_C c.timew3w4##c.Agew1_C c.timew3w4##c.Agew3_C c.timew3w4##sex c.timew3w4##race c.timew3w4##pir c.timew3w4##edubr c.timew3w4##employed c.timew3w4##wrattbr c.timew3w4##smoke c.timew3w4##currdrugs c.timew3w4##c.bmi_C c.timew3w4##SRHbr c.timew3w4##c.kcalw1w3mean_C c.timew3w4##c.energystoresw1w3mean_C c.timew3w4##c.invmillsmixed if sample8==1 || HNDID: timew3w4

//MEN//

xtmixed chol c.timew3w4##c.food_price_BALTfinW1W3mean_C c.timew3w4##c.Agew1_C c.timew3w4##c.Agew3_C c.timew3w4##sex c.timew3w4##race c.timew3w4##pir c.timew3w4##edubr c.timew3w4##employed c.timew3w4##wrattbr c.timew3w4##smoke c.timew3w4##currdrugs c.timew3w4##c.bmi_C c.timew3w4##SRHbr c.timew3w4##c.kcalw1w3mean_C c.timew3w4##c.energystoresw1w3mean_C c.timew3w4##c.invmillsmixed if sample8==1 & sex==1 || HNDID: timew3w4

//WOMEN//

xtmixed chol c.timew3w4##c.food_price_BALTfinW1W3mean_C c.timew3w4##c.Agew1_C c.timew3w4##c.Agew3_C c.timew3w4##sex c.timew3w4##race c.timew3w4##pir c.timew3w4##edubr c.timew3w4##employed c.timew3w4##wrattbr c.timew3w4##smoke c.timew3w4##currdrugs c.timew3w4##c.bmi_C c.timew3w4##SRHbr c.timew3w4##c.kcalw1w3mean_C c.timew3w4##c.energystoresw1w3mean_C c.timew3w4##c.invmillsmixed if sample8==1 & sex==0 || HNDID: timew3w4

//WHITES//

xtmixed chol c.timew3w4##c.food_price_BALTfinW1W3mean_C c.timew3w4##c.Agew1_C c.timew3w4##c.Agew3_C c.timew3w4##sex c.timew3w4##race c.timew3w4##pir c.timew3w4##edubr c.timew3w4##employed c.timew3w4##wrattbr

c.timew3w4##smoke c.timew3w4##currdrugs c.timew3w4##c.bmi_C c.timew3w4##SRHbr c.timew3w4##c.kcalw1w3mean_C c.timew3w4##c.energystoresw1w3mean_C c.timew3w4##c.invmillsmixed if sample8==1 & race==0 || HNDID: timew3w4

//AA//

xtmixed chol c.timew3w4##c.food_price_BALTfinW1W3mean_C c.timew3w4##c.Agew1_C c.timew3w4##c.Agew3_C c.timew3w4##sex c.timew3w4##race c.timew3w4##pir c.timew3w4##edubr c.timew3w4##employed c.timew3w4##wrattbr c.timew3w4##smoke c.timew3w4##currdrugs c.timew3w4##c.bmi_C c.timew3w4##SRHbr c.timew3w4##c.kcalw1w3mean_C c.timew3w4##c.energystoresw1w3mean_C c.timew3w4##c.invmillsmixed if sample8==1 & race==1 || HNDID: timew3w4

//BELOW POVERTY//

xtmixed chol c.timew3w4##c.food_price_BALTfinW1W3mean_C c.timew3w4##c.Agew1_C c.timew3w4##c.Agew3_C c.timew3w4##sex c.timew3w4##race c.timew3w4##pir c.timew3w4##edubr c.timew3w4##employed c.timew3w4##wrattbr c.timew3w4##smoke c.timew3w4##currdrugs c.timew3w4##c.bmi_C c.timew3w4##SRHbr c.timew3w4##c.kcalw1w3mean_C c.timew3w4##c.energystoresw1w3mean_C c.timew3w4##c.invmillsmixed if sample8==1 & pir==0 || HNDID: timew3w4

//ABOVE POVERTY//

xtmixed chol c.timew3w4##c.food_price_BALTfinW1W3mean_C c.timew3w4##c.Agew1_C c.timew3w4##c.Agew3_C c.timew3w4##sex c.timew3w4##race c.timew3w4##pir c.timew3w4##edubr c.timew3w4##employed c.timew3w4##wrattbr c.timew3w4##smoke c.timew3w4##currdrugs c.timew3w4##c.bmi_C c.timew3w4##SRHbr c.timew3w4##c.kcalw1w3mean_C c.timew3w4##c.energystoresw1w3mean_C c.timew3w4##c.invmillsmixed if sample8==1 & pir==1 || HNDID: timew3w4

***************HDL-C*******************

//TOTAL SAMPLE//

xtmixed hdl c.timew3w4##c.food_price_BALTfinW1W3mean_C c.timew3w4##c.Agew1_C c.timew3w4##c.Agew3_C c.timew3w4##sex c.timew3w4##race c.timew3w4##pir c.timew3w4##edubr c.timew3w4##employed c.timew3w4##wrattbr c.timew3w4##smoke c.timew3w4##currdrugs c.timew3w4##c.bmi_C c.timew3w4##SRHbr c.timew3w4##c.kcalw1w3mean_C c.timew3w4##c.energystoresw1w3mean_C c.timew3w4##c.invmillsmixed if sample8==1 || HNDID: timew3w4

//MEN//

xtmixed hdl c.timew3w4##c.food_price_BALTfinW1W3mean_C c.timew3w4##c.Agew1_C c.timew3w4##c.Agew3_C c.timew3w4##sex c.timew3w4##race c.timew3w4##pir c.timew3w4##edubr c.timew3w4##employed c.timew3w4##wrattbr c.timew3w4##smoke c.timew3w4##currdrugs c.timew3w4##c.bmi_C c.timew3w4##SRHbr c.timew3w4##c.kcalw1w3mean_C c.timew3w4##c.energystoresw1w3mean_C c.timew3w4##c.invmillsmixed if sample8==1 & sex==1 || HNDID: timew3w4

//WOMEN//

xtmixed hdl c.timew3w4##c.food_price_BALTfinW1W3mean_C c.timew3w4##c.Agew1_C c.timew3w4##c.Agew3_C c.timew3w4##sex c.timew3w4##race c.timew3w4##pir c.timew3w4##edubr c.timew3w4##employed c.timew3w4##wrattbr c.timew3w4##smoke c.timew3w4##currdrugs c.timew3w4##c.bmi_C c.timew3w4##SRHbr c.timew3w4##c.kcalw1w3mean_C c.timew3w4##c.energystoresw1w3mean_C c.timew3w4##c.invmillsmixed if sample8==1 & sex==0 || HNDID: timew3w4

//WHITES//

xtmixed hdl c.timew3w4##c.food_price_BALTfinW1W3mean_C c.timew3w4##c.Agew1_C c.timew3w4##c.Agew3_C c.timew3w4##sex c.timew3w4##race c.timew3w4##pir c.timew3w4##edubr c.timew3w4##employed c.timew3w4##wrattbr c.timew3w4##smoke c.timew3w4##currdrugs c.timew3w4##c.bmi_C c.timew3w4##SRHbr c.timew3w4##c.kcalw1w3mean_C c.timew3w4##c.energystoresw1w3mean_C c.timew3w4##c.invmillsmixed if sample8==1 & race==0 || HNDID: timew3w4

//AA//

xtmixed hdl c.timew3w4##c.food_price_BALTfinW1W3mean_C c.timew3w4##c.Agew1_C c.timew3w4##c.Agew3_C c.timew3w4##sex c.timew3w4##race c.timew3w4##pir c.timew3w4##edubr c.timew3w4##employed c.timew3w4##wrattbr c.timew3w4##smoke c.timew3w4##currdrugs c.timew3w4##c.bmi_C c.timew3w4##SRHbr c.timew3w4##c.kcalw1w3mean_C c.timew3w4##c.energystoresw1w3mean_C c.timew3w4##c.invmillsmixed if sample8==1 & race==1 || HNDID: timew3w4

//BELOW POVERTY//

xtmixed hdl c.timew3w4##c.food_price_BALTfinW1W3mean_C c.timew3w4##c.Agew1_C c.timew3w4##c.Agew3_C c.timew3w4##sex c.timew3w4##race c.timew3w4##pir c.timew3w4##edubr c.timew3w4##employed c.timew3w4##wrattbr c.timew3w4##smoke c.timew3w4##currdrugs c.timew3w4##c.bmi_C c.timew3w4##SRHbr c.timew3w4##c.kcalw1w3mean_C c.timew3w4##c.energystoresw1w3mean_C c.timew3w4##c.invmillsmixed if sample8==1 & pir==0 || HNDID: timew3w4

//ABOVE POVERTY//

xtmixed hdl c.timew3w4##c.food_price_BALTfinW1W3mean_C c.timew3w4##c.Agew1_C c.timew3w4##c.Agew3_C c.timew3w4##sex c.timew3w4##race c.timew3w4##pir c.timew3w4##edubr c.timew3w4##employed c.timew3w4##wrattbr c.timew3w4##smoke c.timew3w4##currdrugs c.timew3w4##c.bmi_C c.timew3w4##SRHbr c.timew3w4##c.kcalw1w3mean_C c.timew3w4##c.energystoresw1w3mean_C c.timew3w4##c.invmillsmixed if sample8==1 & pir==1 || HNDID: timew3w4

//////////////GLYCATED HEMOGLOBIN//////////////

//TOTAL SAMPLE//

xtmixed hgba1c c.timew3w4##c.food_price_BALTfinW1W3mean_C c.timew3w4##c.Agew1_C c.timew3w4##c.Agew3_C c.timew3w4##sex c.timew3w4##race c.timew3w4##pir c.timew3w4##edubr c.timew3w4##employed c.timew3w4##wrattbr c.timew3w4##smoke c.timew3w4##currdrugs c.timew3w4##c.bmi_C c.timew3w4##SRHbr c.timew3w4##c.kcalw1w3mean_C c.timew3w4##c.energystoresw1w3mean_C c.timew3w4##c.invmillsmixed if sample8==1 || HNDID: timew3w4

//MEN//

xtmixed hgba1c c.timew3w4##c.food_price_BALTfinW1W3mean_C c.timew3w4##c.Agew1_C c.timew3w4##c.Agew3_C c.timew3w4##sex c.timew3w4##race c.timew3w4##pir c.timew3w4##edubr c.timew3w4##employed c.timew3w4##wrattbr c.timew3w4##smoke c.timew3w4##currdrugs c.timew3w4##c.bmi_C c.timew3w4##SRHbr c.timew3w4##c.kcalw1w3mean_C c.timew3w4##c.energystoresw1w3mean_C c.timew3w4##c.invmillsmixed if sample8==1 & sex==1 || HNDID: timew3w4

//WOMEN//

xtmixed hgba1c c.timew3w4##c.food_price_BALTfinW1W3mean_C c.timew3w4##c.Agew1_C c.timew3w4##c.Agew3_C c.timew3w4##sex c.timew3w4##race c.timew3w4##pir c.timew3w4##edubr c.timew3w4##employed c.timew3w4##wrattbr

c.timew3w4##smoke c.timew3w4##currdrugs c.timew3w4##c.bmi_C c.timew3w4##SRHbr c.timew3w4##c.kcalw1w3mean_C c.timew3w4##c.energystoresw1w3mean_C c.timew3w4##c.invmillsmixed if sample8==1 & sex==0 || HNDID: timew3w4

//WHITES//

xtmixed hgba1c c.timew3w4##c.food_price_BALTfinW1W3mean_C c.timew3w4##c.Agew1_C c.timew3w4##c.Agew3_C c.timew3w4##sex c.timew3w4##race c.timew3w4##pir c.timew3w4##edubr c.timew3w4##employed c.timew3w4##wrattbr c.timew3w4##smoke c.timew3w4##currdrugs c.timew3w4##c.bmi_C c.timew3w4##SRHbr c.timew3w4##c.kcalw1w3mean_C c.timew3w4##c.energystoresw1w3mean_C c.timew3w4##c.invmillsmixed if sample8==1 & race==0 || HNDID: timew3w4

//AA//

xtmixed hgba1c c.timew3w4##c.food_price_BALTfinW1W3mean_C c.timew3w4##c.Agew1_C c.timew3w4##c.Agew3_C c.timew3w4##sex c.timew3w4##race c.timew3w4##pir c.timew3w4##edubr c.timew3w4##employed c.timew3w4##wrattbr c.timew3w4##smoke c.timew3w4##currdrugs c.timew3w4##c.bmi_C c.timew3w4##SRHbr c.timew3w4##c.kcalw1w3mean_C c.timew3w4##c.energystoresw1w3mean_C c.timew3w4##c.invmillsmixed if sample8==1 & race==1 || HNDID: timew3w4

//BELOW POVERTY//

xtmixed hgba1c c.timew3w4##c.food_price_BALTfinW1W3mean_C c.timew3w4##c.Agew1_C c.timew3w4##c.Agew3_C c.timew3w4##sex c.timew3w4##race c.timew3w4##pir c.timew3w4##edubr c.timew3w4##employed c.timew3w4##wrattbr c.timew3w4##smoke c.timew3w4##currdrugs c.timew3w4##c.bmi_C c.timew3w4##SRHbr c.timew3w4##c.kcalw1w3mean_C c.timew3w4##c.energystoresw1w3mean_C c.timew3w4##c.invmillsmixed if sample8==1 & pir==0 || HNDID: timew3w4

//ABOVE POVERTY//

xtmixed hgba1c c.timew3w4##c.food_price_BALTfinW1W3mean_C c.timew3w4##c.Agew1_C c.timew3w4##c.Agew3_C c.timew3w4##sex c.timew3w4##race c.timew3w4##pir c.timew3w4##edubr c.timew3w4##employed c.timew3w4##wrattbr c.timew3w4##smoke c.timew3w4##currdrugs c.timew3w4##c.bmi_C c.timew3w4##SRHbr c.timew3w4##c.kcalw1w3mean_C c.timew3w4##c.energystoresw1w3mean_C c.timew3w4##c.invmillsmixed if sample8==1 & pir==1 || HNDID: timew3w4

////////////////WHR////////////////

//TOTAL SAMPLE//

xtmixed whr c.timew3w4##c.food_price_BALTfinW1W3mean_C c.timew3w4##c.Agew1_C c.timew3w4##c.Agew3_C c.timew3w4##sex c.timew3w4##race c.timew3w4##pir c.timew3w4##edubr c.timew3w4##employed c.timew3w4##wrattbr c.timew3w4##smoke c.timew3w4##currdrugs c.timew3w4##c.bmi_C c.timew3w4##SRHbr c.timew3w4##c.kcalw1w3mean_C c.timew3w4##c.energystoresw1w3mean_C c.timew3w4##c.invmillsmixed if sample8==1 || HNDID: timew3w4

//MEN//

xtmixed whr c.timew3w4##c.food_price_BALTfinW1W3mean_C c.timew3w4##c.Agew1_C c.timew3w4##c.Agew3_C c.timew3w4##sex c.timew3w4##race c.timew3w4##pir c.timew3w4##edubr c.timew3w4##employed c.timew3w4##wrattbr c.timew3w4##smoke c.timew3w4##currdrugs c.timew3w4##c.bmi_C c.timew3w4##SRHbr c.timew3w4##c.kcalw1w3mean_C c.timew3w4##c.energystoresw1w3mean_C c.timew3w4##c.invmillsmixed if sample8==1 & sex==1 || HNDID: timew3w4

//WOMEN//

xtmixed whr c.timew3w4##c.food_price_BALTfinW1W3mean_C c.timew3w4##c.Agew1_C c.timew3w4##c.Agew3_C c.timew3w4##sex c.timew3w4##race c.timew3w4##pir c.timew3w4##edubr c.timew3w4##employed c.timew3w4##wrattbr c.timew3w4##smoke c.timew3w4##currdrugs c.timew3w4##c.bmi_C c.timew3w4##SRHbr c.timew3w4##c.kcalw1w3mean_C c.timew3w4##c.energystoresw1w3mean_C c.timew3w4##c.invmillsmixed if sample8==1 & sex==0 || HNDID: timew3w4

//WHITES//

xtmixed whr c.timew3w4##c.food_price_BALTfinW1W3mean_C c.timew3w4##c.Agew1_C c.timew3w4##c.Agew3_C c.timew3w4##sex c.timew3w4##race c.timew3w4##pir c.timew3w4##edubr c.timew3w4##employed c.timew3w4##wrattbr c.timew3w4##smoke c.timew3w4##currdrugs c.timew3w4##c.bmi_C c.timew3w4##SRHbr c.timew3w4##c.kcalw1w3mean_C c.timew3w4##c.energystoresw1w3mean_C c.timew3w4##c.invmillsmixed if sample8==1 & race==0 || HNDID: timew3w4

//AA//

xtmixed whr c.timew3w4##c.food_price_BALTfinW1W3mean_C c.timew3w4##c.Agew1_C c.timew3w4##c.Agew3_C c.timew3w4##sex c.timew3w4##race c.timew3w4##pir c.timew3w4##edubr c.timew3w4##employed c.timew3w4##wrattbr c.timew3w4##smoke c.timew3w4##currdrugs c.timew3w4##c.bmi_C c.timew3w4##SRHbr c.timew3w4##c.kcalw1w3mean_C c.timew3w4##c.energystoresw1w3mean_C c.timew3w4##c.invmillsmixed if sample8==1 & race==1 || HNDID: timew3w4

//BELOW POVERTY//

xtmixed whr c.timew3w4##c.food_price_BALTfinW1W3mean_C c.timew3w4##c.Agew1_C c.timew3w4##c.Agew3_C c.timew3w4##sex c.timew3w4##race c.timew3w4##pir c.timew3w4##edubr c.timew3w4##employed c.timew3w4##wrattbr c.timew3w4##smoke c.timew3w4##currdrugs c.timew3w4##c.bmi_C c.timew3w4##SRHbr c.timew3w4##c.kcalw1w3mean_C c.timew3w4##c.energystoresw1w3mean_C c.timew3w4##c.invmillsmixed if sample8==1 & pir==0 || HNDID: timew3w4

//ABOVE POVERTY//

xtmixed whr c.timew3w4##c.food_price_BALTfinW1W3mean_C c.timew3w4##c.Agew1_C c.timew3w4##c.Agew3_C c.timew3w4##sex c.timew3w4##race c.timew3w4##pir c.timew3w4##edubr c.timew3w4##employed c.timew3w4##wrattbr c.timew3w4##smoke c.timew3w4##currdrugs c.timew3w4##c.bmi_C c.timew3w4##SRHbr c.timew3w4##c.kcalw1w3mean_C c.timew3w4##c.energystoresw1w3mean_C c.timew3w4##c.invmillsmixed if sample8==1 & pir==1 || HNDID: timew3w4

//////////////////////////SYSTOLIC BLOOD PRESSURE////////////////////

//TOTAL SAMPLE//

xtmixed bpsys c.timew3w4##c.food_price_BALTfinW1W3mean_C c.timew3w4##c.Agew1_C c.timew3w4##c.Agew3_C c.timew3w4##sex c.timew3w4##race c.timew3w4##pir c.timew3w4##edubr c.timew3w4##employed c.timew3w4##wrattbr c.timew3w4##smoke c.timew3w4##currdrugs c.timew3w4##c.bmi_C c.timew3w4##SRHbr c.timew3w4##c.kcalw1w3mean_C c.timew3w4##c.energystoresw1w3mean_C c.timew3w4##c.invmillsmixed if sample8==1 || HNDID: timew3w4

//MEN//

xtmixed bpsys c.timew3w4##c.food_price_BALTfinW1W3mean_C c.timew3w4##c.Agew1_C c.timew3w4##c.Agew3_C c.timew3w4##sex c.timew3w4##race c.timew3w4##pir c.timew3w4##edubr c.timew3w4##employed c.timew3w4##wrattbr c.timew3w4##smoke c.timew3w4##currdrugs c.timew3w4##c.bmi_C c.timew3w4##SRHbr c.timew3w4##c.kcalw1w3mean_C c.timew3w4##c.energystoresw1w3mean_C c.timew3w4##c.invmillsmixed if sample8==1 & sex==1 || HNDID: timew3w4

//WOMEN//

```
xtmixed bpsys c.timew3w4##c.food_price_BALTfinW1W3mean_C c.timew3w4##c.Agew1_C c.timew3w4##c.Agew3_C c.timew3w4##sex c.timew3w4##race c.timew3w4##pir c.timew3w4##edubr c.timew3w4##employed c.timew3w4##wrattbr c.timew3w4##smoke c.timew3w4##currdrugs c.timew3w4##c.bmi_C c.timew3w4##SRHbr c.timew3w4##c.kcalw1w3mean_C c.timew3w4##c.energystoresw1w3mean_C c.timew3w4##c.invmillsmixed if sample8==1 & sex==0 || HNDID: timew3w4
```

//WHITES//

```
xtmixed bpsys c.timew3w4##c.food_price_BALTfinW1W3mean_C c.timew3w4##c.Agew1_C c.timew3w4##c.Agew3_C c.timew3w4##sex c.timew3w4##race c.timew3w4##pir c.timew3w4##edubr c.timew3w4##employed c.timew3w4##wrattbr c.timew3w4##smoke c.timew3w4##currdrugs c.timew3w4##c.bmi_C c.timew3w4##SRHbr c.timew3w4##c.kcalw1w3mean_C c.timew3w4##c.energystoresw1w3mean_C c.timew3w4##c.invmillsmixed if sample8==1 & race==0 || HNDID: timew3w4
```

//AA//

```
xtmixed bpsys c.timew3w4##c.food_price_BALTfinW1W3mean_C c.timew3w4##c.Agew1_C c.timew3w4##c.Agew3_C c.timew3w4##sex c.timew3w4##race c.timew3w4##pir c.timew3w4##edubr c.timew3w4##employed c.timew3w4##wrattbr c.timew3w4##smoke c.timew3w4##currdrugs c.timew3w4##c.bmi_C c.timew3w4##SRHbr c.timew3w4##c.kcalw1w3mean_C c.timew3w4##c.energystoresw1w3mean_C c.timew3w4##c.invmillsmixed if sample8==1 & race==1 || HNDID: timew3w4
```

//BELOW POVERTY//

```
xtmixed bpsys c.timew3w4##c.food_price_BALTfinW1W3mean_C c.timew3w4##c.Agew1_C c.timew3w4##c.Agew3_C c.timew3w4##sex c.timew3w4##race c.timew3w4##pir c.timew3w4##edubr c.timew3w4##employed c.timew3w4##wrattbr c.timew3w4##smoke c.timew3w4##currdrugs c.timew3w4##c.bmi_C c.timew3w4##SRHbr c.timew3w4##c.kcalw1w3mean_C c.timew3w4##c.energystoresw1w3mean_C c.timew3w4##c.invmillsmixed if sample8==1 & pir==0 || HNDID: timew3w4
```

//ABOVE POVERTY//

```
xtmixed bpsys c.timew3w4##c.food_price_BALTfinW1W3mean_C c.timew3w4##c.Agew1_C c.timew3w4##c.Agew3_C c.timew3w4##sex c.timew3w4##race c.timew3w4##pir c.timew3w4##edubr c.timew3w4##employed c.timew3w4##wrattbr c.timew3w4##smoke c.timew3w4##currdrugs c.timew3w4##c.bmi_C c.timew3w4##SRHbr c.timew3w4##c.kcalw1w3mean_C c.timew3w4##c.energystoresw1w3mean_C c.timew3w4##c.invmillsmixed if sample8==1 & pir==1 || HNDID: timew3w4
```

////////////////////////DIASTOLIC BLOOD PRESSURE////////////

//TOTAL SAMPLE//

```
xtmixed bpdia c.timew3w4##c.food_price_BALTfinW1W3mean_C c.timew3w4##c.Agew1_C c.timew3w4##c.Agew3_C c.timew3w4##sex c.timew3w4##race c.timew3w4##pir c.timew3w4##edubr c.timew3w4##employed c.timew3w4##wrattbr c.timew3w4##smoke c.timew3w4##currdrugs c.timew3w4##c.bmi_C c.timew3w4##SRHbr c.timew3w4##c.kcalw1w3mean_C c.timew3w4##c.energystoresw1w3mean_C c.timew3w4##c.invmillsmixed if sample8==1 || HNDID: timew3w4
```

//MEN//

```
xtmixed bpdia c.timew3w4##c.food_price_BALTfinW1W3mean_C c.timew3w4##c.Agew1_C c.timew3w4##c.Agew3_C c.timew3w4##sex c.timew3w4##race c.timew3w4##pir c.timew3w4##edubr c.timew3w4##employed c.timew3w4##wrattbr c.timew3w4##smoke c.timew3w4##currdrugs c.timew3w4##c.bmi_C c.timew3w4##SRHbr c.timew3w4##c.kcalw1w3mean_C c.timew3w4##c.energystoresw1w3mean_C c.timew3w4##c.invmillsmixed if sample8==1 & sex==1 || HNDID: timew3w4
```

```
//WOMEN//
xtmixed bpdia c.timew3w4##c.food_price_BALTfinW1W3mean_C c.timew3w4##c.Agew1_C c.timew3w4##c.Agew3_C
c.timew3w4##sex c.timew3w4##race c.timew3w4##pir c.timew3w4##edubr c.timew3w4##employed c.timew3w4##wrattbr
c.timew3w4##smoke c.timew3w4##currdrugs c.timew3w4##c.bmi_C c.timew3w4##SRHbr c.timew3w4##c.kcalw1w3mean_C
c.timew3w4##c.energystoresw1w3mean_C c.timew3w4##c.invmillsmixed if sample8==1 & sex==0 || HNDID: timew3w4

//WHITES//
xtmixed bpdia c.timew3w4##c.food_price_BALTfinW1W3mean_C c.timew3w4##c.Agew1_C c.timew3w4##c.Agew3_C
c.timew3w4##sex c.timew3w4##race c.timew3w4##pir c.timew3w4##edubr c.timew3w4##employed c.timew3w4##wrattbr
c.timew3w4##smoke c.timew3w4##currdrugs c.timew3w4##c.bmi_C c.timew3w4##SRHbr c.timew3w4##c.kcalw1w3mean_C
c.timew3w4##c.energystoresw1w3mean_C c.timew3w4##c.invmillsmixed if sample8==1 & race==0 || HNDID: timew3w4

//AA//
xtmixed bpdia c.timew3w4##c.food_price_BALTfinW1W3mean_C c.timew3w4##c.Agew1_C c.timew3w4##c.Agew3_C
c.timew3w4##sex c.timew3w4##race c.timew3w4##pir c.timew3w4##edubr c.timew3w4##employed c.timew3w4##wrattbr
c.timew3w4##smoke c.timew3w4##currdrugs c.timew3w4##c.bmi_C c.timew3w4##SRHbr c.timew3w4##c.kcalw1w3mean_C
c.timew3w4##c.energystoresw1w3mean_C c.timew3w4##c.invmillsmixed if sample8==1 & race==1 || HNDID: timew3w4

//BELOW POVERTY//
xtmixed bpdia c.timew3w4##c.food_price_BALTfinW1W3mean_C c.timew3w4##c.Agew1_C c.timew3w4##c.Agew3_C
c.timew3w4##sex c.timew3w4##race c.timew3w4##pir c.timew3w4##edubr c.timew3w4##employed c.timew3w4##wrattbr
c.timew3w4##smoke c.timew3w4##currdrugs c.timew3w4##c.bmi_C c.timew3w4##SRHbr c.timew3w4##c.kcalw1w3mean_C
c.timew3w4##c.energystoresw1w3mean_C c.timew3w4##c.invmillsmixed if sample8==1 & pir==0 || HNDID: timew3w4

//ABOVE POVERTY//
xtmixed bpdia c.timew3w4##c.food_price_BALTfinW1W3mean_C c.timew3w4##c.Agew1_C c.timew3w4##c.Agew3_C
c.timew3w4##sex c.timew3w4##race c.timew3w4##pir c.timew3w4##edubr c.timew3w4##employed c.timew3w4##wrattbr
c.timew3w4##smoke c.timew3w4##currdrugs c.timew3w4##c.bmi_C c.timew3w4##SRHbr c.timew3w4##c.kcalw1w3mean_C
c.timew3w4##c.energystoresw1w3mean_C c.timew3w4##c.invmillsmixed if sample8==1 & pir==1 || HNDID: timew3w4


/////////////////////HEART RATE///////////////////

//TOTAL SAMPLE//
xtmixed hr c.timew3w4##c.food_price_BALTfinW1W3mean_C c.timew3w4##c.Agew1_C c.timew3w4##c.Agew3_C
c.timew3w4##sex c.timew3w4##race c.timew3w4##pir c.timew3w4##edubr c.timew3w4##employed c.timew3w4##wrattbr
c.timew3w4##smoke c.timew3w4##currdrugs c.timew3w4##c.bmi_C c.timew3w4##SRHbr c.timew3w4##c.kcalw1w3mean_C
c.timew3w4##c.energystoresw1w3mean_C c.timew3w4##c.invmillsmixed if sample8==1 || HNDID: timew3w4


//MEN//
xtmixed hr c.timew3w4##c.food_price_BALTfinW1W3mean_C c.timew3w4##c.Agew1_C c.timew3w4##c.Agew3_C
c.timew3w4##sex c.timew3w4##race c.timew3w4##pir c.timew3w4##edubr c.timew3w4##employed c.timew3w4##wrattbr
```

```
c.timew3w4##smoke c.timew3w4##currdrugs c.timew3w4##c.bmi_C c.timew3w4##SRHbr c.timew3w4##c.kcalw1w3mean_C
c.timew3w4##c.energystoresw1w3mean_C c.timew3w4##c.invmillsmixed if sample8==1 & sex==1 || HNDID: timew3w4

//WOMEN//
xtmixed hr c.timew3w4##c.food_price_BALTfinW1W3mean_C c.timew3w4##c.Agew1_C c.timew3w4##c.Agew3_C
c.timew3w4##sex c.timew3w4##race c.timew3w4##pir c.timew3w4##edubr c.timew3w4##employed c.timew3w4##wrattbr
c.timew3w4##smoke c.timew3w4##currdrugs c.timew3w4##c.bmi_C c.timew3w4##SRHbr c.timew3w4##c.kcalw1w3mean_C
c.timew3w4##c.energystoresw1w3mean_C c.timew3w4##c.invmillsmixed if sample8==1 & sex==0 || HNDID: timew3w4

//WHITES//
xtmixed hr c.timew3w4##c.food_price_BALTfinW1W3mean_C c.timew3w4##c.Agew1_C c.timew3w4##c.Agew3_C
c.timew3w4##sex c.timew3w4##race c.timew3w4##pir c.timew3w4##edubr c.timew3w4##employed c.timew3w4##wrattbr
c.timew3w4##smoke c.timew3w4##currdrugs c.timew3w4##c.bmi_C c.timew3w4##SRHbr c.timew3w4##c.kcalw1w3mean_C
c.timew3w4##c.energystoresw1w3mean_C c.timew3w4##c.invmillsmixed if sample8==1 & race==0 || HNDID: timew3w4

//AA//
xtmixed hr c.timew3w4##c.food_price_BALTfinW1W3mean_C c.timew3w4##c.Agew1_C c.timew3w4##c.Agew3_C
c.timew3w4##sex c.timew3w4##race c.timew3w4##pir c.timew3w4##edubr c.timew3w4##employed c.timew3w4##wrattbr
c.timew3w4##smoke c.timew3w4##currdrugs c.timew3w4##c.bmi_C c.timew3w4##SRHbr c.timew3w4##c.kcalw1w3mean_C
c.timew3w4##c.energystoresw1w3mean_C c.timew3w4##c.invmillsmixed if sample8==1 & race==1 || HNDID: timew3w4

//BELOW POVERTY//
xtmixed hr c.timew3w4##c.food_price_BALTfinW1W3mean_C c.timew3w4##c.Agew1_C c.timew3w4##c.Agew3_C
c.timew3w4##sex c.timew3w4##race c.timew3w4##pir c.timew3w4##edubr c.timew3w4##employed c.timew3w4##wrattbr
c.timew3w4##smoke c.timew3w4##currdrugs c.timew3w4##c.bmi_C c.timew3w4##SRHbr c.timew3w4##c.kcalw1w3mean_C
c.timew3w4##c.energystoresw1w3mean_C c.timew3w4##c.invmillsmixed if sample8==1 & pir==0 || HNDID: timew3w4

//ABOVE POVERTY//
xtmixed hr c.timew3w4##c.food_price_BALTfinW1W3mean_C c.timew3w4##c.Agew1_C c.timew3w4##c.Agew3_C
c.timew3w4##sex c.timew3w4##race c.timew3w4##pir c.timew3w4##edubr c.timew3w4##employed c.timew3w4##wrattbr
c.timew3w4##smoke c.timew3w4##currdrugs c.timew3w4##c.bmi_C c.timew3w4##SRHbr c.timew3w4##c.kcalw1w3mean_C
c.timew3w4##c.energystoresw1w3mean_C c.timew3w4##c.invmillsmixed if sample8==1 & pir==1 || HNDID: timew3w4


save, replace

///////////////DASH DIET ALONE: MODEL 3//////////////////////

//TOTAL SAMPLE//
xtmixed allostatic_prop c.timew3w4##c.DASH_scorew1w3mean_C c.timew3w4##c.Agew1_C c.timew3w4##c.Agew3_C
c.timew3w4##sex c.timew3w4##race c.timew3w4##pir c.timew3w4##edubr c.timew3w4##employed c.timew3w4##wrattbr
c.timew3w4##smoke c.timew3w4##currdrugs c.timew3w4##c.bmi_C c.timew3w4##SRHbr c.timew3w4##c.kcalw1w3mean_C
c.timew3w4##c.energystoresw1w3mean_C c.timew3w4##c.invmillsmixed if sample8==1 || HNDID: timew3w4
```

//MEN//

xtmixed allostatic_prop c.timew3w4##c.DASH_scorew1w3mean_C c.timew3w4##c.Agew1_C c.timew3w4##c.Agew3_C c.timew3w4##sex c.timew3w4##race c.timew3w4##pir c.timew3w4##edubr c.timew3w4##employed c.timew3w4##wrattbr c.timew3w4##smoke c.timew3w4##currdrugs c.timew3w4##c.bmi_C c.timew3w4##SRHbr c.timew3w4##c.kcalw1w3mean_C c.timew3w4##c.energystoresw1w3mean_C c.timew3w4##c.invmillsmixed if sample8==1 & sex==1 || HNDID: timew3w4

//WOMEN//

xtmixed allostatic_prop c.timew3w4##c.DASH_scorew1w3mean_C c.timew3w4##c.Agew1_C c.timew3w4##c.Agew3_C c.timew3w4##sex c.timew3w4##race c.timew3w4##pir c.timew3w4##edubr c.timew3w4##employed c.timew3w4##wrattbr c.timew3w4##smoke c.timew3w4##currdrugs c.timew3w4##c.bmi_C c.timew3w4##SRHbr c.timew3w4##c.kcalw1w3mean_C c.timew3w4##c.energystoresw1w3mean_C c.timew3w4##c.invmillsmixed if sample8==1 & sex==0 || HNDID: timew3w4

//WHITES//

xtmixed allostatic_prop c.timew3w4##c.DASH_scorew1w3mean_C c.timew3w4##c.Agew1_C c.timew3w4##c.Agew3_C c.timew3w4##sex c.timew3w4##race c.timew3w4##pir c.timew3w4##edubr c.timew3w4##employed c.timew3w4##wrattbr c.timew3w4##smoke c.timew3w4##currdrugs c.timew3w4##c.bmi_C c.timew3w4##SRHbr c.timew3w4##c.kcalw1w3mean_C c.timew3w4##c.energystoresw1w3mean_C c.timew3w4##c.invmillsmixed if sample8==1 & race==0 || HNDID: timew3w4

//AA//

xtmixed allostatic_prop c.timew3w4##c.DASH_scorew1w3mean_C c.timew3w4##c.Agew1_C c.timew3w4##c.Agew3_C c.timew3w4##sex c.timew3w4##race c.timew3w4##pir c.timew3w4##edubr c.timew3w4##employed c.timew3w4##wrattbr c.timew3w4##smoke c.timew3w4##currdrugs c.timew3w4##c.bmi_C c.timew3w4##SRHbr c.timew3w4##c.kcalw1w3mean_C c.timew3w4##c.energystoresw1w3mean_C c.timew3w4##c.invmillsmixed if sample8==1 & race==1 || HNDID: timew3w4

//BELOW POVERTY//

xtmixed allostatic_prop c.timew3w4##c.DASH_scorew1w3mean_C c.timew3w4##c.Agew1_C c.timew3w4##c.Agew3_C c.timew3w4##sex c.timew3w4##race c.timew3w4##pir c.timew3w4##edubr c.timew3w4##employed c.timew3w4##wrattbr c.timew3w4##smoke c.timew3w4##currdrugs c.timew3w4##c.bmi_C c.timew3w4##SRHbr c.timew3w4##c.kcalw1w3mean_C c.timew3w4##c.energystoresw1w3mean_C c.timew3w4##c.invmillsmixed if sample8==1 & pir==0 || HNDID: timew3w4

//ABOVE POVERTY//

xtmixed allostatic_prop c.timew3w4##c.DASH_scorew1w3mean_C c.timew3w4##c.Agew1_C c.timew3w4##c.Agew3_C c.timew3w4##sex c.timew3w4##race c.timew3w4##pir c.timew3w4##edubr c.timew3w4##employed c.timew3w4##wrattbr c.timew3w4##smoke c.timew3w4##currdrugs c.timew3w4##c.bmi_C c.timew3w4##SRHbr c.timew3w4##c.kcalw1w3mean_C c.timew3w4##c.energystoresw1w3mean_C c.timew3w4##c.invmillsmixed if sample8==1 & pir==1 || HNDID: timew3w4

Components of Allostatic load

//albumin crp chol hdl hgba1c whr bpsys bpdia hr//

****Albumin***********

//TOTAL SAMPLE//

xtmixed albumin c.timew3w4##c.DASH_scorew1w3mean_C c.timew3w4##c.Agew1_C c.timew3w4##c.Agew3_C c.timew3w4##sex c.timew3w4##race c.timew3w4##pir c.timew3w4##edubr c.timew3w4##employed c.timew3w4##wrattbr

c.timew3w4##smoke c.timew3w4##currdrugs c.timew3w4##c.bmi_C c.timew3w4##SRHbr c.timew3w4##c.kcalw1w3mean_C c.timew3w4##c.energystoresw1w3mean_C c.timew3w4##c.invmillsmixed if sample8==1 || HNDID: timew3w4

//MEN//

xtmixed albumin c.timew3w4##c.DASH_scorew1w3mean_C c.timew3w4##c.Agew1_C c.timew3w4##c.Agew3_C c.timew3w4##sex c.timew3w4##race c.timew3w4##pir c.timew3w4##edubr c.timew3w4##employed c.timew3w4##wrattbr c.timew3w4##smoke c.timew3w4##currdrugs c.timew3w4##c.bmi_C c.timew3w4##SRHbr c.timew3w4##c.kcalw1w3mean_C c.timew3w4##c.energystoresw1w3mean_C c.timew3w4##c.invmillsmixed if sample8==1 & sex==1 || HNDID: timew3w4

//WOMEN//

xtmixed albumin c.timew3w4##c.DASH_scorew1w3mean_C c.timew3w4##c.Agew1_C c.timew3w4##c.Agew3_C c.timew3w4##sex c.timew3w4##race c.timew3w4##pir c.timew3w4##edubr c.timew3w4##employed c.timew3w4##wrattbr c.timew3w4##smoke c.timew3w4##currdrugs c.timew3w4##c.bmi_C c.timew3w4##SRHbr c.timew3w4##c.kcalw1w3mean_C c.timew3w4##c.energystoresw1w3mean_C c.timew3w4##c.invmillsmixed if sample8==1 & sex==0 || HNDID: timew3w4

//WHITES//

xtmixed albumin c.timew3w4##c.DASH_scorew1w3mean_C c.timew3w4##c.Agew1_C c.timew3w4##c.Agew3_C c.timew3w4##sex c.timew3w4##race c.timew3w4##pir c.timew3w4##edubr c.timew3w4##employed c.timew3w4##wrattbr c.timew3w4##smoke c.timew3w4##currdrugs c.timew3w4##c.bmi_C c.timew3w4##SRHbr c.timew3w4##c.kcalw1w3mean_C c.timew3w4##c.energystoresw1w3mean_C c.timew3w4##c.invmillsmixed if sample8==1 & race==0 || HNDID: timew3w4

//AA//

xtmixed albumin c.timew3w4##c.DASH_scorew1w3mean_C c.timew3w4##c.Agew1_C c.timew3w4##c.Agew3_C c.timew3w4##sex c.timew3w4##race c.timew3w4##pir c.timew3w4##edubr c.timew3w4##employed c.timew3w4##wrattbr c.timew3w4##smoke c.timew3w4##currdrugs c.timew3w4##c.bmi_C c.timew3w4##SRHbr c.timew3w4##c.kcalw1w3mean_C c.timew3w4##c.energystoresw1w3mean_C c.timew3w4##c.invmillsmixed if sample8==1 & race==1 || HNDID: timew3w4

//BELOW POVERTY//

xtmixed albumin c.timew3w4##c.DASH_scorew1w3mean_C c.timew3w4##c.Agew1_C c.timew3w4##c.Agew3_C c.timew3w4##sex c.timew3w4##race c.timew3w4##pir c.timew3w4##edubr c.timew3w4##employed c.timew3w4##wrattbr c.timew3w4##smoke c.timew3w4##currdrugs c.timew3w4##c.bmi_C c.timew3w4##SRHbr c.timew3w4##c.kcalw1w3mean_C c.timew3w4##c.energystoresw1w3mean_C c.timew3w4##c.invmillsmixed if sample8==1 & pir==0 || HNDID: timew3w4

//ABOVE POVERTY//

xtmixed albumin c.timew3w4##c.DASH_scorew1w3mean_C c.timew3w4##c.Agew1_C c.timew3w4##c.Agew3_C c.timew3w4##sex c.timew3w4##race c.timew3w4##pir c.timew3w4##edubr c.timew3w4##employed c.timew3w4##wrattbr c.timew3w4##smoke c.timew3w4##currdrugs c.timew3w4##c.bmi_C c.timew3w4##SRHbr c.timew3w4##c.kcalw1w3mean_C c.timew3w4##c.energystoresw1w3mean_C c.timew3w4##c.invmillsmixed if sample8==1 & pir==1 || HNDID: timew3w4

*****C-reactive protein*******

//TOTAL SAMPLE//

```
xtmixed crp c.timew3w4##c.DASH_scorew1w3mean_C c.timew3w4##c.Agew1_C c.timew3w4##c.Agew3_C c.timew3w4##sex c.timew3w4##race c.timew3w4##pir c.timew3w4##edubr c.timew3w4##employed c.timew3w4##wrattbr c.timew3w4##smoke c.timew3w4##currdrugs c.timew3w4##c.bmi_C c.timew3w4##SRHbr c.timew3w4##c.kcalw1w3mean_C c.timew3w4##c.energystoresw1w3mean_C c.timew3w4##c.invmillsmixed if sample8==1 || HNDID: timew3w4


//MEN//
xtmixed crp c.timew3w4##c.DASH_scorew1w3mean_C c.timew3w4##c.Agew1_C c.timew3w4##c.Agew3_C c.timew3w4##sex c.timew3w4##race c.timew3w4##pir c.timew3w4##edubr c.timew3w4##employed c.timew3w4##wrattbr c.timew3w4##smoke c.timew3w4##currdrugs c.timew3w4##c.bmi_C c.timew3w4##SRHbr c.timew3w4##c.kcalw1w3mean_C c.timew3w4##c.energystoresw1w3mean_C c.timew3w4##c.invmillsmixed if sample8==1 & sex==1 || HNDID: timew3w4

//WOMEN//
xtmixed crp c.timew3w4##c.DASH_scorew1w3mean_C c.timew3w4##c.Agew1_C c.timew3w4##c.Agew3_C c.timew3w4##sex c.timew3w4##race c.timew3w4##pir c.timew3w4##edubr c.timew3w4##employed c.timew3w4##wrattbr c.timew3w4##smoke c.timew3w4##currdrugs c.timew3w4##c.bmi_C c.timew3w4##SRHbr c.timew3w4##c.kcalw1w3mean_C c.timew3w4##c.energystoresw1w3mean_C c.timew3w4##c.invmillsmixed if sample8==1 & sex==0 || HNDID: timew3w4

//WHITES//
xtmixed crp c.timew3w4##c.DASH_scorew1w3mean_C c.timew3w4##c.Agew1_C c.timew3w4##c.Agew3_C c.timew3w4##sex c.timew3w4##race c.timew3w4##pir c.timew3w4##edubr c.timew3w4##employed c.timew3w4##wrattbr c.timew3w4##smoke c.timew3w4##currdrugs c.timew3w4##c.bmi_C c.timew3w4##SRHbr c.timew3w4##c.kcalw1w3mean_C c.timew3w4##c.energystoresw1w3mean_C c.timew3w4##c.invmillsmixed if sample8==1 & race==0 || HNDID: timew3w4

//AA//
xtmixed crp c.timew3w4##c.DASH_scorew1w3mean_C c.timew3w4##c.Agew1_C c.timew3w4##c.Agew3_C c.timew3w4##sex c.timew3w4##race c.timew3w4##pir c.timew3w4##edubr c.timew3w4##employed c.timew3w4##wrattbr c.timew3w4##smoke c.timew3w4##currdrugs c.timew3w4##c.bmi_C c.timew3w4##SRHbr c.timew3w4##c.kcalw1w3mean_C c.timew3w4##c.energystoresw1w3mean_C c.timew3w4##c.invmillsmixed if sample8==1 & race==1 || HNDID: timew3w4

//BELOW POVERTY//
xtmixed crp c.timew3w4##c.DASH_scorew1w3mean_C c.timew3w4##c.Agew1_C c.timew3w4##c.Agew3_C c.timew3w4##sex c.timew3w4##race c.timew3w4##pir c.timew3w4##edubr c.timew3w4##employed c.timew3w4##wrattbr c.timew3w4##smoke c.timew3w4##currdrugs c.timew3w4##c.bmi_C c.timew3w4##SRHbr c.timew3w4##c.kcalw1w3mean_C c.timew3w4##c.energystoresw1w3mean_C c.timew3w4##c.invmillsmixed if sample8==1 & pir==0 || HNDID: timew3w4

//ABOVE POVERTY//
xtmixed crp c.timew3w4##c.DASH_scorew1w3mean_C c.timew3w4##c.Agew1_C c.timew3w4##c.Agew3_C c.timew3w4##sex c.timew3w4##race c.timew3w4##pir c.timew3w4##edubr c.timew3w4##employed c.timew3w4##wrattbr c.timew3w4##smoke c.timew3w4##currdrugs c.timew3w4##c.bmi_C c.timew3w4##SRHbr c.timew3w4##c.kcalw1w3mean_C c.timew3w4##c.energystoresw1w3mean_C c.timew3w4##c.invmillsmixed if sample8==1 & pir==1 || HNDID: timew3w4


**********Cholesterol**************
```

```
//TOTAL SAMPLE//
xtmixed chol c.timew3w4##c.DASH_scorew1w3mean_C c.timew3w4##c.Agew1_C c.timew3w4##c.Agew3_C
c.timew3w4##sex c.timew3w4##race c.timew3w4##pir c.timew3w4##edubr c.timew3w4##employed c.timew3w4##wrattbr
c.timew3w4##smoke c.timew3w4##currdrugs c.timew3w4##c.bmi_C c.timew3w4##SRHbr c.timew3w4##c.kcalw1w3mean_C
c.timew3w4##c.energystoresw1w3mean_C c.timew3w4##c.invmillsmixed if sample8==1 || HNDID: timew3w4

//MEN//
xtmixed chol c.timew3w4##c.DASH_scorew1w3mean_C c.timew3w4##c.Agew1_C c.timew3w4##c.Agew3_C
c.timew3w4##sex c.timew3w4##race c.timew3w4##pir c.timew3w4##edubr c.timew3w4##employed c.timew3w4##wrattbr
c.timew3w4##smoke c.timew3w4##currdrugs c.timew3w4##c.bmi_C c.timew3w4##SRHbr c.timew3w4##c.kcalw1w3mean_C
c.timew3w4##c.energystoresw1w3mean_C c.timew3w4##c.invmillsmixed if sample8==1 & sex==1 || HNDID: timew3w4

//WOMEN//
xtmixed chol c.timew3w4##c.DASH_scorew1w3mean_C c.timew3w4##c.Agew1_C c.timew3w4##c.Agew3_C
c.timew3w4##sex c.timew3w4##race c.timew3w4##pir c.timew3w4##edubr c.timew3w4##employed c.timew3w4##wrattbr
c.timew3w4##smoke c.timew3w4##currdrugs c.timew3w4##c.bmi_C c.timew3w4##SRHbr c.timew3w4##c.kcalw1w3mean_C
c.timew3w4##c.energystoresw1w3mean_C c.timew3w4##c.invmillsmixed if sample8==1 & sex==0 || HNDID: timew3w4

//WHITES//
xtmixed chol c.timew3w4##c.DASH_scorew1w3mean_C c.timew3w4##c.Agew1_C c.timew3w4##c.Agew3_C
c.timew3w4##sex c.timew3w4##race c.timew3w4##pir c.timew3w4##edubr c.timew3w4##employed c.timew3w4##wrattbr
c.timew3w4##smoke c.timew3w4##currdrugs c.timew3w4##c.bmi_C c.timew3w4##SRHbr c.timew3w4##c.kcalw1w3mean_C
c.timew3w4##c.energystoresw1w3mean_C c.timew3w4##c.invmillsmixed if sample8==1 & race==0 || HNDID: timew3w4

//AA//
xtmixed chol c.timew3w4##c.DASH_scorew1w3mean_C c.timew3w4##c.Agew1_C c.timew3w4##c.Agew3_C
c.timew3w4##sex c.timew3w4##race c.timew3w4##pir c.timew3w4##edubr c.timew3w4##employed c.timew3w4##wrattbr
c.timew3w4##smoke c.timew3w4##currdrugs c.timew3w4##c.bmi_C c.timew3w4##SRHbr c.timew3w4##c.kcalw1w3mean_C
c.timew3w4##c.energystoresw1w3mean_C c.timew3w4##c.invmillsmixed if sample8==1 & race==1 || HNDID: timew3w4

//BELOW POVERTY//
xtmixed chol c.timew3w4##c.DASH_scorew1w3mean_C c.timew3w4##c.Agew1_C c.timew3w4##c.Agew3_C
c.timew3w4##sex c.timew3w4##race c.timew3w4##pir c.timew3w4##edubr c.timew3w4##employed c.timew3w4##wrattbr
c.timew3w4##smoke c.timew3w4##currdrugs c.timew3w4##c.bmi_C c.timew3w4##SRHbr c.timew3w4##c.kcalw1w3mean_C
c.timew3w4##c.energystoresw1w3mean_C c.timew3w4##c.invmillsmixed if sample8==1 & pir==0 || HNDID: timew3w4

//ABOVE POVERTY//
xtmixed chol c.timew3w4##c.DASH_scorew1w3mean_C c.timew3w4##c.Agew1_C c.timew3w4##c.Agew3_C
c.timew3w4##sex c.timew3w4##race c.timew3w4##pir c.timew3w4##edubr c.timew3w4##employed c.timew3w4##wrattbr
c.timew3w4##smoke c.timew3w4##currdrugs c.timew3w4##c.bmi_C c.timew3w4##SRHbr c.timew3w4##c.kcalw1w3mean_C
c.timew3w4##c.energystoresw1w3mean_C c.timew3w4##c.invmillsmixed if sample8==1 & pir==1 || HNDID: timew3w4
```

***************HDL-C*******************

//TOTAL SAMPLE//

```
xtmixed hdl c.timew3w4##c.DASH_scorew1w3mean_C c.timew3w4##c.Agew1_C c.timew3w4##c.Agew3_C c.timew3w4##sex c.timew3w4##race c.timew3w4##pir c.timew3w4##edubr c.timew3w4##employed c.timew3w4##wrattbr c.timew3w4##smoke c.timew3w4##currdrugs c.timew3w4##c.bmi_C c.timew3w4##SRHbr c.timew3w4##c.kcalw1w3mean_C c.timew3w4##c.energystoresw1w3mean_C c.timew3w4##c.invmillsmixed if sample8==1 || HNDID: timew3w4
```

//MEN//

```
xtmixed hdl c.timew3w4##c.DASH_scorew1w3mean_C c.timew3w4##c.Agew1_C c.timew3w4##c.Agew3_C c.timew3w4##sex c.timew3w4##race c.timew3w4##pir c.timew3w4##edubr c.timew3w4##employed c.timew3w4##wrattbr c.timew3w4##smoke c.timew3w4##currdrugs c.timew3w4##c.bmi_C c.timew3w4##SRHbr c.timew3w4##c.kcalw1w3mean_C c.timew3w4##c.energystoresw1w3mean_C c.timew3w4##c.invmillsmixed if sample8==1 & sex==1 || HNDID: timew3w4
```

//WOMEN//

```
xtmixed hdl c.timew3w4##c.DASH_scorew1w3mean_C c.timew3w4##c.Agew1_C c.timew3w4##c.Agew3_C c.timew3w4##sex c.timew3w4##race c.timew3w4##pir c.timew3w4##edubr c.timew3w4##employed c.timew3w4##wrattbr c.timew3w4##smoke c.timew3w4##currdrugs c.timew3w4##c.bmi_C c.timew3w4##SRHbr c.timew3w4##c.kcalw1w3mean_C c.timew3w4##c.energystoresw1w3mean_C c.timew3w4##c.invmillsmixed if sample8==1 & sex==0 || HNDID: timew3w4
```

//WHITES//

```
xtmixed hdl c.timew3w4##c.DASH_scorew1w3mean_C c.timew3w4##c.Agew1_C c.timew3w4##c.Agew3_C c.timew3w4##sex c.timew3w4##race c.timew3w4##pir c.timew3w4##edubr c.timew3w4##employed c.timew3w4##wrattbr c.timew3w4##smoke c.timew3w4##currdrugs c.timew3w4##c.bmi_C c.timew3w4##SRHbr c.timew3w4##c.kcalw1w3mean_C c.timew3w4##c.energystoresw1w3mean_C c.timew3w4##c.invmillsmixed if sample8==1 & race==0 || HNDID: timew3w4
```

//AA//

```
xtmixed hdl c.timew3w4##c.DASH_scorew1w3mean_C c.timew3w4##c.Agew1_C c.timew3w4##c.Agew3_C c.timew3w4##sex c.timew3w4##race c.timew3w4##pir c.timew3w4##edubr c.timew3w4##employed c.timew3w4##wrattbr c.timew3w4##smoke c.timew3w4##currdrugs c.timew3w4##c.bmi_C c.timew3w4##SRHbr c.timew3w4##c.kcalw1w3mean_C c.timew3w4##c.energystoresw1w3mean_C c.timew3w4##c.invmillsmixed if sample8==1 & race==1 || HNDID: timew3w4
```

//BELOW POVERTY//

```
xtmixed hdl c.timew3w4##c.DASH_scorew1w3mean_C c.timew3w4##c.Agew1_C c.timew3w4##c.Agew3_C c.timew3w4##sex c.timew3w4##race c.timew3w4##pir c.timew3w4##edubr c.timew3w4##employed c.timew3w4##wrattbr c.timew3w4##smoke c.timew3w4##currdrugs c.timew3w4##c.bmi_C c.timew3w4##SRHbr c.timew3w4##c.kcalw1w3mean_C c.timew3w4##c.energystoresw1w3mean_C c.timew3w4##c.invmillsmixed if sample8==1 & pir==0 || HNDID: timew3w4
```

//ABOVE POVERTY//

```
xtmixed hdl c.timew3w4##c.DASH_scorew1w3mean_C c.timew3w4##c.Agew1_C c.timew3w4##c.Agew3_C c.timew3w4##sex c.timew3w4##race c.timew3w4##pir c.timew3w4##edubr c.timew3w4##employed c.timew3w4##wrattbr
```

```
c.timew3w4##smoke c.timew3w4##currdrugs c.timew3w4##c.bmi_C c.timew3w4##SRHbr c.timew3w4##c.kcalw1w3mean_C
c.timew3w4##c.energystoresw1w3mean_C c.timew3w4##c.invmillsmixed if sample8==1 & pir==1 || HNDID: timew3w4

//////////////GLYCATED HEMOGLOBIN//////////////

//TOTAL SAMPLE//
xtmixed hgba1c c.timew3w4##c.DASH_scorew1w3mean_C c.timew3w4##c.Agew1_C c.timew3w4##c.Agew3_C
c.timew3w4##sex c.timew3w4##race c.timew3w4##pir c.timew3w4##edubr c.timew3w4##employed c.timew3w4##wrattbr
c.timew3w4##smoke c.timew3w4##currdrugs c.timew3w4##c.bmi_C c.timew3w4##SRHbr c.timew3w4##c.kcalw1w3mean_C
c.timew3w4##c.energystoresw1w3mean_C c.timew3w4##c.invmillsmixed if sample8==1 || HNDID: timew3w4

//MEN//
xtmixed hgba1c c.timew3w4##c.DASH_scorew1w3mean_C c.timew3w4##c.Agew1_C c.timew3w4##c.Agew3_C
c.timew3w4##sex c.timew3w4##race c.timew3w4##pir c.timew3w4##edubr c.timew3w4##employed c.timew3w4##wrattbr
c.timew3w4##smoke c.timew3w4##currdrugs c.timew3w4##c.bmi_C c.timew3w4##SRHbr c.timew3w4##c.kcalw1w3mean_C
c.timew3w4##c.energystoresw1w3mean_C c.timew3w4##c.invmillsmixed if sample8==1 & sex==1 || HNDID: timew3w4

//WOMEN//
xtmixed hgba1c c.timew3w4##c.DASH_scorew1w3mean_C c.timew3w4##c.Agew1_C c.timew3w4##c.Agew3_C
c.timew3w4##sex c.timew3w4##race c.timew3w4##pir c.timew3w4##edubr c.timew3w4##employed c.timew3w4##wrattbr
c.timew3w4##smoke c.timew3w4##currdrugs c.timew3w4##c.bmi_C c.timew3w4##SRHbr c.timew3w4##c.kcalw1w3mean_C
c.timew3w4##c.energystoresw1w3mean_C c.timew3w4##c.invmillsmixed if sample8==1 & sex==0 || HNDID: timew3w4

//WHITES//
xtmixed hgba1c c.timew3w4##c.DASH_scorew1w3mean_C c.timew3w4##c.Agew1_C c.timew3w4##c.Agew3_C
c.timew3w4##sex c.timew3w4##race c.timew3w4##pir c.timew3w4##edubr c.timew3w4##employed c.timew3w4##wrattbr
c.timew3w4##smoke c.timew3w4##currdrugs c.timew3w4##c.bmi_C c.timew3w4##SRHbr c.timew3w4##c.kcalw1w3mean_C
c.timew3w4##c.energystoresw1w3mean_C c.timew3w4##c.invmillsmixed if sample8==1 & race==0 || HNDID: timew3w4

//AA//
xtmixed hgba1c c.timew3w4##c.DASH_scorew1w3mean_C c.timew3w4##c.Agew1_C c.timew3w4##c.Agew3_C
c.timew3w4##sex c.timew3w4##race c.timew3w4##pir c.timew3w4##edubr c.timew3w4##employed c.timew3w4##wrattbr
c.timew3w4##smoke c.timew3w4##currdrugs c.timew3w4##c.bmi_C c.timew3w4##SRHbr c.timew3w4##c.kcalw1w3mean_C
c.timew3w4##c.energystoresw1w3mean_C c.timew3w4##c.invmillsmixed if sample8==1 & race==1 || HNDID: timew3w4

//BELOW POVERTY//
xtmixed hgba1c c.timew3w4##c.DASH_scorew1w3mean_C c.timew3w4##c.Agew1_C c.timew3w4##c.Agew3_C
c.timew3w4##sex c.timew3w4##race c.timew3w4##pir c.timew3w4##edubr c.timew3w4##employed c.timew3w4##wrattbr
c.timew3w4##smoke c.timew3w4##currdrugs c.timew3w4##c.bmi_C c.timew3w4##SRHbr c.timew3w4##c.kcalw1w3mean_C
c.timew3w4##c.energystoresw1w3mean_C c.timew3w4##c.invmillsmixed if sample8==1 & pir==0 || HNDID: timew3w4
```

//ABOVE POVERTY//

xtmixed hgba1c c.timew3w4##c.DASH_scorew1w3mean_C c.timew3w4##c.Agew1_C c.timew3w4##c.Agew3_C c.timew3w4##sex c.timew3w4##race c.timew3w4##pir c.timew3w4##edubr c.timew3w4##employed c.timew3w4##wrattbr c.timew3w4##smoke c.timew3w4##currdrugs c.timew3w4##c.bmi_C c.timew3w4##SRHbr c.timew3w4##c.kcalw1w3mean_C c.timew3w4##c.energystoresw1w3mean_C c.timew3w4##c.invmillsmixed if sample8==1 & pir==1 || HNDID: timew3w4

////////////////WHR////////////////

//TOTAL SAMPLE//

xtmixed whr c.timew3w4##c.DASH_scorew1w3mean_C c.timew3w4##c.Agew1_C c.timew3w4##c.Agew3_C c.timew3w4##sex c.timew3w4##race c.timew3w4##pir c.timew3w4##edubr c.timew3w4##employed c.timew3w4##wrattbr c.timew3w4##smoke c.timew3w4##currdrugs c.timew3w4##c.bmi_C c.timew3w4##SRHbr c.timew3w4##c.kcalw1w3mean_C c.timew3w4##c.energystoresw1w3mean_C c.timew3w4##c.invmillsmixed if sample8==1 || HNDID: timew3w4

//MEN//

xtmixed whr c.timew3w4##c.DASH_scorew1w3mean_C c.timew3w4##c.Agew1_C c.timew3w4##c.Agew3_C c.timew3w4##sex c.timew3w4##race c.timew3w4##pir c.timew3w4##edubr c.timew3w4##employed c.timew3w4##wrattbr c.timew3w4##smoke c.timew3w4##currdrugs c.timew3w4##c.bmi_C c.timew3w4##SRHbr c.timew3w4##c.kcalw1w3mean_C c.timew3w4##c.energystoresw1w3mean_C c.timew3w4##c.invmillsmixed if sample8==1 & sex==1 || HNDID: timew3w4

//WOMEN//

xtmixed whr c.timew3w4##c.DASH_scorew1w3mean_C c.timew3w4##c.Agew1_C c.timew3w4##c.Agew3_C c.timew3w4##sex c.timew3w4##race c.timew3w4##pir c.timew3w4##edubr c.timew3w4##employed c.timew3w4##wrattbr c.timew3w4##smoke c.timew3w4##currdrugs c.timew3w4##c.bmi_C c.timew3w4##SRHbr c.timew3w4##c.kcalw1w3mean_C c.timew3w4##c.energystoresw1w3mean_C c.timew3w4##c.invmillsmixed if sample8==1 & sex==0 || HNDID: timew3w4

//WHITES//

xtmixed whr c.timew3w4##c.DASH_scorew1w3mean_C c.timew3w4##c.Agew1_C c.timew3w4##c.Agew3_C c.timew3w4##sex c.timew3w4##race c.timew3w4##pir c.timew3w4##edubr c.timew3w4##employed c.timew3w4##wrattbr c.timew3w4##smoke c.timew3w4##currdrugs c.timew3w4##c.bmi_C c.timew3w4##SRHbr c.timew3w4##c.kcalw1w3mean_C c.timew3w4##c.energystoresw1w3mean_C c.timew3w4##c.invmillsmixed if sample8==1 & race==0 || HNDID: timew3w4

//AA//

xtmixed whr c.timew3w4##c.DASH_scorew1w3mean_C c.timew3w4##c.Agew1_C c.timew3w4##c.Agew3_C c.timew3w4##sex c.timew3w4##race c.timew3w4##pir c.timew3w4##edubr c.timew3w4##employed c.timew3w4##wrattbr c.timew3w4##smoke c.timew3w4##currdrugs c.timew3w4##c.bmi_C c.timew3w4##SRHbr c.timew3w4##c.kcalw1w3mean_C c.timew3w4##c.energystoresw1w3mean_C c.timew3w4##c.invmillsmixed if sample8==1 & race==1 || HNDID: timew3w4

//BELOW POVERTY//

xtmixed whr c.timew3w4##c.DASH_scorew1w3mean_C c.timew3w4##c.Agew1_C c.timew3w4##c.Agew3_C c.timew3w4##sex c.timew3w4##race c.timew3w4##pir c.timew3w4##edubr c.timew3w4##employed c.timew3w4##wrattbr

c.timew3w4##smoke c.timew3w4##currdrugs c.timew3w4##c.bmi_C c.timew3w4##SRHbr c.timew3w4##c.kcalw1w3mean_C c.timew3w4##c.energystoresw1w3mean_C c.timew3w4##c.invmillsmixed if sample8==1 & pir==0 || HNDID: timew3w4

//ABOVE POVERTY//

xtmixed whr c.timew3w4##c.DASH_scorew1w3mean_C c.timew3w4##c.Agew1_C c.timew3w4##c.Agew3_C c.timew3w4##sex c.timew3w4##race c.timew3w4##pir c.timew3w4##edubr c.timew3w4##employed c.timew3w4##wrattbr c.timew3w4##smoke c.timew3w4##currdrugs c.timew3w4##c.bmi_C c.timew3w4##SRHbr c.timew3w4##c.kcalw1w3mean_C c.timew3w4##c.energystoresw1w3mean_C c.timew3w4##c.invmillsmixed if sample8==1 & pir==1 || HNDID: timew3w4

///////////////////////////SYSTOLIC BLOOD PRESSURE/////////////////////

//TOTAL SAMPLE//

xtmixed bpsys c.timew3w4##c.DASH_scorew1w3mean_C c.timew3w4##c.Agew1_C c.timew3w4##c.Agew3_C c.timew3w4##sex c.timew3w4##race c.timew3w4##pir c.timew3w4##edubr c.timew3w4##employed c.timew3w4##wrattbr c.timew3w4##smoke c.timew3w4##currdrugs c.timew3w4##c.bmi_C c.timew3w4##SRHbr c.timew3w4##c.kcalw1w3mean_C c.timew3w4##c.energystoresw1w3mean_C c.timew3w4##c.invmillsmixed if sample8==1 || HNDID: timew3w4

//MEN//

xtmixed bpsys c.timew3w4##c.DASH_scorew1w3mean_C c.timew3w4##c.Agew1_C c.timew3w4##c.Agew3_C c.timew3w4##sex c.timew3w4##race c.timew3w4##pir c.timew3w4##edubr c.timew3w4##employed c.timew3w4##wrattbr c.timew3w4##smoke c.timew3w4##currdrugs c.timew3w4##c.bmi_C c.timew3w4##SRHbr c.timew3w4##c.kcalw1w3mean_C c.timew3w4##c.energystoresw1w3mean_C c.timew3w4##c.invmillsmixed if sample8==1 & sex==1 || HNDID: timew3w4

//WOMEN//

xtmixed bpsys c.timew3w4##c.DASH_scorew1w3mean_C c.timew3w4##c.Agew1_C c.timew3w4##c.Agew3_C c.timew3w4##sex c.timew3w4##race c.timew3w4##pir c.timew3w4##edubr c.timew3w4##employed c.timew3w4##wrattbr c.timew3w4##smoke c.timew3w4##currdrugs c.timew3w4##c.bmi_C c.timew3w4##SRHbr c.timew3w4##c.kcalw1w3mean_C c.timew3w4##c.energystoresw1w3mean_C c.timew3w4##c.invmillsmixed if sample8==1 & sex==0 || HNDID: timew3w4

//WHITES//

xtmixed bpsys c.timew3w4##c.DASH_scorew1w3mean_C c.timew3w4##c.Agew1_C c.timew3w4##c.Agew3_C c.timew3w4##sex c.timew3w4##race c.timew3w4##pir c.timew3w4##edubr c.timew3w4##employed c.timew3w4##wrattbr c.timew3w4##smoke c.timew3w4##currdrugs c.timew3w4##c.bmi_C c.timew3w4##SRHbr c.timew3w4##c.kcalw1w3mean_C c.timew3w4##c.energystoresw1w3mean_C c.timew3w4##c.invmillsmixed if sample8==1 & race==0 || HNDID: timew3w4

//AA//

xtmixed bpsys c.timew3w4##c.DASH_scorew1w3mean_C c.timew3w4##c.Agew1_C c.timew3w4##c.Agew3_C c.timew3w4##sex c.timew3w4##race c.timew3w4##pir c.timew3w4##edubr c.timew3w4##employed c.timew3w4##wrattbr c.timew3w4##smoke c.timew3w4##currdrugs c.timew3w4##c.bmi_C c.timew3w4##SRHbr c.timew3w4##c.kcalw1w3mean_C c.timew3w4##c.energystoresw1w3mean_C c.timew3w4##c.invmillsmixed if sample8==1 & race==1 || HNDID: timew3w4

//BELOW POVERTY//

```
xtmixed bpsys c.timew3w4##c.DASH_scorew1w3mean_C c.timew3w4##c.Agew1_C c.timew3w4##c.Agew3_C
c.timew3w4##sex c.timew3w4##race c.timew3w4##pir c.timew3w4##edubr c.timew3w4##employed c.timew3w4##wrattbr
c.timew3w4##smoke c.timew3w4##currdrugs c.timew3w4##c.bmi_C c.timew3w4##SRHbr c.timew3w4##c.kcalw1w3mean_C
c.timew3w4##c.energystoresw1w3mean_C c.timew3w4##c.invmillsmixed if sample8==1 & pir==0 || HNDID: timew3w4

//ABOVE POVERTY//
xtmixed bpsys c.timew3w4##c.DASH_scorew1w3mean_C c.timew3w4##c.Agew1_C c.timew3w4##c.Agew3_C
c.timew3w4##sex c.timew3w4##race c.timew3w4##pir c.timew3w4##edubr c.timew3w4##employed c.timew3w4##wrattbr
c.timew3w4##smoke c.timew3w4##currdrugs c.timew3w4##c.bmi_C c.timew3w4##SRHbr c.timew3w4##c.kcalw1w3mean_C
c.timew3w4##c.energystoresw1w3mean_C c.timew3w4##c.invmillsmixed if sample8==1 & pir==1 || HNDID: timew3w4

///////////////////////////DIASTOLIC BLOOD PRESSURE//////////////

//TOTAL SAMPLE//
xtmixed bpdia c.timew3w4##c.DASH_scorew1w3mean_C c.timew3w4##c.Agew1_C c.timew3w4##c.Agew3_C
c.timew3w4##sex c.timew3w4##race c.timew3w4##pir c.timew3w4##edubr c.timew3w4##employed c.timew3w4##wrattbr
c.timew3w4##smoke c.timew3w4##currdrugs c.timew3w4##c.bmi_C c.timew3w4##SRHbr c.timew3w4##c.kcalw1w3mean_C
c.timew3w4##c.energystoresw1w3mean_C c.timew3w4##c.invmillsmixed if sample8==1 || HNDID: timew3w4


//MEN//
xtmixed bpdia c.timew3w4##c.DASH_scorew1w3mean_C c.timew3w4##c.Agew1_C c.timew3w4##c.Agew3_C
c.timew3w4##sex c.timew3w4##race c.timew3w4##pir c.timew3w4##edubr c.timew3w4##employed c.timew3w4##wrattbr
c.timew3w4##smoke c.timew3w4##currdrugs c.timew3w4##c.bmi_C c.timew3w4##SRHbr c.timew3w4##c.kcalw1w3mean_C
c.timew3w4##c.energystoresw1w3mean_C c.timew3w4##c.invmillsmixed if sample8==1 & sex==1 || HNDID: timew3w4

//WOMEN//
xtmixed bpdia c.timew3w4##c.DASH_scorew1w3mean_C c.timew3w4##c.Agew1_C c.timew3w4##c.Agew3_C
c.timew3w4##sex c.timew3w4##race c.timew3w4##pir c.timew3w4##edubr c.timew3w4##employed c.timew3w4##wrattbr
c.timew3w4##smoke c.timew3w4##currdrugs c.timew3w4##c.bmi_C c.timew3w4##SRHbr c.timew3w4##c.kcalw1w3mean_C
c.timew3w4##c.energystoresw1w3mean_C c.timew3w4##c.invmillsmixed if sample8==1 & sex==0 || HNDID: timew3w4

//WHITES//
xtmixed bpdia c.timew3w4##c.DASH_scorew1w3mean_C c.timew3w4##c.Agew1_C c.timew3w4##c.Agew3_C
c.timew3w4##sex c.timew3w4##race c.timew3w4##pir c.timew3w4##edubr c.timew3w4##employed c.timew3w4##wrattbr
c.timew3w4##smoke c.timew3w4##currdrugs c.timew3w4##c.bmi_C c.timew3w4##SRHbr c.timew3w4##c.kcalw1w3mean_C
c.timew3w4##c.energystoresw1w3mean_C c.timew3w4##c.invmillsmixed if sample8==1 & race==0 || HNDID: timew3w4

//AA//
xtmixed bpdia c.timew3w4##c.DASH_scorew1w3mean_C c.timew3w4##c.Agew1_C c.timew3w4##c.Agew3_C
c.timew3w4##sex c.timew3w4##race c.timew3w4##pir c.timew3w4##edubr c.timew3w4##employed c.timew3w4##wrattbr
c.timew3w4##smoke c.timew3w4##currdrugs c.timew3w4##c.bmi_C c.timew3w4##SRHbr c.timew3w4##c.kcalw1w3mean_C
c.timew3w4##c.energystoresw1w3mean_C c.timew3w4##c.invmillsmixed if sample8==1 & race==1 || HNDID: timew3w4
```

//BELOW POVERTY//

xtmixed bpdia c.timew3w4##c.DASH_scorew1w3mean_C c.timew3w4##c.Agew1_C c.timew3w4##c.Agew3_C c.timew3w4##sex c.timew3w4##race c.timew3w4##pir c.timew3w4##edubr c.timew3w4##employed c.timew3w4##wrattbr c.timew3w4##smoke c.timew3w4##currdrugs c.timew3w4##c.bmi_C c.timew3w4##SRHbr c.timew3w4##c.kcalw1w3mean_C c.timew3w4##c.energystoresw1w3mean_C c.timew3w4##c.invmillsmixed if sample8==1 & pir==0 || HNDID: timew3w4

//ABOVE POVERTY//

xtmixed bpdia c.timew3w4##c.DASH_scorew1w3mean_C c.timew3w4##c.Agew1_C c.timew3w4##c.Agew3_C c.timew3w4##sex c.timew3w4##race c.timew3w4##pir c.timew3w4##edubr c.timew3w4##employed c.timew3w4##wrattbr c.timew3w4##smoke c.timew3w4##currdrugs c.timew3w4##c.bmi_C c.timew3w4##SRHbr c.timew3w4##c.kcalw1w3mean_C c.timew3w4##c.energystoresw1w3mean_C c.timew3w4##c.invmillsmixed if sample8==1 & pir==1 || HNDID: timew3w4

/////////////////////HEART RATE///////////////////

//TOTAL SAMPLE//

xtmixed hr c.timew3w4##c.DASH_scorew1w3mean_C c.timew3w4##c.Agew1_C c.timew3w4##c.Agew3_C c.timew3w4##sex c.timew3w4##race c.timew3w4##pir c.timew3w4##edubr c.timew3w4##employed c.timew3w4##wrattbr c.timew3w4##smoke c.timew3w4##currdrugs c.timew3w4##c.bmi_C c.timew3w4##SRHbr c.timew3w4##c.kcalw1w3mean_C c.timew3w4##c.energystoresw1w3mean_C c.timew3w4##c.invmillsmixed if sample8==1 || HNDID: timew3w4

//MEN//

xtmixed hr c.timew3w4##c.DASH_scorew1w3mean_C c.timew3w4##c.Agew1_C c.timew3w4##c.Agew3_C c.timew3w4##sex c.timew3w4##race c.timew3w4##pir c.timew3w4##edubr c.timew3w4##employed c.timew3w4##wrattbr c.timew3w4##smoke c.timew3w4##currdrugs c.timew3w4##c.bmi_C c.timew3w4##SRHbr c.timew3w4##c.kcalw1w3mean_C c.timew3w4##c.energystoresw1w3mean_C c.timew3w4##c.invmillsmixed if sample8==1 & sex==1 || HNDID: timew3w4

//WOMEN//

xtmixed hr c.timew3w4##c.DASH_scorew1w3mean_C c.timew3w4##c.Agew1_C c.timew3w4##c.Agew3_C c.timew3w4##sex c.timew3w4##race c.timew3w4##pir c.timew3w4##edubr c.timew3w4##employed c.timew3w4##wrattbr c.timew3w4##smoke c.timew3w4##currdrugs c.timew3w4##c.bmi_C c.timew3w4##SRHbr c.timew3w4##c.kcalw1w3mean_C c.timew3w4##c.energystoresw1w3mean_C c.timew3w4##c.invmillsmixed if sample8==1 & sex==0 || HNDID: timew3w4

//WHITES//

xtmixed hr c.timew3w4##c.DASH_scorew1w3mean_C c.timew3w4##c.Agew1_C c.timew3w4##c.Agew3_C c.timew3w4##sex c.timew3w4##race c.timew3w4##pir c.timew3w4##edubr c.timew3w4##employed c.timew3w4##wrattbr c.timew3w4##smoke c.timew3w4##currdrugs c.timew3w4##c.bmi_C c.timew3w4##SRHbr c.timew3w4##c.kcalw1w3mean_C c.timew3w4##c.energystoresw1w3mean_C c.timew3w4##c.invmillsmixed if sample8==1 & race==0 || HNDID: timew3w4

//AA//

xtmixed hr c.timew3w4##c.DASH_scorew1w3mean_C c.timew3w4##c.Agew1_C c.timew3w4##c.Agew3_C c.timew3w4##sex c.timew3w4##race c.timew3w4##pir c.timew3w4##edubr c.timew3w4##employed c.timew3w4##wrattbr

c.timew3w4##smoke c.timew3w4##currdrugs c.timew3w4##c.bmi_C c.timew3w4##SRHbr c.timew3w4##c.kcalw1w3mean_C c.timew3w4##c.energystoresw1w3mean_C c.timew3w4##c.invmillsmixed if sample8==1 & race==1 || HNDID: timew3w4

//BELOW POVERTY//

xtmixed hr c.timew3w4##c.DASH_scorew1w3mean_C c.timew3w4##c.Agew1_C c.timew3w4##c.Agew3_C c.timew3w4##sex c.timew3w4##race c.timew3w4##pir c.timew3w4##edubr c.timew3w4##employed c.timew3w4##wrattbr c.timew3w4##smoke c.timew3w4##currdrugs c.timew3w4##c.bmi_C c.timew3w4##SRHbr c.timew3w4##c.kcalw1w3mean_C c.timew3w4##c.energystoresw1w3mean_C c.timew3w4##c.invmillsmixed if sample8==1 & pir==0 || HNDID: timew3w4

//ABOVE POVERTY//

xtmixed hr c.timew3w4##c.DASH_scorew1w3mean_C c.timew3w4##c.Agew1_C c.timew3w4##c.Agew3_C c.timew3w4##sex c.timew3w4##race c.timew3w4##pir c.timew3w4##edubr c.timew3w4##employed c.timew3w4##wrattbr c.timew3w4##smoke c.timew3w4##currdrugs c.timew3w4##c.bmi_C c.timew3w4##SRHbr c.timew3w4##c.kcalw1w3mean_C c.timew3w4##c.energystoresw1w3mean_C c.timew3w4##c.invmillsmixed if sample8==1 & pir==1 || HNDID: timew3w4

save, replace

TABLE 3, MODEL 1, INTERACTION WITH FOOD COST*

cd "G:\...\DATA"

use HANDLS_Allostaticload_dietfcfinal, clear

//ALLOSTATIC LOAD//

xtmixed allostatic_prop c.timew3w4##c.food_price_BALTfinW1W3mean_C##sex c.timew3w4##c.DASH_scorew1w3mean_C c.timew3w4##c.Agew1_C c.timew3w4##c.Agew3_C c.timew3w4##sex c.timew3w4##race c.timew3w4##pir c.timew3w4##edubr c.timew3w4##employed c.timew3w4##wrattbr c.timew3w4##smoke c.timew3w4##currdrugs c.timew3w4##c.bmi_C c.timew3w4##SRHbr c.timew3w4##c.kcalw1w3mean_C c.timew3w4##c.energystoresw1w3mean_C c.timew3w4##c.invmillsmixed if sample8==1 || HNDID: timew3w4

xtmixed allostatic_prop c.timew3w4##c.food_price_BALTfinW1W3mean_C##race c.timew3w4##c.DASH_scorew1w3mean_C c.timew3w4##c.Agew1_C c.timew3w4##c.Agew3_C c.timew3w4##sex c.timew3w4##race c.timew3w4##pir c.timew3w4##edubr c.timew3w4##employed c.timew3w4##wrattbr c.timew3w4##smoke c.timew3w4##currdrugs c.timew3w4##c.bmi_C c.timew3w4##SRHbr c.timew3w4##c.kcalw1w3mean_C c.timew3w4##c.energystoresw1w3mean_C c.timew3w4##c.invmillsmixed if sample8==1 || HNDID: timew3w4

xtmixed allostatic_prop c.timew3w4##c.food_price_BALTfinW1W3mean_C##pir c.timew3w4##c.DASH_scorew1w3mean_C c.timew3w4##c.Agew1_C c.timew3w4##c.Agew3_C c.timew3w4##sex c.timew3w4##race c.timew3w4##pir c.timew3w4##edubr c.timew3w4##employed c.timew3w4##wrattbr c.timew3w4##smoke c.timew3w4##currdrugs

c.timew3w4##c.bmi_C c.timew3w4##SRHbr c.timew3w4##c.kcalw1w3mean_C c.timew3w4##c.energystoresw1w3mean_C c.timew3w4##c.invmillsmixed if sample8==1 || HNDID: timew3w4

//ALB//

xtmixed albumin c.timew3w4##c.food_price_BALTfinW1W3mean_C##sex c.timew3w4##c.DASH_scorew1w3mean_C c.timew3w4##c.Agew1_C c.timew3w4##c.Agew3_C c.timew3w4##sex c.timew3w4##race c.timew3w4##pir c.timew3w4##edubr c.timew3w4##employed c.timew3w4##wrattbr c.timew3w4##smoke c.timew3w4##currdrugs c.timew3w4##c.bmi_C c.timew3w4##SRHbr c.timew3w4##c.kcalw1w3mean_C c.timew3w4##c.energystoresw1w3mean_C c.timew3w4##c.invmillsmixed if sample8==1 || HNDID: timew3w4

xtmixed albumin c.timew3w4##c.food_price_BALTfinW1W3mean_C##race c.timew3w4##c.DASH_scorew1w3mean_C c.timew3w4##c.Agew1_C c.timew3w4##c.Agew3_C c.timew3w4##sex c.timew3w4##race c.timew3w4##pir c.timew3w4##edubr c.timew3w4##employed c.timew3w4##wrattbr c.timew3w4##smoke c.timew3w4##currdrugs c.timew3w4##c.bmi_C c.timew3w4##SRHbr c.timew3w4##c.kcalw1w3mean_C c.timew3w4##c.energystoresw1w3mean_C c.timew3w4##c.invmillsmixed if sample8==1 || HNDID: timew3w4

xtmixed albumin c.timew3w4##c.food_price_BALTfinW1W3mean_C##pir c.timew3w4##c.DASH_scorew1w3mean_C c.timew3w4##c.Agew1_C c.timew3w4##c.Agew3_C c.timew3w4##sex c.timew3w4##race c.timew3w4##pir c.timew3w4##edubr c.timew3w4##employed c.timew3w4##wrattbr c.timew3w4##smoke c.timew3w4##currdrugs c.timew3w4##c.bmi_C c.timew3w4##SRHbr c.timew3w4##c.kcalw1w3mean_C c.timew3w4##c.energystoresw1w3mean_C c.timew3w4##c.invmillsmixed if sample8==1 || HNDID: timew3w4

//CRP//

xtmixed crp c.timew3w4##c.food_price_BALTfinW1W3mean_C##sex c.timew3w4##c.DASH_scorew1w3mean_C c.timew3w4##c.Agew1_C c.timew3w4##c.Agew3_C c.timew3w4##sex c.timew3w4##race c.timew3w4##pir c.timew3w4##edubr c.timew3w4##employed c.timew3w4##wrattbr c.timew3w4##smoke c.timew3w4##currdrugs c.timew3w4##c.bmi_C c.timew3w4##SRHbr c.timew3w4##c.kcalw1w3mean_C c.timew3w4##c.energystoresw1w3mean_C c.timew3w4##c.invmillsmixed if sample8==1 || HNDID: timew3w4

xtmixed crp c.timew3w4##c.food_price_BALTfinW1W3mean_C##race c.timew3w4##c.DASH_scorew1w3mean_C c.timew3w4##c.Agew1_C c.timew3w4##c.Agew3_C c.timew3w4##sex c.timew3w4##race c.timew3w4##pir c.timew3w4##edubr c.timew3w4##employed c.timew3w4##wrattbr c.timew3w4##smoke c.timew3w4##currdrugs c.timew3w4##c.bmi_C c.timew3w4##SRHbr c.timew3w4##c.kcalw1w3mean_C c.timew3w4##c.energystoresw1w3mean_C c.timew3w4##c.invmillsmixed if sample8==1 || HNDID: timew3w4

xtmixed crp c.timew3w4##c.food_price_BALTfinW1W3mean_C##pir c.timew3w4##c.DASH_scorew1w3mean_C c.timew3w4##c.Agew1_C c.timew3w4##c.Agew3_C c.timew3w4##sex c.timew3w4##race c.timew3w4##pir c.timew3w4##edubr c.timew3w4##employed c.timew3w4##wrattbr c.timew3w4##smoke c.timew3w4##currdrugs c.timew3w4##c.bmi_C c.timew3w4##SRHbr c.timew3w4##c.kcalw1w3mean_C c.timew3w4##c.energystoresw1w3mean_C c.timew3w4##c.invmillsmixed if sample8==1 || HNDID: timew3w4

//Cholesterol//

xtmixed chol c.timew3w4##c.food_price_BALTfinW1W3mean_C##sex c.timew3w4##c.DASH_scorew1w3mean_C c.timew3w4##c.Agew1_C c.timew3w4##c.Agew3_C c.timew3w4##sex c.timew3w4##race c.timew3w4##pir c.timew3w4##edubr c.timew3w4##employed c.timew3w4##wrattbr c.timew3w4##smoke c.timew3w4##currdrugs c.timew3w4##c.bmi_C c.timew3w4##SRHbr c.timew3w4##c.kcalw1w3mean_C c.timew3w4##c.energystoresw1w3mean_C c.timew3w4##c.invmillsmixed if sample8==1 || HNDID: timew3w4

xtmixed chol c.timew3w4##c.food_price_BALTfinW1W3mean_C##race c.timew3w4##c.DASH_scorew1w3mean_C c.timew3w4##c.Agew1_C c.timew3w4##c.Agew3_C c.timew3w4##sex c.timew3w4##race c.timew3w4##pir c.timew3w4##edubr c.timew3w4##employed c.timew3w4##wrattbr c.timew3w4##smoke c.timew3w4##currdrugs c.timew3w4##c.bmi_C c.timew3w4##SRHbr c.timew3w4##c.kcalw1w3mean_C c.timew3w4##c.energystoresw1w3mean_C c.timew3w4##c.invmillsmixed if sample8==1 || HNDID: timew3w4

xtmixed chol c.timew3w4##c.food_price_BALTfinW1W3mean_C##pir c.timew3w4##c.DASH_scorew1w3mean_C c.timew3w4##c.Agew1_C c.timew3w4##c.Agew3_C c.timew3w4##sex c.timew3w4##race c.timew3w4##pir c.timew3w4##edubr c.timew3w4##employed c.timew3w4##wrattbr c.timew3w4##smoke c.timew3w4##currdrugs c.timew3w4##c.bmi_C c.timew3w4##SRHbr c.timew3w4##c.kcalw1w3mean_C c.timew3w4##c.energystoresw1w3mean_C c.timew3w4##c.invmillsmixed if sample8==1 || HNDID: timew3w4

//HDL-C//

xtmixed hdl c.timew3w4##c.food_price_BALTfinW1W3mean_C##sex c.timew3w4##c.DASH_scorew1w3mean_C c.timew3w4##c.Agew1_C c.timew3w4##c.Agew3_C c.timew3w4##sex c.timew3w4##race c.timew3w4##pir c.timew3w4##edubr c.timew3w4##employed c.timew3w4##wrattbr c.timew3w4##smoke c.timew3w4##currdrugs c.timew3w4##c.bmi_C c.timew3w4##SRHbr c.timew3w4##c.kcalw1w3mean_C c.timew3w4##c.energystoresw1w3mean_C c.timew3w4##c.invmillsmixed if sample8==1 || HNDID: timew3w4

xtmixed hdl c.timew3w4##c.food_price_BALTfinW1W3mean_C##race c.timew3w4##c.DASH_scorew1w3mean_C c.timew3w4##c.Agew1_C c.timew3w4##c.Agew3_C c.timew3w4##sex c.timew3w4##race c.timew3w4##pir c.timew3w4##edubr c.timew3w4##employed c.timew3w4##wrattbr c.timew3w4##smoke c.timew3w4##currdrugs c.timew3w4##c.bmi_C c.timew3w4##SRHbr c.timew3w4##c.kcalw1w3mean_C c.timew3w4##c.energystoresw1w3mean_C c.timew3w4##c.invmillsmixed if sample8==1 || HNDID: timew3w4

xtmixed hdl c.timew3w4##c.food_price_BALTfinW1W3mean_C##pir c.timew3w4##c.DASH_scorew1w3mean_C c.timew3w4##c.Agew1_C c.timew3w4##c.Agew3_C c.timew3w4##sex c.timew3w4##race c.timew3w4##pir c.timew3w4##edubr c.timew3w4##employed c.timew3w4##wrattbr c.timew3w4##smoke c.timew3w4##currdrugs c.timew3w4##c.bmi_C c.timew3w4##SRHbr c.timew3w4##c.kcalw1w3mean_C c.timew3w4##c.energystoresw1w3mean_C c.timew3w4##c.invmillsmixed if sample8==1 || HNDID: timew3w4

//HBGA1C//

```
xtmixed hgba1c c.timew3w4##c.food_price_BALTfinW1W3mean_C##sex c.timew3w4##c.DASH_scorew1w3mean_C c.timew3w4##c.Agew1_C c.timew3w4##c.Agew3_C c.timew3w4##sex c.timew3w4##race c.timew3w4##pir c.timew3w4##edubr c.timew3w4##employed c.timew3w4##wrattbr c.timew3w4##smoke c.timew3w4##currdrugs c.timew3w4##c.bmi_C c.timew3w4##SRHbr c.timew3w4##c.kcalw1w3mean_C c.timew3w4##c.energystoresw1w3mean_C c.timew3w4##c.invmillsmixed if sample8==1 || HNDID: timew3w4

xtmixed hgba1c c.timew3w4##c.food_price_BALTfinW1W3mean_C##race c.timew3w4##c.DASH_scorew1w3mean_C c.timew3w4##c.Agew1_C c.timew3w4##c.Agew3_C c.timew3w4##sex c.timew3w4##race c.timew3w4##pir c.timew3w4##edubr c.timew3w4##employed c.timew3w4##wrattbr c.timew3w4##smoke c.timew3w4##currdrugs c.timew3w4##c.bmi_C c.timew3w4##SRHbr c.timew3w4##c.kcalw1w3mean_C c.timew3w4##c.energystoresw1w3mean_C c.timew3w4##c.invmillsmixed if sample8==1 || HNDID: timew3w4

xtmixed hgba1c c.timew3w4##c.food_price_BALTfinW1W3mean_C##pir c.timew3w4##c.DASH_scorew1w3mean_C c.timew3w4##c.Agew1_C c.timew3w4##c.Agew3_C c.timew3w4##sex c.timew3w4##race c.timew3w4##pir c.timew3w4##edubr c.timew3w4##employed c.timew3w4##wrattbr c.timew3w4##smoke c.timew3w4##currdrugs c.timew3w4##c.bmi_C c.timew3w4##SRHbr c.timew3w4##c.kcalw1w3mean_C c.timew3w4##c.energystoresw1w3mean_C c.timew3w4##c.invmillsmixed if sample8==1 || HNDID: timew3w4
```

//WHR//

```
xtmixed whr c.timew3w4##c.food_price_BALTfinW1W3mean_C##sex c.timew3w4##c.DASH_scorew1w3mean_C c.timew3w4##c.Agew1_C c.timew3w4##c.Agew3_C c.timew3w4##sex c.timew3w4##race c.timew3w4##pir c.timew3w4##edubr c.timew3w4##employed c.timew3w4##wrattbr c.timew3w4##smoke c.timew3w4##currdrugs c.timew3w4##c.bmi_C c.timew3w4##SRHbr c.timew3w4##c.kcalw1w3mean_C c.timew3w4##c.energystoresw1w3mean_C c.timew3w4##c.invmillsmixed if sample8==1 || HNDID: timew3w4

xtmixed whr c.timew3w4##c.food_price_BALTfinW1W3mean_C##race c.timew3w4##c.DASH_scorew1w3mean_C c.timew3w4##c.Agew1_C c.timew3w4##c.Agew3_C c.timew3w4##sex c.timew3w4##race c.timew3w4##pir c.timew3w4##edubr c.timew3w4##employed c.timew3w4##wrattbr c.timew3w4##smoke c.timew3w4##currdrugs c.timew3w4##c.bmi_C c.timew3w4##SRHbr c.timew3w4##c.kcalw1w3mean_C c.timew3w4##c.energystoresw1w3mean_C c.timew3w4##c.invmillsmixed if sample8==1 || HNDID: timew3w4

xtmixed whr c.timew3w4##c.food_price_BALTfinW1W3mean_C##pir c.timew3w4##c.DASH_scorew1w3mean_C c.timew3w4##c.Agew1_C c.timew3w4##c.Agew3_C c.timew3w4##sex c.timew3w4##race c.timew3w4##pir c.timew3w4##edubr c.timew3w4##employed c.timew3w4##wrattbr c.timew3w4##smoke c.timew3w4##currdrugs
```

c.timew3w4##c.bmi_C c.timew3w4##SRHbr c.timew3w4##c.kcalw1w3mean_C c.timew3w4##c.energystoresw1w3mean_C c.timew3w4##c.invmillsmixed if sample8==1 || HNDID: timew3w4

//SBP//

xtmixed bpsys c.timew3w4##c.food_price_BALTfinW1W3mean_C##sex c.timew3w4##c.DASH_scorew1w3mean_C c.timew3w4##c.Agew1_C c.timew3w4##c.Agew3_C c.timew3w4##sex c.timew3w4##race c.timew3w4##pir c.timew3w4##edubr c.timew3w4##employed c.timew3w4##wrattbr c.timew3w4##smoke c.timew3w4##currdrugs c.timew3w4##c.bmi_C c.timew3w4##SRHbr c.timew3w4##c.kcalw1w3mean_C c.timew3w4##c.energystoresw1w3mean_C c.timew3w4##c.invmillsmixed if sample8==1 || HNDID: timew3w4

xtmixed bpsys c.timew3w4##c.food_price_BALTfinW1W3mean_C##race c.timew3w4##c.DASH_scorew1w3mean_C c.timew3w4##c.Agew1_C c.timew3w4##c.Agew3_C c.timew3w4##sex c.timew3w4##race c.timew3w4##pir c.timew3w4##edubr c.timew3w4##employed c.timew3w4##wrattbr c.timew3w4##smoke c.timew3w4##currdrugs c.timew3w4##c.bmi_C c.timew3w4##SRHbr c.timew3w4##c.kcalw1w3mean_C c.timew3w4##c.energystoresw1w3mean_C c.timew3w4##c.invmillsmixed if sample8==1 || HNDID: timew3w4

xtmixed bpsys c.timew3w4##c.food_price_BALTfinW1W3mean_C##pir c.timew3w4##c.DASH_scorew1w3mean_C c.timew3w4##c.Agew1_C c.timew3w4##c.Agew3_C c.timew3w4##sex c.timew3w4##race c.timew3w4##pir c.timew3w4##edubr c.timew3w4##employed c.timew3w4##wrattbr c.timew3w4##smoke c.timew3w4##currdrugs c.timew3w4##c.bmi_C c.timew3w4##SRHbr c.timew3w4##c.kcalw1w3mean_C c.timew3w4##c.energystoresw1w3mean_C c.timew3w4##c.invmillsmixed if sample8==1 || HNDID: timew3w4

//DBP//

xtmixed bpdia c.timew3w4##c.food_price_BALTfinW1W3mean_C##sex c.timew3w4##c.DASH_scorew1w3mean_C c.timew3w4##c.Agew1_C c.timew3w4##c.Agew3_C c.timew3w4##sex c.timew3w4##race c.timew3w4##pir c.timew3w4##edubr c.timew3w4##employed c.timew3w4##wrattbr c.timew3w4##smoke c.timew3w4##currdrugs c.timew3w4##c.bmi_C c.timew3w4##SRHbr c.timew3w4##c.kcalw1w3mean_C c.timew3w4##c.energystoresw1w3mean_C c.timew3w4##c.invmillsmixed if sample8==1 || HNDID: timew3w4

xtmixed bpdia c.timew3w4##c.food_price_BALTfinW1W3mean_C##race c.timew3w4##c.DASH_scorew1w3mean_C c.timew3w4##c.Agew1_C c.timew3w4##c.Agew3_C c.timew3w4##sex c.timew3w4##race c.timew3w4##pir c.timew3w4##edubr c.timew3w4##employed c.timew3w4##wrattbr c.timew3w4##smoke c.timew3w4##currdrugs c.timew3w4##c.bmi_C c.timew3w4##SRHbr c.timew3w4##c.kcalw1w3mean_C c.timew3w4##c.energystoresw1w3mean_C c.timew3w4##c.invmillsmixed if sample8==1 || HNDID: timew3w4

xtmixed bpdia c.timew3w4##c.food_price_BALTfinW1W3mean_C##pir c.timew3w4##c.DASH_scorew1w3mean_C c.timew3w4##c.Agew1_C c.timew3w4##c.Agew3_C c.timew3w4##sex c.timew3w4##race c.timew3w4##pir c.timew3w4##edubr c.timew3w4##employed c.timew3w4##wrattbr c.timew3w4##smoke c.timew3w4##currdrugs

c.timew3w4##c.bmi_C c.timew3w4##SRHbr c.timew3w4##c.kcalw1w3mean_C c.timew3w4##c.energystoresw1w3mean_C c.timew3w4##c.invmillsmixed if sample8==1 || HNDID: timew3w4

//HR//

xtmixed hr c.timew3w4##c.food_price_BALTfinW1W3mean_C##sex c.timew3w4##c.DASH_scorew1w3mean_C c.timew3w4##c.Agew1_C c.timew3w4##c.Agew3_C c.timew3w4##sex c.timew3w4##race c.timew3w4##pir c.timew3w4##edubr c.timew3w4##employed c.timew3w4##wrattbr c.timew3w4##smoke c.timew3w4##currdrugs c.timew3w4##c.bmi_C c.timew3w4##SRHbr c.timew3w4##c.kcalw1w3mean_C c.timew3w4##c.energystoresw1w3mean_C c.timew3w4##c.invmillsmixed if sample8==1 || HNDID: timew3w4

xtmixed hr c.timew3w4##c.food_price_BALTfinW1W3mean_C##race c.timew3w4##c.DASH_scorew1w3mean_C c.timew3w4##c.Agew1_C c.timew3w4##c.Agew3_C c.timew3w4##sex c.timew3w4##race c.timew3w4##pir c.timew3w4##edubr c.timew3w4##employed c.timew3w4##wrattbr c.timew3w4##smoke c.timew3w4##currdrugs c.timew3w4##c.bmi_C c.timew3w4##SRHbr c.timew3w4##c.kcalw1w3mean_C c.timew3w4##c.energystoresw1w3mean_C c.timew3w4##c.invmillsmixed if sample8==1 || HNDID: timew3w4

xtmixed hr c.timew3w4##c.food_price_BALTfinW1W3mean_C##pir c.timew3w4##c.DASH_scorew1w3mean_C c.timew3w4##c.Agew1_C c.timew3w4##c.Agew3_C c.timew3w4##sex c.timew3w4##race c.timew3w4##pir c.timew3w4##edubr c.timew3w4##employed c.timew3w4##wrattbr c.timew3w4##smoke c.timew3w4##currdrugs c.timew3w4##c.bmi_C c.timew3w4##SRHbr c.timew3w4##c.kcalw1w3mean_C c.timew3w4##c.energystoresw1w3mean_C c.timew3w4##c.invmillsmixed if sample8==1 || HNDID: timew3w4

TABLE 3, MODEL 1, INTERACTION WITH DASH*

cd "G:\...\DATA"

use HANDLS_Allostaticload_dietfcfinal, clear

//ALLOSTATIC LOAD//

xtmixed allostatic_prop c.timew3w4##c.food_price_BALTfinW1W3mean_C c.timew3w4##c.DASH_scorew1w3mean_C##sex c.timew3w4##c.Agew1_C c.timew3w4##c.Agew3_C c.timew3w4 c.timew3w4 c.timew3w4 c.timew3w4##edubr c.timew3w4##employed c.timew3w4##wrattbr c.timew3w4##smoke c.timew3w4##currdrugs c.timew3w4##c.bmi_C c.timew3w4##SRHbr c.timew3w4##c.kcalw1w3mean_C c.timew3w4##c.energystoresw1w3mean_C c.timew3w4##c.invmillsmixed if sample8==1 || HNDID: timew3w4

xtmixed allostatic_prop c.timew3w4##c.food_price_BALTfinW1W3mean_C c.timew3w4##c.DASH_scorew1w3mean_C##race c.timew3w4##c.Agew1_C c.timew3w4##c.Agew3_C c.timew3w4 c.timew3w4 c.timew3w4 c.timew3w4##edubr c.timew3w4##employed c.timew3w4##wrattbr c.timew3w4##smoke c.timew3w4##currdrugs c.timew3w4##c.bmi_C c.timew3w4##SRHbr c.timew3w4##c.kcalw1w3mean_C c.timew3w4##c.energystoresw1w3mean_C c.timew3w4##c.invmillsmixed if sample8==1 || HNDID: timew3w4

xtmixed allostatic_prop c.timew3w4##c.food_price_BALTfinW1W3mean_C c.timew3w4##c.DASH_scorew1w3mean_C##pir c.timew3w4##c.Agew1_C c.timew3w4##c.Agew3_C c.timew3w4 c.timew3w4 c.timew3w4 c.timew3w4##edubr c.timew3w4##employed c.timew3w4##wrattbr c.timew3w4##smoke c.timew3w4##currdrugs c.timew3w4##c.bmi_C c.timew3w4##SRHbr c.timew3w4##c.kcalw1w3mean_C c.timew3w4##c.energystoresw1w3mean_C c.timew3w4##c.invmillsmixed if sample8==1 || HNDID: timew3w4

//ALB//

xtmixed albumin c.timew3w4##c.food_price_BALTfinW1W3mean_C c.timew3w4##c.DASH_scorew1w3mean_C##sex c.timew3w4##c.Agew1_C c.timew3w4##c.Agew3_C c.timew3w4 c.timew3w4 c.timew3w4 c.timew3w4##edubr c.timew3w4##employed c.timew3w4##wrattbr c.timew3w4##smoke c.timew3w4##currdrugs c.timew3w4##c.bmi_C c.timew3w4##SRHbr c.timew3w4##c.kcalw1w3mean_C c.timew3w4##c.energystoresw1w3mean_C c.timew3w4##c.invmillsmixed if sample8==1 || HNDID: timew3w4

xtmixed albumin c.timew3w4##c.food_price_BALTfinW1W3mean_C c.timew3w4##c.DASH_scorew1w3mean_C##race c.timew3w4##c.Agew1_C c.timew3w4##c.Agew3_C c.timew3w4 c.timew3w4 c.timew3w4 c.timew3w4##edubr c.timew3w4##employed c.timew3w4##wrattbr c.timew3w4##smoke c.timew3w4##currdrugs c.timew3w4##c.bmi_C c.timew3w4##SRHbr c.timew3w4##c.kcalw1w3mean_C c.timew3w4##c.energystoresw1w3mean_C c.timew3w4##c.invmillsmixed if sample8==1 || HNDID: timew3w4

xtmixed albumin c.timew3w4##c.food_price_BALTfinW1W3mean_C c.timew3w4##c.DASH_scorew1w3mean_C##pir c.timew3w4##c.Agew1_C c.timew3w4##c.Agew3_C c.timew3w4 c.timew3w4 c.timew3w4 c.timew3w4##edubr c.timew3w4##employed c.timew3w4##wrattbr c.timew3w4##smoke c.timew3w4##currdrugs c.timew3w4##c.bmi_C c.timew3w4##SRHbr c.timew3w4##c.kcalw1w3mean_C c.timew3w4##c.energystoresw1w3mean_C c.timew3w4##c.invmillsmixed if sample8==1 || HNDID: timew3w4

//CRP//

xtmixed crp c.timew3w4##c.food_price_BALTfinW1W3mean_C c.timew3w4##c.DASH_scorew1w3mean_C##sex c.timew3w4##c.Agew1_C c.timew3w4##c.Agew3_C c.timew3w4 c.timew3w4 c.timew3w4 c.timew3w4##edubr c.timew3w4##employed c.timew3w4##wrattbr c.timew3w4##smoke c.timew3w4##currdrugs c.timew3w4##c.bmi_C c.timew3w4##SRHbr c.timew3w4##c.kcalw1w3mean_C c.timew3w4##c.energystoresw1w3mean_C c.timew3w4##c.invmillsmixed if sample8==1 || HNDID: timew3w4

xtmixed crp c.timew3w4##c.food_price_BALTfinW1W3mean_C c.timew3w4##c.DASH_scorew1w3mean_C##race c.timew3w4##c.Agew1_C c.timew3w4##c.Agew3_C c.timew3w4 c.timew3w4 c.timew3w4 c.timew3w4##edubr

c.timew3w4##employed c.timew3w4##wrattbr c.timew3w4##smoke c.timew3w4##currdrugs c.timew3w4##c.bmi_C c.timew3w4##SRHbr c.timew3w4##c.kcalw1w3mean_C c.timew3w4##c.energystoresw1w3mean_C c.timew3w4##c.invmillsmixed if sample8==1 || HNDID: timew3w4

xtmixed crp c.timew3w4##c.food_price_BALTfinW1W3mean_C c.timew3w4##c.DASH_scorew1w3mean_C##pir c.timew3w4##c.Agew1_C c.timew3w4##c.Agew3_C c.timew3w4 c.timew3w4 c.timew3w4 c.timew3w4##edubr c.timew3w4##employed c.timew3w4##wrattbr c.timew3w4##smoke c.timew3w4##currdrugs c.timew3w4##c.bmi_C c.timew3w4##SRHbr c.timew3w4##c.kcalw1w3mean_C c.timew3w4##c.energystoresw1w3mean_C c.timew3w4##c.invmillsmixed if sample8==1 || HNDID: timew3w4

//Cholesterol//

xtmixed chol c.timew3w4##c.food_price_BALTfinW1W3mean_C c.timew3w4##c.DASH_scorew1w3mean_C##sex c.timew3w4##c.Agew1_C c.timew3w4##c.Agew3_C c.timew3w4 c.timew3w4 c.timew3w4 c.timew3w4##edubr c.timew3w4##employed c.timew3w4##wrattbr c.timew3w4##smoke c.timew3w4##currdrugs c.timew3w4##c.bmi_C c.timew3w4##SRHbr c.timew3w4##c.kcalw1w3mean_C c.timew3w4##c.energystoresw1w3mean_C c.timew3w4##c.invmillsmixed if sample8==1 || HNDID: timew3w4

xtmixed chol c.timew3w4##c.food_price_BALTfinW1W3mean_C c.timew3w4##c.DASH_scorew1w3mean_C##race c.timew3w4##c.Agew1_C c.timew3w4##c.Agew3_C c.timew3w4 c.timew3w4 c.timew3w4 c.timew3w4##edubr c.timew3w4##employed c.timew3w4##wrattbr c.timew3w4##smoke c.timew3w4##currdrugs c.timew3w4##c.bmi_C c.timew3w4##SRHbr c.timew3w4##c.kcalw1w3mean_C c.timew3w4##c.energystoresw1w3mean_C c.timew3w4##c.invmillsmixed if sample8==1 || HNDID: timew3w4

xtmixed chol c.timew3w4##c.food_price_BALTfinW1W3mean_C c.timew3w4##c.DASH_scorew1w3mean_C##pir c.timew3w4##c.Agew1_C c.timew3w4##c.Agew3_C c.timew3w4 c.timew3w4 c.timew3w4 c.timew3w4##edubr c.timew3w4##employed c.timew3w4##wrattbr c.timew3w4##smoke c.timew3w4##currdrugs c.timew3w4##c.bmi_C c.timew3w4##SRHbr c.timew3w4##c.kcalw1w3mean_C c.timew3w4##c.energystoresw1w3mean_C c.timew3w4##c.invmillsmixed if sample8==1 || HNDID: timew3w4

//HDL-C//

xtmixed hdl c.timew3w4##c.food_price_BALTfinW1W3mean_C c.timew3w4##c.DASH_scorew1w3mean_C##sex c.timew3w4##c.Agew1_C c.timew3w4##c.Agew3_C c.timew3w4 c.timew3w4 c.timew3w4 c.timew3w4##edubr c.timew3w4##employed c.timew3w4##wrattbr c.timew3w4##smoke c.timew3w4##currdrugs c.timew3w4##c.bmi_C c.timew3w4##SRHbr c.timew3w4##c.kcalw1w3mean_C c.timew3w4##c.energystoresw1w3mean_C c.timew3w4##c.invmillsmixed if sample8==1 || HNDID: timew3w4

xtmixed hdl c.timew3w4##c.food_price_BALTfinW1W3mean_C c.timew3w4##c.DASH_scorew1w3mean_C##race c.timew3w4##c.Agew1_C c.timew3w4##c.Agew3_C c.timew3w4 c.timew3w4 c.timew3w4 c.timew3w4##edubr c.timew3w4##employed c.timew3w4##wrattbr c.timew3w4##smoke c.timew3w4##currdrugs c.timew3w4##c.bmi_C c.timew3w4##SRHbr c.timew3w4##c.kcalw1w3mean_C c.timew3w4##c.energystoresw1w3mean_C c.timew3w4##c.invmillsmixed if sample8==1 || HNDID: timew3w4

xtmixed hdl c.timew3w4##c.food_price_BALTfinW1W3mean_C c.timew3w4##c.DASH_scorew1w3mean_C##pir c.timew3w4##c.Agew1_C c.timew3w4##c.Agew3_C c.timew3w4 c.timew3w4 c.timew3w4 c.timew3w4##edubr c.timew3w4##employed c.timew3w4##wrattbr c.timew3w4##smoke c.timew3w4##currdrugs c.timew3w4##c.bmi_C c.timew3w4##SRHbr c.timew3w4##c.kcalw1w3mean_C c.timew3w4##c.energystoresw1w3mean_C c.timew3w4##c.invmillsmixed if sample8==1 || HNDID: timew3w4

//HBGA1C//

xtmixed hgba1c c.timew3w4##c.food_price_BALTfinW1W3mean_C c.timew3w4##c.DASH_scorew1w3mean_C##sex c.timew3w4##c.Agew1_C c.timew3w4##c.Agew3_C c.timew3w4 c.timew3w4 c.timew3w4 c.timew3w4##edubr c.timew3w4##employed c.timew3w4##wrattbr c.timew3w4##smoke c.timew3w4##currdrugs c.timew3w4##c.bmi_C c.timew3w4##SRHbr c.timew3w4##c.kcalw1w3mean_C c.timew3w4##c.energystoresw1w3mean_C c.timew3w4##c.invmillsmixed if sample8==1 || HNDID: timew3w4

xtmixed hgba1c c.timew3w4##c.food_price_BALTfinW1W3mean_C c.timew3w4##c.DASH_scorew1w3mean_C##race c.timew3w4##c.Agew1_C c.timew3w4##c.Agew3_C c.timew3w4 c.timew3w4 c.timew3w4 c.timew3w4##edubr c.timew3w4##employed c.timew3w4##wrattbr c.timew3w4##smoke c.timew3w4##currdrugs c.timew3w4##c.bmi_C c.timew3w4##SRHbr c.timew3w4##c.kcalw1w3mean_C c.timew3w4##c.energystoresw1w3mean_C c.timew3w4##c.invmillsmixed if sample8==1 || HNDID: timew3w4

xtmixed hgba1c c.timew3w4##c.food_price_BALTfinW1W3mean_C c.timew3w4##c.DASH_scorew1w3mean_C##pir c.timew3w4##c.Agew1_C c.timew3w4##c.Agew3_C c.timew3w4 c.timew3w4 c.timew3w4 c.timew3w4##edubr c.timew3w4##employed c.timew3w4##wrattbr c.timew3w4##smoke c.timew3w4##currdrugs c.timew3w4##c.bmi_C c.timew3w4##SRHbr c.timew3w4##c.kcalw1w3mean_C c.timew3w4##c.energystoresw1w3mean_C c.timew3w4##c.invmillsmixed if sample8==1 || HNDID: timew3w4

//WHR//

xtmixed whr c.timew3w4##c.food_price_BALTfinW1W3mean_C c.timew3w4##c.DASH_scorew1w3mean_C##sex c.timew3w4##c.Agew1_C c.timew3w4##c.Agew3_C c.timew3w4 c.timew3w4 c.timew3w4 c.timew3w4##edubr c.timew3w4##employed c.timew3w4##wrattbr c.timew3w4##smoke c.timew3w4##currdrugs c.timew3w4##c.bmi_C

c.timew3w4##SRHbr c.timew3w4##c.kcalw1w3mean_C c.timew3w4##c.energystoresw1w3mean_C c.timew3w4##c.invmillsmixed if sample8==1 || HNDID: timew3w4

xtmixed whr c.timew3w4##c.food_price_BALTfinW1W3mean_C c.timew3w4##c.DASH_scorew1w3mean_C##race c.timew3w4##c.Agew1_C c.timew3w4##c.Agew3_C c.timew3w4 c.timew3w4 c.timew3w4 c.timew3w4##edubr c.timew3w4##employed c.timew3w4##wrattbr c.timew3w4##smoke c.timew3w4##currdrugs c.timew3w4##c.bmi_C c.timew3w4##SRHbr c.timew3w4##c.kcalw1w3mean_C c.timew3w4##c.energystoresw1w3mean_C c.timew3w4##c.invmillsmixed if sample8==1 || HNDID: timew3w4

xtmixed whr c.timew3w4##c.food_price_BALTfinW1W3mean_C c.timew3w4##c.DASH_scorew1w3mean_C##pir c.timew3w4##c.Agew1_C c.timew3w4##c.Agew3_C c.timew3w4 c.timew3w4 c.timew3w4 c.timew3w4##edubr c.timew3w4##employed c.timew3w4##wrattbr c.timew3w4##smoke c.timew3w4##currdrugs c.timew3w4##c.bmi_C c.timew3w4##SRHbr c.timew3w4##c.kcalw1w3mean_C c.timew3w4##c.energystoresw1w3mean_C c.timew3w4##c.invmillsmixed if sample8==1 || HNDID: timew3w4

//SBP//

xtmixed bpsys c.timew3w4##c.food_price_BALTfinW1W3mean_C c.timew3w4##c.DASH_scorew1w3mean_C##sex c.timew3w4##c.Agew1_C c.timew3w4##c.Agew3_C c.timew3w4 c.timew3w4 c.timew3w4 c.timew3w4##edubr c.timew3w4##employed c.timew3w4##wrattbr c.timew3w4##smoke c.timew3w4##currdrugs c.timew3w4##c.bmi_C c.timew3w4##SRHbr c.timew3w4##c.kcalw1w3mean_C c.timew3w4##c.energystoresw1w3mean_C c.timew3w4##c.invmillsmixed if sample8==1 || HNDID: timew3w4

xtmixed bpsys c.timew3w4##c.food_price_BALTfinW1W3mean_C c.timew3w4##c.DASH_scorew1w3mean_C##race c.timew3w4##c.Agew1_C c.timew3w4##c.Agew3_C c.timew3w4 c.timew3w4 c.timew3w4 c.timew3w4##edubr c.timew3w4##employed c.timew3w4##wrattbr c.timew3w4##smoke c.timew3w4##currdrugs c.timew3w4##c.bmi_C c.timew3w4##SRHbr c.timew3w4##c.kcalw1w3mean_C c.timew3w4##c.energystoresw1w3mean_C c.timew3w4##c.invmillsmixed if sample8==1 || HNDID: timew3w4

xtmixed bpsys c.timew3w4##c.food_price_BALTfinW1W3mean_C c.timew3w4##c.DASH_scorew1w3mean_C##pir c.timew3w4##c.Agew1_C c.timew3w4##c.Agew3_C c.timew3w4 c.timew3w4 c.timew3w4 c.timew3w4##edubr c.timew3w4##employed c.timew3w4##wrattbr c.timew3w4##smoke c.timew3w4##currdrugs c.timew3w4##c.bmi_C c.timew3w4##SRHbr c.timew3w4##c.kcalw1w3mean_C c.timew3w4##c.energystoresw1w3mean_C c.timew3w4##c.invmillsmixed if sample8==1 || HNDID: timew3w4

//DBP//

xtmixed bpdia c.timew3w4##c.food_price_BALTfinW1W3mean_C c.timew3w4##c.DASH_scorew1w3mean_C##sex c.timew3w4##c.Agew1_C c.timew3w4##c.Agew3_C c.timew3w4 c.timew3w4 c.timew3w4 c.timew3w4##edubr c.timew3w4##employed c.timew3w4##wrattbr c.timew3w4##smoke c.timew3w4##currdrugs c.timew3w4##c.bmi_C

c.timew3w4##SRHbr c.timew3w4##c.kcalw1w3mean_C c.timew3w4##c.energystoresw1w3mean_C c.timew3w4##c.invmillsmixed if sample8==1 || HNDID: timew3w4

xtmixed bpdia c.timew3w4##c.food_price_BALTfinW1W3mean_C c.timew3w4##c.DASH_scorew1w3mean_C##race c.timew3w4##c.Agew1_C c.timew3w4##c.Agew3_C c.timew3w4 c.timew3w4 c.timew3w4 c.timew3w4##edubr c.timew3w4##employed c.timew3w4##wrattbr c.timew3w4##smoke c.timew3w4##currdrugs c.timew3w4##c.bmi_C c.timew3w4##SRHbr c.timew3w4##c.kcalw1w3mean_C c.timew3w4##c.energystoresw1w3mean_C c.timew3w4##c.invmillsmixed if sample8==1 || HNDID: timew3w4

xtmixed bpdia c.timew3w4##c.food_price_BALTfinW1W3mean_C c.timew3w4##c.DASH_scorew1w3mean_C##pir c.timew3w4##c.Agew1_C c.timew3w4##c.Agew3_C c.timew3w4 c.timew3w4 c.timew3w4 c.timew3w4##edubr c.timew3w4##employed c.timew3w4##wrattbr c.timew3w4##smoke c.timew3w4##currdrugs c.timew3w4##c.bmi_C c.timew3w4##SRHbr c.timew3w4##c.kcalw1w3mean_C c.timew3w4##c.energystoresw1w3mean_C c.timew3w4##c.invmillsmixed if sample8==1 || HNDID: timew3w4

//HR//

xtmixed hr c.timew3w4##c.food_price_BALTfinW1W3mean_C c.timew3w4##c.DASH_scorew1w3mean_C##sex c.timew3w4##c.Agew1_C c.timew3w4##c.Agew3_C c.timew3w4 c.timew3w4 c.timew3w4 c.timew3w4##edubr c.timew3w4##employed c.timew3w4##wrattbr c.timew3w4##smoke c.timew3w4##currdrugs c.timew3w4##c.bmi_C c.timew3w4##SRHbr c.timew3w4##c.kcalw1w3mean_C c.timew3w4##c.energystoresw1w3mean_C c.timew3w4##c.invmillsmixed if sample8==1 || HNDID: timew3w4

xtmixed hr c.timew3w4##c.food_price_BALTfinW1W3mean_C c.timew3w4##c.DASH_scorew1w3mean_C##race c.timew3w4##c.Agew1_C c.timew3w4##c.Agew3_C c.timew3w4 c.timew3w4 c.timew3w4 c.timew3w4##edubr c.timew3w4##employed c.timew3w4##wrattbr c.timew3w4##smoke c.timew3w4##currdrugs c.timew3w4##c.bmi_C c.timew3w4##SRHbr c.timew3w4##c.kcalw1w3mean_C c.timew3w4##c.energystoresw1w3mean_C c.timew3w4##c.invmillsmixed if sample8==1 || HNDID: timew3w4

xtmixed hr c.timew3w4##c.food_price_BALTfinW1W3mean_C c.timew3w4##c.DASH_scorew1w3mean_C##pir c.timew3w4##c.Agew1_C c.timew3w4##c.Agew3_C c.timew3w4 c.timew3w4 c.timew3w4 c.timew3w4##edubr c.timew3w4##employed c.timew3w4##wrattbr c.timew3w4##smoke c.timew3w4##currdrugs c.timew3w4##c.bmi_C c.timew3w4##SRHbr c.timew3w4##c.kcalw1w3mean_C c.timew3w4##c.energystoresw1w3mean_C c.timew3w4##c.invmillsmixed if sample8==1 || HNDID: timew3w4

TABLE 3, MODEL 2, INTERACTION WITH FOOD COST*

cd "G:\...\DATA"

use HANDLS_Allostaticload_dietfcfinal, clear

//ALLOSTATIC LOAD//

xtmixed allostatic_prop c.timew3w4##c.food_price_BALTfinW1W3mean_C##sex c.timew3w4##c.Agew1_C c.timew3w4##c.Agew3_C c.timew3w4##sex c.timew3w4##race c.timew3w4##pir c.timew3w4##edubr c.timew3w4##employed c.timew3w4##wrattbr c.timew3w4##smoke c.timew3w4##currdrugs c.timew3w4##c.bmi_C c.timew3w4##SRHbr c.timew3w4##c.kcalw1w3mean_C c.timew3w4##c.energystoresw1w3mean_C c.timew3w4##c.invmillsmixed if sample8==1 || HNDID: timew3w4

xtmixed allostatic_prop c.timew3w4##c.food_price_BALTfinW1W3mean_C##race c.timew3w4##c.Agew1_C c.timew3w4##c.Agew3_C c.timew3w4##sex c.timew3w4##race c.timew3w4##pir c.timew3w4##edubr c.timew3w4##employed c.timew3w4##wrattbr c.timew3w4##smoke c.timew3w4##currdrugs c.timew3w4##c.bmi_C c.timew3w4##SRHbr c.timew3w4##c.kcalw1w3mean_C c.timew3w4##c.energystoresw1w3mean_C c.timew3w4##c.invmillsmixed if sample8==1 || HNDID: timew3w4

xtmixed allostatic_prop c.timew3w4##c.food_price_BALTfinW1W3mean_C##pir c.timew3w4##c.Agew1_C c.timew3w4##c.Agew3_C c.timew3w4##sex c.timew3w4##race c.timew3w4##pir c.timew3w4##edubr c.timew3w4##employed c.timew3w4##wrattbr c.timew3w4##smoke c.timew3w4##currdrugs c.timew3w4##c.bmi_C c.timew3w4##SRHbr c.timew3w4##c.kcalw1w3mean_C c.timew3w4##c.energystoresw1w3mean_C c.timew3w4##c.invmillsmixed if sample8==1 || HNDID: timew3w4

TABLE 3, MODEL 3, INTERACTION WITH DASH*

cd "G:\...\DATA"

use HANDLS_Allostaticload_dietfcfinal, clear

//ALLOSTATIC LOAD//

xtmixed allostatic_prop c.timew3w4##c.DASH_scorew1w3mean_C##sex c.timew3w4##c.Agew1_C c.timew3w4##c.Agew3_C c.timew3w4 c.timew3w4 c.timew3w4 c.timew3w4##edubr c.timew3w4##employed c.timew3w4##wrattbr c.timew3w4##smoke c.timew3w4##currdrugs c.timew3w4##c.bmi_C c.timew3w4##SRHbr c.timew3w4##c.kcalw1w3mean_C c.timew3w4##c.energystoresw1w3mean_C c.timew3w4##c.invmillsmixed if sample8==1 || HNDID: timew3w4

xtmixed allostatic_prop c.timew3w4##c.DASH_scorew1w3mean_C##race c.timew3w4##c.Agew1_C c.timew3w4##c.Agew3_C c.timew3w4 c.timew3w4 c.timew3w4 c.timew3w4##edubr c.timew3w4##employed c.timew3w4##wrattbr c.timew3w4##smoke c.timew3w4##currdrugs c.timew3w4##c.bmi_C c.timew3w4##SRHbr

```
c.timew3w4##c.kcalw1w3mean_C c.timew3w4##c.energystoresw1w3mean_C c.timew3w4##c.invmillsmixed if sample8==1 || HNDID: timew3w4

xtmixed allostatic_prop c.timew3w4##c.DASH_scorew1w3mean_C##pir c.timew3w4##c.Agew1_C c.timew3w4##c.Agew3_C c.timew3w4 c.timew3w4 c.timew3w4 c.timew3w4##edubr c.timew3w4##employed c.timew3w4##wrattbr c.timew3w4##smoke c.timew3w4##currdrugs c.timew3w4##c.bmi_C c.timew3w4##SRHbr c.timew3w4##c.kcalw1w3mean_C c.timew3w4##c.energystoresw1w3mean_C c.timew3w4##c.invmillsmixed if sample8==1 || HNDID: timew3w4

****************************Preliminary analyses for SEM******************************************

reg allostatic_propmean DASH_scorew1w3mean food_price_BALTfinW1W3mean sex Agew1 sex Agew1 race pir if finalsample==1

reg allostatic_propmean food_price_BALTfinW1W3mean sex Agew1 race pir if finalsample==1

reg DASH_scorew1w3mean food_price_BALTfinWave1 sex Agew1 race pir if finalsample==1

foreach var of varlist DASH_SatFatw3 DASH_Fatw3 Dash_protein_W3 DASH_cholesterolw3 DASH_fiberw3 DASH_Magnesiumw3 DASH_calciumw3 DASH_potassiumw3 DASH_Sodiumw3 {
reg `var' food_price_BALTfinW1W3mean sex Agew1 race pir if finalsample==1 & sex==0

}

foreach var of varlist DASH_SatFatw3 DASH_Fatw3 Dash_protein_W3 DASH_cholesterolw3 DASH_fiberw3 DASH_Magnesiumw3 DASH_calciumw3 DASH_potassiumw3 DASH_Sodiumw3 {
reg `var' food_price_BALTfinWave3 sex Agew1 race pir if finalsample==1 & sex==0

}

foreach var of varlist albuminw4 crpw4 cholw4 hdlw4 hgba1cw4 whrw4 bpsysw4 bpdiaw4 hrw4 {
reg `var' food_price_BALTfinW1W3mean sex Agew1 race pir if finalsample==1 & sex==0

}
```

```
foreach var of varlist albuminw4 crpw4 cholw4 hdlw4 hgba1cw4 whrw4 bpsysw4 bpdiaw4 hrw4 {
reg `var' DASH_scorew1w3mean sex Agew1 race pir if finalsample==1 & sex==0

}



reg allostatic_propw4 DASH_scorew1w3mean sex Agew1 race pir if finalsample==1 & sex==0
reg allostatic_propw4 food_price_BALTfinW1W3mean sex Agew1 race pir if finalsample==1 & sex==0
reg DASH_scorew1w3mean food_price_BALTfinW1W3mean sex Agew1 race pir if finalsample==1 & sex==0



reg allostatic_propw4 DASH_scorew1w3mean sex Agew1 race pir if finalsample==1 & sex==1
reg allostatic_propw4 food_price_BALTfinW1W3mean sex Agew1 race pir if finalsample==1 & sex==1
reg DASH_scorew1w3mean food_price_BALTfinW1W3mean sex Agew1 race pir if finalsample==1 & sex==1



reg allostatic_propw4 DASH_scorew1w3mean sex Agew1 race pir if finalsample==1 & race==0
reg allostatic_propw4 food_price_BALTfinW1W3mean sex Agew1 race pir if finalsample==1 & race==0
reg DASH_scorew1w3mean food_price_BALTfinW1W3mean sex Agew1 race pir if finalsample==1 & race==0




reg allostatic_propw4 DASH_scorew1w3mean sex Agew1 race pir if finalsample==1 & pir==0
reg allostatic_propw4 food_price_BALTfinW1W3mean sex Agew1 race pir if finalsample==1 & pir==0
reg DASH_scorew1w3mean food_price_BALTfinW1W3mean sex Agew1 race pir if finalsample==1 & pir==0



reg allostatic_propw4 DASH_scorew1w3mean sex Agew1 race pir if finalsample==1 & pir==1
reg allostatic_propw4 food_price_BALTfinW1W3mean sex Agew1 race pir if finalsample==1 & pir==1
reg DASH_scorew1w3mean food_price_BALTfinW1W3mean sex Agew1 race pir if finalsample==1 & pir==1

**sex and race**

reg allostatic_propw4 DASH_scorew1w3mean sex Agew1 race pir if finalsample==1 & sex==0 & race==0
reg allostatic_propw4 food_price_BALTfinW1W3mean sex Agew1 race pir if finalsample==1 & sex==0 & race==0
reg DASH_scorew1w3mean food_price_BALTfinW1W3mean sex Agew1 race pir if finalsample==1 & sex==0 & race==0




reg allostatic_propw4 DASH_scorew1w3mean sex Agew1 race pir if finalsample==1 & sex==0 & race==1
reg allostatic_propw4 food_price_BALTfinW1W3mean sex Agew1 race pir if finalsample==1 & sex==0 & race==1
reg DASH_scorew1w3mean food_price_BALTfinW1W3mean sex Agew1 race pir if finalsample==1 & sex==0 & race==1
```

```
reg allostatic_propw4 DASH_scorew1w3mean sex Agew1 race pir if finalsample==1 & sex==1 & race==0
reg allostatic_propw4 food_price_BALTfinW1W3mean sex Agew1 race pir if finalsample==1 & sex==1 & race==0
reg DASH_scorew1w3mean food_price_BALTfinW1W3mean sex Agew1 race pir if finalsample==1 & sex==1 & race==0



reg allostatic_propw4 DASH_scorew1w3mean sex Agew1 race pir if finalsample==1 & sex==1 & race==1
reg allostatic_propw4 food_price_BALTfinW1W3mean sex Agew1 race pir if finalsample==1 & sex==1 & race==1
reg DASH_scorew1w3mean food_price_BALTfinW1W3mean sex Agew1 race pir if finalsample==1 & sex==1 & race==1

save, replace


//////////////////////////////TABLE 4: paramed MODELS//////////////////////////////

cd "G:\...\DATA"

use HANDLS_Allostaticload_dietfcfinal, clear
foreach var of varlist DASH_SatFatw1w3mean DASH_Fatw1w3mean Dash_protein_W1W3mean DASH_cholesterolw1w3 DASH_fiberw1w3 DASH_Magnesiumw1w3mean DASH_calciumw1w3mean DASH_potassiumw1w3mean DASH_Sodiumw1w3mean {
egen z`var'=std(`var') if finalsample==1
}
keep if finalsample==1
save HANDLS_Allostaticload_dietfcfinal_PARAMED, replace

capture drop zfood_price_BALTfinW1W3mean
egen zfood_price_BALTfinW1W3mean=std(food_price_BALTfinW1W3mean)

capture drop zDASH_scorew1w3mean
egen zDASH_scorew1w3mean=std(DASH_scorew1w3mean)

capture drop FCOSTEXP
gen FCOSTEXP=zfood_price_BALTfinW1W3mean

capture drop DASHDIETEXP
gen DASHDIETEXP=zDASH_scorew1w3mean

capture drop ALLOSTATICLOADW4
egen ALW4=std(allostatic_propw4)



save, replace
```

```
////////DUMMY VARIABLES//

**edubr: edubr2 edubr3 edubr9**
capture drop edubr2
gen edubr2=1 if edubr==2
replace edubr2=0 if edubr2~=1 & edubr~=.

capture drop edubr3
gen edubr3=1 if edubr==3
replace edubr3=0 if edubr3~=1 & edubr~=.

capture drop edubr9
gen edubr9=1 if edubr==3
replace edubr9=0 if edubr9~=1 & edubr~=.

**employed: employed1 employed9**

capture drop employed1
gen employed1=1 if employed==1
replace employed1=0 if employed1~=1 & employed~=.

capture drop employed9
gen employed9=1 if employed==1
replace employed9=0 if employed9~=1 & employed~=.

**wrattbr: wrattbr2 wrattbr3 wrattbr4**

capture drop wrattbr2
gen wrattbr2=1 if wrattbr==2
replace wrattbr2=0 if wrattbr2~=1 & wrattbr~=.

capture drop wrattbr3
gen wrattbr3=1 if wrattbr==3
replace wrattbr3=0 if wrattbr3~=1 & wrattbr~=.
```

```
capture drop wrattbr4
gen wrattbr4=1 if wrattbr==4
replace wrattbr4=0 if wrattbr4~=1 & wrattbr~=.


**smoke: smoke1 smoke9**

capture drop smoke1
gen smoke1=1 if smoke==1
replace smoke1=0 if smoke1~=1 & smoke~=.


capture drop smoke9
gen smoke9=1 if smoke==9
replace smoke9=0 if smoke9~=1 & smoke~=.


**currdrugs: currdrugs1 currdrugs9**

capture drop currdrugs1
gen currdrugs1=1 if currdrugs==1
replace currdrugs1=0 if currdrugs1~=1 & currdrugs~=.


capture drop currdrugs9
gen currdrugs9=1 if currdrugs==9
replace currdrugs9=0 if currdrugs9~=1 & currdrugs~=.

**SRHbr: SRHbr2 SRHbr3**
capture drop SRHbr2
gen SRHbr2=1 if SRHbr==2
replace SRHbr2=0 if SRHbr2~=1 & SRHbr~=.


capture drop SRHbr3
gen SRHbr3=1 if SRHbr==3
replace SRHbr3=0 if SRHbr3~=1 & SRHbr~=.


save, replace
```

```
////////////////////////////////////////////////////////////////////////ALLOSTATIC LOAD, WAVE 4///////////////////////////////////////

//ALL SAMPLE//

use HANDLS_Allostaticload_dietfcfinal_PARAMED, clear

paramed ALW4, avar(FCOSTEXP) mvar(DASHDIETEXP) cvars(Agew1_C Agew3_C sex race pir edubr2 edubr3 edubr9 employed1 employed9 wrattbr2 wrattbr3 wrattbr4 smoke1 smoke9 currdrugs1 currdrugs9 bmi_C SRHbr2 SRHbr3 kcalw1w3mean_C energystoresw1w3mean_C invmillssem)    yreg(linear) mreg(linear) a0(0) a1(1) m(0)

//MEN//
use HANDLS_Allostaticload_dietfcfinal_PARAMED,clear
keep if sex==1
save HANDLS_Allostaticload_dietfcfinal_PARAMEDMEN, replace

paramed ALW4, avar(FCOSTEXP) mvar(DASHDIETEXP) cvars(Agew1_C Agew3_C sex race pir edubr2 edubr3 edubr9 employed1 employed9 wrattbr2 wrattbr3 wrattbr4 smoke1 smoke9 currdrugs1 currdrugs9 bmi_C SRHbr2 SRHbr3 kcalw1w3mean_C energystoresw1w3mean_C invmillssem)    yreg(linear) mreg(linear) a0(0) a1(1) m(0)

//WOMEN//
use HANDLS_Allostaticload_dietfcfinal_PARAMED,clear
keep if sex==0
save HANDLS_Allostaticload_dietfcfinal_PARAMEDWOMEN, replace

paramed ALW4, avar(FCOSTEXP) mvar(DASHDIETEXP) cvars(Agew1_C Agew3_C sex race pir edubr2 edubr3 edubr9 employed1 employed9 wrattbr2 wrattbr3 wrattbr4 smoke1 smoke9 currdrugs1 currdrugs9 bmi_C SRHbr2 SRHbr3 kcalw1w3mean_C energystoresw1w3mean_C invmillssem)    yreg(linear) mreg(linear) a0(0) a1(1) m(0)

//WHITES//
use HANDLS_Allostaticload_dietfcfinal_PARAMED,clear
keep if race==0
save HANDLS_Allostaticload_dietfcfinal_PARAMEDWHITES, replace

paramed ALW4, avar(FCOSTEXP) mvar(DASHDIETEXP) cvars(Agew1_C Agew3_C sex race pir edubr2 edubr3 edubr9 employed1 employed9 wrattbr2 wrattbr3 wrattbr4 smoke1 smoke9 currdrugs1 currdrugs9 bmi_C SRHbr2 SRHbr3 kcalw1w3mean_C energystoresw1w3mean_C invmillssem)    yreg(linear) mreg(linear) a0(0) a1(1) m(0)

//African-Americans//
use HANDLS_Allostaticload_dietfcfinal_PARAMED,clear
keep if race==1
save HANDLS_Allostaticload_dietfcfinal_PARAMEDAA, replace
```

paramed ALW4, avar(FCOSTEXP) mvar(DASHDIETEXP) cvars(Agew1_C Agew3_C sex race pir edubr2 edubr3 edubr9 employed1 employed9 wrattbr2 wrattbr3 wrattbr4 smoke1 smoke9 currdrugs1 currdrugs9 bmi_C SRHbr2 SRHbr3 kcalw1w3mean_C energystoresw1w3mean_C invmillssem) yreg(linear) mreg(linear) a0(0) a1(1) m(0)

//BELOW POVERTY//

use HANDLS_Allostaticload_dietfcfinal_PARAMED,clear
keep if pir==0
save HANDLS_Allostaticload_dietfcfinal_PARAMEDBP, replace

paramed ALW4, avar(FCOSTEXP) mvar(DASHDIETEXP) cvars(Agew1_C Agew3_C sex race pir edubr2 edubr3 edubr9 employed1 employed9 wrattbr2 wrattbr3 wrattbr4 smoke1 smoke9 currdrugs1 currdrugs9 bmi_C SRHbr2 SRHbr3 kcalw1w3mean_C energystoresw1w3mean_C invmillssem) yreg(linear) mreg(linear) a0(0) a1(1) m(0)

//ABOVE POVERTY//

use HANDLS_Allostaticload_dietfcfinal_PARAMED,clear
keep if pir==1
save HANDLS_Allostaticload_dietfcfinal_PARAMEDAP, replace

paramed ALW4, avar(FCOSTEXP) mvar(DASHDIETEXP) cvars(Agew1_C Agew3_C sex race pir edubr2 edubr3 edubr9 employed1 employed9 wrattbr2 wrattbr3 wrattbr4 smoke1 smoke9 currdrugs1 currdrugs9 bmi_C SRHbr2 SRHbr3 kcalw1w3mean_C energystoresw1w3mean_C invmillssem) yreg(linear) mreg(linear) a0(0) a1(1) m(0)

save, replace

//ALLOSTATIC LOAD, WAVE 4; SATURATED FAT//////////////////////////////////////

//ALL SAMPLE//

use HANDLS_Allostaticload_dietfcfinal_PARAMED, clear

paramed ALW4, avar(FCOSTEXP) mvar(zDASH_SatFatw1w3mean) cvars(Agew1_C Agew3_C sex race pir edubr2 edubr3 edubr9 employed1 employed9 wrattbr2 wrattbr3 wrattbr4 smoke1 smoke9 currdrugs1 currdrugs9 bmi_C SRHbr2 SRHbr3 kcalw1w3mean_C energystoresw1w3mean_C invmillssem) yreg(linear) mreg(linear) a0(0) a1(1) m(0) nointer

//MEN//
use HANDLS_Allostaticload_dietfcfinal_PARAMED,clear
keep if sex==1
save HANDLS_Allostaticload_dietfcfinal_PARAMEDMEN, replace

```
paramed ALW4, avar(FCOSTEXP) mvar(zDASH_SatFatw1w3mean) cvars(Agew1_C Agew3_C sex race pir edubr2 edubr3 edubr9 employed1 employed9 wrattbr2 wrattbr3 wrattbr4 smoke1 smoke9 currdrugs1 currdrugs9 bmi_C SRHbr2 SRHbr3 kcalw1w3mean_C energystoresw1w3mean_C invmillssem)    yreg(linear) mreg(linear) a0(0) a1(1) m(0) nointer



//WOMEN//
use HANDLS_Allostaticload_dietfcfinal_PARAMED,clear
keep if sex==0
save HANDLS_Allostaticload_dietfcfinal_PARAMEDWOMEN, replace

paramed ALW4, avar(FCOSTEXP) mvar(zDASH_SatFatw1w3mean) cvars(Agew1_C Agew3_C sex race pir edubr2 edubr3 edubr9 employed1 employed9 wrattbr2 wrattbr3 wrattbr4 smoke1 smoke9 currdrugs1 currdrugs9 bmi_C SRHbr2 SRHbr3 kcalw1w3mean_C energystoresw1w3mean_C invmillssem)    yreg(linear) mreg(linear) a0(0) a1(1) m(0) nointer

//WHITES//
use HANDLS_Allostaticload_dietfcfinal_PARAMED,clear
keep if race==0
save HANDLS_Allostaticload_dietfcfinal_PARAMEDWHITES, replace

paramed ALW4, avar(FCOSTEXP) mvar(zDASH_SatFatw1w3mean) cvars(Agew1_C Agew3_C sex race pir edubr2 edubr3 edubr9 employed1 employed9 wrattbr2 wrattbr3 wrattbr4 smoke1 smoke9 currdrugs1 currdrugs9 bmi_C SRHbr2 SRHbr3 kcalw1w3mean_C energystoresw1w3mean_C invmillssem)    yreg(linear) mreg(linear) a0(0) a1(1) m(0) nointer



//African-Americans//
use HANDLS_Allostaticload_dietfcfinal_PARAMED,clear
keep if race==1
save HANDLS_Allostaticload_dietfcfinal_PARAMEDAA, replace

paramed ALW4, avar(FCOSTEXP) mvar(zDASH_SatFatw1w3mean) cvars(Agew1_C Agew3_C sex race pir edubr2 edubr3 edubr9 employed1 employed9 wrattbr2 wrattbr3 wrattbr4 smoke1 smoke9 currdrugs1 currdrugs9 bmi_C SRHbr2 SRHbr3 kcalw1w3mean_C energystoresw1w3mean_C invmillssem)    yreg(linear) mreg(linear) a0(0) a1(1) m(0) nointer

//BELOW POVERTY//

use HANDLS_Allostaticload_dietfcfinal_PARAMED,clear
keep if pir==0
save HANDLS_Allostaticload_dietfcfinal_PARAMEDBP, replace

paramed ALW4, avar(FCOSTEXP) mvar(zDASH_SatFatw1w3mean) cvars(Agew1_C Agew3_C sex race pir edubr2 edubr3 edubr9 employed1 employed9 wrattbr2 wrattbr3 wrattbr4 smoke1 smoke9 currdrugs1 currdrugs9 bmi_C SRHbr2 SRHbr3 kcalw1w3mean_C energystoresw1w3mean_C invmillssem)    yreg(linear) mreg(linear) a0(0) a1(1) m(0) nointer

//ABOVE POVERTY//
```

```
use HANDLS_Allostaticload_dietfcfinal_PARAMED,clear
keep if pir==1
save HANDLS_Allostaticload_dietfcfinal_PARAMEDAP, replace

paramed ALW4, avar(FCOSTEXP) mvar(zDASH_SatFatw1w3mean) cvars(Agew1_C Agew3_C sex race pir edubr2 edubr3 edubr9 employed1 employed9 wrattbr2 wrattbr3 wrattbr4 smoke1 smoke9 currdrugs1 currdrugs9 bmi_C SRHbr2 SRHbr3 kcalw1w3mean_C energystoresw1w3mean_C invmillssem)    yreg(linear) mreg(linear) a0(0) a1(1) m(0) nointer

save, replace


////////////////////////////////////////////////////////////////////////ALLOSTATIC LOAD, WAVE 4; FAT/////////////////////////////////////////

//ALL SAMPLE//

use HANDLS_Allostaticload_dietfcfinal_PARAMED, clear

paramed ALW4, avar(FCOSTEXP) mvar(zDASH_Fatw1w3mean) cvars(Agew1_C Agew3_C sex race pir edubr2 edubr3 edubr9 employed1 employed9 wrattbr2 wrattbr3 wrattbr4 smoke1 smoke9 currdrugs1 currdrugs9 bmi_C SRHbr2 SRHbr3 kcalw1w3mean_C energystoresw1w3mean_C invmillssem)    yreg(linear) mreg(linear) a0(0) a1(1) m(0) nointer

//MEN//
use HANDLS_Allostaticload_dietfcfinal_PARAMED,clear
keep if sex==1
save HANDLS_Allostaticload_dietfcfinal_PARAMEDMEN, replace

paramed ALW4, avar(FCOSTEXP) mvar(zDASH_Fatw1w3mean) cvars(Agew1_C Agew3_C sex race pir edubr2 edubr3 edubr9 employed1 employed9 wrattbr2 wrattbr3 wrattbr4 smoke1 smoke9 currdrugs1 currdrugs9 bmi_C SRHbr2 SRHbr3 kcalw1w3mean_C energystoresw1w3mean_C invmillssem)    yreg(linear) mreg(linear) a0(0) a1(1) m(0) nointer


//WOMEN//
use HANDLS_Allostaticload_dietfcfinal_PARAMED,clear
keep if sex==0
save HANDLS_Allostaticload_dietfcfinal_PARAMEDWOMEN, replace

paramed ALW4, avar(FCOSTEXP) mvar(zDASH_Fatw1w3mean) cvars(Agew1_C Agew3_C sex race pir edubr2 edubr3 edubr9 employed1 employed9 wrattbr2 wrattbr3 wrattbr4 smoke1 smoke9 currdrugs1 currdrugs9 bmi_C SRHbr2 SRHbr3 kcalw1w3mean_C energystoresw1w3mean_C invmillssem)    yreg(linear) mreg(linear) a0(0) a1(1) m(0) nointer

//WHITES//
use HANDLS_Allostaticload_dietfcfinal_PARAMED,clear
```

```
keep if race==0
save HANDLS_Allostaticload_dietfcfinal_PARAMEDWHITES, replace

paramed ALW4, avar(FCOSTEXP) mvar(zDASH_Fatw1w3mean) cvars(Agew1_C Agew3_C sex race pir edubr2 edubr3 edubr9 employed1 employed9 wrattbr2 wrattbr3 wrattbr4 smoke1 smoke9 currdrugs1 currdrugs9 bmi_C SRHbr2 SRHbr3 kcalw1w3mean_C energystoresw1w3mean_C invmillssem)    yreg(linear) mreg(linear) a0(0) a1(1) m(0) nointer


//African-Americans//
use HANDLS_Allostaticload_dietfcfinal_PARAMED,clear
keep if race==1
save HANDLS_Allostaticload_dietfcfinal_PARAMEDAA, replace

paramed ALW4, avar(FCOSTEXP) mvar(zDASH_Fatw1w3mean) cvars(Agew1_C Agew3_C sex race pir edubr2 edubr3 edubr9 employed1 employed9 wrattbr2 wrattbr3 wrattbr4 smoke1 smoke9 currdrugs1 currdrugs9 bmi_C SRHbr2 SRHbr3 kcalw1w3mean_C energystoresw1w3mean_C invmillssem)    yreg(linear) mreg(linear) a0(0) a1(1) m(0) nointer

//BELOW POVERTY//

use HANDLS_Allostaticload_dietfcfinal_PARAMED,clear
keep if pir==0
save HANDLS_Allostaticload_dietfcfinal_PARAMEDBP, replace

paramed ALW4, avar(FCOSTEXP) mvar(zDASH_Fatw1w3mean) cvars(Agew1_C Agew3_C sex race pir edubr2 edubr3 edubr9 employed1 employed9 wrattbr2 wrattbr3 wrattbr4 smoke1 smoke9 currdrugs1 currdrugs9 bmi_C SRHbr2 SRHbr3 kcalw1w3mean_C energystoresw1w3mean_C invmillssem)    yreg(linear) mreg(linear) a0(0) a1(1) m(0) nointer

//ABOVE POVERTY//

use HANDLS_Allostaticload_dietfcfinal_PARAMED,clear
keep if pir==1
save HANDLS_Allostaticload_dietfcfinal_PARAMEDAP, replace

paramed ALW4, avar(FCOSTEXP) mvar(zDASH_Fatw1w3mean) cvars(Agew1_C Agew3_C sex race pir edubr2 edubr3 edubr9 employed1 employed9 wrattbr2 wrattbr3 wrattbr4 smoke1 smoke9 currdrugs1 currdrugs9 bmi_C SRHbr2 SRHbr3 kcalw1w3mean_C energystoresw1w3mean_C invmillssem)    yreg(linear) mreg(linear) a0(0) a1(1) m(0) nointer

save, replace


//////////////////////////////////////////////////////////////////////ALLOSTATIC LOAD, WAVE 4; PROTEIN//////////////////////////////////////

//ALL SAMPLE//
```

```
use HANDLS_Allostaticload_dietfcfinal_PARAMED, clear

capture drop FCOSTEXP_X_* _zDash_protein_W1W3mean_*

paramed ALW4, avar(FCOSTEXP) mvar(zDash_protein_W1W3mean) cvars(Agew1_C Agew3_C sex race pir edubr2 edubr3 edubr9 employed1 employed9 wrattbr2 wrattbr3 wrattbr4 smoke1 smoke9 currdrugs1 currdrugs9 bmi_C SRHbr2 SRHbr3 kcalw1w3mean_C energystoresw1w3mean_C invmillssem)    yreg(linear) mreg(linear) a0(0) a1(1) m(0) nointer

//MEN//
use HANDLS_Allostaticload_dietfcfinal_PARAMED,clear
keep if sex==1
save HANDLS_Allostaticload_dietfcfinal_PARAMEDMEN, replace

paramed ALW4, avar(FCOSTEXP) mvar(zDash_protein_W1W3mean) cvars(Agew1_C Agew3_C sex race pir edubr2 edubr3 edubr9 employed1 employed9 wrattbr2 wrattbr3 wrattbr4 smoke1 smoke9 currdrugs1 currdrugs9 bmi_C SRHbr2 SRHbr3 kcalw1w3mean_C energystoresw1w3mean_C invmillssem)    yreg(linear) mreg(linear) a0(0) a1(1) m(0) nointer


//WOMEN//
use HANDLS_Allostaticload_dietfcfinal_PARAMED,clear
keep if sex==0
save HANDLS_Allostaticload_dietfcfinal_PARAMEDWOMEN, replace

paramed ALW4, avar(FCOSTEXP) mvar(zDash_protein_W1W3mean) cvars(Agew1_C Agew3_C sex race pir edubr2 edubr3 edubr9 employed1 employed9 wrattbr2 wrattbr3 wrattbr4 smoke1 smoke9 currdrugs1 currdrugs9 bmi_C SRHbr2 SRHbr3 kcalw1w3mean_C energystoresw1w3mean_C invmillssem)    yreg(linear) mreg(linear) a0(0) a1(1) m(0) nointer

//WHITES//
use HANDLS_Allostaticload_dietfcfinal_PARAMED,clear
keep if race==0
save HANDLS_Allostaticload_dietfcfinal_PARAMEDWHITES, replace

paramed ALW4, avar(FCOSTEXP) mvar(zDash_protein_W1W3mean) cvars(Agew1_C Agew3_C sex race pir edubr2 edubr3 edubr9 employed1 employed9 wrattbr2 wrattbr3 wrattbr4 smoke1 smoke9 currdrugs1 currdrugs9 bmi_C SRHbr2 SRHbr3 kcalw1w3mean_C energystoresw1w3mean_C invmillssem)    yreg(linear) mreg(linear) a0(0) a1(1) m(0) nointer


//African-Americans//
use HANDLS_Allostaticload_dietfcfinal_PARAMED,clear
keep if race==1
save HANDLS_Allostaticload_dietfcfinal_PARAMEDAA, replace
```

paramed ALW4, avar(FCOSTEXP) mvar(zDash_protein_W1W3mean) cvars(Agew1_C Agew3_C sex race pir edubr2 edubr3 edubr9 employed1 employed9 wrattbr2 wrattbr3 wrattbr4 smoke1 smoke9 currdrugs1 currdrugs9 bmi_C SRHbr2 SRHbr3 kcalw1w3mean_C energystoresw1w3mean_C invmillssem) yreg(linear) mreg(linear) a0(0) a1(1) m(0) nointer

//BELOW POVERTY//

use HANDLS_Allostaticload_dietfcfinal_PARAMED,clear
keep if pir==0
save HANDLS_Allostaticload_dietfcfinal_PARAMEDBP, replace

paramed ALW4, avar(FCOSTEXP) mvar(zDash_protein_W1W3mean) cvars(Agew1_C Agew3_C sex race pir edubr2 edubr3 edubr9 employed1 employed9 wrattbr2 wrattbr3 wrattbr4 smoke1 smoke9 currdrugs1 currdrugs9 bmi_C SRHbr2 SRHbr3 kcalw1w3mean_C energystoresw1w3mean_C invmillssem) yreg(linear) mreg(linear) a0(0) a1(1) m(0) nointer

//ABOVE POVERTY//

use HANDLS_Allostaticload_dietfcfinal_PARAMED,clear
keep if pir==1
save HANDLS_Allostaticload_dietfcfinal_PARAMEDAP, replace

paramed ALW4, avar(FCOSTEXP) mvar(zDash_protein_W1W3mean) cvars(Agew1_C Agew3_C sex race pir edubr2 edubr3 edubr9 employed1 employed9 wrattbr2 wrattbr3 wrattbr4 smoke1 smoke9 currdrugs1 currdrugs9 bmi_C SRHbr2 SRHbr3 kcalw1w3mean_C energystoresw1w3mean_C invmillssem) yreg(linear) mreg(linear) a0(0) a1(1) m(0) nointer

save, replace

//ALLOSTATIC LOAD, WAVE 4; CHOLESTEROL//

//ALL SAMPLE//

use HANDLS_Allostaticload_dietfcfinal_PARAMED, clear

paramed ALW4, avar(FCOSTEXP) mvar(zDASH_cholesterolw1w3) cvars(Agew1_C Agew3_C sex race pir edubr2 edubr3 edubr9 employed1 employed9 wrattbr2 wrattbr3 wrattbr4 smoke1 smoke9 currdrugs1 currdrugs9 bmi_C SRHbr2 SRHbr3 kcalw1w3mean_C energystoresw1w3mean_C invmillssem) yreg(linear) mreg(linear) a0(0) a1(1) m(0) nointer

//MEN//
use HANDLS_Allostaticload_dietfcfinal_PARAMED,clear
keep if sex==1
save HANDLS_Allostaticload_dietfcfinal_PARAMEDMEN, replace

```
paramed ALW4, avar(FCOSTEXP) mvar(zDASH_cholesterolw1w3 ) cvars(Agew1_C Agew3_C sex race pir edubr2 edubr3 edubr9 employed1 employed9 wrattbr2 wrattbr3 wrattbr4 smoke1 smoke9 currdrugs1 currdrugs9 bmi_C SRHbr2 SRHbr3 kcalw1w3mean_C energystoresw1w3mean_C invmillssem)    yreg(linear) mreg(linear) a0(0) a1(1) m(0) nointer



//WOMEN//
use HANDLS_Allostaticload_dietfcfinal_PARAMED,clear
keep if sex==0
save HANDLS_Allostaticload_dietfcfinal_PARAMEDWOMEN, replace

paramed ALW4, avar(FCOSTEXP) mvar(zDASH_cholesterolw1w3 ) cvars(Agew1_C Agew3_C sex race pir edubr2 edubr3 edubr9 employed1 employed9 wrattbr2 wrattbr3 wrattbr4 smoke1 smoke9 currdrugs1 currdrugs9 bmi_C SRHbr2 SRHbr3 kcalw1w3mean_C energystoresw1w3mean_C invmillssem)    yreg(linear) mreg(linear) a0(0) a1(1) m(0) nointer

//WHITES//
use HANDLS_Allostaticload_dietfcfinal_PARAMED,clear
keep if race==0
save HANDLS_Allostaticload_dietfcfinal_PARAMEDWHITES, replace

paramed ALW4, avar(FCOSTEXP) mvar(zDASH_cholesterolw1w3 ) cvars(Agew1_C Agew3_C sex race pir edubr2 edubr3 edubr9 employed1 employed9 wrattbr2 wrattbr3 wrattbr4 smoke1 smoke9 currdrugs1 currdrugs9 bmi_C SRHbr2 SRHbr3 kcalw1w3mean_C energystoresw1w3mean_C invmillssem)    yreg(linear) mreg(linear) a0(0) a1(1) m(0) nointer



//African-Americans//
use HANDLS_Allostaticload_dietfcfinal_PARAMED,clear
keep if race==1
save HANDLS_Allostaticload_dietfcfinal_PARAMEDAA, replace

paramed ALW4, avar(FCOSTEXP) mvar(zDASH_cholesterolw1w3 ) cvars(Agew1_C Agew3_C sex race pir edubr2 edubr3 edubr9 employed1 employed9 wrattbr2 wrattbr3 wrattbr4 smoke1 smoke9 currdrugs1 currdrugs9 bmi_C SRHbr2 SRHbr3 kcalw1w3mean_C energystoresw1w3mean_C invmillssem)    yreg(linear) mreg(linear) a0(0) a1(1) m(0) nointer

//BELOW POVERTY//

use HANDLS_Allostaticload_dietfcfinal_PARAMED,clear
keep if pir==0
save HANDLS_Allostaticload_dietfcfinal_PARAMEDBP, replace

paramed ALW4, avar(FCOSTEXP) mvar(zDASH_cholesterolw1w3 ) cvars(Agew1_C Agew3_C sex race pir edubr2 edubr3 edubr9 employed1 employed9 wrattbr2 wrattbr3 wrattbr4 smoke1 smoke9 currdrugs1 currdrugs9 bmi_C SRHbr2 SRHbr3 kcalw1w3mean_C energystoresw1w3mean_C invmillssem)    yreg(linear) mreg(linear) a0(0) a1(1) m(0) nointer

//ABOVE POVERTY//
```

```
use HANDLS_Allostaticload_dietfcfinal_PARAMED,clear
keep if pir==1
save HANDLS_Allostaticload_dietfcfinal_PARAMEDAP, replace

paramed ALW4, avar(FCOSTEXP) mvar(zDASH_cholesterolw1w3 ) cvars(Agew1_C Agew3_C sex race pir edubr2 edubr3 edubr9 employed1 employed9 wrattbr2 wrattbr3 wrattbr4 smoke1 smoke9 currdrugs1 currdrugs9 bmi_C SRHbr2 SRHbr3 kcalw1w3mean_C energystoresw1w3mean_C invmillssem)    yreg(linear) mreg(linear) a0(0) a1(1) m(0) nointer

save, replace

//////////////////////////////////////////////////////////////////////ALLOSTATIC LOAD, WAVE 4; FIBER////////////////////////////////////////

//ALL SAMPLE//

use HANDLS_Allostaticload_dietfcfinal_PARAMED, clear

paramed ALW4, avar(FCOSTEXP) mvar(zDASH_fiberw1w3 ) cvars(Agew1_C Agew3_C sex race pir edubr2 edubr3 edubr9 employed1 employed9 wrattbr2 wrattbr3 wrattbr4 smoke1 smoke9 currdrugs1 currdrugs9 bmi_C SRHbr2 SRHbr3 kcalw1w3mean_C energystoresw1w3mean_C invmillssem)    yreg(linear) mreg(linear) a0(0) a1(1) m(0) nointer

//MEN//
use HANDLS_Allostaticload_dietfcfinal_PARAMED,clear
keep if sex==1
save HANDLS_Allostaticload_dietfcfinal_PARAMEDMEN, replace

paramed ALW4, avar(FCOSTEXP) mvar(zDASH_fiberw1w3 ) cvars(Agew1_C Agew3_C sex race pir edubr2 edubr3 edubr9 employed1 employed9 wrattbr2 wrattbr3 wrattbr4 smoke1 smoke9 currdrugs1 currdrugs9 bmi_C SRHbr2 SRHbr3 kcalw1w3mean_C energystoresw1w3mean_C invmillssem)    yreg(linear) mreg(linear) a0(0) a1(1) m(0) nointer


//WOMEN//
use HANDLS_Allostaticload_dietfcfinal_PARAMED,clear
keep if sex==0
save HANDLS_Allostaticload_dietfcfinal_PARAMEDWOMEN, replace

paramed ALW4, avar(FCOSTEXP) mvar(zDASH_fiberw1w3 ) cvars(Agew1_C Agew3_C sex race pir edubr2 edubr3 edubr9 employed1 employed9 wrattbr2 wrattbr3 wrattbr4 smoke1 smoke9 currdrugs1 currdrugs9 bmi_C SRHbr2 SRHbr3 kcalw1w3mean_C energystoresw1w3mean_C invmillssem)    yreg(linear) mreg(linear) a0(0) a1(1) m(0) nointer

//WHITES//
use HANDLS_Allostaticload_dietfcfinal_PARAMED,clear
keep if race==0
save HANDLS_Allostaticload_dietfcfinal_PARAMEDWHITES, replace
```

paramed ALW4, avar(FCOSTEXP) mvar(zDASH_fiberw1w3) cvars(Agew1_C Agew3_C sex race pir edubr2 edubr3 edubr9 employed1 employed9 wrattbr2 wrattbr3 wrattbr4 smoke1 smoke9 currdrugs1 currdrugs9 bmi_C SRHbr2 SRHbr3 kcalw1w3mean_C energystoresw1w3mean_C invmillssem) yreg(linear) mreg(linear) a0(0) a1(1) m(0) nointer

//African-Americans//
use HANDLS_Allostaticload_dietfcfinal_PARAMED,clear
keep if race==1
save HANDLS_Allostaticload_dietfcfinal_PARAMEDAA, replace

paramed ALW4, avar(FCOSTEXP) mvar(zDASH_fiberw1w3) cvars(Agew1_C Agew3_C sex race pir edubr2 edubr3 edubr9 employed1 employed9 wrattbr2 wrattbr3 wrattbr4 smoke1 smoke9 currdrugs1 currdrugs9 bmi_C SRHbr2 SRHbr3 kcalw1w3mean_C energystoresw1w3mean_C invmillssem) yreg(linear) mreg(linear) a0(0) a1(1) m(0) nointer

//BELOW POVERTY//

use HANDLS_Allostaticload_dietfcfinal_PARAMED,clear
keep if pir==0
save HANDLS_Allostaticload_dietfcfinal_PARAMEDBP, replace

paramed ALW4, avar(FCOSTEXP) mvar(zDASH_fiberw1w3) cvars(Agew1_C Agew3_C sex race pir edubr2 edubr3 edubr9 employed1 employed9 wrattbr2 wrattbr3 wrattbr4 smoke1 smoke9 currdrugs1 currdrugs9 bmi_C SRHbr2 SRHbr3 kcalw1w3mean_C energystoresw1w3mean_C invmillssem) yreg(linear) mreg(linear) a0(0) a1(1) m(0) nointer

//ABOVE POVERTY//

use HANDLS_Allostaticload_dietfcfinal_PARAMED,clear
keep if pir==1
save HANDLS_Allostaticload_dietfcfinal_PARAMEDAP, replace

paramed ALW4, avar(FCOSTEXP) mvar(zDASH_fiberw1w3) cvars(Agew1_C Agew3_C sex race pir edubr2 edubr3 edubr9 employed1 employed9 wrattbr2 wrattbr3 wrattbr4 smoke1 smoke9 currdrugs1 currdrugs9 bmi_C SRHbr2 SRHbr3 kcalw1w3mean_C energystoresw1w3mean_C invmillssem) yreg(linear) mreg(linear) a0(0) a1(1) m(0) nointer

save, replace

//ALLOSTATIC LOAD, WAVE 4; MAGNESIUM//////////////////////////////////////

//ALL SAMPLE//

```
use HANDLS_Allostaticload_dietfcfinal_PARAMED, clear

paramed ALW4, avar(FCOSTEXP) mvar(zDASH_Magnesiumw1w3 ) cvars(Agew1_C Agew3_C sex race pir edubr2 edubr3
edubr9 employed1 employed9 wrattbr2 wrattbr3 wrattbr4 smoke1 smoke9 currdrugs1 currdrugs9 bmi_C SRHbr2 SRHbr3
kcalw1w3mean_C energystoresw1w3mean_C invmillssem)    yreg(linear) mreg(linear) a0(0) a1(1) m(0) nointer

//MEN//
use HANDLS_Allostaticload_dietfcfinal_PARAMED,clear
keep if sex==1
save HANDLS_Allostaticload_dietfcfinal_PARAMEDMEN, replace

paramed ALW4, avar(FCOSTEXP) mvar(zDASH_Magnesiumw1w3 ) cvars(Agew1_C Agew3_C sex race pir edubr2 edubr3
edubr9 employed1 employed9 wrattbr2 wrattbr3 wrattbr4 smoke1 smoke9 currdrugs1 currdrugs9 bmi_C SRHbr2 SRHbr3
kcalw1w3mean_C energystoresw1w3mean_C invmillssem)    yreg(linear) mreg(linear) a0(0) a1(1) m(0) nointer

//WOMEN//
use HANDLS_Allostaticload_dietfcfinal_PARAMED,clear
keep if sex==0
save HANDLS_Allostaticload_dietfcfinal_PARAMEDWOMEN, replace

paramed ALW4, avar(FCOSTEXP) mvar(zDASH_Magnesiumw1w3 ) cvars(Agew1_C Agew3_C sex race pir edubr2 edubr3
edubr9 employed1 employed9 wrattbr2 wrattbr3 wrattbr4 smoke1 smoke9 currdrugs1 currdrugs9 bmi_C SRHbr2 SRHbr3
kcalw1w3mean_C energystoresw1w3mean_C invmillssem)    yreg(linear) mreg(linear) a0(0) a1(1) m(0) nointer

//WHITES//
use HANDLS_Allostaticload_dietfcfinal_PARAMED,clear
keep if race==0
save HANDLS_Allostaticload_dietfcfinal_PARAMEDWHITES, replace

paramed ALW4, avar(FCOSTEXP) mvar(zDASH_Magnesiumw1w3 ) cvars(Agew1_C Agew3_C sex race pir edubr2 edubr3
edubr9 employed1 employed9 wrattbr2 wrattbr3 wrattbr4 smoke1 smoke9 currdrugs1 currdrugs9 bmi_C SRHbr2 SRHbr3
kcalw1w3mean_C energystoresw1w3mean_C invmillssem)    yreg(linear) mreg(linear) a0(0) a1(1) m(0) nointer

//African-Americans//
use HANDLS_Allostaticload_dietfcfinal_PARAMED,clear
keep if race==1
save HANDLS_Allostaticload_dietfcfinal_PARAMEDAA, replace

paramed ALW4, avar(FCOSTEXP) mvar(zDASH_Magnesiumw1w3 ) cvars(Agew1_C Agew3_C sex race pir edubr2 edubr3
edubr9 employed1 employed9 wrattbr2 wrattbr3 wrattbr4 smoke1 smoke9 currdrugs1 currdrugs9 bmi_C SRHbr2 SRHbr3
kcalw1w3mean_C energystoresw1w3mean_C invmillssem)    yreg(linear) mreg(linear) a0(0) a1(1) m(0) nointer
```

//BELOW POVERTY//

use HANDLS_Allostaticload_dietfcfinal_PARAMED,clear
keep if pir==0
save HANDLS_Allostaticload_dietfcfinal_PARAMEDBP, replace

paramed ALW4, avar(FCOSTEXP) mvar(zDASH_Magnesiumw1w3) cvars(Agew1_C Agew3_C sex race pir edubr2 edubr3 edubr9 employed1 employed9 wrattbr2 wrattbr3 wrattbr4 smoke1 smoke9 currdrugs1 currdrugs9 bmi_C SRHbr2 SRHbr3 kcalw1w3mean_C energystoresw1w3mean_C invmillssem)   yreg(linear) mreg(linear) a0(0) a1(1) m(0) nointer

//ABOVE POVERTY//

use HANDLS_Allostaticload_dietfcfinal_PARAMED,clear
keep if pir==1
save HANDLS_Allostaticload_dietfcfinal_PARAMEDAP, replace

paramed ALW4, avar(FCOSTEXP) mvar(zDASH_Magnesiumw1w3) cvars(Agew1_C Agew3_C sex race pir edubr2 edubr3 edubr9 employed1 employed9 wrattbr2 wrattbr3 wrattbr4 smoke1 smoke9 currdrugs1 currdrugs9 bmi_C SRHbr2 SRHbr3 kcalw1w3mean_C energystoresw1w3mean_C invmillssem)   yreg(linear) mreg(linear) a0(0) a1(1) m(0) nointer

save, replace

//ALLOSTATIC LOAD, WAVE 4; calcium//////////////////////////////////////

//ALL SAMPLE//

use HANDLS_Allostaticload_dietfcfinal_PARAMED, clear

paramed ALW4, avar(FCOSTEXP) mvar(zDASH_calciumw1w3) cvars(Agew1_C Agew3_C sex race pir edubr2 edubr3 edubr9 employed1 employed9 wrattbr2 wrattbr3 wrattbr4 smoke1 smoke9 currdrugs1 currdrugs9 bmi_C SRHbr2 SRHbr3 kcalw1w3mean_C energystoresw1w3mean_C invmillssem)   yreg(linear) mreg(linear) a0(0) a1(1) m(0) nointer

//MEN//
use HANDLS_Allostaticload_dietfcfinal_PARAMED,clear
keep if sex==1
save HANDLS_Allostaticload_dietfcfinal_PARAMEDMEN, replace

paramed ALW4, avar(FCOSTEXP) mvar(zDASH_calciumw1w3) cvars(Agew1_C Agew3_C sex race pir edubr2 edubr3 edubr9 employed1 employed9 wrattbr2 wrattbr3 wrattbr4 smoke1 smoke9 currdrugs1 currdrugs9 bmi_C SRHbr2 SRHbr3 kcalw1w3mean_C energystoresw1w3mean_C invmillssem)   yreg(linear) mreg(linear) a0(0) a1(1) m(0) nointer

//WOMEN//

```
use HANDLS_Allostaticload_dietfcfinal_PARAMED,clear
keep if sex==0
save HANDLS_Allostaticload_dietfcfinal_PARAMEDWOMEN, replace

paramed ALW4, avar(FCOSTEXP) mvar(zDASH_calciumw1w3 ) cvars(Agew1_C Agew3_C sex race pir edubr2 edubr3 edubr9 employed1 employed9 wrattbr2 wrattbr3 wrattbr4 smoke1 smoke9 currdrugs1 currdrugs9 bmi_C SRHbr2 SRHbr3 kcalw1w3mean_C energystoresw1w3mean_C invmillssem)    yreg(linear) mreg(linear) a0(0) a1(1) m(0) nointer

//WHITES//
use HANDLS_Allostaticload_dietfcfinal_PARAMED,clear
keep if race==0
save HANDLS_Allostaticload_dietfcfinal_PARAMEDWHITES, replace

paramed ALW4, avar(FCOSTEXP) mvar(zDASH_calciumw1w3 ) cvars(Agew1_C Agew3_C sex race pir edubr2 edubr3 edubr9 employed1 employed9 wrattbr2 wrattbr3 wrattbr4 smoke1 smoke9 currdrugs1 currdrugs9 bmi_C SRHbr2 SRHbr3 kcalw1w3mean_C energystoresw1w3mean_C invmillssem)    yreg(linear) mreg(linear) a0(0) a1(1) m(0) nointer


//African-Americans//
use HANDLS_Allostaticload_dietfcfinal_PARAMED,clear
keep if race==1
save HANDLS_Allostaticload_dietfcfinal_PARAMEDAA, replace

paramed ALW4, avar(FCOSTEXP) mvar(zDASH_calciumw1w3 ) cvars(Agew1_C Agew3_C sex race pir edubr2 edubr3 edubr9 employed1 employed9 wrattbr2 wrattbr3 wrattbr4 smoke1 smoke9 currdrugs1 currdrugs9 bmi_C SRHbr2 SRHbr3 kcalw1w3mean_C energystoresw1w3mean_C invmillssem)    yreg(linear) mreg(linear) a0(0) a1(1) m(0) nointer

//BELOW POVERTY//

use HANDLS_Allostaticload_dietfcfinal_PARAMED,clear
keep if pir==0
save HANDLS_Allostaticload_dietfcfinal_PARAMEDBP, replace

paramed ALW4, avar(FCOSTEXP) mvar(zDASH_calciumw1w3 ) cvars(Agew1_C Agew3_C sex race pir edubr2 edubr3 edubr9 employed1 employed9 wrattbr2 wrattbr3 wrattbr4 smoke1 smoke9 currdrugs1 currdrugs9 bmi_C SRHbr2 SRHbr3 kcalw1w3mean_C energystoresw1w3mean_C invmillssem)    yreg(linear) mreg(linear) a0(0) a1(1) m(0) nointer

//ABOVE POVERTY//

use HANDLS_Allostaticload_dietfcfinal_PARAMED,clear
keep if pir==1
save HANDLS_Allostaticload_dietfcfinal_PARAMEDAP, replace
```

```
paramed ALW4, avar(FCOSTEXP) mvar(zDASH_calciumw1w3 ) cvars(Agew1_C Agew3_C sex race pir edubr2 edubr3 edubr9 employed1 employed9 wrattbr2 wrattbr3 wrattbr4 smoke1 smoke9 currdrugs1 currdrugs9 bmi_C SRHbr2 SRHbr3 kcalw1w3mean_C energystoresw1w3mean_C invmillssem)    yreg(linear) mreg(linear) a0(0) a1(1) m(0) nointer

save, replace

////////////////////////////////////////////////////////////////////////ALLOSTATIC LOAD, WAVE 4; Potassium/////////////////////////////////////////

//ALL SAMPLE//

use HANDLS_Allostaticload_dietfcfinal_PARAMED, clear

paramed ALW4, avar(FCOSTEXP) mvar(zDASH_potassiumw1w3 ) cvars(Agew1_C Agew3_C sex race pir edubr2 edubr3 edubr9 employed1 employed9 wrattbr2 wrattbr3 wrattbr4 smoke1 smoke9 currdrugs1 currdrugs9 bmi_C SRHbr2 SRHbr3 kcalw1w3mean_C energystoresw1w3mean_C invmillssem)    yreg(linear) mreg(linear) a0(0) a1(1) m(0) nointer

//MEN//
use HANDLS_Allostaticload_dietfcfinal_PARAMED,clear
keep if sex==1
save HANDLS_Allostaticload_dietfcfinal_PARAMEDMEN, replace

paramed ALW4, avar(FCOSTEXP) mvar(zDASH_potassiumw1w3 ) cvars(Agew1_C Agew3_C sex race pir edubr2 edubr3 edubr9 employed1 employed9 wrattbr2 wrattbr3 wrattbr4 smoke1 smoke9 currdrugs1 currdrugs9 bmi_C SRHbr2 SRHbr3 kcalw1w3mean_C energystoresw1w3mean_C invmillssem)    yreg(linear) mreg(linear) a0(0) a1(1) m(0) nointer


//WOMEN//
use HANDLS_Allostaticload_dietfcfinal_PARAMED,clear
keep if sex==0
save HANDLS_Allostaticload_dietfcfinal_PARAMEDWOMEN, replace

paramed ALW4, avar(FCOSTEXP) mvar(zDASH_potassiumw1w3 ) cvars(Agew1_C Agew3_C sex race pir edubr2 edubr3 edubr9 employed1 employed9 wrattbr2 wrattbr3 wrattbr4 smoke1 smoke9 currdrugs1 currdrugs9 bmi_C SRHbr2 SRHbr3 kcalw1w3mean_C energystoresw1w3mean_C invmillssem)    yreg(linear) mreg(linear) a0(0) a1(1) m(0) nointer

//WHITES//
use HANDLS_Allostaticload_dietfcfinal_PARAMED,clear
keep if race==0
save HANDLS_Allostaticload_dietfcfinal_PARAMEDWHITES, replace

paramed ALW4, avar(FCOSTEXP) mvar(zDASH_potassiumw1w3 ) cvars(Agew1_C Agew3_C sex race pir edubr2 edubr3 edubr9 employed1 employed9 wrattbr2 wrattbr3 wrattbr4 smoke1 smoke9 currdrugs1 currdrugs9 bmi_C SRHbr2 SRHbr3 kcalw1w3mean_C energystoresw1w3mean_C invmillssem)    yreg(linear) mreg(linear) a0(0) a1(1) m(0) nointer
```

```
//African-Americans//
use HANDLS_Allostaticload_dietfcfinal_PARAMED,clear
keep if race==1
save HANDLS_Allostaticload_dietfcfinal_PARAMEDAA, replace

paramed ALW4, avar(FCOSTEXP) mvar(zDASH_potassiumw1w3 ) cvars(Agew1_C Agew3_C sex race pir edubr2 edubr3 edubr9 employed1 employed9 wrattbr2 wrattbr3 wrattbr4 smoke1 smoke9 currdrugs1 currdrugs9 bmi_C SRHbr2 SRHbr3 kcalw1w3mean_C energystoresw1w3mean_C invmillssem)    yreg(linear) mreg(linear) a0(0) a1(1) m(0) nointer

//BELOW POVERTY//

use HANDLS_Allostaticload_dietfcfinal_PARAMED,clear
keep if pir==0
save HANDLS_Allostaticload_dietfcfinal_PARAMEDBP, replace

paramed ALW4, avar(FCOSTEXP) mvar(zDASH_potassiumw1w3 ) cvars(Agew1_C Agew3_C sex race pir edubr2 edubr3 edubr9 employed1 employed9 wrattbr2 wrattbr3 wrattbr4 smoke1 smoke9 currdrugs1 currdrugs9 bmi_C SRHbr2 SRHbr3 kcalw1w3mean_C energystoresw1w3mean_C invmillssem)    yreg(linear) mreg(linear) a0(0) a1(1) m(0) nointer

//ABOVE POVERTY//

use HANDLS_Allostaticload_dietfcfinal_PARAMED,clear
keep if pir==1
save HANDLS_Allostaticload_dietfcfinal_PARAMEDAP, replace

paramed ALW4, avar(FCOSTEXP) mvar(zDASH_potassiumw1w3 ) cvars(Agew1_C Agew3_C sex race pir edubr2 edubr3 edubr9 employed1 employed9 wrattbr2 wrattbr3 wrattbr4 smoke1 smoke9 currdrugs1 currdrugs9 bmi_C SRHbr2 SRHbr3 kcalw1w3mean_C energystoresw1w3mean_C invmillssem)    yreg(linear) mreg(linear) a0(0) a1(1) m(0) nointer

save, replace

/////////////////////////////////////////////////////////////////////ALLOSTATIC LOAD, WAVE 4; SODIUM//////////////////////////////////////

//ALL SAMPLE//

use HANDLS_Allostaticload_dietfcfinal_PARAMED, clear

paramed ALW4, avar(FCOSTEXP) mvar(zDASH_Sodiumw1w3 ) cvars(Agew1_C Agew3_C sex race pir edubr2 edubr3 edubr9 employed1 employed9 wrattbr2 wrattbr3 wrattbr4 smoke1 smoke9 currdrugs1 currdrugs9 bmi_C SRHbr2 SRHbr3 kcalw1w3mean_C energystoresw1w3mean_C invmillssem)    yreg(linear) mreg(linear) a0(0) a1(1) m(0) nointer

//MEN//
use HANDLS_Allostaticload_dietfcfinal_PARAMED,clear
```

```
keep if sex==1
save HANDLS_Allostaticload_dietfcfinal_PARAMEDMEN, replace

paramed ALW4, avar(FCOSTEXP) mvar(zDASH_Sodiumw1w3 ) cvars(Agew1_C Agew3_C sex race pir edubr2 edubr3 edubr9 employed1 employed9 wrattbr2 wrattbr3 wrattbr4 smoke1 smoke9 currdrugs1 currdrugs9 bmi_C SRHbr2 SRHbr3 kcalw1w3mean_C energystoresw1w3mean_C invmillssem)    yreg(linear) mreg(linear) a0(0) a1(1) m(0) nointer



//WOMEN//
use HANDLS_Allostaticload_dietfcfinal_PARAMED,clear
keep if sex==0
save HANDLS_Allostaticload_dietfcfinal_PARAMEDWOMEN, replace

paramed ALW4, avar(FCOSTEXP) mvar(zDASH_Sodiumw1w3 ) cvars(Agew1_C Agew3_C sex race pir edubr2 edubr3 edubr9 employed1 employed9 wrattbr2 wrattbr3 wrattbr4 smoke1 smoke9 currdrugs1 currdrugs9 bmi_C SRHbr2 SRHbr3 kcalw1w3mean_C energystoresw1w3mean_C invmillssem)    yreg(linear) mreg(linear) a0(0) a1(1) m(0) nointer

//WHITES//
use HANDLS_Allostaticload_dietfcfinal_PARAMED,clear
keep if race==0
save HANDLS_Allostaticload_dietfcfinal_PARAMEDWHITES, replace

paramed ALW4, avar(FCOSTEXP) mvar(zDASH_Sodiumw1w3 ) cvars(Agew1_C Agew3_C sex race pir edubr2 edubr3 edubr9 employed1 employed9 wrattbr2 wrattbr3 wrattbr4 smoke1 smoke9 currdrugs1 currdrugs9 bmi_C SRHbr2 SRHbr3 kcalw1w3mean_C energystoresw1w3mean_C invmillssem)    yreg(linear) mreg(linear) a0(0) a1(1) m(0) nointer



//African-Americans//
use HANDLS_Allostaticload_dietfcfinal_PARAMED,clear
keep if race==1
save HANDLS_Allostaticload_dietfcfinal_PARAMEDAA, replace

paramed ALW4, avar(FCOSTEXP) mvar(zDASH_Sodiumw1w3 ) cvars(Agew1_C Agew3_C sex race pir edubr2 edubr3 edubr9 employed1 employed9 wrattbr2 wrattbr3 wrattbr4 smoke1 smoke9 currdrugs1 currdrugs9 bmi_C SRHbr2 SRHbr3 kcalw1w3mean_C energystoresw1w3mean_C invmillssem)    yreg(linear) mreg(linear) a0(0) a1(1) m(0) nointer

//BELOW POVERTY//

use HANDLS_Allostaticload_dietfcfinal_PARAMED,clear
keep if pir==0
save HANDLS_Allostaticload_dietfcfinal_PARAMEDBP, replace
```

```
paramed ALW4, avar(FCOSTEXP) mvar(zDASH_Sodiumw1w3 ) cvars(Agew1_C Agew3_C sex race pir edubr2 edubr3 edubr9 employed1 employed9 wrattbr2 wrattbr3 wrattbr4 smoke1 smoke9 currdrugs1 currdrugs9 bmi_C SRHbr2 SRHbr3 kcalw1w3mean_C energystoresw1w3mean_C invmillssem)    yreg(linear) mreg(linear) a0(0) a1(1) m(0) nointer

//ABOVE POVERTY//

use HANDLS_Allostaticload_dietfcfinal_PARAMED,clear
keep if pir==1
save HANDLS_Allostaticload_dietfcfinal_PARAMEDAP, replace

paramed ALW4, avar(FCOSTEXP) mvar(zDASH_Sodiumw1w3 ) cvars(Agew1_C Agew3_C sex race pir edubr2 edubr3 edubr9 employed1 employed9 wrattbr2 wrattbr3 wrattbr4 smoke1 smoke9 currdrugs1 currdrugs9 bmi_C SRHbr2 SRHbr3 kcalw1w3mean_C energystoresw1w3mean_C invmillssem)    yreg(linear) mreg(linear) a0(0) a1(1) m(0) nointer

save, replace


////////////////COMPONENTS OF AL wave 4////////////////////
cd "G:\16GBBACKUPUSB\BACKUP_USB_SEPTEMBER2014\May Baydoun_folder\HANDLS_paper30_foodcost_MetSlong\DATA"

use HANDLS_Allostaticload_dietfcfinal_PARAMED, clear

capture drop zalbuminw4
egen zalbuminw4=std(albuminw4)

capture drop zcrpw4
egen zcrpw4=std(crpw4)


capture drop zcholw4
egen zcholw4=std(cholw4)

capture drop zhdlw4
egen zhdlw4=std(hdlw4)

capture drop zhgba1cw4
egen zhgba1cw4=std(hgba1cw4)

capture drop zwhrw4
egen zwhrw4=std(whrw4)

capture drop zbpsysw4
egen zbpsysw4=std(bpsysw4)
```

```
capture drop zbpdiaw4
egen zbpdiaw4=std(bpdiaw4)

capture drop zhrw4
egen zhrw4=std(hrw4)

save, replace

**albuminw4 crpw4 cholw4 hdlw4 hgba1cw4 whrw4 bpsysw4 bpdiaw4 hrw4

////////////////////////////////////////////////////////////////////////////////STANDARDIZED ALBUMIN, WAVE 4/////////////////////////////////////////
cd "G:\16GBBACKUPUSB\BACKUP_USB_SEPTEMBER2014\May Baydoun_folder\HANDLS_paper30_foodcost_MetSlong\DATA"

use HANDLS_Allostaticload_dietfcfinal_PARAMED, clear

capture drop ALBUMINW4
gen ALBUMINW4=zalbuminw4

save, replace

//ALL SAMPLE//

use HANDLS_Allostaticload_dietfcfinal_PARAMED, clear

paramed ALBUMINW4, avar(FCOSTEXP) mvar(DASHDIETEXP) cvars(Agew1_C Agew3_C sex race pir edubr2 edubr3 edubr9 employed1 employed9 wrattbr2 wrattbr3 wrattbr4 smoke1 smoke9 currdrugs1 currdrugs9 bmi_C SRHbr2 SRHbr3 kcalw1w3mean_C energystoresw1w3mean_C invmillssem)    yreg(linear) mreg(linear) a0(0) a1(1) m(0)

//MEN//
use HANDLS_Allostaticload_dietfcfinal_PARAMED,clear
```

```
keep if sex==1
save HANDLS_Allostaticload_dietfcfinal_PARAMEDMEN, replace

paramed ALBUMINW4, avar(FCOSTEXP) mvar(DASHDIETEXP) cvars(Agew1_C Agew3_C sex race pir edubr2 edubr3 edubr9 employed1 employed9 wrattbr2 wrattbr3 wrattbr4 smoke1 smoke9 currdrugs1 currdrugs9 bmi_C SRHbr2 SRHbr3 kcalw1w3mean_C energystoresw1w3mean_C invmillssem)    yreg(linear) mreg(linear) a0(0) a1(1) m(0)



//WOMEN//
use HANDLS_Allostaticload_dietfcfinal_PARAMED,clear
keep if sex==0
save HANDLS_Allostaticload_dietfcfinal_PARAMEDWOMEN, replace

paramed ALBUMINW4, avar(FCOSTEXP) mvar(DASHDIETEXP) cvars(Agew1_C Agew3_C sex race pir edubr2 edubr3 edubr9 employed1 employed9 wrattbr2 wrattbr3 wrattbr4 smoke1 smoke9 currdrugs1 currdrugs9 bmi_C SRHbr2 SRHbr3 kcalw1w3mean_C energystoresw1w3mean_C invmillssem)    yreg(linear) mreg(linear) a0(0) a1(1) m(0)

//WHITES//
use HANDLS_Allostaticload_dietfcfinal_PARAMED,clear
keep if race==0
save HANDLS_Allostaticload_dietfcfinal_PARAMEDWHITES, replace

paramed ALBUMINW4, avar(FCOSTEXP) mvar(DASHDIETEXP) cvars(Agew1_C Agew3_C sex race pir edubr2 edubr3 edubr9 employed1 employed9 wrattbr2 wrattbr3 wrattbr4 smoke1 smoke9 currdrugs1 currdrugs9 bmi_C SRHbr2 SRHbr3 kcalw1w3mean_C energystoresw1w3mean_C invmillssem)    yreg(linear) mreg(linear) a0(0) a1(1) m(0)



//African-Americans//
use HANDLS_Allostaticload_dietfcfinal_PARAMED,clear
keep if race==1
save HANDLS_Allostaticload_dietfcfinal_PARAMEDAA, replace

paramed ALBUMINW4, avar(FCOSTEXP) mvar(DASHDIETEXP) cvars(Agew1_C Agew3_C sex race pir edubr2 edubr3 edubr9 employed1 employed9 wrattbr2 wrattbr3 wrattbr4 smoke1 smoke9 currdrugs1 currdrugs9 bmi_C SRHbr2 SRHbr3 kcalw1w3mean_C energystoresw1w3mean_C invmillssem)    yreg(linear) mreg(linear) a0(0) a1(1) m(0)

//BELOW POVERTY//

use HANDLS_Allostaticload_dietfcfinal_PARAMED,clear
keep if pir==0
save HANDLS_Allostaticload_dietfcfinal_PARAMEDBP, replace
```

```
paramed ALBUMINW4, avar(FCOSTEXP) mvar(DASHDIETEXP) cvars(Agew1_C Agew3_C sex race pir edubr2 edubr3 edubr9 employed1 employed9 wrattbr2 wrattbr3 wrattbr4 smoke1 smoke9 currdrugs1 currdrugs9 bmi_C SRHbr2 SRHbr3 kcalw1w3mean_C energystoresw1w3mean_C invmillssem)    yreg(linear) mreg(linear) a0(0) a1(1) m(0)

//ABOVE POVERTY//

use HANDLS_Allostaticload_dietfcfinal_PARAMED,clear
keep if pir==1
save HANDLS_Allostaticload_dietfcfinal_PARAMEDAP, replace

paramed ALBUMINW4, avar(FCOSTEXP) mvar(DASHDIETEXP) cvars(Agew1_C Agew3_C sex race pir edubr2 edubr3 edubr9 employed1 employed9 wrattbr2 wrattbr3 wrattbr4 smoke1 smoke9 currdrugs1 currdrugs9 bmi_C SRHbr2 SRHbr3 kcalw1w3mean_C energystoresw1w3mean_C invmillssem)    yreg(linear) mreg(linear) a0(0) a1(1) m(0)

save, replace



////////////////////////////////////////////////////////////////////////STANDARDIZED CRP, WAVE 4////////////////////////////////////////
cd "G:\16GBBACKUPUSB\BACKUP_USB_SEPTEMBER2014\May Baydoun_folder\HANDLS_paper30_foodcost_MetSlong\DATA"

use HANDLS_Allostaticload_dietfcfinal_PARAMED, clear


capture drop CRPW4
gen CRPW4=zcrpw4

save, replace

//ALL SAMPLE//

use HANDLS_Allostaticload_dietfcfinal_PARAMED, clear



paramed CRPW4, avar(FCOSTEXP) mvar(DASHDIETEXP) cvars(Agew1_C Agew3_C sex race pir edubr2 edubr3 edubr9 employed1 employed9 wrattbr2 wrattbr3 wrattbr4 smoke1 smoke9 currdrugs1 currdrugs9 bmi_C SRHbr2 SRHbr3 kcalw1w3mean_C energystoresw1w3mean_C invmillssem)    yreg(linear) mreg(linear) a0(0) a1(1) m(0)

//MEN//
use HANDLS_Allostaticload_dietfcfinal_PARAMED,clear
keep if sex==1
save HANDLS_Allostaticload_dietfcfinal_PARAMEDMEN, replace
```

```
paramed CRPW4, avar(FCOSTEXP) mvar(DASHDIETEXP) cvars(Agew1_C Agew3_C sex race pir edubr2 edubr3 edubr9 employed1 employed9 wrattbr2 wrattbr3 wrattbr4 smoke1 smoke9 currdrugs1 currdrugs9 bmi_C SRHbr2 SRHbr3 kcalw1w3mean_C energystoresw1w3mean_C invmillssem)    yreg(linear) mreg(linear) a0(0) a1(1) m(0)


//WOMEN//
use HANDLS_Allostaticload_dietfcfinal_PARAMED,clear
keep if sex==0
save HANDLS_Allostaticload_dietfcfinal_PARAMEDWOMEN, replace

paramed CRPW4, avar(FCOSTEXP) mvar(DASHDIETEXP) cvars(Agew1_C Agew3_C sex race pir edubr2 edubr3 edubr9 employed1 employed9 wrattbr2 wrattbr3 wrattbr4 smoke1 smoke9 currdrugs1 currdrugs9 bmi_C SRHbr2 SRHbr3 kcalw1w3mean_C energystoresw1w3mean_C invmillssem)    yreg(linear) mreg(linear) a0(0) a1(1) m(0)

//WHITES//
use HANDLS_Allostaticload_dietfcfinal_PARAMED,clear
keep if race==0
save HANDLS_Allostaticload_dietfcfinal_PARAMEDWHITES, replace

paramed CRPW4, avar(FCOSTEXP) mvar(DASHDIETEXP) cvars(Agew1_C Agew3_C sex race pir edubr2 edubr3 edubr9 employed1 employed9 wrattbr2 wrattbr3 wrattbr4 smoke1 smoke9 currdrugs1 currdrugs9 bmi_C SRHbr2 SRHbr3 kcalw1w3mean_C energystoresw1w3mean_C invmillssem)    yreg(linear) mreg(linear) a0(0) a1(1) m(0)


//African-Americans//
use HANDLS_Allostaticload_dietfcfinal_PARAMED,clear
keep if race==1
save HANDLS_Allostaticload_dietfcfinal_PARAMEDAA, replace

paramed CRPW4, avar(FCOSTEXP) mvar(DASHDIETEXP) cvars(Agew1_C Agew3_C sex race pir edubr2 edubr3 edubr9 employed1 employed9 wrattbr2 wrattbr3 wrattbr4 smoke1 smoke9 currdrugs1 currdrugs9 bmi_C SRHbr2 SRHbr3 kcalw1w3mean_C energystoresw1w3mean_C invmillssem)    yreg(linear) mreg(linear) a0(0) a1(1) m(0)

//BELOW POVERTY//

use HANDLS_Allostaticload_dietfcfinal_PARAMED,clear
keep if pir==0
save HANDLS_Allostaticload_dietfcfinal_PARAMEDBP, replace

paramed CRPW4, avar(FCOSTEXP) mvar(DASHDIETEXP) cvars(Agew1_C Agew3_C sex race pir edubr2 edubr3 edubr9 employed1 employed9 wrattbr2 wrattbr3 wrattbr4 smoke1 smoke9 currdrugs1 currdrugs9 bmi_C SRHbr2 SRHbr3 kcalw1w3mean_C energystoresw1w3mean_C invmillssem)    yreg(linear) mreg(linear) a0(0) a1(1) m(0)
```

//ABOVE POVERTY//

use HANDLS_Allostaticload_dietfcfinal_PARAMED,clear
keep if pir==1
save HANDLS_Allostaticload_dietfcfinal_PARAMEDAP, replace

paramed CRPW4, avar(FCOSTEXP) mvar(DASHDIETEXP) cvars(Agew1_C Agew3_C sex race pir edubr2 edubr3 edubr9 employed1 employed9 wrattbr2 wrattbr3 wrattbr4 smoke1 smoke9 currdrugs1 currdrugs9 bmi_C SRHbr2 SRHbr3 kcalw1w3mean_C energystoresw1w3mean_C invmillssem) yreg(linear) mreg(linear) a0(0) a1(1) m(0)

save, replace

//STANDARDIZED CHOLESTEROL, WAVE 4//
cd "G:\16GBBACKUPUSB\BACKUP_USB_SEPTEMBER2014\May Baydoun_folder\HANDLS_paper30_foodcost_MetSlong\DATA"

use HANDLS_Allostaticload_dietfcfinal_PARAMED, clear

capture drop CHOLW4
gen CHOLW4=zcholw4

save, replace

//ALL SAMPLE//

use HANDLS_Allostaticload_dietfcfinal_PARAMED, clear

paramed CHOLW4, avar(FCOSTEXP) mvar(DASHDIETEXP) cvars(Agew1_C Agew3_C sex race pir edubr2 edubr3 edubr9 employed1 employed9 wrattbr2 wrattbr3 wrattbr4 smoke1 smoke9 currdrugs1 currdrugs9 bmi_C SRHbr2 SRHbr3 kcalw1w3mean_C energystoresw1w3mean_C invmillssem) yreg(linear) mreg(linear) a0(0) a1(1) m(0)

//MEN//
use HANDLS_Allostaticload_dietfcfinal_PARAMED,clear
keep if sex==1
save HANDLS_Allostaticload_dietfcfinal_PARAMEDMEN, replace

paramed CHOLW4, avar(FCOSTEXP) mvar(DASHDIETEXP) cvars(Agew1_C Agew3_C sex race pir edubr2 edubr3 edubr9 employed1 employed9 wrattbr2 wrattbr3 wrattbr4 smoke1 smoke9 currdrugs1 currdrugs9 bmi_C SRHbr2 SRHbr3 kcalw1w3mean_C energystoresw1w3mean_C invmillssem) yreg(linear) mreg(linear) a0(0) a1(1) m(0)

```
//WOMEN//
use HANDLS_Allostaticload_dietfcfinal_PARAMED,clear
keep if sex==0
save HANDLS_Allostaticload_dietfcfinal_PARAMEDWOMEN, replace

paramed CHOLW4, avar(FCOSTEXP) mvar(DASHDIETEXP) cvars(Agew1_C Agew3_C sex race pir edubr2 edubr3 edubr9 employed1 employed9 wrattbr2 wrattbr3 wrattbr4 smoke1 smoke9 currdrugs1 currdrugs9 bmi_C SRHbr2 SRHbr3 kcalw1w3mean_C energystoresw1w3mean_C invmillssem)    yreg(linear) mreg(linear) a0(0) a1(1) m(0)

//WHITES//
use HANDLS_Allostaticload_dietfcfinal_PARAMED,clear
keep if race==0
save HANDLS_Allostaticload_dietfcfinal_PARAMEDWHITES, replace

paramed CHOLW4, avar(FCOSTEXP) mvar(DASHDIETEXP) cvars(Agew1_C Agew3_C sex race pir edubr2 edubr3 edubr9 employed1 employed9 wrattbr2 wrattbr3 wrattbr4 smoke1 smoke9 currdrugs1 currdrugs9 bmi_C SRHbr2 SRHbr3 kcalw1w3mean_C energystoresw1w3mean_C invmillssem)    yreg(linear) mreg(linear) a0(0) a1(1) m(0)


//African-Americans//
use HANDLS_Allostaticload_dietfcfinal_PARAMED,clear
keep if race==1
save HANDLS_Allostaticload_dietfcfinal_PARAMEDAA, replace

paramed CHOLW4, avar(FCOSTEXP) mvar(DASHDIETEXP) cvars(Agew1_C Agew3_C sex race pir edubr2 edubr3 edubr9 employed1 employed9 wrattbr2 wrattbr3 wrattbr4 smoke1 smoke9 currdrugs1 currdrugs9 bmi_C SRHbr2 SRHbr3 kcalw1w3mean_C energystoresw1w3mean_C invmillssem)    yreg(linear) mreg(linear) a0(0) a1(1) m(0)

//BELOW POVERTY//

use HANDLS_Allostaticload_dietfcfinal_PARAMED,clear
keep if pir==0
save HANDLS_Allostaticload_dietfcfinal_PARAMEDBP, replace

paramed CHOLW4, avar(FCOSTEXP) mvar(DASHDIETEXP) cvars(Agew1_C Agew3_C sex race pir edubr2 edubr3 edubr9 employed1 employed9 wrattbr2 wrattbr3 wrattbr4 smoke1 smoke9 currdrugs1 currdrugs9 bmi_C SRHbr2 SRHbr3 kcalw1w3mean_C energystoresw1w3mean_C invmillssem)    yreg(linear) mreg(linear) a0(0) a1(1) m(0)

//ABOVE POVERTY//

use HANDLS_Allostaticload_dietfcfinal_PARAMED,clear
keep if pir==1
save HANDLS_Allostaticload_dietfcfinal_PARAMEDAP, replace
```

```
paramed CHOLW4, avar(FCOSTEXP) mvar(DASHDIETEXP) cvars(Agew1_C Agew3_C sex race pir edubr2 edubr3 edubr9 employed1 employed9 wrattbr2 wrattbr3 wrattbr4 smoke1 smoke9 currdrugs1 currdrugs9 bmi_C SRHbr2 SRHbr3 kcalw1w3mean_C energystoresw1w3mean_C invmillssem)    yreg(linear) mreg(linear) a0(0) a1(1) m(0)

save, replace



/////////////////////////////////////////////////////////////////////////STANDARDIZED HDL-C, WAVE 4////////////////////////////////////////
cd "G:\16GBBACKUPUSB\BACKUP_USB_SEPTEMBER2014\May Baydoun_folder\HANDLS_paper30_foodcost_MetSlong\DATA"

use HANDLS_Allostaticload_dietfcfinal_PARAMED, clear


capture drop HDLW4
gen HDLW4=zhdlw4

save, replace

//ALL SAMPLE//

use HANDLS_Allostaticload_dietfcfinal_PARAMED, clear



paramed HDLW4, avar(FCOSTEXP) mvar(DASHDIETEXP) cvars(Agew1_C Agew3_C sex race pir edubr2 edubr3 edubr9 employed1 employed9 wrattbr2 wrattbr3 wrattbr4 smoke1 smoke9 currdrugs1 currdrugs9 bmi_C SRHbr2 SRHbr3 kcalw1w3mean_C energystoresw1w3mean_C invmillssem)    yreg(linear) mreg(linear) a0(0) a1(1) m(0)

//MEN//
use HANDLS_Allostaticload_dietfcfinal_PARAMED,clear
keep if sex==1
save HANDLS_Allostaticload_dietfcfinal_PARAMEDMEN, replace

paramed HDLW4, avar(FCOSTEXP) mvar(DASHDIETEXP) cvars(Agew1_C Agew3_C sex race pir edubr2 edubr3 edubr9 employed1 employed9 wrattbr2 wrattbr3 wrattbr4 smoke1 smoke9 currdrugs1 currdrugs9 bmi_C SRHbr2 SRHbr3 kcalw1w3mean_C energystoresw1w3mean_C invmillssem)    yreg(linear) mreg(linear) a0(0) a1(1) m(0)


//WOMEN//
use HANDLS_Allostaticload_dietfcfinal_PARAMED,clear
keep if sex==0
```

```
save HANDLS_Allostaticload_dietfcfinal_PARAMEDWOMEN, replace

paramed HDLW4, avar(FCOSTEXP) mvar(DASHDIETEXP) cvars(Agew1_C Agew3_C sex race pir edubr2 edubr3 edubr9 employed1 employed9 wrattbr2 wrattbr3 wrattbr4 smoke1 smoke9 currdrugs1 currdrugs9 bmi_C SRHbr2 SRHbr3 kcalw1w3mean_C energystoresw1w3mean_C invmillssem)    yreg(linear) mreg(linear) a0(0) a1(1) m(0)

//WHITES//
use HANDLS_Allostaticload_dietfcfinal_PARAMED,clear
keep if race==0
save HANDLS_Allostaticload_dietfcfinal_PARAMEDWHITES, replace

paramed HDLW4, avar(FCOSTEXP) mvar(DASHDIETEXP) cvars(Agew1_C Agew3_C sex race pir edubr2 edubr3 edubr9 employed1 employed9 wrattbr2 wrattbr3 wrattbr4 smoke1 smoke9 currdrugs1 currdrugs9 bmi_C SRHbr2 SRHbr3 kcalw1w3mean_C energystoresw1w3mean_C invmillssem)    yreg(linear) mreg(linear) a0(0) a1(1) m(0)


//African-Americans//
use HANDLS_Allostaticload_dietfcfinal_PARAMED,clear
keep if race==1
save HANDLS_Allostaticload_dietfcfinal_PARAMEDAA, replace

paramed HDLW4, avar(FCOSTEXP) mvar(DASHDIETEXP) cvars(Agew1_C Agew3_C sex race pir edubr2 edubr3 edubr9 employed1 employed9 wrattbr2 wrattbr3 wrattbr4 smoke1 smoke9 currdrugs1 currdrugs9 bmi_C SRHbr2 SRHbr3 kcalw1w3mean_C energystoresw1w3mean_C invmillssem)    yreg(linear) mreg(linear) a0(0) a1(1) m(0)

//BELOW POVERTY//

use HANDLS_Allostaticload_dietfcfinal_PARAMED,clear
keep if pir==0
save HANDLS_Allostaticload_dietfcfinal_PARAMEDBP, replace

paramed HDLW4, avar(FCOSTEXP) mvar(DASHDIETEXP) cvars(Agew1_C Agew3_C sex race pir edubr2 edubr3 edubr9 employed1 employed9 wrattbr2 wrattbr3 wrattbr4 smoke1 smoke9 currdrugs1 currdrugs9 bmi_C SRHbr2 SRHbr3 kcalw1w3mean_C energystoresw1w3mean_C invmillssem)    yreg(linear) mreg(linear) a0(0) a1(1) m(0)

//ABOVE POVERTY//

use HANDLS_Allostaticload_dietfcfinal_PARAMED,clear
keep if pir==1
save HANDLS_Allostaticload_dietfcfinal_PARAMEDAP, replace

paramed HDLW4, avar(FCOSTEXP) mvar(DASHDIETEXP) cvars(Agew1_C Agew3_C sex race pir edubr2 edubr3 edubr9 employed1 employed9 wrattbr2 wrattbr3 wrattbr4 smoke1 smoke9 currdrugs1 currdrugs9 bmi_C SRHbr2 SRHbr3 kcalw1w3mean_C energystoresw1w3mean_C invmillssem)    yreg(linear) mreg(linear) a0(0) a1(1) m(0)
```

save, replace

//STANDARDIZED HGBA1C, WAVE 4//////////////////////////////////////

cd "G:\16GBBACKUPUSB\BACKUP_USB_SEPTEMBER2014\May Baydoun_folder\HANDLS_paper30_foodcost_MetSlong\DATA"

use HANDLS_Allostaticload_dietfcfinal_PARAMED, clear

capture drop HGBA1C
gen HGBA1C=zhgba1cw4

save, replace

//ALL SAMPLE//

use HANDLS_Allostaticload_dietfcfinal_PARAMED, clear

paramed HGBA1C, avar(FCOSTEXP) mvar(DASHDIETEXP) cvars(Agew1_C Agew3_C sex race pir edubr2 edubr3 edubr9 employed1 employed9 wrattbr2 wrattbr3 wrattbr4 smoke1 smoke9 currdrugs1 currdrugs9 bmi_C SRHbr2 SRHbr3 kcalw1w3mean_C energystoresw1w3mean_C invmillssem) yreg(linear) mreg(linear) a0(0) a1(1) m(0)

//MEN//
use HANDLS_Allostaticload_dietfcfinal_PARAMED,clear
keep if sex==1
save HANDLS_Allostaticload_dietfcfinal_PARAMEDMEN, replace

paramed HGBA1C, avar(FCOSTEXP) mvar(DASHDIETEXP) cvars(Agew1_C Agew3_C sex race pir edubr2 edubr3 edubr9 employed1 employed9 wrattbr2 wrattbr3 wrattbr4 smoke1 smoke9 currdrugs1 currdrugs9 bmi_C SRHbr2 SRHbr3 kcalw1w3mean_C energystoresw1w3mean_C invmillssem) yreg(linear) mreg(linear) a0(0) a1(1) m(0)

//WOMEN//
use HANDLS_Allostaticload_dietfcfinal_PARAMED,clear
keep if sex==0
save HANDLS_Allostaticload_dietfcfinal_PARAMEDWOMEN, replace

```
paramed HGBA1C, avar(FCOSTEXP) mvar(DASHDIETEXP) cvars(Agew1_C Agew3_C sex race pir edubr2 edubr3 edubr9 employed1 employed9 wrattbr2 wrattbr3 wrattbr4 smoke1 smoke9 currdrugs1 currdrugs9 bmi_C SRHbr2 SRHbr3 kcalw1w3mean_C energystoresw1w3mean_C invmillssem)    yreg(linear) mreg(linear) a0(0) a1(1) m(0)

//WHITES//
use HANDLS_Allostaticload_dietfcfinal_PARAMED,clear
keep if race==0
save HANDLS_Allostaticload_dietfcfinal_PARAMEDWHITES, replace

paramed HGBA1C, avar(FCOSTEXP) mvar(DASHDIETEXP) cvars(Agew1_C Agew3_C sex race pir edubr2 edubr3 edubr9 employed1 employed9 wrattbr2 wrattbr3 wrattbr4 smoke1 smoke9 currdrugs1 currdrugs9 bmi_C SRHbr2 SRHbr3 kcalw1w3mean_C energystoresw1w3mean_C invmillssem)    yreg(linear) mreg(linear) a0(0) a1(1) m(0)



//African-Americans//
use HANDLS_Allostaticload_dietfcfinal_PARAMED,clear
keep if race==1
save HANDLS_Allostaticload_dietfcfinal_PARAMEDAA, replace

paramed HGBA1C, avar(FCOSTEXP) mvar(DASHDIETEXP) cvars(Agew1_C Agew3_C sex race pir edubr2 edubr3 edubr9 employed1 employed9 wrattbr2 wrattbr3 wrattbr4 smoke1 smoke9 currdrugs1 currdrugs9 bmi_C SRHbr2 SRHbr3 kcalw1w3mean_C energystoresw1w3mean_C invmillssem)    yreg(linear) mreg(linear) a0(0) a1(1) m(0)

//BELOW POVERTY//

use HANDLS_Allostaticload_dietfcfinal_PARAMED,clear
keep if pir==0
save HANDLS_Allostaticload_dietfcfinal_PARAMEDBP, replace

paramed HGBA1C, avar(FCOSTEXP) mvar(DASHDIETEXP) cvars(Agew1_C Agew3_C sex race pir edubr2 edubr3 edubr9 employed1 employed9 wrattbr2 wrattbr3 wrattbr4 smoke1 smoke9 currdrugs1 currdrugs9 bmi_C SRHbr2 SRHbr3 kcalw1w3mean_C energystoresw1w3mean_C invmillssem)    yreg(linear) mreg(linear) a0(0) a1(1) m(0)

//ABOVE POVERTY//

use HANDLS_Allostaticload_dietfcfinal_PARAMED,clear
keep if pir==1
save HANDLS_Allostaticload_dietfcfinal_PARAMEDAP, replace

paramed HGBA1C, avar(FCOSTEXP) mvar(DASHDIETEXP) cvars(Agew1_C Agew3_C sex race pir edubr2 edubr3 edubr9 employed1 employed9 wrattbr2 wrattbr3 wrattbr4 smoke1 smoke9 currdrugs1 currdrugs9 bmi_C SRHbr2 SRHbr3 kcalw1w3mean_C energystoresw1w3mean_C invmillssem)    yreg(linear) mreg(linear) a0(0) a1(1) m(0)

save, replace
```

```
//////////////////////////////////////////////////////////////////////STANDARDIZED WAIST-TO-HIP RATIO WAVE 4/////////////////////////////////////
cd "G:\16GBBACKUPUSB\BACKUP_USB_SEPTEMBER2014\May Baydoun_folder\HANDLS_paper30_foodcost_MetSlong\DATA"

use HANDLS_Allostaticload_dietfcfinal_PARAMED, clear



capture drop WHRW4
gen WHRW4=zwhrw4

save, replace

//ALL SAMPLE//

use HANDLS_Allostaticload_dietfcfinal_PARAMED, clear




paramed WHRW4, avar(FCOSTEXP) mvar(DASHDIETEXP) cvars(Agew1_C Agew3_C sex race pir edubr2 edubr3 edubr9 employed1 employed9 wrattbr2 wrattbr3 wrattbr4 smoke1 smoke9 currdrugs1 currdrugs9 bmi_C SRHbr2 SRHbr3 kcalw1w3mean_C energystoresw1w3mean_C invmillssem)    yreg(linear) mreg(linear) a0(0) a1(1) m(0)

//MEN//
use HANDLS_Allostaticload_dietfcfinal_PARAMED,clear
keep if sex==1
save HANDLS_Allostaticload_dietfcfinal_PARAMEDMEN, replace

paramed WHRW4, avar(FCOSTEXP) mvar(DASHDIETEXP) cvars(Agew1_C Agew3_C sex race pir edubr2 edubr3 edubr9 employed1 employed9 wrattbr2 wrattbr3 wrattbr4 smoke1 smoke9 currdrugs1 currdrugs9 bmi_C SRHbr2 SRHbr3 kcalw1w3mean_C energystoresw1w3mean_C invmillssem)    yreg(linear) mreg(linear) a0(0) a1(1) m(0)



//WOMEN//
use HANDLS_Allostaticload_dietfcfinal_PARAMED,clear
keep if sex==0
save HANDLS_Allostaticload_dietfcfinal_PARAMEDWOMEN, replace
```

```
paramed WHRW4, avar(FCOSTEXP) mvar(DASHDIETEXP) cvars(Agew1_C Agew3_C sex race pir edubr2 edubr3 edubr9 employed1 employed9 wrattbr2 wrattbr3 wrattbr4 smoke1 smoke9 currdrugs1 currdrugs9 bmi_C SRHbr2 SRHbr3 kcalw1w3mean_C energystoresw1w3mean_C invmillssem)    yreg(linear) mreg(linear) a0(0) a1(1) m(0)

//WHITES//
use HANDLS_Allostaticload_dietfcfinal_PARAMED,clear
keep if race==0
save HANDLS_Allostaticload_dietfcfinal_PARAMEDWHITES, replace

paramed WHRW4, avar(FCOSTEXP) mvar(DASHDIETEXP) cvars(Agew1_C Agew3_C sex race pir edubr2 edubr3 edubr9 employed1 employed9 wrattbr2 wrattbr3 wrattbr4 smoke1 smoke9 currdrugs1 currdrugs9 bmi_C SRHbr2 SRHbr3 kcalw1w3mean_C energystoresw1w3mean_C invmillssem)    yreg(linear) mreg(linear) a0(0) a1(1) m(0)

//African-Americans//
use HANDLS_Allostaticload_dietfcfinal_PARAMED,clear
keep if race==1
save HANDLS_Allostaticload_dietfcfinal_PARAMEDAA, replace

paramed WHRW4, avar(FCOSTEXP) mvar(DASHDIETEXP) cvars(Agew1_C Agew3_C sex race pir edubr2 edubr3 edubr9 employed1 employed9 wrattbr2 wrattbr3 wrattbr4 smoke1 smoke9 currdrugs1 currdrugs9 bmi_C SRHbr2 SRHbr3 kcalw1w3mean_C energystoresw1w3mean_C invmillssem)    yreg(linear) mreg(linear) a0(0) a1(1) m(0)

//BELOW POVERTY//

use HANDLS_Allostaticload_dietfcfinal_PARAMED,clear
keep if pir==0
save HANDLS_Allostaticload_dietfcfinal_PARAMEDBP, replace

paramed WHRW4, avar(FCOSTEXP) mvar(DASHDIETEXP) cvars(Agew1_C Agew3_C sex race pir edubr2 edubr3 edubr9 employed1 employed9 wrattbr2 wrattbr3 wrattbr4 smoke1 smoke9 currdrugs1 currdrugs9 bmi_C SRHbr2 SRHbr3 kcalw1w3mean_C energystoresw1w3mean_C invmillssem)    yreg(linear) mreg(linear) a0(0) a1(1) m(0)

//ABOVE POVERTY//

use HANDLS_Allostaticload_dietfcfinal_PARAMED,clear
keep if pir==1
save HANDLS_Allostaticload_dietfcfinal_PARAMEDAP, replace

paramed WHRW4, avar(FCOSTEXP) mvar(DASHDIETEXP) cvars(Agew1_C Agew3_C sex race pir edubr2 edubr3 edubr9 employed1 employed9 wrattbr2 wrattbr3 wrattbr4 smoke1 smoke9 currdrugs1 currdrugs9 bmi_C SRHbr2 SRHbr3 kcalw1w3mean_C energystoresw1w3mean_C invmillssem)    yreg(linear) mreg(linear) a0(0) a1(1) m(0)

save, replace
```

//STANDARDIZED SYSTOLIC BLOOD PRESSURE WAVE 4: bpsysw4 //////////////////////////////////////

cd "G:\16GBBACKUPUSB\BACKUP_USB_SEPTEMBER2014\May Baydoun_folder\HANDLS_paper30_foodcost_MetSlong\DATA"

use HANDLS_Allostaticload_dietfcfinal_PARAMED, clear

capture drop BPSYSW4
gen BPSYSW4=zbpsysw4

save, replace

//ALL SAMPLE//

use HANDLS_Allostaticload_dietfcfinal_PARAMED, clear

paramed BPSYSW4, avar(FCOSTEXP) mvar(DASHDIETEXP) cvars(Agew1_C Agew3_C sex race pir edubr2 edubr3 edubr9 employed1 employed9 wrattbr2 wrattbr3 wrattbr4 smoke1 smoke9 currdrugs1 currdrugs9 bmi_C SRHbr2 SRHbr3 kcalw1w3mean_C energystoresw1w3mean_C invmillssem) yreg(linear) mreg(linear) a0(0) a1(1) m(0)

//MEN//
use HANDLS_Allostaticload_dietfcfinal_PARAMED,clear
keep if sex==1
save HANDLS_Allostaticload_dietfcfinal_PARAMEDMEN, replace

paramed BPSYSW4, avar(FCOSTEXP) mvar(DASHDIETEXP) cvars(Agew1_C Agew3_C sex race pir edubr2 edubr3 edubr9 employed1 employed9 wrattbr2 wrattbr3 wrattbr4 smoke1 smoke9 currdrugs1 currdrugs9 bmi_C SRHbr2 SRHbr3 kcalw1w3mean_C energystoresw1w3mean_C invmillssem) yreg(linear) mreg(linear) a0(0) a1(1) m(0)

//WOMEN//
use HANDLS_Allostaticload_dietfcfinal_PARAMED,clear
keep if sex==0
save HANDLS_Allostaticload_dietfcfinal_PARAMEDWOMEN, replace

```
paramed BPSYSW4, avar(FCOSTEXP) mvar(DASHDIETEXP) cvars(Agew1_C Agew3_C sex race pir edubr2 edubr3 edubr9 employed1 employed9 wrattbr2 wrattbr3 wrattbr4 smoke1 smoke9 currdrugs1 currdrugs9 bmi_C SRHbr2 SRHbr3 kcalw1w3mean_C energystoresw1w3mean_C invmillssem)    yreg(linear) mreg(linear) a0(0) a1(1) m(0)

//WHITES//
use HANDLS_Allostaticload_dietfcfinal_PARAMED,clear
keep if race==0
save HANDLS_Allostaticload_dietfcfinal_PARAMEDWHITES, replace

paramed BPSYSW4, avar(FCOSTEXP) mvar(DASHDIETEXP) cvars(Agew1_C Agew3_C sex race pir edubr2 edubr3 edubr9 employed1 employed9 wrattbr2 wrattbr3 wrattbr4 smoke1 smoke9 currdrugs1 currdrugs9 bmi_C SRHbr2 SRHbr3 kcalw1w3mean_C energystoresw1w3mean_C invmillssem)    yreg(linear) mreg(linear) a0(0) a1(1) m(0)



//African-Americans//
use HANDLS_Allostaticload_dietfcfinal_PARAMED,clear
keep if race==1
save HANDLS_Allostaticload_dietfcfinal_PARAMEDAA, replace

paramed BPSYSW4, avar(FCOSTEXP) mvar(DASHDIETEXP) cvars(Agew1_C Agew3_C sex race pir edubr2 edubr3 edubr9 employed1 employed9 wrattbr2 wrattbr3 wrattbr4 smoke1 smoke9 currdrugs1 currdrugs9 bmi_C SRHbr2 SRHbr3 kcalw1w3mean_C energystoresw1w3mean_C invmillssem)    yreg(linear) mreg(linear) a0(0) a1(1) m(0)

//BELOW POVERTY//

use HANDLS_Allostaticload_dietfcfinal_PARAMED,clear
keep if pir==0
save HANDLS_Allostaticload_dietfcfinal_PARAMEDBP, replace

paramed BPSYSW4, avar(FCOSTEXP) mvar(DASHDIETEXP) cvars(Agew1_C Agew3_C sex race pir edubr2 edubr3 edubr9 employed1 employed9 wrattbr2 wrattbr3 wrattbr4 smoke1 smoke9 currdrugs1 currdrugs9 bmi_C SRHbr2 SRHbr3 kcalw1w3mean_C energystoresw1w3mean_C invmillssem)    yreg(linear) mreg(linear) a0(0) a1(1) m(0)

//ABOVE POVERTY//

use HANDLS_Allostaticload_dietfcfinal_PARAMED,clear
keep if pir==1
save HANDLS_Allostaticload_dietfcfinal_PARAMEDAP, replace

paramed BPSYSW4, avar(FCOSTEXP) mvar(DASHDIETEXP) cvars(Agew1_C Agew3_C sex race pir edubr2 edubr3 edubr9 employed1 employed9 wrattbr2 wrattbr3 wrattbr4 smoke1 smoke9 currdrugs1 currdrugs9 bmi_C SRHbr2 SRHbr3 kcalw1w3mean_C energystoresw1w3mean_C invmillssem)    yreg(linear) mreg(linear) a0(0) a1(1) m(0)

save, replace
```

```
//////////////////////////////////////////////////////////////////////DIASTOLIC BLOOD PRESSURE WAVE 4: BPDIAW4 //////////////////////////////////////
cd "G:\16GBBACKUPUSB\BACKUP_USB_SEPTEMBER2014\May Baydoun_folder\HANDLS_paper30_foodcost_MetSlong\DATA"

use HANDLS_Allostaticload_dietfcfinal_PARAMED, clear


capture drop BPDIAW4
gen BPDIAW4=zbpdiaw4

save, replace

//ALL SAMPLE//

use HANDLS_Allostaticload_dietfcfinal_PARAMED, clear


paramed BPDIAW4, avar(FCOSTEXP) mvar(DASHDIETEXP) cvars(Agew1_C Agew3_C sex race pir edubr2 edubr3 edubr9 employed1 employed9 wrattbr2 wrattbr3 wrattbr4 smoke1 smoke9 currdrugs1 currdrugs9 bmi_C SRHbr2 SRHbr3 kcalw1w3mean_C energystoresw1w3mean_C invmillssem)    yreg(linear) mreg(linear) a0(0) a1(1) m(0)

//MEN//
use HANDLS_Allostaticload_dietfcfinal_PARAMED,clear
keep if sex==1
save HANDLS_Allostaticload_dietfcfinal_PARAMEDMEN, replace

paramed BPDIAW4, avar(FCOSTEXP) mvar(DASHDIETEXP) cvars(Agew1_C Agew3_C sex race pir edubr2 edubr3 edubr9 employed1 employed9 wrattbr2 wrattbr3 wrattbr4 smoke1 smoke9 currdrugs1 currdrugs9 bmi_C SRHbr2 SRHbr3 kcalw1w3mean_C energystoresw1w3mean_C invmillssem)    yreg(linear) mreg(linear) a0(0) a1(1) m(0)


//WOMEN//
use HANDLS_Allostaticload_dietfcfinal_PARAMED,clear
keep if sex==0
save HANDLS_Allostaticload_dietfcfinal_PARAMEDWOMEN, replace

paramed BPDIAW4, avar(FCOSTEXP) mvar(DASHDIETEXP) cvars(Agew1_C Agew3_C sex race pir edubr2 edubr3 edubr9 employed1 employed9 wrattbr2 wrattbr3 wrattbr4 smoke1 smoke9 currdrugs1 currdrugs9 bmi_C SRHbr2 SRHbr3 kcalw1w3mean_C energystoresw1w3mean_C invmillssem)    yreg(linear) mreg(linear) a0(0) a1(1) m(0)
```

```
//WHITES//
use HANDLS_Allostaticload_dietfcfinal_PARAMED,clear
keep if race==0
save HANDLS_Allostaticload_dietfcfinal_PARAMEDWHITES, replace

paramed BPDIAW4, avar(FCOSTEXP) mvar(DASHDIETEXP) cvars(Agew1_C Agew3_C sex race pir edubr2 edubr3 edubr9 employed1 employed9 wrattbr2 wrattbr3 wrattbr4 smoke1 smoke9 currdrugs1 currdrugs9 bmi_C SRHbr2 SRHbr3 kcalw1w3mean_C energystoresw1w3mean_C invmillssem)    yreg(linear) mreg(linear) a0(0) a1(1) m(0)



//African-Americans//
use HANDLS_Allostaticload_dietfcfinal_PARAMED,clear
keep if race==1
save HANDLS_Allostaticload_dietfcfinal_PARAMEDAA, replace

paramed BPDIAW4, avar(FCOSTEXP) mvar(DASHDIETEXP) cvars(Agew1_C Agew3_C sex race pir edubr2 edubr3 edubr9 employed1 employed9 wrattbr2 wrattbr3 wrattbr4 smoke1 smoke9 currdrugs1 currdrugs9 bmi_C SRHbr2 SRHbr3 kcalw1w3mean_C energystoresw1w3mean_C invmillssem)    yreg(linear) mreg(linear) a0(0) a1(1) m(0)

//BELOW POVERTY//

use HANDLS_Allostaticload_dietfcfinal_PARAMED,clear
keep if pir==0
save HANDLS_Allostaticload_dietfcfinal_PARAMEDBP, replace

paramed BPDIAW4, avar(FCOSTEXP) mvar(DASHDIETEXP) cvars(Agew1_C Agew3_C sex race pir edubr2 edubr3 edubr9 employed1 employed9 wrattbr2 wrattbr3 wrattbr4 smoke1 smoke9 currdrugs1 currdrugs9 bmi_C SRHbr2 SRHbr3 kcalw1w3mean_C energystoresw1w3mean_C invmillssem)    yreg(linear) mreg(linear) a0(0) a1(1) m(0)

//ABOVE POVERTY//

use HANDLS_Allostaticload_dietfcfinal_PARAMED,clear
keep if pir==1
save HANDLS_Allostaticload_dietfcfinal_PARAMEDAP, replace

paramed BPDIAW4, avar(FCOSTEXP) mvar(DASHDIETEXP) cvars(Agew1_C Agew3_C sex race pir edubr2 edubr3 edubr9 employed1 employed9 wrattbr2 wrattbr3 wrattbr4 smoke1 smoke9 currdrugs1 currdrugs9 bmi_C SRHbr2 SRHbr3 kcalw1w3mean_C energystoresw1w3mean_C invmillssem)    yreg(linear) mreg(linear) a0(0) a1(1) m(0)

save, replace


///////////////////////////////////////////////////////////////////HEART RATE WAVE 4: //////////////////////////////////////
```

```
cd "G:\16GBBACKUPUSB\BACKUP_USB_SEPTEMBER2014\May Baydoun_folder\HANDLS_paper30_foodcost_MetSlong\DATA"

use HANDLS_Allostaticload_dietfcfinal_PARAMED, clear


capture drop HRW4
gen HRW4=zhrw4

save, replace

//ALL SAMPLE//

use HANDLS_Allostaticload_dietfcfinal_PARAMED, clear



paramed HRW4, avar(FCOSTEXP) mvar(DASHDIETEXP) cvars(Agew1_C Agew3_C sex race pir edubr2 edubr3 edubr9 employed1 employed9 wrattbr2 wrattbr3 wrattbr4 smoke1 smoke9 currdrugs1 currdrugs9 bmi_C SRHbr2 SRHbr3 kcalw1w3mean_C energystoresw1w3mean_C invmillssem)    yreg(linear) mreg(linear) a0(0) a1(1) m(0)

//MEN//
use HANDLS_Allostaticload_dietfcfinal_PARAMED,clear
keep if sex==1
save HANDLS_Allostaticload_dietfcfinal_PARAMEDMEN, replace

paramed HRW4, avar(FCOSTEXP) mvar(DASHDIETEXP) cvars(Agew1_C Agew3_C sex race pir edubr2 edubr3 edubr9 employed1 employed9 wrattbr2 wrattbr3 wrattbr4 smoke1 smoke9 currdrugs1 currdrugs9 bmi_C SRHbr2 SRHbr3 kcalw1w3mean_C energystoresw1w3mean_C invmillssem)    yreg(linear) mreg(linear) a0(0) a1(1) m(0)


//WOMEN//
use HANDLS_Allostaticload_dietfcfinal_PARAMED,clear
keep if sex==0
save HANDLS_Allostaticload_dietfcfinal_PARAMEDWOMEN, replace

paramed HRW4, avar(FCOSTEXP) mvar(DASHDIETEXP) cvars(Agew1_C Agew3_C sex race pir edubr2 edubr3 edubr9 employed1 employed9 wrattbr2 wrattbr3 wrattbr4 smoke1 smoke9 currdrugs1 currdrugs9 bmi_C SRHbr2 SRHbr3 kcalw1w3mean_C energystoresw1w3mean_C invmillssem)    yreg(linear) mreg(linear) a0(0) a1(1) m(0)

//WHITES//
use HANDLS_Allostaticload_dietfcfinal_PARAMED,clear
keep if race==0
save HANDLS_Allostaticload_dietfcfinal_PARAMEDWHITES, replace
```

```
paramed HRW4, avar(FCOSTEXP) mvar(DASHDIETEXP) cvars(Agew1_C Agew3_C sex race pir edubr2 edubr3 edubr9 employed1 employed9 wrattbr2 wrattbr3 wrattbr4 smoke1 smoke9 currdrugs1 currdrugs9 bmi_C SRHbr2 SRHbr3 kcalw1w3mean_C energystoresw1w3mean_C invmillssem)    yreg(linear) mreg(linear) a0(0) a1(1) m(0)


//African-Americans//
use HANDLS_Allostaticload_dietfcfinal_PARAMED,clear
keep if race==1
save HANDLS_Allostaticload_dietfcfinal_PARAMEDAA, replace

paramed HRW4, avar(FCOSTEXP) mvar(DASHDIETEXP) cvars(Agew1_C Agew3_C sex race pir edubr2 edubr3 edubr9 employed1 employed9 wrattbr2 wrattbr3 wrattbr4 smoke1 smoke9 currdrugs1 currdrugs9 bmi_C SRHbr2 SRHbr3 kcalw1w3mean_C energystoresw1w3mean_C invmillssem)    yreg(linear) mreg(linear) a0(0) a1(1) m(0)

//BELOW POVERTY//

use HANDLS_Allostaticload_dietfcfinal_PARAMED,clear
keep if pir==0
save HANDLS_Allostaticload_dietfcfinal_PARAMEDBP, replace

paramed HRW4, avar(FCOSTEXP) mvar(DASHDIETEXP) cvars(Agew1_C Agew3_C sex race pir edubr2 edubr3 edubr9 employed1 employed9 wrattbr2 wrattbr3 wrattbr4 smoke1 smoke9 currdrugs1 currdrugs9 bmi_C SRHbr2 SRHbr3 kcalw1w3mean_C energystoresw1w3mean_C invmillssem)    yreg(linear) mreg(linear) a0(0) a1(1) m(0)

//ABOVE POVERTY//

use HANDLS_Allostaticload_dietfcfinal_PARAMED,clear
keep if pir==1
save HANDLS_Allostaticload_dietfcfinal_PARAMEDAP, replace

paramed HRW4, avar(FCOSTEXP) mvar(DASHDIETEXP) cvars(Agew1_C Agew3_C sex race pir edubr2 edubr3 edubr9 employed1 employed9 wrattbr2 wrattbr3 wrattbr4 smoke1 smoke9 currdrugs1 currdrugs9 bmi_C SRHbr2 SRHbr3 kcalw1w3mean_C energystoresw1w3mean_C invmillssem)    yreg(linear) mreg(linear) a0(0) a1(1) m(0)

save, replace



////////////////////////////TABLE 5: STRUCTURAL EQUATIONS MODELS////////////////////////////


cd "G:\...\DATA"
```

```
use HANDLS_Allostaticload_dietfcfinal_PARAMED, clear

*****************************ALLOSTATIC LOAD WAVE 4********************************************
//MULTI-GROUP//

**All**
sem (FCOSTEXP -> ALW4, ) (DASHDIETEXP -> ALW4, ) ( Agew1_C-> ALW4, ) (Agew3_C -> ALW4, ) (sex -> ALW4, ) (race
-> ALW4, ) (pir -> ALW4, ) (edubr2 -> ALW4, ) (edubr3 -> ALW4, ) (employed1 -> ALW4, )    (wrattbr2 -> ALW4, ) (wrattbr3 ->
ALW4, ) (wrattbr4 -> ALW4, )(smoke1 -> ALW4, ) (smoke9 -> ALW4, )(currdrugs1 -> ALW4, ) (currdrugs9 -> ALW4, ) (bmi_C
-> ALW4, ) (SRHbr2 -> ALW4, ) (SRHbr3 -> ALW4, )(kcalw1w3mean_C -> ALW4, ) (energystoresw1w3mean_C -> ALW4, )
(invmillssem -> ALW4, ) ///
(FCOSTEXP -> DASHDIETEXP, )( Agew1_C-> DASHDIETEXP, ) (Agew3_C -> DASHDIETEXP, ) (sex -> DASHDIETEXP, )
(race -> DASHDIETEXP, ) (pir -> DASHDIETEXP, ) (edubr2 -> DASHDIETEXP, ) (edubr3 -> DASHDIETEXP, ) (employed1 ->
DASHDIETEXP, )    (wrattbr2 -> DASHDIETEXP, ) (wrattbr3 -> DASHDIETEXP, ) (wrattbr4 -> DASHDIETEXP, )(smoke1 ->
DASHDIETEXP, ) (smoke9 -> DASHDIETEXP, )(currdrugs1 -> DASHDIETEXP, ) (currdrugs9 -> DASHDIETEXP, ) (bmi_C ->
DASHDIETEXP, ) (SRHbr2 -> DASHDIETEXP, ) (SRHbr3 -> DASHDIETEXP, )(kcalw1w3mean_C -> DASHDIETEXP, )
(energystoresw1w3mean_C -> DASHDIETEXP, ) (invmillssem -> DASHDIETEXP, ) , nocapslatent method(ml)
group(finalsample)

estat ginvariant

estat gof, stats(all)
estat teffects

**By sex**

sem (FCOSTEXP -> ALW4, ) (DASHDIETEXP -> ALW4, ) ( Agew1_C-> ALW4, ) (Agew3_C -> ALW4, ) (race -> ALW4, ) (pir ->
ALW4, ) (edubr2 -> ALW4, ) (edubr3 -> ALW4, ) (employed1 -> ALW4, )    (wrattbr2 -> ALW4, ) (wrattbr3 -> ALW4, ) (wrattbr4
-> ALW4, )(smoke1 -> ALW4, ) (smoke9 -> ALW4, )(currdrugs1 -> ALW4, ) (currdrugs9 -> ALW4, ) (bmi_C -> ALW4, ) (SRHbr2
-> ALW4, ) (SRHbr3 -> ALW4, )(kcalw1w3mean_C -> ALW4, ) (energystoresw1w3mean_C -> ALW4, ) (invmillssem -> ALW4, )
///
(FCOSTEXP -> DASHDIETEXP, )( Agew1_C-> DASHDIETEXP, ) (Agew3_C -> DASHDIETEXP, ) (race -> DASHDIETEXP, )
(pir -> DASHDIETEXP, ) (edubr2 -> DASHDIETEXP, ) (edubr3 -> DASHDIETEXP, ) (employed1 -> DASHDIETEXP, )
(wrattbr2 -> DASHDIETEXP, ) (wrattbr3 -> DASHDIETEXP, )(wrattbr4 -> DASHDIETEXP, ) (smoke1 -> DASHDIETEXP, )
(smoke9 -> DASHDIETEXP, )(currdrugs1 -> DASHDIETEXP, ) (currdrugs9 -> DASHDIETEXP, ) (bmi_C -> DASHDIETEXP, )
(SRHbr2 -> DASHDIETEXP, ) (SRHbr3 -> DASHDIETEXP, )(kcalw1w3mean_C -> DASHDIETEXP, )
(energystoresw1w3mean_C -> DASHDIETEXP, ) (invmillssem -> DASHDIETEXP, ) if finalsample==1 , nocapslatent
method(ml) group(sex)

estat ginvariant



**By race**
```

sem (FCOSTEXP -> ALW4,) (DASHDIETEXP -> ALW4,) (Agew1_C-> ALW4,) (Agew3_C -> ALW4,) (sex -> ALW4,) (pir -> ALW4,) (edubr2 -> ALW4,) (edubr3 -> ALW4,) (employed1 -> ALW4,) (wrattbr2 -> ALW4,) (wrattbr3 -> ALW4,) (wrattbr4 -> ALW4,)(smoke1 -> ALW4,) (smoke9 -> ALW4,)(currdrugs1 -> ALW4,) (currdrugs9 -> ALW4,) (bmi_C -> ALW4,) (SRHbr2 -> ALW4,) (SRHbr3 -> ALW4,)(kcalw1w3mean_C -> ALW4,) (energystoresw1w3mean_C -> ALW4,) (invmillssem -> ALW4,) ///
(FCOSTEXP -> DASHDIETEXP,)(Agew1_C-> DASHDIETEXP,) (Agew3_C -> DASHDIETEXP,) (sex -> DASHDIETEXP,) (pir -> DASHDIETEXP,) (edubr2 -> DASHDIETEXP,) (edubr3 -> DASHDIETEXP,) (employed1 -> DASHDIETEXP,) (wrattbr2 -> DASHDIETEXP,) (wrattbr3 -> DASHDIETEXP,) (wrattbr4 -> DASHDIETEXP,)(smoke1 -> DASHDIETEXP,) (smoke9 -> DASHDIETEXP,)(currdrugs1 -> DASHDIETEXP,) (currdrugs9 -> DASHDIETEXP,) (bmi_C -> DASHDIETEXP,) (SRHbr2 -> DASHDIETEXP,) (SRHbr3 -> DASHDIETEXP,)(kcalw1w3mean_C -> DASHDIETEXP,) (energystoresw1w3mean_C -> DASHDIETEXP,) (invmillssem -> DASHDIETEXP,) if finalsample==1 , nocapslatent method(ml) group(race)

estat ginvariant

By pir

sem (FCOSTEXP -> ALW4,) (DASHDIETEXP -> ALW4,) (Agew1_C-> ALW4,) (Agew3_C -> ALW4,) (sex -> ALW4,) (race -> ALW4,) (edubr2 -> ALW4,) (edubr3 -> ALW4,) (employed1 -> ALW4,) (wrattbr2 -> ALW4,) (wrattbr3 -> ALW4,) (wrattbr4 -> ALW4,)(smoke1 -> ALW4,) (smoke9 -> ALW4,)(currdrugs1 -> ALW4,) (currdrugs9 -> ALW4,) (bmi_C -> ALW4,) (SRHbr2 -> ALW4,) (SRHbr3 -> ALW4,)(kcalw1w3mean_C -> ALW4,) (energystoresw1w3mean_C -> ALW4,) (invmillssem -> ALW4,) ///
(FCOSTEXP -> DASHDIETEXP,)(Agew1_C-> DASHDIETEXP,) (Agew3_C -> DASHDIETEXP,) (sex -> DASHDIETEXP,) (race -> DASHDIETEXP,) (edubr2 -> DASHDIETEXP,) (edubr3 -> DASHDIETEXP,) (employed1 -> DASHDIETEXP,) (wrattbr2 -> DASHDIETEXP,) (wrattbr3 -> DASHDIETEXP,) (wrattbr4 -> DASHDIETEXP,)(smoke1 -> DASHDIETEXP,) (smoke9 -> DASHDIETEXP,)(currdrugs1 -> DASHDIETEXP,) (currdrugs9 -> DASHDIETEXP,) (bmi_C -> DASHDIETEXP,) (SRHbr2 -> DASHDIETEXP,) (SRHbr3 -> DASHDIETEXP,)(kcalw1w3mean_C -> DASHDIETEXP,) (energystoresw1w3mean_C -> DASHDIETEXP,) (invmillssem -> DASHDIETEXP,) if finalsample==1 , nocapslatent method(ml) group(pir)

estat ginvariant

********************STRATIFIED ANALYSIS********************************

//MEN//
sem (FCOSTEXP -> ALW4,) (DASHDIETEXP -> ALW4,) (Agew1_C-> ALW4,) (Agew3_C -> ALW4,) (race -> ALW4,) (pir -> ALW4,) (edubr2 -> ALW4,) (edubr3 -> ALW4,) (employed1 -> ALW4,) (wrattbr2 -> ALW4,) (wrattbr3 -> ALW4,) (wrattbr4 -> ALW4,)(smoke1 -> ALW4,) (smoke9 -> ALW4,)(currdrugs1 -> ALW4,) (currdrugs9 -> ALW4,) (bmi_C -> ALW4,) (SRHbr2 -> ALW4,) (SRHbr3 -> ALW4,)(kcalw1w3mean_C -> ALW4,) (energystoresw1w3mean_C -> ALW4,) (invmillssem -> ALW4,) ///
(FCOSTEXP -> DASHDIETEXP,)(Agew1_C-> DASHDIETEXP,) (Agew3_C -> DASHDIETEXP,) (race -> DASHDIETEXP,) (pir -> DASHDIETEXP,) (edubr2 -> DASHDIETEXP,) (edubr3 -> DASHDIETEXP,) (employed1 -> DASHDIETEXP,) (wrattbr2 -> DASHDIETEXP,) (wrattbr3 -> DASHDIETEXP,)(wrattbr4 -> DASHDIETEXP,) (smoke1 -> DASHDIETEXP,)

(smoke9 -> DASHDIETEXP,)(currdrugs1 -> DASHDIETEXP,) (currdrugs9 -> DASHDIETEXP,) (bmi_C -> DASHDIETEXP,) (SRHbr2 -> DASHDIETEXP,) (SRHbr3 -> DASHDIETEXP,)(kcalw1w3mean_C -> DASHDIETEXP,) (energystoresw1w3mean_C -> DASHDIETEXP,) (invmillssem -> DASHDIETEXP,) if finalsample==1 & sex==1 , nocapslatent method(ml)

estat gof, stats(all)
estat teffects

//WOMEN//
sem (FCOSTEXP -> ALW4,) (DASHDIETEXP -> ALW4,) (Agew1_C-> ALW4,) (Agew3_C -> ALW4,) (race -> ALW4,) (pir -> ALW4,) (edubr2 -> ALW4,) (edubr3 -> ALW4,) (employed1 -> ALW4,) (wrattbr2 -> ALW4,) (wrattbr3 -> ALW4,) (wrattbr4 -> ALW4,)(smoke1 -> ALW4,) (smoke9 -> ALW4,)(currdrugs1 -> ALW4,) (currdrugs9 -> ALW4,) (bmi_C -> ALW4,) (SRHbr2 -> ALW4,) (SRHbr3 -> ALW4,)(kcalw1w3mean_C -> ALW4,) (energystoresw1w3mean_C -> ALW4,) (invmillssem -> ALW4,) ///
(FCOSTEXP -> DASHDIETEXP,)(Agew1_C-> DASHDIETEXP,) (Agew3_C -> DASHDIETEXP,) (race -> DASHDIETEXP,) (pir -> DASHDIETEXP,) (edubr2 -> DASHDIETEXP,) (edubr3 -> DASHDIETEXP,) (employed1 -> DASHDIETEXP,) (wrattbr2 -> DASHDIETEXP,) (wrattbr3 -> DASHDIETEXP,) (wrattbr4 -> DASHDIETEXP,)(smoke1 -> DASHDIETEXP,) (smoke9 -> DASHDIETEXP,)(currdrugs1 -> DASHDIETEXP,) (currdrugs9 -> DASHDIETEXP,) (bmi_C -> DASHDIETEXP,) (SRHbr2 -> DASHDIETEXP,) (SRHbr3 -> DASHDIETEXP,)(kcalw1w3mean_C -> DASHDIETEXP,) (energystoresw1w3mean_C -> DASHDIETEXP,) (invmillssem -> DASHDIETEXP,) if finalsample==1 & sex==0 , nocapslatent method(ml)

estat gof, stats(all)
estat teffects

//WHITES//
sem (FCOSTEXP -> ALW4,) (DASHDIETEXP -> ALW4,) (Agew1_C-> ALW4,) (Agew3_C -> ALW4,) (sex -> ALW4,) (pir -> ALW4,) (edubr2 -> ALW4,) (edubr3 -> ALW4,) (employed1 -> ALW4,) (wrattbr2 -> ALW4,) (wrattbr3 -> ALW4,)(wrattbr4 -> ALW4,) (smoke1 -> ALW4,) (smoke9 -> ALW4,)(currdrugs1 -> ALW4,) (currdrugs9 -> ALW4,) (bmi_C -> ALW4,) (SRHbr2 -> ALW4,) (SRHbr3 -> ALW4,)(kcalw1w3mean_C -> ALW4,) (energystoresw1w3mean_C -> ALW4,) (invmillssem -> ALW4,) ///
(FCOSTEXP -> DASHDIETEXP,)(Agew1_C-> DASHDIETEXP,) (Agew3_C -> DASHDIETEXP,) (sex -> DASHDIETEXP,) (pir -> DASHDIETEXP,) (edubr2 -> DASHDIETEXP,) (edubr3 -> DASHDIETEXP,) (employed1 -> DASHDIETEXP,) (wrattbr2 -> DASHDIETEXP,) (wrattbr3 -> DASHDIETEXP,) (wrattbr4 -> DASHDIETEXP,)(smoke1 -> DASHDIETEXP,) (smoke9 -> DASHDIETEXP,)(currdrugs1 -> DASHDIETEXP,) (currdrugs9 -> DASHDIETEXP,) (bmi_C -> DASHDIETEXP,) (SRHbr2 -> DASHDIETEXP,) (SRHbr3 -> DASHDIETEXP,)(kcalw1w3mean_C -> DASHDIETEXP,) (energystoresw1w3mean_C -> DASHDIETEXP,) (invmillssem -> DASHDIETEXP,) if finalsample==1 & race==0 , nocapslatent method(ml)

estat gof, stats(all)
estat teffects

//African-Americans//

sem (FCOSTEXP -> ALW4,) (DASHDIETEXP -> ALW4,) (Agew1_C-> ALW4,) (Agew3_C -> ALW4,) (sex -> ALW4,) (pir -> ALW4,) (edubr2 -> ALW4,) (edubr3 -> ALW4,) (employed1 -> ALW4,) (wrattbr2 -> ALW4,) (wrattbr3 -> ALW4,) (wrattbr4 -> ALW4,)(smoke1 -> ALW4,) (smoke9 -> ALW4,)(currdrugs1 -> ALW4,) (currdrugs9 -> ALW4,) (bmi_C -> ALW4,) (SRHbr2 -> ALW4,) (SRHbr3 -> ALW4,)(kcalw1w3mean_C -> ALW4,) (energystoresw1w3mean_C -> ALW4,) (invmillssem -> ALW4,) ///

(FCOSTEXP -> DASHDIETEXP,)(Agew1_C-> DASHDIETEXP,) (Agew3_C -> DASHDIETEXP,) (sex -> DASHDIETEXP,) (pir -> DASHDIETEXP,) (edubr2 -> DASHDIETEXP,) (edubr3 -> DASHDIETEXP,) (employed1 -> DASHDIETEXP,) (wrattbr2 -> DASHDIETEXP,) (wrattbr3 -> DASHDIETEXP,) (wrattbr4 -> DASHDIETEXP,)(smoke1 -> DASHDIETEXP,) (smoke9 -> DASHDIETEXP,)(currdrugs1 -> DASHDIETEXP,) (currdrugs9 -> DASHDIETEXP,) (bmi_C -> DASHDIETEXP,) (SRHbr2 -> DASHDIETEXP,) (SRHbr3 -> DASHDIETEXP,)(kcalw1w3mean_C -> DASHDIETEXP,) (energystoresw1w3mean_C -> DASHDIETEXP,) (invmillssem -> DASHDIETEXP,) if finalsample==1 & race==1 , nocapslatent method(ml)

estat gof, stats(all)

estat teffects

//BELOW POVERTY//

sem (FCOSTEXP -> ALW4,) (DASHDIETEXP -> ALW4,) (Agew1_C-> ALW4,) (Agew3_C -> ALW4,) (sex -> ALW4,) (race -> ALW4,) (edubr2 -> ALW4,) (edubr3 -> ALW4,) (employed1 -> ALW4,) (wrattbr2 -> ALW4,) (wrattbr3 -> ALW4,) (wrattbr4 -> ALW4,)(smoke1 -> ALW4,) (smoke9 -> ALW4,)(currdrugs1 -> ALW4,) (currdrugs9 -> ALW4,) (bmi_C -> ALW4,) (SRHbr2 -> ALW4,) (SRHbr3 -> ALW4,)(kcalw1w3mean_C -> ALW4,) (energystoresw1w3mean_C -> ALW4,) (invmillssem -> ALW4,) ///

(FCOSTEXP -> DASHDIETEXP,)(Agew1_C-> DASHDIETEXP,) (Agew3_C -> DASHDIETEXP,) (sex -> DASHDIETEXP,) (race -> DASHDIETEXP,) (edubr2 -> DASHDIETEXP,) (edubr3 -> DASHDIETEXP,) (employed1 -> DASHDIETEXP,) (wrattbr2 -> DASHDIETEXP,) (wrattbr3 -> DASHDIETEXP,) (wrattbr4 -> DASHDIETEXP,)(smoke1 -> DASHDIETEXP,) (smoke9 -> DASHDIETEXP,)(currdrugs1 -> DASHDIETEXP,) (currdrugs9 -> DASHDIETEXP,) (bmi_C -> DASHDIETEXP,) (SRHbr2 -> DASHDIETEXP,) (SRHbr3 -> DASHDIETEXP,)(kcalw1w3mean_C -> DASHDIETEXP,) (energystoresw1w3mean_C -> DASHDIETEXP,) (invmillssem -> DASHDIETEXP,) if finalsample==1 & pir==0 , nocapslatent method(ml)

estat gof, stats(all)

estat teffects

//ABOVE POVERTY//

sem (FCOSTEXP -> ALW4,) (DASHDIETEXP -> ALW4,) (Agew1_C-> ALW4,) (Agew3_C -> ALW4,) (sex -> ALW4,) (race -> ALW4,) (edubr2 -> ALW4,) (edubr3 -> ALW4,) (employed1 -> ALW4,) (wrattbr2 -> ALW4,) (wrattbr3 -> ALW4,) (wrattbr4 -> ALW4,)(smoke1 -> ALW4,) (smoke9 -> ALW4,)(currdrugs1 -> ALW4,) (currdrugs9 -> ALW4,) (bmi_C -> ALW4,) (SRHbr2 -> ALW4,) (SRHbr3 -> ALW4,)(kcalw1w3mean_C -> ALW4,) (energystoresw1w3mean_C -> ALW4,) (invmillssem -> ALW4,) ///

(FCOSTEXP -> DASHDIETEXP,)(Agew1_C-> DASHDIETEXP,) (Agew3_C -> DASHDIETEXP,) (sex -> DASHDIETEXP,) (race -> DASHDIETEXP,) (edubr2 -> DASHDIETEXP,) (edubr3 -> DASHDIETEXP,) (employed1 -> DASHDIETEXP,)

(wrattbr2 -> DASHDIETEXP,) (wrattbr3 -> DASHDIETEXP,) (wrattbr4 -> DASHDIETEXP,)(smoke1 -> DASHDIETEXP,) (smoke9 -> DASHDIETEXP,)(currdrugs1 -> DASHDIETEXP,) (currdrugs9 -> DASHDIETEXP,) (bmi_C -> DASHDIETEXP,) (SRHbr2 -> DASHDIETEXP,) (SRHbr3 -> DASHDIETEXP,)(kcalw1w3mean_C -> DASHDIETEXP,) (energystoresw1w3mean_C -> DASHDIETEXP,) (invmillssem -> DASHDIETEXP,) if finalsample==1 & pir==1 , nocapslatent method(ml)

estat gof, stats(all)
estat teffects

****************************ALBUMIN WAVE 4***
//MULTI-GROUP//

All
sem (FCOSTEXP -> ALBUMINW4,) (DASHDIETEXP -> ALBUMINW4,) (Agew1_C-> ALBUMINW4,) (Agew3_C -> ALBUMINW4,) (sex -> ALBUMINW4,) (race -> ALBUMINW4,) (pir -> ALBUMINW4,) (edubr2 -> ALBUMINW4,) (edubr3 -> ALBUMINW4,) (employed1 -> ALBUMINW4,) (wrattbr2 -> ALBUMINW4,) (wrattbr3 -> ALBUMINW4,) (wrattbr4 -> ALBUMINW4,)(smoke1 -> ALBUMINW4,) (smoke9 -> ALBUMINW4,)(currdrugs1 -> ALBUMINW4,) (currdrugs9 -> ALBUMINW4,) (bmi_C -> ALBUMINW4,) (SRHbr2 -> ALBUMINW4,) (SRHbr3 -> ALBUMINW4,)(kcalw1w3mean_C -> ALBUMINW4,) (energystoresw1w3mean_C -> ALBUMINW4,) (invmillssem -> ALBUMINW4,) ///
(FCOSTEXP -> DASHDIETEXP,)(Agew1_C-> DASHDIETEXP,) (Agew3_C -> DASHDIETEXP,) (sex -> DASHDIETEXP,) (race -> DASHDIETEXP,) (pir -> DASHDIETEXP,) (edubr2 -> DASHDIETEXP,) (edubr3 -> DASHDIETEXP,) (employed1 -> DASHDIETEXP,) (wrattbr2 -> DASHDIETEXP,) (wrattbr3 -> DASHDIETEXP,) (wrattbr4 -> DASHDIETEXP,)(smoke1 -> DASHDIETEXP,) (smoke9 -> DASHDIETEXP,)(currdrugs1 -> DASHDIETEXP,) (currdrugs9 -> DASHDIETEXP,) (bmi_C -> DASHDIETEXP,) (SRHbr2 -> DASHDIETEXP,) (SRHbr3 -> DASHDIETEXP,)(kcalw1w3mean_C -> DASHDIETEXP,) (energystoresw1w3mean_C -> DASHDIETEXP,) (invmillssem -> DASHDIETEXP,) , nocapslatent method(ml) group(finalsample)

estat ginvariant

estat gof, stats(all)
estat teffects

By sex

sem (FCOSTEXP -> ALBUMINW4,) (DASHDIETEXP -> ALBUMINW4,) (Agew1_C-> ALBUMINW4,) (Agew3_C -> ALBUMINW4,) (race -> ALBUMINW4,) (pir -> ALBUMINW4,) (edubr2 -> ALBUMINW4,) (edubr3 -> ALBUMINW4,) (employed1 -> ALBUMINW4,) (wrattbr2 -> ALBUMINW4,) (wrattbr3 -> ALBUMINW4,) (wrattbr4 -> ALBUMINW4,)(smoke1 -> ALBUMINW4,) (smoke9 -> ALBUMINW4,)(currdrugs1 -> ALBUMINW4,) (currdrugs9 -> ALBUMINW4,) (bmi_C -> ALBUMINW4,) (SRHbr2 -> ALBUMINW4,) (SRHbr3 -> ALBUMINW4,)(kcalw1w3mean_C -> ALBUMINW4,) (energystoresw1w3mean_C -> ALBUMINW4,) (invmillssem -> ALBUMINW4,) ///
(FCOSTEXP -> DASHDIETEXP,)(Agew1_C-> DASHDIETEXP,) (Agew3_C -> DASHDIETEXP,) (race -> DASHDIETEXP,) (pir -> DASHDIETEXP,) (edubr2 -> DASHDIETEXP,) (edubr3 -> DASHDIETEXP,) (employed1 -> DASHDIETEXP,)

(wrattbr2 -> DASHDIETEXP,) (wrattbr3 -> DASHDIETEXP,)(wrattbr4 -> DASHDIETEXP,) (smoke1 -> DASHDIETEXP,) (smoke9 -> DASHDIETEXP,)(currdrugs1 -> DASHDIETEXP,) (currdrugs9 -> DASHDIETEXP,) (bmi_C -> DASHDIETEXP,) (SRHbr2 -> DASHDIETEXP,) (SRHbr3 -> DASHDIETEXP,)(kcalw1w3mean_C -> DASHDIETEXP,) (energystoresw1w3mean_C -> DASHDIETEXP,) (invmillssem -> DASHDIETEXP,) if finalsample==1 , nocapslatent method(ml) group(sex)

estat ginvariant

By race

sem (FCOSTEXP -> ALBUMINW4,) (DASHDIETEXP -> ALBUMINW4,) (Agew1_C-> ALBUMINW4,) (Agew3_C -> ALBUMINW4,) (sex -> ALBUMINW4,) (pir -> ALBUMINW4,) (edubr2 -> ALBUMINW4,) (edubr3 -> ALBUMINW4,) (employed1 -> ALBUMINW4,) (wrattbr2 -> ALBUMINW4,) (wrattbr3 -> ALBUMINW4,) (wrattbr4 -> ALBUMINW4,)(smoke1 -> ALBUMINW4,) (smoke9 -> ALBUMINW4,)(currdrugs1 -> ALBUMINW4,) (currdrugs9 -> ALBUMINW4,) (bmi_C -> ALBUMINW4,) (SRHbr2 -> ALBUMINW4,) (SRHbr3 -> ALBUMINW4,)(kcalw1w3mean_C -> ALBUMINW4,) (energystoresw1w3mean_C -> ALBUMINW4,) (invmillssem -> ALBUMINW4,) ///
(FCOSTEXP -> DASHDIETEXP,)(Agew1_C-> DASHDIETEXP,) (Agew3_C -> DASHDIETEXP,) (sex -> DASHDIETEXP,) (pir -> DASHDIETEXP,) (edubr2 -> DASHDIETEXP,) (edubr3 -> DASHDIETEXP,) (employed1 -> DASHDIETEXP,) (wrattbr2 -> DASHDIETEXP,) (wrattbr3 -> DASHDIETEXP,) (wrattbr4 -> DASHDIETEXP,)(smoke1 -> DASHDIETEXP,) (smoke9 -> DASHDIETEXP,)(currdrugs1 -> DASHDIETEXP,) (currdrugs9 -> DASHDIETEXP,) (bmi_C -> DASHDIETEXP,) (SRHbr2 -> DASHDIETEXP,) (SRHbr3 -> DASHDIETEXP,)(kcalw1w3mean_C -> DASHDIETEXP,) (energystoresw1w3mean_C -> DASHDIETEXP,) (invmillssem -> DASHDIETEXP,) if finalsample==1 , nocapslatent method(ml) group(race)

estat ginvariant

By pir

sem (FCOSTEXP -> ALBUMINW4,) (DASHDIETEXP -> ALBUMINW4,) (Agew1_C-> ALBUMINW4,) (Agew3_C -> ALBUMINW4,) (sex -> ALBUMINW4,) (race -> ALBUMINW4,) (edubr2 -> ALBUMINW4,) (edubr3 -> ALBUMINW4,) (employed1 -> ALBUMINW4,) (wrattbr2 -> ALBUMINW4,) (wrattbr3 -> ALBUMINW4,) (wrattbr4 -> ALBUMINW4,)(smoke1 -> ALBUMINW4,) (smoke9 -> ALBUMINW4,)(currdrugs1 -> ALBUMINW4,) (currdrugs9 -> ALBUMINW4,) (bmi_C -> ALBUMINW4,) (SRHbr2 -> ALBUMINW4,) (SRHbr3 -> ALBUMINW4,)(kcalw1w3mean_C -> ALBUMINW4,) (energystoresw1w3mean_C -> ALBUMINW4,) (invmillssem -> ALBUMINW4,) ///
(FCOSTEXP -> DASHDIETEXP,)(Agew1_C-> DASHDIETEXP,) (Agew3_C -> DASHDIETEXP,) (sex -> DASHDIETEXP,) (race -> DASHDIETEXP,) (edubr2 -> DASHDIETEXP,) (edubr3 -> DASHDIETEXP,) (employed1 -> DASHDIETEXP,) (wrattbr2 -> DASHDIETEXP,) (wrattbr3 -> DASHDIETEXP,) (wrattbr4 -> DASHDIETEXP,)(smoke1 -> DASHDIETEXP,) (smoke9 -> DASHDIETEXP,)(currdrugs1 -> DASHDIETEXP,) (currdrugs9 -> DASHDIETEXP,) (bmi_C -> DASHDIETEXP,) (SRHbr2 -> DASHDIETEXP,) (SRHbr3 -> DASHDIETEXP,)(kcalw1w3mean_C -> DASHDIETEXP,) (energystoresw1w3mean_C -> DASHDIETEXP,) (invmillssem -> DASHDIETEXP,) if finalsample==1 , nocapslatent method(ml) group(pir)

estat ginvariant

********************STRATIFIED ANALYSIS*******************************

//MEN//

sem (FCOSTEXP -> ALBUMINW4,) (DASHDIETEXP -> ALBUMINW4,) (Agew1_C-> ALBUMINW4,) (Agew3_C -> ALBUMINW4,) (race -> ALBUMINW4,) (pir -> ALBUMINW4,) (edubr2 -> ALBUMINW4,) (edubr3 -> ALBUMINW4,) (employed1 -> ALBUMINW4,) (wrattbr2 -> ALBUMINW4,) (wrattbr3 -> ALBUMINW4,) (wrattbr4 -> ALBUMINW4,)(smoke1 -> ALBUMINW4,) (smoke9 -> ALBUMINW4,)(currdrugs1 -> ALBUMINW4,) (currdrugs9 -> ALBUMINW4,) (bmi_C -> ALBUMINW4,) (SRHbr2 -> ALBUMINW4,) (SRHbr3 -> ALBUMINW4,)(kcalw1w3mean_C -> ALBUMINW4,) (energystoresw1w3mean_C -> ALBUMINW4,) (invmillssem -> ALBUMINW4,) ///
(FCOSTEXP -> DASHDIETEXP,)(Agew1_C-> DASHDIETEXP,) (Agew3_C -> DASHDIETEXP,) (race -> DASHDIETEXP,) (pir -> DASHDIETEXP,) (edubr2 -> DASHDIETEXP,) (edubr3 -> DASHDIETEXP,) (employed1 -> DASHDIETEXP,) (wrattbr2 -> DASHDIETEXP,) (wrattbr3 -> DASHDIETEXP,)(wrattbr4 -> DASHDIETEXP,) (smoke1 -> DASHDIETEXP,) (smoke9 -> DASHDIETEXP,)(currdrugs1 -> DASHDIETEXP,) (currdrugs9 -> DASHDIETEXP,) (bmi_C -> DASHDIETEXP,) (SRHbr2 -> DASHDIETEXP,) (SRHbr3 -> DASHDIETEXP,)(kcalw1w3mean_C -> DASHDIETEXP,) (energystoresw1w3mean_C -> DASHDIETEXP,) (invmillssem -> DASHDIETEXP,) if finalsample==1 & sex==1 , nocapslatent method(ml)

estat gof, stats(all)
estat teffects

//WOMEN//

sem (FCOSTEXP -> ALBUMINW4,) (DASHDIETEXP -> ALBUMINW4,) (Agew1_C-> ALBUMINW4,) (Agew3_C -> ALBUMINW4,) (race -> ALBUMINW4,) (pir -> ALBUMINW4,) (edubr2 -> ALBUMINW4,) (edubr3 -> ALBUMINW4,) (employed1 -> ALBUMINW4,) (wrattbr2 -> ALBUMINW4,) (wrattbr3 -> ALBUMINW4,) (wrattbr4 -> ALBUMINW4,)(smoke1 -> ALBUMINW4,) (smoke9 -> ALBUMINW4,)(currdrugs1 -> ALBUMINW4,) (currdrugs9 -> ALBUMINW4,) (bmi_C -> ALBUMINW4,) (SRHbr2 -> ALBUMINW4,) (SRHbr3 -> ALBUMINW4,)(kcalw1w3mean_C -> ALBUMINW4,) (energystoresw1w3mean_C -> ALBUMINW4,) (invmillssem -> ALBUMINW4,) ///
(FCOSTEXP -> DASHDIETEXP,)(Agew1_C-> DASHDIETEXP,) (Agew3_C -> DASHDIETEXP,) (race -> DASHDIETEXP,) (pir -> DASHDIETEXP,) (edubr2 -> DASHDIETEXP,) (edubr3 -> DASHDIETEXP,) (employed1 -> DASHDIETEXP,) (wrattbr2 -> DASHDIETEXP,) (wrattbr3 -> DASHDIETEXP,) (wrattbr4 -> DASHDIETEXP,)(smoke1 -> DASHDIETEXP,) (smoke9 -> DASHDIETEXP,)(currdrugs1 -> DASHDIETEXP,) (currdrugs9 -> DASHDIETEXP,) (bmi_C -> DASHDIETEXP,) (SRHbr2 -> DASHDIETEXP,) (SRHbr3 -> DASHDIETEXP,)(kcalw1w3mean_C -> DASHDIETEXP,) (energystoresw1w3mean_C -> DASHDIETEXP,) (invmillssem -> DASHDIETEXP,) if finalsample==1 & sex==0 , nocapslatent method(ml)

estat gof, stats(all)
estat teffects

//WHITES//

```
 sem (FCOSTEXP -> ALBUMINW4, ) (DASHDIETEXP -> ALBUMINW4, ) ( Agew1_C-> ALBUMINW4, ) (Agew3_C -> ALBUMINW4, ) (sex -> ALBUMINW4, ) (pir -> ALBUMINW4, ) (edubr2 -> ALBUMINW4, ) (edubr3 -> ALBUMINW4, ) (employed1 -> ALBUMINW4, )   (wrattbr2 -> ALBUMINW4, ) (wrattbr3 -> ALBUMINW4, )(wrattbr4 -> ALBUMINW4, ) (smoke1 -> ALBUMINW4, ) (smoke9 -> ALBUMINW4, )(currdrugs1 -> ALBUMINW4, ) (currdrugs9 -> ALBUMINW4, ) (bmi_C -> ALBUMINW4, ) (SRHbr2 -> ALBUMINW4, ) (SRHbr3 -> ALBUMINW4, )(kcalw1w3mean_C -> ALBUMINW4, ) (energystoresw1w3mean_C -> ALBUMINW4, ) (invmillssem -> ALBUMINW4, ) ///
(FCOSTEXP -> DASHDIETEXP, )( Agew1_C-> DASHDIETEXP, ) (Agew3_C -> DASHDIETEXP, ) (sex -> DASHDIETEXP, ) (pir -> DASHDIETEXP, ) (edubr2 -> DASHDIETEXP, ) (edubr3 -> DASHDIETEXP, ) (employed1 -> DASHDIETEXP, ) (wrattbr2 -> DASHDIETEXP, ) (wrattbr3 -> DASHDIETEXP, ) (wrattbr4 -> DASHDIETEXP, )(smoke1 -> DASHDIETEXP, ) (smoke9 -> DASHDIETEXP, )(currdrugs1 -> DASHDIETEXP, ) (currdrugs9 -> DASHDIETEXP, ) (bmi_C -> DASHDIETEXP, ) (SRHbr2 -> DASHDIETEXP, ) (SRHbr3 -> DASHDIETEXP, )(kcalw1w3mean_C -> DASHDIETEXP, ) (energystoresw1w3mean_C -> DASHDIETEXP, ) (invmillssem -> DASHDIETEXP, ) if finalsample==1 & race==0 , nocapslatent method(ml)

estat gof, stats(all)
estat teffects



//African-Americans//
 sem (FCOSTEXP -> ALBUMINW4, ) (DASHDIETEXP -> ALBUMINW4, ) ( Agew1_C-> ALBUMINW4, ) (Agew3_C -> ALBUMINW4, ) (sex -> ALBUMINW4, ) (pir -> ALBUMINW4, ) (edubr2 -> ALBUMINW4, ) (edubr3 -> ALBUMINW4, ) (employed1 -> ALBUMINW4, )   (wrattbr2 -> ALBUMINW4, ) (wrattbr3 -> ALBUMINW4, ) (wrattbr4 -> ALBUMINW4, )(smoke1 -> ALBUMINW4, ) (smoke9 -> ALBUMINW4, )(currdrugs1 -> ALBUMINW4, ) (currdrugs9 -> ALBUMINW4, ) (bmi_C -> ALBUMINW4, ) (SRHbr2 -> ALBUMINW4, ) (SRHbr3 -> ALBUMINW4, )(kcalw1w3mean_C -> ALBUMINW4, ) (energystoresw1w3mean_C -> ALBUMINW4, ) (invmillssem -> ALBUMINW4, ) ///
(FCOSTEXP -> DASHDIETEXP, )( Agew1_C-> DASHDIETEXP, ) (Agew3_C -> DASHDIETEXP, ) (sex -> DASHDIETEXP, ) (pir -> DASHDIETEXP, ) (edubr2 -> DASHDIETEXP, ) (edubr3 -> DASHDIETEXP, ) (employed1 -> DASHDIETEXP, ) (wrattbr2 -> DASHDIETEXP, ) (wrattbr3 -> DASHDIETEXP, ) (wrattbr4 -> DASHDIETEXP, )(smoke1 -> DASHDIETEXP, ) (smoke9 -> DASHDIETEXP, )(currdrugs1 -> DASHDIETEXP, ) (currdrugs9 -> DASHDIETEXP, ) (bmi_C -> DASHDIETEXP, ) (SRHbr2 -> DASHDIETEXP, ) (SRHbr3 -> DASHDIETEXP, )(kcalw1w3mean_C -> DASHDIETEXP, ) (energystoresw1w3mean_C -> DASHDIETEXP, ) (invmillssem -> DASHDIETEXP, ) if finalsample==1 & race==1 , nocapslatent method(ml)

estat gof, stats(all)
estat teffects



//BELOW POVERTY//
sem (FCOSTEXP -> ALBUMINW4, ) (DASHDIETEXP -> ALBUMINW4, ) ( Agew1_C-> ALBUMINW4, ) (Agew3_C -> ALBUMINW4, ) (sex -> ALBUMINW4, ) (race -> ALBUMINW4, ) (edubr2 -> ALBUMINW4, ) (edubr3 -> ALBUMINW4, ) (employed1 -> ALBUMINW4, )   (wrattbr2 -> ALBUMINW4, ) (wrattbr3 -> ALBUMINW4, ) (wrattbr4 -> ALBUMINW4, )(smoke1 -> ALBUMINW4, ) (smoke9 -> ALBUMINW4, )(currdrugs1 -> ALBUMINW4, ) (currdrugs9 -> ALBUMINW4, ) (bmi_C -> ALBUMINW4, ) (SRHbr2 -> ALBUMINW4, ) (SRHbr3 -> ALBUMINW4, )(kcalw1w3mean_C -> ALBUMINW4, ) (energystoresw1w3mean_C -> ALBUMINW4, ) (invmillssem -> ALBUMINW4, ) ///
```

(FCOSTEXP -> DASHDIETEXP,)(Agew1_C-> DASHDIETEXP,) (Agew3_C -> DASHDIETEXP,) (sex -> DASHDIETEXP,) (race -> DASHDIETEXP,) (edubr2 -> DASHDIETEXP,) (edubr3 -> DASHDIETEXP,) (employed1 -> DASHDIETEXP,) (wrattbr2 -> DASHDIETEXP,) (wrattbr3 -> DASHDIETEXP,) (wrattbr4 -> DASHDIETEXP,)(smoke1 -> DASHDIETEXP,) (smoke9 -> DASHDIETEXP,)(currdrugs1 -> DASHDIETEXP,) (currdrugs9 -> DASHDIETEXP,) (bmi_C -> DASHDIETEXP,) (SRHbr2 -> DASHDIETEXP,) (SRHbr3 -> DASHDIETEXP,)(kcalw1w3mean_C -> DASHDIETEXP,) (energystoresw1w3mean_C -> DASHDIETEXP,) (invmillssem -> DASHDIETEXP,) if finalsample==1 & pir==0 , nocapslatent method(ml)

estat gof, stats(all)
estat teffects

//ABOVE POVERTY//
sem (FCOSTEXP -> ALBUMINW4,) (DASHDIETEXP -> ALBUMINW4,) (Agew1_C-> ALBUMINW4,) (Agew3_C -> ALBUMINW4,) (sex -> ALBUMINW4,) (race -> ALBUMINW4,) (edubr2 -> ALBUMINW4,) (edubr3 -> ALBUMINW4,) (employed1 -> ALBUMINW4,) (wrattbr2 -> ALBUMINW4,) (wrattbr3 -> ALBUMINW4,) (wrattbr4 -> ALBUMINW4,)(smoke1 -> ALBUMINW4,) (smoke9 -> ALBUMINW4,)(currdrugs1 -> ALBUMINW4,) (currdrugs9 -> ALBUMINW4,) (bmi_C -> ALBUMINW4,) (SRHbr2 -> ALBUMINW4,) (SRHbr3 -> ALBUMINW4,)(kcalw1w3mean_C -> ALBUMINW4,) (energystoresw1w3mean_C -> ALBUMINW4,) (invmillssem -> ALBUMINW4,) ///
(FCOSTEXP -> DASHDIETEXP,)(Agew1_C-> DASHDIETEXP,) (Agew3_C -> DASHDIETEXP,) (sex -> DASHDIETEXP,) (race -> DASHDIETEXP,) (edubr2 -> DASHDIETEXP,) (edubr3 -> DASHDIETEXP,) (employed1 -> DASHDIETEXP,) (wrattbr2 -> DASHDIETEXP,) (wrattbr3 -> DASHDIETEXP,) (wrattbr4 -> DASHDIETEXP,)(smoke1 -> DASHDIETEXP,) (smoke9 -> DASHDIETEXP,)(currdrugs1 -> DASHDIETEXP,) (currdrugs9 -> DASHDIETEXP,) (bmi_C -> DASHDIETEXP,) (SRHbr2 -> DASHDIETEXP,) (SRHbr3 -> DASHDIETEXP,)(kcalw1w3mean_C -> DASHDIETEXP,) (energystoresw1w3mean_C -> DASHDIETEXP,) (invmillssem -> DASHDIETEXP,) if finalsample==1 & pir==1 , nocapslatent method(ml)

estat gof, stats(all)
estat teffects

***************************CRP WAVE 4***
//MULTI-GROUP//

All
sem (FCOSTEXP -> CRPW4,) (DASHDIETEXP -> CRPW4,) (Agew1_C-> CRPW4,) (Agew3_C -> CRPW4,) (sex -> CRPW4,) (race -> CRPW4,) (pir -> CRPW4,) (edubr2 -> CRPW4,) (edubr3 -> CRPW4,) (employed1 -> CRPW4,) (wrattbr2 -> CRPW4,) (wrattbr3 -> CRPW4,) (wrattbr4 -> CRPW4,)(smoke1 -> CRPW4,) (smoke9 -> CRPW4,)(currdrugs1 -> CRPW4,) (currdrugs9 -> CRPW4,) (bmi_C -> CRPW4,) (SRHbr2 -> CRPW4,) (SRHbr3 -> CRPW4,)(kcalw1w3mean_C -> CRPW4,) (energystoresw1w3mean_C -> CRPW4,) (invmillssem -> CRPW4,) ///
(FCOSTEXP -> DASHDIETEXP,)(Agew1_C-> DASHDIETEXP,) (Agew3_C -> DASHDIETEXP,) (sex -> DASHDIETEXP,) (race -> DASHDIETEXP,) (pir -> DASHDIETEXP,) (edubr2 -> DASHDIETEXP,) (edubr3 -> DASHDIETEXP,) (employed1 -> DASHDIETEXP,) (wrattbr2 -> DASHDIETEXP,) (wrattbr3 -> DASHDIETEXP,) (wrattbr4 -> DASHDIETEXP,)(smoke1 -> DASHDIETEXP,) (smoke9 -> DASHDIETEXP,)(currdrugs1 -> DASHDIETEXP,) (currdrugs9 -> DASHDIETEXP,) (bmi_C -> DASHDIETEXP,) (SRHbr2 -> DASHDIETEXP,) (SRHbr3 -> DASHDIETEXP,)(kcalw1w3mean_C -> DASHDIETEXP,)

(energystoresw1w3mean_C -> DASHDIETEXP,) (invmillssem -> DASHDIETEXP,) , nocapslatent method(ml) group(finalsample)

estat ginvariant

estat gof, stats(all)
estat teffects

By sex

sem (FCOSTEXP -> CRPW4,) (DASHDIETEXP -> CRPW4,) (Agew1_C-> CRPW4,) (Agew3_C -> CRPW4,) (race -> CRPW4,) (pir -> CRPW4,) (edubr2 -> CRPW4,) (edubr3 -> CRPW4,) (employed1 -> CRPW4,) (wrattbr2 -> CRPW4,) (wrattbr3 -> CRPW4,) (wrattbr4 -> CRPW4,)(smoke1 -> CRPW4,) (smoke9 -> CRPW4,)(currdrugs1 -> CRPW4,) (currdrugs9 -> CRPW4,) (bmi_C -> CRPW4,) (SRHbr2 -> CRPW4,) (SRHbr3 -> CRPW4,)(kcalw1w3mean_C -> CRPW4,) (energystoresw1w3mean_C -> CRPW4,) (invmillssem -> CRPW4,) ///
(FCOSTEXP -> DASHDIETEXP,)(Agew1_C-> DASHDIETEXP,) (Agew3_C -> DASHDIETEXP,) (race -> DASHDIETEXP,) (pir -> DASHDIETEXP,) (edubr2 -> DASHDIETEXP,) (edubr3 -> DASHDIETEXP,) (employed1 -> DASHDIETEXP,) (wrattbr2 -> DASHDIETEXP,) (wrattbr3 -> DASHDIETEXP,)(wrattbr4 -> DASHDIETEXP,) (smoke1 -> DASHDIETEXP,) (smoke9 -> DASHDIETEXP,)(currdrugs1 -> DASHDIETEXP,) (currdrugs9 -> DASHDIETEXP,) (bmi_C -> DASHDIETEXP,) (SRHbr2 -> DASHDIETEXP,) (SRHbr3 -> DASHDIETEXP,)(kcalw1w3mean_C -> DASHDIETEXP,) (energystoresw1w3mean_C -> DASHDIETEXP,) (invmillssem -> DASHDIETEXP,) if finalsample==1 , nocapslatent method(ml) group(sex)

estat ginvariant

By race

sem (FCOSTEXP -> CRPW4,) (DASHDIETEXP -> CRPW4,) (Agew1_C-> CRPW4,) (Agew3_C -> CRPW4,) (sex -> CRPW4,) (pir -> CRPW4,) (edubr2 -> CRPW4,) (edubr3 -> CRPW4,) (employed1 -> CRPW4,) (wrattbr2 -> CRPW4,) (wrattbr3 -> CRPW4,) (wrattbr4 -> CRPW4,)(smoke1 -> CRPW4,) (smoke9 -> CRPW4,)(currdrugs1 -> CRPW4,) (currdrugs9 -> CRPW4,) (bmi_C -> CRPW4,) (SRHbr2 -> CRPW4,) (SRHbr3 -> CRPW4,)(kcalw1w3mean_C -> CRPW4,) (energystoresw1w3mean_C -> CRPW4,) (invmillssem -> CRPW4,) ///
(FCOSTEXP -> DASHDIETEXP,)(Agew1_C-> DASHDIETEXP,) (Agew3_C -> DASHDIETEXP,) (sex -> DASHDIETEXP,) (pir -> DASHDIETEXP,) (edubr2 -> DASHDIETEXP,) (edubr3 -> DASHDIETEXP,) (employed1 -> DASHDIETEXP,) (wrattbr2 -> DASHDIETEXP,) (wrattbr3 -> DASHDIETEXP,) (wrattbr4 -> DASHDIETEXP,)(smoke1 -> DASHDIETEXP,) (smoke9 -> DASHDIETEXP,)(currdrugs1 -> DASHDIETEXP,) (currdrugs9 -> DASHDIETEXP,) (bmi_C -> DASHDIETEXP,) (SRHbr2 -> DASHDIETEXP,) (SRHbr3 -> DASHDIETEXP,)(kcalw1w3mean_C -> DASHDIETEXP,) (energystoresw1w3mean_C -> DASHDIETEXP,) (invmillssem -> DASHDIETEXP,) if finalsample==1 , nocapslatent method(ml) group(race)

```
estat ginvariant

**By pir**

sem (FCOSTEXP -> CRPW4, ) (DASHDIETEXP -> CRPW4, ) ( Agew1_C-> CRPW4, ) (Agew3_C -> CRPW4, ) (sex -> CRPW4, ) (race -> CRPW4, ) (edubr2 -> CRPW4, ) (edubr3 -> CRPW4, ) (employed1 -> CRPW4, )  (wrattbr2 -> CRPW4, ) (wrattbr3 -> CRPW4, ) (wrattbr4 -> CRPW4, )(smoke1 -> CRPW4, ) (smoke9 -> CRPW4, )(currdrugs1 -> CRPW4, ) (currdrugs9 -> CRPW4, ) (bmi_C -> CRPW4, ) (SRHbr2 -> CRPW4, ) (SRHbr3 -> CRPW4, )(kcalw1w3mean_C -> CRPW4, ) (energystoresw1w3mean_C -> CRPW4, ) (invmillssem -> CRPW4, ) ///
(FCOSTEXP -> DASHDIETEXP, )( Agew1_C-> DASHDIETEXP, ) (Agew3_C -> DASHDIETEXP, ) (sex -> DASHDIETEXP, ) (race -> DASHDIETEXP, )  (edubr2 -> DASHDIETEXP, ) (edubr3 -> DASHDIETEXP, ) (employed1 -> DASHDIETEXP, ) (wrattbr2 -> DASHDIETEXP, ) (wrattbr3 -> DASHDIETEXP, ) (wrattbr4 -> DASHDIETEXP, )(smoke1 -> DASHDIETEXP, ) (smoke9 -> DASHDIETEXP, )(currdrugs1 -> DASHDIETEXP, ) (currdrugs9 -> DASHDIETEXP, ) (bmi_C -> DASHDIETEXP, ) (SRHbr2 -> DASHDIETEXP, ) (SRHbr3 -> DASHDIETEXP, )(kcalw1w3mean_C -> DASHDIETEXP, ) (energystoresw1w3mean_C -> DASHDIETEXP, ) (invmillssem -> DASHDIETEXP, ) if finalsample==1 , nocapslatent method(ml) group(pir)

estat ginvariant



*********************STRATIFIED ANALYSIS********************************

//MEN//
sem (FCOSTEXP -> CRPW4, ) (DASHDIETEXP -> CRPW4, ) ( Agew1_C-> CRPW4, ) (Agew3_C -> CRPW4, ) (race -> CRPW4, ) (pir -> CRPW4, ) (edubr2 -> CRPW4, ) (edubr3 -> CRPW4, ) (employed1 -> CRPW4, )  (wrattbr2 -> CRPW4, ) (wrattbr3 -> CRPW4, ) (wrattbr4 -> CRPW4, )(smoke1 -> CRPW4, ) (smoke9 -> CRPW4, )(currdrugs1 -> CRPW4, ) (currdrugs9 -> CRPW4, ) (bmi_C -> CRPW4, ) (SRHbr2 -> CRPW4, ) (SRHbr3 -> CRPW4, )(kcalw1w3mean_C -> CRPW4, ) (energystoresw1w3mean_C -> CRPW4, ) (invmillssem -> CRPW4, ) ///
(FCOSTEXP -> DASHDIETEXP, )( Agew1_C-> DASHDIETEXP, ) (Agew3_C -> DASHDIETEXP, ) (race -> DASHDIETEXP, ) (pir -> DASHDIETEXP, ) (edubr2 -> DASHDIETEXP, ) (edubr3 -> DASHDIETEXP, ) (employed1 -> DASHDIETEXP, ) (wrattbr2 -> DASHDIETEXP, ) (wrattbr3 -> DASHDIETEXP, )(wrattbr4 -> DASHDIETEXP, ) (smoke1 -> DASHDIETEXP, ) (smoke9 -> DASHDIETEXP, )(currdrugs1 -> DASHDIETEXP, ) (currdrugs9 -> DASHDIETEXP, ) (bmi_C -> DASHDIETEXP, ) (SRHbr2 -> DASHDIETEXP, ) (SRHbr3 -> DASHDIETEXP, )(kcalw1w3mean_C -> DASHDIETEXP, ) (energystoresw1w3mean_C -> DASHDIETEXP, ) (invmillssem -> DASHDIETEXP, ) if finalsample==1 & sex==1 , nocapslatent method(ml)

estat gof, stats(all)
estat teffects



//WOMEN//
sem (FCOSTEXP -> CRPW4, ) (DASHDIETEXP -> CRPW4, ) ( Agew1_C-> CRPW4, ) (Agew3_C -> CRPW4, ) (race -> CRPW4, ) (pir -> CRPW4, ) (edubr2 -> CRPW4, ) (edubr3 -> CRPW4, ) (employed1 -> CRPW4, )  (wrattbr2 -> CRPW4, ) (wrattbr3 -> CRPW4, ) (wrattbr4 -> CRPW4, )(smoke1 -> CRPW4, ) (smoke9 -> CRPW4, )(currdrugs1 -> CRPW4, ) (currdrugs9 -> CRPW4, )
```

(bmi_C -> CRPW4,) (SRHbr2 -> CRPW4,) (SRHbr3 -> CRPW4,)(kcalw1w3mean_C -> CRPW4,) (energystoresw1w3mean_C -> CRPW4,) (invmillssem -> CRPW4,) ///
(FCOSTEXP -> DASHDIETEXP,)(Agew1_C-> DASHDIETEXP,) (Agew3_C -> DASHDIETEXP,) (race -> DASHDIETEXP,) (pir -> DASHDIETEXP,) (edubr2 -> DASHDIETEXP,) (edubr3 -> DASHDIETEXP,) (employed1 -> DASHDIETEXP,) (wrattbr2 -> DASHDIETEXP,) (wrattbr3 -> DASHDIETEXP,) (wrattbr4 -> DASHDIETEXP,)(smoke1 -> DASHDIETEXP,) (smoke9 -> DASHDIETEXP,)(currdrugs1 -> DASHDIETEXP,) (currdrugs9 -> DASHDIETEXP,) (bmi_C -> DASHDIETEXP,) (SRHbr2 -> DASHDIETEXP,) (SRHbr3 -> DASHDIETEXP,)(kcalw1w3mean_C -> DASHDIETEXP,) (energystoresw1w3mean_C -> DASHDIETEXP,) (invmillssem -> DASHDIETEXP,) if finalsample==1 & sex==0 , nocapslatent method(ml)

estat gof, stats(all)
estat teffects

//WHITES//
sem (FCOSTEXP -> CRPW4,) (DASHDIETEXP -> CRPW4,) (Agew1_C-> CRPW4,) (Agew3_C -> CRPW4,) (sex -> CRPW4,) (pir -> CRPW4,) (edubr2 -> CRPW4,) (edubr3 -> CRPW4,) (employed1 -> CRPW4,) (wrattbr2 -> CRPW4,) (wrattbr3 -> CRPW4,)(wrattbr4 -> CRPW4,) (smoke1 -> CRPW4,) (smoke9 -> CRPW4,)(currdrugs1 -> CRPW4,) (currdrugs9 -> CRPW4,) (bmi_C -> CRPW4,) (SRHbr2 -> CRPW4,) (SRHbr3 -> CRPW4,)(kcalw1w3mean_C -> CRPW4,) (energystoresw1w3mean_C -> CRPW4,) (invmillssem -> CRPW4,) ///
(FCOSTEXP -> DASHDIETEXP,)(Agew1_C-> DASHDIETEXP,) (Agew3_C -> DASHDIETEXP,) (sex -> DASHDIETEXP,) (pir -> DASHDIETEXP,) (edubr2 -> DASHDIETEXP,) (edubr3 -> DASHDIETEXP,) (employed1 -> DASHDIETEXP,) (wrattbr2 -> DASHDIETEXP,) (wrattbr3 -> DASHDIETEXP,) (wrattbr4 -> DASHDIETEXP,)(smoke1 -> DASHDIETEXP,) (smoke9 -> DASHDIETEXP,)(currdrugs1 -> DASHDIETEXP,) (currdrugs9 -> DASHDIETEXP,) (bmi_C -> DASHDIETEXP,) (SRHbr2 -> DASHDIETEXP,) (SRHbr3 -> DASHDIETEXP,)(kcalw1w3mean_C -> DASHDIETEXP,) (energystoresw1w3mean_C -> DASHDIETEXP,) (invmillssem -> DASHDIETEXP,) if finalsample==1 & race==0 , nocapslatent method(ml)

estat gof, stats(all)
estat teffects

//African-Americans//
sem (FCOSTEXP -> CRPW4,) (DASHDIETEXP -> CRPW4,) (Agew1_C-> CRPW4,) (Agew3_C -> CRPW4,) (sex -> CRPW4,) (pir -> CRPW4,) (edubr2 -> CRPW4,) (edubr3 -> CRPW4,) (employed1 -> CRPW4,) (wrattbr2 -> CRPW4,) (wrattbr3 -> CRPW4,) (wrattbr4 -> CRPW4,)(smoke1 -> CRPW4,) (smoke9 -> CRPW4,)(currdrugs1 -> CRPW4,) (currdrugs9 -> CRPW4,) (bmi_C -> CRPW4,) (SRHbr2 -> CRPW4,) (SRHbr3 -> CRPW4,)(kcalw1w3mean_C -> CRPW4,) (energystoresw1w3mean_C -> CRPW4,) (invmillssem -> CRPW4,) ///
(FCOSTEXP -> DASHDIETEXP,)(Agew1_C-> DASHDIETEXP,) (Agew3_C -> DASHDIETEXP,) (sex -> DASHDIETEXP,) (pir -> DASHDIETEXP,) (edubr2 -> DASHDIETEXP,) (edubr3 -> DASHDIETEXP,) (employed1 -> DASHDIETEXP,) (wrattbr2 -> DASHDIETEXP,) (wrattbr3 -> DASHDIETEXP,) (wrattbr4 -> DASHDIETEXP,)(smoke1 -> DASHDIETEXP,) (smoke9 -> DASHDIETEXP,)(currdrugs1 -> DASHDIETEXP,) (currdrugs9 -> DASHDIETEXP,) (bmi_C -> DASHDIETEXP,) (SRHbr2 -> DASHDIETEXP,) (SRHbr3 -> DASHDIETEXP,)(kcalw1w3mean_C -> DASHDIETEXP,)

(energystoresw1w3mean_C -> DASHDIETEXP,) (invmillssem -> DASHDIETEXP,) if finalsample==1 & race==1 , nocapslatent method(ml)

estat gof, stats(all)
estat teffects

//BELOW POVERTY//
sem (FCOSTEXP -> CRPW4,) (DASHDIETEXP -> CRPW4,) (Agew1_C-> CRPW4,) (Agew3_C -> CRPW4,) (sex -> CRPW4,) (race -> CRPW4,) (edubr2 -> CRPW4,) (edubr3 -> CRPW4,) (employed1 -> CRPW4,) (wrattbr2 -> CRPW4,) (wrattbr3 -> CRPW4,) (wrattbr4 -> CRPW4,)(smoke1 -> CRPW4,) (smoke9 -> CRPW4,)(currdrugs1 -> CRPW4,) (currdrugs9 -> CRPW4,) (bmi_C -> CRPW4,) (SRHbr2 -> CRPW4,) (SRHbr3 -> CRPW4,)(kcalw1w3mean_C -> CRPW4,) (energystoresw1w3mean_C -> CRPW4,) (invmillssem -> CRPW4,) ///
(FCOSTEXP -> DASHDIETEXP,)(Agew1_C-> DASHDIETEXP,) (Agew3_C -> DASHDIETEXP,) (sex -> DASHDIETEXP,) (race -> DASHDIETEXP,) (edubr2 -> DASHDIETEXP,) (edubr3 -> DASHDIETEXP,) (employed1 -> DASHDIETEXP,) (wrattbr2 -> DASHDIETEXP,) (wrattbr3 -> DASHDIETEXP,) (wrattbr4 -> DASHDIETEXP,)(smoke1 -> DASHDIETEXP,) (smoke9 -> DASHDIETEXP,)(currdrugs1 -> DASHDIETEXP,) (currdrugs9 -> DASHDIETEXP,) (bmi_C -> DASHDIETEXP,) (SRHbr2 -> DASHDIETEXP,) (SRHbr3 -> DASHDIETEXP,)(kcalw1w3mean_C -> DASHDIETEXP,) (energystoresw1w3mean_C -> DASHDIETEXP,) (invmillssem -> DASHDIETEXP,) if finalsample==1 & pir==0 , nocapslatent method(ml)

estat gof, stats(all)
estat teffects

//ABOVE POVERTY//
sem (FCOSTEXP -> CRPW4,) (DASHDIETEXP -> CRPW4,) (Agew1_C-> CRPW4,) (Agew3_C -> CRPW4,) (sex -> CRPW4,) (race -> CRPW4,) (edubr2 -> CRPW4,) (edubr3 -> CRPW4,) (employed1 -> CRPW4,) (wrattbr2 -> CRPW4,) (wrattbr3 -> CRPW4,) (wrattbr4 -> CRPW4,)(smoke1 -> CRPW4,) (smoke9 -> CRPW4,)(currdrugs1 -> CRPW4,) (currdrugs9 -> CRPW4,) (bmi_C -> CRPW4,) (SRHbr2 -> CRPW4,) (SRHbr3 -> CRPW4,)(kcalw1w3mean_C -> CRPW4,) (energystoresw1w3mean_C -> CRPW4,) (invmillssem -> CRPW4,) ///
(FCOSTEXP -> DASHDIETEXP,)(Agew1_C-> DASHDIETEXP,) (Agew3_C -> DASHDIETEXP,) (sex -> DASHDIETEXP,) (race -> DASHDIETEXP,) (edubr2 -> DASHDIETEXP,) (edubr3 -> DASHDIETEXP,) (employed1 -> DASHDIETEXP,) (wrattbr2 -> DASHDIETEXP,) (wrattbr3 -> DASHDIETEXP,) (wrattbr4 -> DASHDIETEXP,)(smoke1 -> DASHDIETEXP,) (smoke9 -> DASHDIETEXP,)(currdrugs1 -> DASHDIETEXP,) (currdrugs9 -> DASHDIETEXP,) (bmi_C -> DASHDIETEXP,) (SRHbr2 -> DASHDIETEXP,) (SRHbr3 -> DASHDIETEXP,)(kcalw1w3mean_C -> DASHDIETEXP,) (energystoresw1w3mean_C -> DASHDIETEXP,) (invmillssem -> DASHDIETEXP,) if finalsample==1 & pir==1 , nocapslatent method(ml)

estat gof, stats(all)
estat teffects

****************************CHOLESTEROL WAVE 4**

//MULTI-GROUP//

All

sem (FCOSTEXP -> CHOLW4,) (DASHDIETEXP -> CHOLW4,) (Agew1_C-> CHOLW4,) (Agew3_C -> CHOLW4,) (sex -> CHOLW4,) (race -> CHOLW4,) (pir -> CHOLW4,) (edubr2 -> CHOLW4,) (edubr3 -> CHOLW4,) (employed1 -> CHOLW4,) (wrattbr2 -> CHOLW4,) (wrattbr3 -> CHOLW4,) (wrattbr4 -> CHOLW4,)(smoke1 -> CHOLW4,) (smoke9 -> CHOLW4,)(currdrugs1 -> CHOLW4,) (currdrugs9 -> CHOLW4,) (bmi_C -> CHOLW4,) (SRHbr2 -> CHOLW4,) (SRHbr3 -> CHOLW4,)(kcalw1w3mean_C -> CHOLW4,) (energystoresw1w3mean_C -> CHOLW4,) (invmillssem -> CHOLW4,) /// (FCOSTEXP -> DASHDIETEXP,)(Agew1_C-> DASHDIETEXP,) (Agew3_C -> DASHDIETEXP,) (sex -> DASHDIETEXP,) (race -> DASHDIETEXP,) (pir -> DASHDIETEXP,) (edubr2 -> DASHDIETEXP,) (edubr3 -> DASHDIETEXP,) (employed1 -> DASHDIETEXP,) (wrattbr2 -> DASHDIETEXP,) (wrattbr3 -> DASHDIETEXP,) (wrattbr4 -> DASHDIETEXP,)(smoke1 -> DASHDIETEXP,) (smoke9 -> DASHDIETEXP,)(currdrugs1 -> DASHDIETEXP,) (currdrugs9 -> DASHDIETEXP,) (bmi_C -> DASHDIETEXP,) (SRHbr2 -> DASHDIETEXP,) (SRHbr3 -> DASHDIETEXP,)(kcalw1w3mean_C -> DASHDIETEXP,) (energystoresw1w3mean_C -> DASHDIETEXP,) (invmillssem -> DASHDIETEXP,) , nocapslatent method(ml) group(finalsample)

estat ginvariant

estat gof, stats(all)
estat teffects

By sex

sem (FCOSTEXP -> CHOLW4,) (DASHDIETEXP -> CHOLW4,) (Agew1_C-> CHOLW4,) (Agew3_C -> CHOLW4,) (race -> CHOLW4,) (pir -> CHOLW4,) (edubr2 -> CHOLW4,) (edubr3 -> CHOLW4,) (employed1 -> CHOLW4,) (wrattbr2 -> CHOLW4,) (wrattbr3 -> CHOLW4,) (wrattbr4 -> CHOLW4,)(smoke1 -> CHOLW4,) (smoke9 -> CHOLW4,)(currdrugs1 -> CHOLW4,) (currdrugs9 -> CHOLW4,) (bmi_C -> CHOLW4,) (SRHbr2 -> CHOLW4,) (SRHbr3 -> CHOLW4,)(kcalw1w3mean_C -> CHOLW4,) (energystoresw1w3mean_C -> CHOLW4,) (invmillssem -> CHOLW4,) /// (FCOSTEXP -> DASHDIETEXP,)(Agew1_C-> DASHDIETEXP,) (Agew3_C -> DASHDIETEXP,) (race -> DASHDIETEXP,) (pir -> DASHDIETEXP,) (edubr2 -> DASHDIETEXP,) (edubr3 -> DASHDIETEXP,) (employed1 -> DASHDIETEXP,) (wrattbr2 -> DASHDIETEXP,) (wrattbr3 -> DASHDIETEXP,)(wrattbr4 -> DASHDIETEXP,) (smoke1 -> DASHDIETEXP,) (smoke9 -> DASHDIETEXP,)(currdrugs1 -> DASHDIETEXP,) (currdrugs9 -> DASHDIETEXP,) (bmi_C -> DASHDIETEXP,) (SRHbr2 -> DASHDIETEXP,) (SRHbr3 -> DASHDIETEXP,)(kcalw1w3mean_C -> DASHDIETEXP,) (energystoresw1w3mean_C -> DASHDIETEXP,) (invmillssem -> DASHDIETEXP,) if finalsample==1 , nocapslatent method(ml) group(sex)

estat ginvariant

By race

```
sem (FCOSTEXP -> CHOLW4, ) (DASHDIETEXP -> CHOLW4, ) ( Agew1_C-> CHOLW4, ) (Agew3_C -> CHOLW4, ) (sex -> CHOLW4, ) (pir -> CHOLW4, ) (edubr2 -> CHOLW4, ) (edubr3 -> CHOLW4, ) (employed1 -> CHOLW4, )  (wrattbr2 -> CHOLW4, ) (wrattbr3 -> CHOLW4, ) (wrattbr4 -> CHOLW4, )(smoke1 -> CHOLW4, ) (smoke9 -> CHOLW4, )(currdrugs1 -> CHOLW4, ) (currdrugs9 -> CHOLW4, ) (bmi_C -> CHOLW4, ) (SRHbr2 -> CHOLW4, ) (SRHbr3 -> CHOLW4, )(kcalw1w3mean_C -> CHOLW4, ) (energystoresw1w3mean_C -> CHOLW4, ) (invmillssem -> CHOLW4, ) ///
(FCOSTEXP -> DASHDIETEXP, )( Agew1_C-> DASHDIETEXP, ) (Agew3_C -> DASHDIETEXP, ) (sex -> DASHDIETEXP, ) (pir -> DASHDIETEXP, ) (edubr2 -> DASHDIETEXP, ) (edubr3 -> DASHDIETEXP, ) (employed1 -> DASHDIETEXP, ) (wrattbr2 -> DASHDIETEXP, ) (wrattbr3 -> DASHDIETEXP, ) (wrattbr4 -> DASHDIETEXP, )(smoke1 -> DASHDIETEXP, ) (smoke9 -> DASHDIETEXP, )(currdrugs1 -> DASHDIETEXP, ) (currdrugs9 -> DASHDIETEXP, ) (bmi_C -> DASHDIETEXP, ) (SRHbr2 -> DASHDIETEXP, ) (SRHbr3 -> DASHDIETEXP, )(kcalw1w3mean_C -> DASHDIETEXP, ) (energystoresw1w3mean_C -> DASHDIETEXP, ) (invmillssem -> DASHDIETEXP, ) if finalsample==1 , nocapslatent method(ml) group(race)

estat ginvariant

**By pir**

sem (FCOSTEXP -> CHOLW4, ) (DASHDIETEXP -> CHOLW4, ) ( Agew1_C-> CHOLW4, ) (Agew3_C -> CHOLW4, ) (sex -> CHOLW4, ) (race -> CHOLW4, ) (edubr2 -> CHOLW4, ) (edubr3 -> CHOLW4, ) (employed1 -> CHOLW4, )  (wrattbr2 -> CHOLW4, ) (wrattbr3 -> CHOLW4, ) (wrattbr4 -> CHOLW4, )(smoke1 -> CHOLW4, ) (smoke9 -> CHOLW4, )(currdrugs1 -> CHOLW4, ) (currdrugs9 -> CHOLW4, ) (bmi_C -> CHOLW4, ) (SRHbr2 -> CHOLW4, ) (SRHbr3 -> CHOLW4, )(kcalw1w3mean_C -> CHOLW4, ) (energystoresw1w3mean_C -> CHOLW4, ) (invmillssem -> CHOLW4, ) ///
(FCOSTEXP -> DASHDIETEXP, )( Agew1_C-> DASHDIETEXP, ) (Agew3_C -> DASHDIETEXP, ) (sex -> DASHDIETEXP, ) (race -> DASHDIETEXP, )  (edubr2 -> DASHDIETEXP, ) (edubr3 -> DASHDIETEXP, ) (employed1 -> DASHDIETEXP, ) (wrattbr2 -> DASHDIETEXP, ) (wrattbr3 -> DASHDIETEXP, ) (wrattbr4 -> DASHDIETEXP, )(smoke1 -> DASHDIETEXP, ) (smoke9 -> DASHDIETEXP, )(currdrugs1 -> DASHDIETEXP, ) (currdrugs9 -> DASHDIETEXP, ) (bmi_C -> DASHDIETEXP, ) (SRHbr2 -> DASHDIETEXP, ) (SRHbr3 -> DASHDIETEXP, )(kcalw1w3mean_C -> DASHDIETEXP, ) (energystoresw1w3mean_C -> DASHDIETEXP, ) (invmillssem -> DASHDIETEXP, ) if finalsample==1 , nocapslatent method(ml) group(pir)

estat ginvariant

********************STRATIFIED ANALYSIS*******************************

//MEN//
sem (FCOSTEXP -> CHOLW4, ) (DASHDIETEXP -> CHOLW4, ) ( Agew1_C-> CHOLW4, ) (Agew3_C -> CHOLW4, ) (race -> CHOLW4, ) (pir -> CHOLW4, ) (edubr2 -> CHOLW4, ) (edubr3 -> CHOLW4, ) (employed1 -> CHOLW4, )  (wrattbr2 -> CHOLW4, ) (wrattbr3 -> CHOLW4, ) (wrattbr4 -> CHOLW4, )(smoke1 -> CHOLW4, ) (smoke9 -> CHOLW4, )(currdrugs1 -> CHOLW4, ) (currdrugs9 -> CHOLW4, ) (bmi_C -> CHOLW4, ) (SRHbr2 -> CHOLW4, ) (SRHbr3 -> CHOLW4, )(kcalw1w3mean_C -> CHOLW4, ) (energystoresw1w3mean_C -> CHOLW4, ) (invmillssem -> CHOLW4, ) ///
(FCOSTEXP -> DASHDIETEXP, )( Agew1_C-> DASHDIETEXP, ) (Agew3_C -> DASHDIETEXP, ) (race -> DASHDIETEXP, ) (pir -> DASHDIETEXP, ) (edubr2 -> DASHDIETEXP, ) (edubr3 -> DASHDIETEXP, ) (employed1 -> DASHDIETEXP, ) (wrattbr2 -> DASHDIETEXP, ) (wrattbr3 -> DASHDIETEXP, )(wrattbr4 -> DASHDIETEXP, ) (smoke1 -> DASHDIETEXP, )
```

(smoke9 -> DASHDIETEXP,)(currdrugs1 -> DASHDIETEXP,) (currdrugs9 -> DASHDIETEXP,) (bmi_C -> DASHDIETEXP,) (SRHbr2 -> DASHDIETEXP,) (SRHbr3 -> DASHDIETEXP,)(kcalw1w3mean_C -> DASHDIETEXP,) (energystoresw1w3mean_C -> DASHDIETEXP,) (invmillssem -> DASHDIETEXP,) if finalsample==1 & sex==1 , nocapslatent method(ml)

estat gof, stats(all)
estat teffects

//WOMEN//
sem (FCOSTEXP -> CHOLW4,) (DASHDIETEXP -> CHOLW4,) (Agew1_C-> CHOLW4,) (Agew3_C -> CHOLW4,) (race -> CHOLW4,) (pir -> CHOLW4,) (edubr2 -> CHOLW4,) (edubr3 -> CHOLW4,) (employed1 -> CHOLW4,) (wrattbr2 -> CHOLW4,) (wrattbr3 -> CHOLW4,) (wrattbr4 -> CHOLW4,)(smoke1 -> CHOLW4,) (smoke9 -> CHOLW4,)(currdrugs1 -> CHOLW4,) (currdrugs9 -> CHOLW4,) (bmi_C -> CHOLW4,) (SRHbr2 -> CHOLW4,) (SRHbr3 -> CHOLW4,)(kcalw1w3mean_C -> CHOLW4,) (energystoresw1w3mean_C -> CHOLW4,) (invmillssem -> CHOLW4,) ///
(FCOSTEXP -> DASHDIETEXP,)(Agew1_C-> DASHDIETEXP,) (Agew3_C -> DASHDIETEXP,) (race -> DASHDIETEXP,) (pir -> DASHDIETEXP,) (edubr2 -> DASHDIETEXP,) (edubr3 -> DASHDIETEXP,) (employed1 -> DASHDIETEXP,) (wrattbr2 -> DASHDIETEXP,) (wrattbr3 -> DASHDIETEXP,) (wrattbr4 -> DASHDIETEXP,)(smoke1 -> DASHDIETEXP,) (smoke9 -> DASHDIETEXP,)(currdrugs1 -> DASHDIETEXP,) (currdrugs9 -> DASHDIETEXP,) (bmi_C -> DASHDIETEXP,) (SRHbr2 -> DASHDIETEXP,) (SRHbr3 -> DASHDIETEXP,)(kcalw1w3mean_C -> DASHDIETEXP,) (energystoresw1w3mean_C -> DASHDIETEXP,) (invmillssem -> DASHDIETEXP,) if finalsample==1 & sex==0 , nocapslatent method(ml)

estat gof, stats(all)
estat teffects

//WHITES//
sem (FCOSTEXP -> CHOLW4,) (DASHDIETEXP -> CHOLW4,) (Agew1_C-> CHOLW4,) (Agew3_C -> CHOLW4,) (sex -> CHOLW4,) (pir -> CHOLW4,) (edubr2 -> CHOLW4,) (edubr3 -> CHOLW4,) (employed1 -> CHOLW4,) (wrattbr2 -> CHOLW4,) (wrattbr3 -> CHOLW4,)(wrattbr4 -> CHOLW4,) (smoke1 -> CHOLW4,) (smoke9 -> CHOLW4,)(currdrugs1 -> CHOLW4,) (currdrugs9 -> CHOLW4,) (bmi_C -> CHOLW4,) (SRHbr2 -> CHOLW4,) (SRHbr3 -> CHOLW4,)(kcalw1w3mean_C -> CHOLW4,) (energystoresw1w3mean_C -> CHOLW4,) (invmillssem -> CHOLW4,) ///
(FCOSTEXP -> DASHDIETEXP,)(Agew1_C-> DASHDIETEXP,) (Agew3_C -> DASHDIETEXP,) (sex -> DASHDIETEXP,) (pir -> DASHDIETEXP,) (edubr2 -> DASHDIETEXP,) (edubr3 -> DASHDIETEXP,) (employed1 -> DASHDIETEXP,) (wrattbr2 -> DASHDIETEXP,) (wrattbr3 -> DASHDIETEXP,) (wrattbr4 -> DASHDIETEXP,)(smoke1 -> DASHDIETEXP,) (smoke9 -> DASHDIETEXP,)(currdrugs1 -> DASHDIETEXP,) (currdrugs9 -> DASHDIETEXP,) (bmi_C -> DASHDIETEXP,) (SRHbr2 -> DASHDIETEXP,) (SRHbr3 -> DASHDIETEXP,)(kcalw1w3mean_C -> DASHDIETEXP,) (energystoresw1w3mean_C -> DASHDIETEXP,) (invmillssem -> DASHDIETEXP,) if finalsample==1 & race==0 , nocapslatent method(ml)

estat gof, stats(all)
estat teffects

//African-Americans//

sem (FCOSTEXP -> CHOLW4,) (DASHDIETEXP -> CHOLW4,) (Agew1_C-> CHOLW4,) (Agew3_C -> CHOLW4,) (sex -> CHOLW4,) (pir -> CHOLW4,) (edubr2 -> CHOLW4,) (edubr3 -> CHOLW4,) (employed1 -> CHOLW4,) (wrattbr2 -> CHOLW4,) (wrattbr3 -> CHOLW4,) (wrattbr4 -> CHOLW4,)(smoke1 -> CHOLW4,) (smoke9 -> CHOLW4,)(currdrugs1 -> CHOLW4,) (currdrugs9 -> CHOLW4,) (bmi_C -> CHOLW4,) (SRHbr2 -> CHOLW4,) (SRHbr3 -> CHOLW4,)(kcalw1w3mean_C -> CHOLW4,) (energystoresw1w3mean_C -> CHOLW4,) (invmillssem -> CHOLW4,) /// (FCOSTEXP -> DASHDIETEXP,)(Agew1_C-> DASHDIETEXP,) (Agew3_C -> DASHDIETEXP,) (sex -> DASHDIETEXP,) (pir -> DASHDIETEXP,) (edubr2 -> DASHDIETEXP,) (edubr3 -> DASHDIETEXP,) (employed1 -> DASHDIETEXP,) (wrattbr2 -> DASHDIETEXP,) (wrattbr3 -> DASHDIETEXP,) (wrattbr4 -> DASHDIETEXP,)(smoke1 -> DASHDIETEXP,) (smoke9 -> DASHDIETEXP,)(currdrugs1 -> DASHDIETEXP,) (currdrugs9 -> DASHDIETEXP,) (bmi_C -> DASHDIETEXP,) (SRHbr2 -> DASHDIETEXP,) (SRHbr3 -> DASHDIETEXP,)(kcalw1w3mean_C -> DASHDIETEXP,) (energystoresw1w3mean_C -> DASHDIETEXP,) (invmillssem -> DASHDIETEXP,) if finalsample==1 & race==1 , nocapslatent method(ml)

estat gof, stats(all)

estat teffects

//BELOW POVERTY//

sem (FCOSTEXP -> CHOLW4,) (DASHDIETEXP -> CHOLW4,) (Agew1_C-> CHOLW4,) (Agew3_C -> CHOLW4,) (sex -> CHOLW4,) (race -> CHOLW4,) (edubr2 -> CHOLW4,) (edubr3 -> CHOLW4,) (employed1 -> CHOLW4,) (wrattbr2 -> CHOLW4,) (wrattbr3 -> CHOLW4,) (wrattbr4 -> CHOLW4,)(smoke1 -> CHOLW4,) (smoke9 -> CHOLW4,)(currdrugs1 -> CHOLW4,) (currdrugs9 -> CHOLW4,) (bmi_C -> CHOLW4,) (SRHbr2 -> CHOLW4,) (SRHbr3 -> CHOLW4,)(kcalw1w3mean_C -> CHOLW4,) (energystoresw1w3mean_C -> CHOLW4,) (invmillssem -> CHOLW4,) /// (FCOSTEXP -> DASHDIETEXP,)(Agew1_C-> DASHDIETEXP,) (Agew3_C -> DASHDIETEXP,) (sex -> DASHDIETEXP,) (race -> DASHDIETEXP,) (edubr2 -> DASHDIETEXP,) (edubr3 -> DASHDIETEXP,) (employed1 -> DASHDIETEXP,) (wrattbr2 -> DASHDIETEXP,) (wrattbr3 -> DASHDIETEXP,) (wrattbr4 -> DASHDIETEXP,)(smoke1 -> DASHDIETEXP,) (smoke9 -> DASHDIETEXP,)(currdrugs1 -> DASHDIETEXP,) (currdrugs9 -> DASHDIETEXP,) (bmi_C -> DASHDIETEXP,) (SRHbr2 -> DASHDIETEXP,) (SRHbr3 -> DASHDIETEXP,)(kcalw1w3mean_C -> DASHDIETEXP,) (energystoresw1w3mean_C -> DASHDIETEXP,) (invmillssem -> DASHDIETEXP,) if finalsample==1 & pir==0 , nocapslatent method(ml)

estat gof, stats(all)

estat teffects

//ABOVE POVERTY//

sem (FCOSTEXP -> CHOLW4,) (DASHDIETEXP -> CHOLW4,) (Agew1_C-> CHOLW4,) (Agew3_C -> CHOLW4,) (sex -> CHOLW4,) (race -> CHOLW4,) (edubr2 -> CHOLW4,) (edubr3 -> CHOLW4,) (employed1 -> CHOLW4,) (wrattbr2 -> CHOLW4,) (wrattbr3 -> CHOLW4,) (wrattbr4 -> CHOLW4,)(smoke1 -> CHOLW4,) (smoke9 -> CHOLW4,)(currdrugs1 -> CHOLW4,) (currdrugs9 -> CHOLW4,) (bmi_C -> CHOLW4,) (SRHbr2 -> CHOLW4,) (SRHbr3 -> CHOLW4,)(kcalw1w3mean_C -> CHOLW4,) (energystoresw1w3mean_C -> CHOLW4,) (invmillssem -> CHOLW4,) /// (FCOSTEXP -> DASHDIETEXP,)(Agew1_C-> DASHDIETEXP,) (Agew3_C -> DASHDIETEXP,) (sex -> DASHDIETEXP,) (race -> DASHDIETEXP,) (edubr2 -> DASHDIETEXP,) (edubr3 -> DASHDIETEXP,) (employed1 -> DASHDIETEXP,)

(wrattbr2 -> DASHDIETEXP,) (wrattbr3 -> DASHDIETEXP,) (wrattbr4 -> DASHDIETEXP,)(smoke1 -> DASHDIETEXP,) (smoke9 -> DASHDIETEXP,)(currdrugs1 -> DASHDIETEXP,) (currdrugs9 -> DASHDIETEXP,) (bmi_C -> DASHDIETEXP,) (SRHbr2 -> DASHDIETEXP,) (SRHbr3 -> DASHDIETEXP,)(kcalw1w3mean_C -> DASHDIETEXP,) (energystoresw1w3mean_C -> DASHDIETEXP,) (invmillssem -> DASHDIETEXP,) if finalsample==1 & pir==1 , nocapslatent method(ml)

estat gof, stats(all)
estat teffects

****************************HDL WAVE 4**
//MULTI-GROUP//

All
sem (FCOSTEXP -> HDLW4,) (DASHDIETEXP -> HDLW4,) (Agew1_C-> HDLW4,) (Agew3_C -> HDLW4,) (sex -> HDLW4,) (race -> HDLW4,) (pir -> HDLW4,) (edubr2 -> HDLW4,) (edubr3 -> HDLW4,) (employed1 -> HDLW4,) (wrattbr2 -> HDLW4,) (wrattbr3 -> HDLW4,) (wrattbr4 -> HDLW4,)(smoke1 -> HDLW4,) (smoke9 -> HDLW4,)(currdrugs1 -> HDLW4,) (currdrugs9 -> HDLW4,) (bmi_C -> HDLW4,) (SRHbr2 -> HDLW4,) (SRHbr3 -> HDLW4,)(kcalw1w3mean_C -> HDLW4,) (energystoresw1w3mean_C -> HDLW4,) (invmillssem -> HDLW4,) ///
(FCOSTEXP -> DASHDIETEXP,)(Agew1_C-> DASHDIETEXP,) (Agew3_C -> DASHDIETEXP,) (sex -> DASHDIETEXP,) (race -> DASHDIETEXP,) (pir -> DASHDIETEXP,) (edubr2 -> DASHDIETEXP,) (edubr3 -> DASHDIETEXP,) (employed1 -> DASHDIETEXP,) (wrattbr2 -> DASHDIETEXP,) (wrattbr3 -> DASHDIETEXP,) (wrattbr4 -> DASHDIETEXP,)(smoke1 -> DASHDIETEXP,) (smoke9 -> DASHDIETEXP,)(currdrugs1 -> DASHDIETEXP,) (currdrugs9 -> DASHDIETEXP,) (bmi_C -> DASHDIETEXP,) (SRHbr2 -> DASHDIETEXP,) (SRHbr3 -> DASHDIETEXP,)(kcalw1w3mean_C -> DASHDIETEXP,) (energystoresw1w3mean_C -> DASHDIETEXP,) (invmillssem -> DASHDIETEXP,) , nocapslatent method(ml) group(finalsample)

estat ginvariant

estat gof, stats(all)
estat teffects

By sex

sem (FCOSTEXP -> HDLW4,) (DASHDIETEXP -> HDLW4,) (Agew1_C-> HDLW4,) (Agew3_C -> HDLW4,) (race -> HDLW4,) (pir -> HDLW4,) (edubr2 -> HDLW4,) (edubr3 -> HDLW4,) (employed1 -> HDLW4,) (wrattbr2 -> HDLW4,) (wrattbr3 -> HDLW4,) (wrattbr4 -> HDLW4,)(smoke1 -> HDLW4,) (smoke9 -> HDLW4,)(currdrugs1 -> HDLW4,) (currdrugs9 -> HDLW4,) (bmi_C -> HDLW4,) (SRHbr2 -> HDLW4,) (SRHbr3 -> HDLW4,)(kcalw1w3mean_C -> HDLW4,) (energystoresw1w3mean_C -> HDLW4,) (invmillssem -> HDLW4,) ///

(FCOSTEXP -> DASHDIETEXP,)(Agew1_C-> DASHDIETEXP,) (Agew3_C -> DASHDIETEXP,) (race -> DASHDIETEXP,) (pir -> DASHDIETEXP,) (edubr2 -> DASHDIETEXP,) (edubr3 -> DASHDIETEXP,) (employed1 -> DASHDIETEXP,) (wrattbr2 -> DASHDIETEXP,) (wrattbr3 -> DASHDIETEXP,)(wrattbr4 -> DASHDIETEXP,) (smoke1 -> DASHDIETEXP,) (smoke9 -> DASHDIETEXP,)(currdrugs1 -> DASHDIETEXP,) (currdrugs9 -> DASHDIETEXP,) (bmi_C -> DASHDIETEXP,) (SRHbr2 -> DASHDIETEXP,) (SRHbr3 -> DASHDIETEXP,)(kcalw1w3mean_C -> DASHDIETEXP,) (energystoresw1w3mean_C -> DASHDIETEXP,) (invmillssem -> DASHDIETEXP,) if finalsample==1 , nocapslatent method(ml) group(sex)

estat ginvariant

By race

sem (FCOSTEXP -> HDLW4,) (DASHDIETEXP -> HDLW4,) (Agew1_C-> HDLW4,) (Agew3_C -> HDLW4,) (sex -> HDLW4,) (pir -> HDLW4,) (edubr2 -> HDLW4,) (edubr3 -> HDLW4,) (employed1 -> HDLW4,) (wrattbr2 -> HDLW4,) (wrattbr3 -> HDLW4,) (wrattbr4 -> HDLW4,)(smoke1 -> HDLW4,) (smoke9 -> HDLW4,)(currdrugs1 -> HDLW4,) (currdrugs9 -> HDLW4,) (bmi_C -> HDLW4,) (SRHbr2 -> HDLW4,) (SRHbr3 -> HDLW4,)(kcalw1w3mean_C -> HDLW4,) (energystoresw1w3mean_C -> HDLW4,) (invmillssem -> HDLW4,) ///
(FCOSTEXP -> DASHDIETEXP,)(Agew1_C-> DASHDIETEXP,) (Agew3_C -> DASHDIETEXP,) (sex -> DASHDIETEXP,) (pir -> DASHDIETEXP,) (edubr2 -> DASHDIETEXP,) (edubr3 -> DASHDIETEXP,) (employed1 -> DASHDIETEXP,) (wrattbr2 -> DASHDIETEXP,) (wrattbr3 -> DASHDIETEXP,) (wrattbr4 -> DASHDIETEXP,)(smoke1 -> DASHDIETEXP,) (smoke9 -> DASHDIETEXP,)(currdrugs1 -> DASHDIETEXP,) (currdrugs9 -> DASHDIETEXP,) (bmi_C -> DASHDIETEXP,) (SRHbr2 -> DASHDIETEXP,) (SRHbr3 -> DASHDIETEXP,)(kcalw1w3mean_C -> DASHDIETEXP,) (energystoresw1w3mean_C -> DASHDIETEXP,) (invmillssem -> DASHDIETEXP,) if finalsample==1 , nocapslatent method(ml) group(race)

estat ginvariant

By pir

sem (FCOSTEXP -> HDLW4,) (DASHDIETEXP -> HDLW4,) (Agew1_C-> HDLW4,) (Agew3_C -> HDLW4,) (sex -> HDLW4,) (race -> HDLW4,) (edubr2 -> HDLW4,) (edubr3 -> HDLW4,) (employed1 -> HDLW4,) (wrattbr2 -> HDLW4,) (wrattbr3 -> HDLW4,) (wrattbr4 -> HDLW4,)(smoke1 -> HDLW4,) (smoke9 -> HDLW4,)(currdrugs1 -> HDLW4,) (currdrugs9 -> HDLW4,) (bmi_C -> HDLW4,) (SRHbr2 -> HDLW4,) (SRHbr3 -> HDLW4,)(kcalw1w3mean_C -> HDLW4,) (energystoresw1w3mean_C -> HDLW4,) (invmillssem -> HDLW4,) ///
(FCOSTEXP -> DASHDIETEXP,)(Agew1_C-> DASHDIETEXP,) (Agew3_C -> DASHDIETEXP,) (sex -> DASHDIETEXP,) (race -> DASHDIETEXP,) (edubr2 -> DASHDIETEXP,) (edubr3 -> DASHDIETEXP,) (employed1 -> DASHDIETEXP,) (wrattbr2 -> DASHDIETEXP,) (wrattbr3 -> DASHDIETEXP,) (wrattbr4 -> DASHDIETEXP,)(smoke1 -> DASHDIETEXP,) (smoke9 -> DASHDIETEXP,)(currdrugs1 -> DASHDIETEXP,) (currdrugs9 -> DASHDIETEXP,) (bmi_C -> DASHDIETEXP,) (SRHbr2 -> DASHDIETEXP,) (SRHbr3 -> DASHDIETEXP,)(kcalw1w3mean_C -> DASHDIETEXP,) (energystoresw1w3mean_C -> DASHDIETEXP,) (invmillssem -> DASHDIETEXP,) if finalsample==1 , nocapslatent method(ml) group(pir)

estat ginvariant

```
********************STRATIFIED ANALYSIS********************************

//MEN//
sem (FCOSTEXP -> HDLW4, ) (DASHDIETEXP -> HDLW4, ) ( Agew1_C-> HDLW4, ) (Agew3_C -> HDLW4, ) (race -> HDLW4, )
(pir -> HDLW4, ) (edubr2 -> HDLW4, ) (edubr3 -> HDLW4, ) (employed1 -> HDLW4, )    (wrattbr2 -> HDLW4, ) (wrattbr3 ->
HDLW4, ) (wrattbr4 -> HDLW4, )(smoke1 -> HDLW4, ) (smoke9 -> HDLW4, )(currdrugs1 -> HDLW4, ) (currdrugs9 ->
HDLW4, ) (bmi_C -> HDLW4, ) (SRHbr2 -> HDLW4, ) (SRHbr3 -> HDLW4, )(kcalw1w3mean_C -> HDLW4, )
(energystoresw1w3mean_C -> HDLW4, ) (invmillssem -> HDLW4, ) ///
(FCOSTEXP -> DASHDIETEXP, )( Agew1_C-> DASHDIETEXP, ) (Agew3_C -> DASHDIETEXP, ) (race -> DASHDIETEXP, )
(pir -> DASHDIETEXP, ) (edubr2 -> DASHDIETEXP, ) (edubr3 -> DASHDIETEXP, ) (employed1 -> DASHDIETEXP, )
(wrattbr2 -> DASHDIETEXP, ) (wrattbr3 -> DASHDIETEXP, )(wrattbr4 -> DASHDIETEXP, ) (smoke1 -> DASHDIETEXP, )
(smoke9 -> DASHDIETEXP, )(currdrugs1 -> DASHDIETEXP, ) (currdrugs9 -> DASHDIETEXP, ) (bmi_C -> DASHDIETEXP, )
(SRHbr2 -> DASHDIETEXP, ) (SRHbr3 -> DASHDIETEXP, )(kcalw1w3mean_C -> DASHDIETEXP, )
(energystoresw1w3mean_C -> DASHDIETEXP, ) (invmillssem -> DASHDIETEXP, ) if finalsample==1 & sex==1 , nocapslatent
method(ml)

estat gof, stats(all)
estat teffects



//WOMEN//
sem (FCOSTEXP -> HDLW4, ) (DASHDIETEXP -> HDLW4, ) ( Agew1_C-> HDLW4, ) (Agew3_C -> HDLW4, ) (race -> HDLW4, )
(pir -> HDLW4, ) (edubr2 -> HDLW4, ) (edubr3 -> HDLW4, ) (employed1 -> HDLW4, )    (wrattbr2 -> HDLW4, ) (wrattbr3 ->
HDLW4, ) (wrattbr4 -> HDLW4, )(smoke1 -> HDLW4, ) (smoke9 -> HDLW4, )(currdrugs1 -> HDLW4, ) (currdrugs9 ->
HDLW4, ) (bmi_C -> HDLW4, ) (SRHbr2 -> HDLW4, ) (SRHbr3 -> HDLW4, )(kcalw1w3mean_C -> HDLW4, )
(energystoresw1w3mean_C -> HDLW4, ) (invmillssem -> HDLW4, ) ///
(FCOSTEXP -> DASHDIETEXP, )( Agew1_C-> DASHDIETEXP, ) (Agew3_C -> DASHDIETEXP, ) (race -> DASHDIETEXP, )
(pir -> DASHDIETEXP, ) (edubr2 -> DASHDIETEXP, ) (edubr3 -> DASHDIETEXP, ) (employed1 -> DASHDIETEXP, )
(wrattbr2 -> DASHDIETEXP, ) (wrattbr3 -> DASHDIETEXP, ) (wrattbr4 -> DASHDIETEXP, )(smoke1 -> DASHDIETEXP, )
(smoke9 -> DASHDIETEXP, )(currdrugs1 -> DASHDIETEXP, ) (currdrugs9 -> DASHDIETEXP, ) (bmi_C -> DASHDIETEXP, )
(SRHbr2 -> DASHDIETEXP, ) (SRHbr3 -> DASHDIETEXP, )(kcalw1w3mean_C -> DASHDIETEXP, )
(energystoresw1w3mean_C -> DASHDIETEXP, ) (invmillssem -> DASHDIETEXP, ) if finalsample==1 & sex==0 , nocapslatent
method(ml)

estat gof, stats(all)
estat teffects



//WHITES//
  sem (FCOSTEXP -> HDLW4, ) (DASHDIETEXP -> HDLW4, ) ( Agew1_C-> HDLW4, ) (Agew3_C -> HDLW4, ) (sex ->
HDLW4, ) (pir -> HDLW4, ) (edubr2 -> HDLW4, ) (edubr3 -> HDLW4, ) (employed1 -> HDLW4, )    (wrattbr2 -> HDLW4, )
(wrattbr3 -> HDLW4, )(wrattbr4 -> HDLW4, ) (smoke1 -> HDLW4, ) (smoke9 -> HDLW4, )(currdrugs1 -> HDLW4, )
```

```
(currdrugs9 -> HDLW4, ) (bmi_C -> HDLW4, ) (SRHbr2 -> HDLW4, ) (SRHbr3 -> HDLW4, )(kcalw1w3mean_C -> HDLW4, ) (energystoresw1w3mean_C -> HDLW4, ) (invmillssem -> HDLW4, ) ///
(FCOSTEXP -> DASHDIETEXP, )( Agew1_C-> DASHDIETEXP, ) (Agew3_C -> DASHDIETEXP, ) (sex -> DASHDIETEXP, ) (pir -> DASHDIETEXP, ) (edubr2 -> DASHDIETEXP, ) (edubr3 -> DASHDIETEXP, ) (employed1 -> DASHDIETEXP, ) (wrattbr2 -> DASHDIETEXP, ) (wrattbr3 -> DASHDIETEXP, ) (wrattbr4 -> DASHDIETEXP, )(smoke1 -> DASHDIETEXP, ) (smoke9 -> DASHDIETEXP, )(currdrugs1 -> DASHDIETEXP, ) (currdrugs9 -> DASHDIETEXP, ) (bmi_C -> DASHDIETEXP, ) (SRHbr2 -> DASHDIETEXP, ) (SRHbr3 -> DASHDIETEXP, )(kcalw1w3mean_C -> DASHDIETEXP, ) (energystoresw1w3mean_C -> DASHDIETEXP, ) (invmillssem -> DASHDIETEXP, ) if finalsample==1 & race==0 , nocapslatent method(ml)

estat gof, stats(all)
estat teffects



//African-Americans//
 sem (FCOSTEXP -> HDLW4, ) (DASHDIETEXP -> HDLW4, ) ( Agew1_C-> HDLW4, ) (Agew3_C -> HDLW4, ) (sex -> HDLW4, ) (pir -> HDLW4, ) (edubr2 -> HDLW4, ) (edubr3 -> HDLW4, ) (employed1 -> HDLW4, )  (wrattbr2 -> HDLW4, ) (wrattbr3 -> HDLW4, ) (wrattbr4 -> HDLW4, )(smoke1 -> HDLW4, ) (smoke9 -> HDLW4, )(currdrugs1 -> HDLW4, ) (currdrugs9 -> HDLW4, ) (bmi_C -> HDLW4, ) (SRHbr2 -> HDLW4, ) (SRHbr3 -> HDLW4, )(kcalw1w3mean_C -> HDLW4, ) (energystoresw1w3mean_C -> HDLW4, ) (invmillssem -> HDLW4, ) ///
(FCOSTEXP -> DASHDIETEXP, )( Agew1_C-> DASHDIETEXP, ) (Agew3_C -> DASHDIETEXP, ) (sex -> DASHDIETEXP, ) (pir -> DASHDIETEXP, ) (edubr2 -> DASHDIETEXP, ) (edubr3 -> DASHDIETEXP, ) (employed1 -> DASHDIETEXP, ) (wrattbr2 -> DASHDIETEXP, ) (wrattbr3 -> DASHDIETEXP, ) (wrattbr4 -> DASHDIETEXP, )(smoke1 -> DASHDIETEXP, ) (smoke9 -> DASHDIETEXP, )(currdrugs1 -> DASHDIETEXP, ) (currdrugs9 -> DASHDIETEXP, ) (bmi_C -> DASHDIETEXP, ) (SRHbr2 -> DASHDIETEXP, ) (SRHbr3 -> DASHDIETEXP, )(kcalw1w3mean_C -> DASHDIETEXP, ) (energystoresw1w3mean_C -> DASHDIETEXP, ) (invmillssem -> DASHDIETEXP, ) if finalsample==1 & race==1 , nocapslatent method(ml)

estat gof, stats(all)
estat teffects



//BELOW POVERTY//
sem (FCOSTEXP -> HDLW4, ) (DASHDIETEXP -> HDLW4, ) ( Agew1_C-> HDLW4, ) (Agew3_C -> HDLW4, ) (sex -> HDLW4, ) (race -> HDLW4, ) (edubr2 -> HDLW4, ) (edubr3 -> HDLW4, ) (employed1 -> HDLW4, )  (wrattbr2 -> HDLW4, ) (wrattbr3 -> HDLW4, ) (wrattbr4 -> HDLW4, )(smoke1 -> HDLW4, ) (smoke9 -> HDLW4, )(currdrugs1 -> HDLW4, ) (currdrugs9 -> HDLW4, ) (bmi_C -> HDLW4, ) (SRHbr2 -> HDLW4, ) (SRHbr3 -> HDLW4, )(kcalw1w3mean_C -> HDLW4, ) (energystoresw1w3mean_C -> HDLW4, ) (invmillssem -> HDLW4, ) ///
(FCOSTEXP -> DASHDIETEXP, )( Agew1_C-> DASHDIETEXP, ) (Agew3_C -> DASHDIETEXP, ) (sex -> DASHDIETEXP, ) (race -> DASHDIETEXP, ) (edubr2 -> DASHDIETEXP, ) (edubr3 -> DASHDIETEXP, ) (employed1 -> DASHDIETEXP, ) (wrattbr2 -> DASHDIETEXP, ) (wrattbr3 -> DASHDIETEXP, ) (wrattbr4 -> DASHDIETEXP, )(smoke1 -> DASHDIETEXP, ) (smoke9 -> DASHDIETEXP, )(currdrugs1 -> DASHDIETEXP, ) (currdrugs9 -> DASHDIETEXP, ) (bmi_C -> DASHDIETEXP, ) (SRHbr2 -> DASHDIETEXP, ) (SRHbr3 -> DASHDIETEXP, )(kcalw1w3mean_C -> DASHDIETEXP, )
```

(energystoresw1w3mean_C -> DASHDIETEXP,) (invmillssem -> DASHDIETEXP,) if finalsample==1 & pir==0 , nocapslatent method(ml)

estat gof, stats(all)
estat teffects

//ABOVE POVERTY//
sem (FCOSTEXP -> HDLW4,) (DASHDIETEXP -> HDLW4,) (Agew1_C-> HDLW4,) (Agew3_C -> HDLW4,) (sex -> HDLW4,) (race -> HDLW4,) (edubr2 -> HDLW4,) (edubr3 -> HDLW4,) (employed1 -> HDLW4,) (wrattbr2 -> HDLW4,) (wrattbr3 -> HDLW4,) (wrattbr4 -> HDLW4,)(smoke1 -> HDLW4,) (smoke9 -> HDLW4,)(currdrugs1 -> HDLW4,) (currdrugs9 -> HDLW4,) (bmi_C -> HDLW4,) (SRHbr2 -> HDLW4,) (SRHbr3 -> HDLW4,)(kcalw1w3mean_C -> HDLW4,) (energystoresw1w3mean_C -> HDLW4,) (invmillssem -> HDLW4,) ///
(FCOSTEXP -> DASHDIETEXP,)(Agew1_C-> DASHDIETEXP,) (Agew3_C -> DASHDIETEXP,) (sex -> DASHDIETEXP,) (race -> DASHDIETEXP,) (edubr2 -> DASHDIETEXP,) (edubr3 -> DASHDIETEXP,) (employed1 -> DASHDIETEXP,) (wrattbr2 -> DASHDIETEXP,) (wrattbr3 -> DASHDIETEXP,) (wrattbr4 -> DASHDIETEXP,)(smoke1 -> DASHDIETEXP,) (smoke9 -> DASHDIETEXP,)(currdrugs1 -> DASHDIETEXP,) (currdrugs9 -> DASHDIETEXP,) (bmi_C -> DASHDIETEXP,) (SRHbr2 -> DASHDIETEXP,) (SRHbr3 -> DASHDIETEXP,)(kcalw1w3mean_C -> DASHDIETEXP,) (energystoresw1w3mean_C -> DASHDIETEXP,) (invmillssem -> DASHDIETEXP,) if finalsample==1 & pir==1 , nocapslatent method(ml)

estat gof, stats(all)
estat teffects

****************************HEMOGLOBIN A 1C WAVE 4***
//MULTI-GROUP//

All
sem (FCOSTEXP -> HGBA1C ,) (DASHDIETEXP -> HGBA1C ,) (Agew1_C-> HGBA1C ,) (Agew3_C -> HGBA1C ,) (sex -> HGBA1C ,) (race -> HGBA1C ,) (pir -> HGBA1C ,) (edubr2 -> HGBA1C ,) (edubr3 -> HGBA1C ,) (employed1 -> HGBA1C ,) (wrattbr2 -> HGBA1C ,) (wrattbr3 -> HGBA1C ,) (wrattbr4 -> HGBA1C ,)(smoke1 -> HGBA1C ,) (smoke9 -> HGBA1C ,)(currdrugs1 -> HGBA1C ,) (currdrugs9 -> HGBA1C ,) (bmi_C -> HGBA1C ,) (SRHbr2 -> HGBA1C ,) (SRHbr3 -> HGBA1C ,)(kcalw1w3mean_C -> HGBA1C ,) (energystoresw1w3mean_C -> HGBA1C ,) (invmillssem -> HGBA1C ,) ///
(FCOSTEXP -> DASHDIETEXP,)(Agew1_C-> DASHDIETEXP,) (Agew3_C -> DASHDIETEXP,) (sex -> DASHDIETEXP,) (race -> DASHDIETEXP,) (pir -> DASHDIETEXP,) (edubr2 -> DASHDIETEXP,) (edubr3 -> DASHDIETEXP,) (employed1 -> DASHDIETEXP,) (wrattbr2 -> DASHDIETEXP,) (wrattbr3 -> DASHDIETEXP,) (wrattbr4 -> DASHDIETEXP,)(smoke1 -> DASHDIETEXP,) (smoke9 -> DASHDIETEXP,)(currdrugs1 -> DASHDIETEXP,) (currdrugs9 -> DASHDIETEXP,) (bmi_C -> DASHDIETEXP,) (SRHbr2 -> DASHDIETEXP,) (SRHbr3 -> DASHDIETEXP,)(kcalw1w3mean_C -> DASHDIETEXP,) (energystoresw1w3mean_C -> DASHDIETEXP,) (invmillssem -> DASHDIETEXP,) , nocapslatent method(ml) group(finalsample)

estat ginvariant

```
estat gof, stats(all)
estat teffects
```

By sex

```
sem (FCOSTEXP -> HGBA1C , ) (DASHDIETEXP -> HGBA1C , ) ( Agew1_C-> HGBA1C , ) (Agew3_C -> HGBA1C , ) (race -> HGBA1C , ) (pir -> HGBA1C , ) (edubr2 -> HGBA1C , ) (edubr3 -> HGBA1C , ) (employed1 -> HGBA1C , )  (wrattbr2 -> HGBA1C , ) (wrattbr3 -> HGBA1C , ) (wrattbr4 -> HGBA1C , )(smoke1 -> HGBA1C , ) (smoke9 -> HGBA1C , )(currdrugs1 -> HGBA1C , ) (currdrugs9 -> HGBA1C , ) (bmi_C -> HGBA1C , ) (SRHbr2 -> HGBA1C , ) (SRHbr3 -> HGBA1C , )(kcalw1w3mean_C -> HGBA1C , ) (energystoresw1w3mean_C -> HGBA1C , ) (invmillssem -> HGBA1C , ) ///
(FCOSTEXP -> DASHDIETEXP, )( Agew1_C-> DASHDIETEXP, ) (Agew3_C -> DASHDIETEXP, ) (race -> DASHDIETEXP, ) (pir -> DASHDIETEXP, ) (edubr2 -> DASHDIETEXP, ) (edubr3 -> DASHDIETEXP, ) (employed1 -> DASHDIETEXP, ) (wrattbr2 -> DASHDIETEXP, ) (wrattbr3 -> DASHDIETEXP, )(wrattbr4 -> DASHDIETEXP, ) (smoke1 -> DASHDIETEXP, ) (smoke9 -> DASHDIETEXP, )(currdrugs1 -> DASHDIETEXP, ) (currdrugs9 -> DASHDIETEXP, ) (bmi_C -> DASHDIETEXP, ) (SRHbr2 -> DASHDIETEXP, ) (SRHbr3 -> DASHDIETEXP, )(kcalw1w3mean_C -> DASHDIETEXP, ) (energystoresw1w3mean_C -> DASHDIETEXP, ) (invmillssem -> DASHDIETEXP, ) if finalsample==1 , nocapslatent method(ml) group(sex)

estat ginvariant
```

By race

```
sem (FCOSTEXP -> HGBA1C , ) (DASHDIETEXP -> HGBA1C , ) ( Agew1_C-> HGBA1C , ) (Agew3_C -> HGBA1C , ) (sex -> HGBA1C , ) (pir -> HGBA1C , ) (edubr2 -> HGBA1C , ) (edubr3 -> HGBA1C , ) (employed1 -> HGBA1C , )  (wrattbr2 -> HGBA1C , ) (wrattbr3 -> HGBA1C , ) (wrattbr4 -> HGBA1C , )(smoke1 -> HGBA1C , ) (smoke9 -> HGBA1C , )(currdrugs1 -> HGBA1C , ) (currdrugs9 -> HGBA1C , ) (bmi_C -> HGBA1C , ) (SRHbr2 -> HGBA1C , ) (SRHbr3 -> HGBA1C , )(kcalw1w3mean_C -> HGBA1C , ) (energystoresw1w3mean_C -> HGBA1C , ) (invmillssem -> HGBA1C , ) ///
(FCOSTEXP -> DASHDIETEXP, )( Agew1_C-> DASHDIETEXP, ) (Agew3_C -> DASHDIETEXP, ) (sex -> DASHDIETEXP, ) (pir -> DASHDIETEXP, ) (edubr2 -> DASHDIETEXP, ) (edubr3 -> DASHDIETEXP, ) (employed1 -> DASHDIETEXP, ) (wrattbr2 -> DASHDIETEXP, ) (wrattbr3 -> DASHDIETEXP, ) (wrattbr4 -> DASHDIETEXP, )(smoke1 -> DASHDIETEXP, ) (smoke9 -> DASHDIETEXP, )(currdrugs1 -> DASHDIETEXP, ) (currdrugs9 -> DASHDIETEXP, ) (bmi_C -> DASHDIETEXP, ) (SRHbr2 -> DASHDIETEXP, ) (SRHbr3 -> DASHDIETEXP, )(kcalw1w3mean_C -> DASHDIETEXP, ) (energystoresw1w3mean_C -> DASHDIETEXP, ) (invmillssem -> DASHDIETEXP, ) if finalsample==1 , nocapslatent method(ml) group(race)

estat ginvariant
```

By pir

```
sem (FCOSTEXP -> HGBA1C , ) (DASHDIETEXP -> HGBA1C , ) ( Agew1_C-> HGBA1C , ) (Agew3_C -> HGBA1C , ) (sex -> HGBA1C , ) (race -> HGBA1C , ) (edubr2 -> HGBA1C , ) (edubr3 -> HGBA1C , ) (employed1 -> HGBA1C , )   (wrattbr2 -> HGBA1C , ) (wrattbr3 -> HGBA1C , ) (wrattbr4 -> HGBA1C , )(smoke1 -> HGBA1C , ) (smoke9 -> HGBA1C , )(currdrugs1 -> HGBA1C , ) (currdrugs9 -> HGBA1C , ) (bmi_C -> HGBA1C , ) (SRHbr2 -> HGBA1C , ) (SRHbr3 -> HGBA1C , )(kcalw1w3mean_C -> HGBA1C , ) (energystoresw1w3mean_C -> HGBA1C , ) (invmillssem -> HGBA1C , ) ///
(FCOSTEXP -> DASHDIETEXP, )( Agew1_C-> DASHDIETEXP, ) (Agew3_C -> DASHDIETEXP, ) (sex -> DASHDIETEXP, ) (race -> DASHDIETEXP, )   (edubr2 -> DASHDIETEXP, ) (edubr3 -> DASHDIETEXP, ) (employed1 -> DASHDIETEXP, ) (wrattbr2 -> DASHDIETEXP, ) (wrattbr3 -> DASHDIETEXP, ) (wrattbr4 -> DASHDIETEXP, )(smoke1 -> DASHDIETEXP, ) (smoke9 -> DASHDIETEXP, )(currdrugs1 -> DASHDIETEXP, ) (currdrugs9 -> DASHDIETEXP, ) (bmi_C -> DASHDIETEXP, ) (SRHbr2 -> DASHDIETEXP, ) (SRHbr3 -> DASHDIETEXP, )(kcalw1w3mean_C -> DASHDIETEXP, ) (energystoresw1w3mean_C -> DASHDIETEXP, ) (invmillssem -> DASHDIETEXP, ) if finalsample==1 , nocapslatent method(ml) group(pir)

estat ginvariant



*********************STRATIFIED ANALYSIS********************************

//MEN//
sem (FCOSTEXP -> HGBA1C , ) (DASHDIETEXP -> HGBA1C , ) ( Agew1_C-> HGBA1C , ) (Agew3_C -> HGBA1C , ) (race -> HGBA1C , ) (pir -> HGBA1C , ) (edubr2 -> HGBA1C , ) (edubr3 -> HGBA1C , ) (employed1 -> HGBA1C , )   (wrattbr2 -> HGBA1C , ) (wrattbr3 -> HGBA1C , ) (wrattbr4 -> HGBA1C , )(smoke1 -> HGBA1C , ) (smoke9 -> HGBA1C , )(currdrugs1 -> HGBA1C , ) (currdrugs9 -> HGBA1C , ) (bmi_C -> HGBA1C , ) (SRHbr2 -> HGBA1C , ) (SRHbr3 -> HGBA1C , )(kcalw1w3mean_C -> HGBA1C , ) (energystoresw1w3mean_C -> HGBA1C , ) (invmillssem -> HGBA1C , ) ///
(FCOSTEXP -> DASHDIETEXP, )( Agew1_C-> DASHDIETEXP, ) (Agew3_C -> DASHDIETEXP, ) (race -> DASHDIETEXP, ) (pir -> DASHDIETEXP, ) (edubr2 -> DASHDIETEXP, ) (edubr3 -> DASHDIETEXP, ) (employed1 -> DASHDIETEXP, ) (wrattbr2 -> DASHDIETEXP, ) (wrattbr3 -> DASHDIETEXP, )(wrattbr4 -> DASHDIETEXP, ) (smoke1 -> DASHDIETEXP, ) (smoke9 -> DASHDIETEXP, )(currdrugs1 -> DASHDIETEXP, ) (currdrugs9 -> DASHDIETEXP, ) (bmi_C -> DASHDIETEXP, ) (SRHbr2 -> DASHDIETEXP, ) (SRHbr3 -> DASHDIETEXP, )(kcalw1w3mean_C -> DASHDIETEXP, ) (energystoresw1w3mean_C -> DASHDIETEXP, ) (invmillssem -> DASHDIETEXP, ) if finalsample==1 & sex==1 , nocapslatent method(ml)

estat gof, stats(all)
estat teffects



//WOMEN//
sem (FCOSTEXP -> HGBA1C , ) (DASHDIETEXP -> HGBA1C , ) ( Agew1_C-> HGBA1C , ) (Agew3_C -> HGBA1C , ) (race -> HGBA1C , ) (pir -> HGBA1C , ) (edubr2 -> HGBA1C , ) (edubr3 -> HGBA1C , ) (employed1 -> HGBA1C , )   (wrattbr2 -> HGBA1C , ) (wrattbr3 -> HGBA1C , ) (wrattbr4 -> HGBA1C , )(smoke1 -> HGBA1C , ) (smoke9 -> HGBA1C , )(currdrugs1 -> HGBA1C , ) (currdrugs9 -> HGBA1C , ) (bmi_C -> HGBA1C , ) (SRHbr2 -> HGBA1C , ) (SRHbr3 -> HGBA1C , )(kcalw1w3mean_C -> HGBA1C , ) (energystoresw1w3mean_C -> HGBA1C , ) (invmillssem -> HGBA1C , ) ///
(FCOSTEXP -> DASHDIETEXP, )( Agew1_C-> DASHDIETEXP, ) (Agew3_C -> DASHDIETEXP, ) (race -> DASHDIETEXP, ) (pir -> DASHDIETEXP, ) (edubr2 -> DASHDIETEXP, ) (edubr3 -> DASHDIETEXP, ) (employed1 -> DASHDIETEXP, )
```

(wrattbr2 -> DASHDIETEXP,) (wrattbr3 -> DASHDIETEXP,) (wrattbr4 -> DASHDIETEXP,)(smoke1 -> DASHDIETEXP,) (smoke9 -> DASHDIETEXP,)(currdrugs1 -> DASHDIETEXP,) (currdrugs9 -> DASHDIETEXP,) (bmi_C -> DASHDIETEXP,) (SRHbr2 -> DASHDIETEXP,) (SRHbr3 -> DASHDIETEXP,)(kcalw1w3mean_C -> DASHDIETEXP,) (energystoresw1w3mean_C -> DASHDIETEXP,) (invmillssem -> DASHDIETEXP,) if finalsample==1 & sex==0 , nocapslatent method(ml)

estat gof, stats(all)
estat teffects

//WHITES//
sem (FCOSTEXP -> HGBA1C ,) (DASHDIETEXP -> HGBA1C ,) (Agew1_C-> HGBA1C ,) (Agew3_C -> HGBA1C ,) (sex -> HGBA1C ,) (pir -> HGBA1C ,) (edubr2 -> HGBA1C ,) (edubr3 -> HGBA1C ,) (employed1 -> HGBA1C ,) (wrattbr2 -> HGBA1C ,) (wrattbr3 -> HGBA1C ,)(wrattbr4 -> HGBA1C ,) (smoke1 -> HGBA1C ,) (smoke9 -> HGBA1C ,)(currdrugs1 -> HGBA1C ,) (currdrugs9 -> HGBA1C ,) (bmi_C -> HGBA1C ,) (SRHbr2 -> HGBA1C ,) (SRHbr3 -> HGBA1C ,)(kcalw1w3mean_C -> HGBA1C ,) (energystoresw1w3mean_C -> HGBA1C ,) (invmillssem -> HGBA1C ,) ///
(FCOSTEXP -> DASHDIETEXP,)(Agew1_C-> DASHDIETEXP,) (Agew3_C -> DASHDIETEXP,) (sex -> DASHDIETEXP,) (pir -> DASHDIETEXP,) (edubr2 -> DASHDIETEXP,) (edubr3 -> DASHDIETEXP,) (employed1 -> DASHDIETEXP,) (wrattbr2 -> DASHDIETEXP,) (wrattbr3 -> DASHDIETEXP,) (wrattbr4 -> DASHDIETEXP,)(smoke1 -> DASHDIETEXP,) (smoke9 -> DASHDIETEXP,)(currdrugs1 -> DASHDIETEXP,) (currdrugs9 -> DASHDIETEXP,) (bmi_C -> DASHDIETEXP,) (SRHbr2 -> DASHDIETEXP,) (SRHbr3 -> DASHDIETEXP,)(kcalw1w3mean_C -> DASHDIETEXP,) (energystoresw1w3mean_C -> DASHDIETEXP,) (invmillssem -> DASHDIETEXP,) if finalsample==1 & race==0 , nocapslatent method(ml)

estat gof, stats(all)
estat teffects

//African-Americans//
sem (FCOSTEXP -> HGBA1C ,) (DASHDIETEXP -> HGBA1C ,) (Agew1_C-> HGBA1C ,) (Agew3_C -> HGBA1C ,) (sex -> HGBA1C ,) (pir -> HGBA1C ,) (edubr2 -> HGBA1C ,) (edubr3 -> HGBA1C ,) (employed1 -> HGBA1C ,) (wrattbr2 -> HGBA1C ,) (wrattbr3 -> HGBA1C ,) (wrattbr4 -> HGBA1C ,)(smoke1 -> HGBA1C ,) (smoke9 -> HGBA1C ,)(currdrugs1 -> HGBA1C ,) (currdrugs9 -> HGBA1C ,) (bmi_C -> HGBA1C ,) (SRHbr2 -> HGBA1C ,) (SRHbr3 -> HGBA1C ,)(kcalw1w3mean_C -> HGBA1C ,) (energystoresw1w3mean_C -> HGBA1C ,) (invmillssem -> HGBA1C ,) ///
(FCOSTEXP -> DASHDIETEXP,)(Agew1_C-> DASHDIETEXP,) (Agew3_C -> DASHDIETEXP,) (sex -> DASHDIETEXP,) (pir -> DASHDIETEXP,) (edubr2 -> DASHDIETEXP,) (edubr3 -> DASHDIETEXP,) (employed1 -> DASHDIETEXP,) (wrattbr2 -> DASHDIETEXP,) (wrattbr3 -> DASHDIETEXP,) (wrattbr4 -> DASHDIETEXP,)(smoke1 -> DASHDIETEXP,) (smoke9 -> DASHDIETEXP,)(currdrugs1 -> DASHDIETEXP,) (currdrugs9 -> DASHDIETEXP,) (bmi_C -> DASHDIETEXP,) (SRHbr2 -> DASHDIETEXP,) (SRHbr3 -> DASHDIETEXP,)(kcalw1w3mean_C -> DASHDIETEXP,) (energystoresw1w3mean_C -> DASHDIETEXP,) (invmillssem -> DASHDIETEXP,) if finalsample==1 & race==1 , nocapslatent method(ml)

estat gof, stats(all)
estat teffects

//BELOW POVERTY//

sem (FCOSTEXP -> HGBA1C ,) (DASHDIETEXP -> HGBA1C ,) (Agew1_C-> HGBA1C ,) (Agew3_C -> HGBA1C ,) (sex -> HGBA1C ,) (race -> HGBA1C ,) (edubr2 -> HGBA1C ,) (edubr3 -> HGBA1C ,) (employed1 -> HGBA1C ,) (wrattbr2 -> HGBA1C ,) (wrattbr3 -> HGBA1C ,) (wrattbr4 -> HGBA1C ,)(smoke1 -> HGBA1C ,) (smoke9 -> HGBA1C ,)(currdrugs1 -> HGBA1C ,) (currdrugs9 -> HGBA1C ,) (bmi_C -> HGBA1C ,) (SRHbr2 -> HGBA1C ,) (SRHbr3 -> HGBA1C ,)(kcalw1w3mean_C -> HGBA1C ,) (energystoresw1w3mean_C -> HGBA1C ,) (invmillssem -> HGBA1C ,) /// (FCOSTEXP -> DASHDIETEXP,)(Agew1_C-> DASHDIETEXP,) (Agew3_C -> DASHDIETEXP,) (sex -> DASHDIETEXP,) (race -> DASHDIETEXP,) (edubr2 -> DASHDIETEXP,) (edubr3 -> DASHDIETEXP,) (employed1 -> DASHDIETEXP,) (wrattbr2 -> DASHDIETEXP,) (wrattbr3 -> DASHDIETEXP,) (wrattbr4 -> DASHDIETEXP,)(smoke1 -> DASHDIETEXP,) (smoke9 -> DASHDIETEXP,)(currdrugs1 -> DASHDIETEXP,) (currdrugs9 -> DASHDIETEXP,) (bmi_C -> DASHDIETEXP,) (SRHbr2 -> DASHDIETEXP,) (SRHbr3 -> DASHDIETEXP,)(kcalw1w3mean_C -> DASHDIETEXP,) (energystoresw1w3mean_C -> DASHDIETEXP,) (invmillssem -> DASHDIETEXP,) if finalsample==1 & pir==0 , nocapslatent method(ml)

estat gof, stats(all)
estat teffects

//ABOVE POVERTY//

sem (FCOSTEXP -> HGBA1C ,) (DASHDIETEXP -> HGBA1C ,) (Agew1_C-> HGBA1C ,) (Agew3_C -> HGBA1C ,) (sex -> HGBA1C ,) (race -> HGBA1C ,) (edubr2 -> HGBA1C ,) (edubr3 -> HGBA1C ,) (employed1 -> HGBA1C ,) (wrattbr2 -> HGBA1C ,) (wrattbr3 -> HGBA1C ,) (wrattbr4 -> HGBA1C ,)(smoke1 -> HGBA1C ,) (smoke9 -> HGBA1C ,)(currdrugs1 -> HGBA1C ,) (currdrugs9 -> HGBA1C ,) (bmi_C -> HGBA1C ,) (SRHbr2 -> HGBA1C ,) (SRHbr3 -> HGBA1C ,)(kcalw1w3mean_C -> HGBA1C ,) (energystoresw1w3mean_C -> HGBA1C ,) (invmillssem -> HGBA1C ,) /// (FCOSTEXP -> DASHDIETEXP,)(Agew1_C-> DASHDIETEXP,) (Agew3_C -> DASHDIETEXP,) (sex -> DASHDIETEXP,) (race -> DASHDIETEXP,) (edubr2 -> DASHDIETEXP,) (edubr3 -> DASHDIETEXP,) (employed1 -> DASHDIETEXP,) (wrattbr2 -> DASHDIETEXP,) (wrattbr3 -> DASHDIETEXP,) (wrattbr4 -> DASHDIETEXP,)(smoke1 -> DASHDIETEXP,) (smoke9 -> DASHDIETEXP,)(currdrugs1 -> DASHDIETEXP,) (currdrugs9 -> DASHDIETEXP,) (bmi_C -> DASHDIETEXP,) (SRHbr2 -> DASHDIETEXP,) (SRHbr3 -> DASHDIETEXP,)(kcalw1w3mean_C -> DASHDIETEXP,) (energystoresw1w3mean_C -> DASHDIETEXP,) (invmillssem -> DASHDIETEXP,) if finalsample==1 & pir==1 , nocapslatent method(ml)

estat gof, stats(all)
estat teffects

****************************WAIST-HIP-RATIO WAVE 4**

//MULTI-GROUP//

All

sem (FCOSTEXP -> WHRW4,) (DASHDIETEXP -> WHRW4,) (Agew1_C-> WHRW4,) (Agew3_C -> WHRW4,) (sex -> WHRW4,) (race -> WHRW4,) (pir -> WHRW4,) (edubr2 -> WHRW4,) (edubr3 -> WHRW4,) (employed1 -> WHRW4,)

(wrattbr2 -> WHRW4,) (wrattbr3 -> WHRW4,) (wrattbr4 -> WHRW4,)(smoke1 -> WHRW4,) (smoke9 -> WHRW4,)(currdrugs1 -> WHRW4,) (currdrugs9 -> WHRW4,) (bmi_C -> WHRW4,) (SRHbr2 -> WHRW4,) (SRHbr3 -> WHRW4,)(kcalw1w3mean_C -> WHRW4,) (energystoresw1w3mean_C -> WHRW4,) (invmillssem -> WHRW4,) ///
(FCOSTEXP -> DASHDIETEXP,)(Agew1_C-> DASHDIETEXP,) (Agew3_C -> DASHDIETEXP,) (sex -> DASHDIETEXP,) (race -> DASHDIETEXP,) (pir -> DASHDIETEXP,) (edubr2 -> DASHDIETEXP,) (edubr3 -> DASHDIETEXP,) (employed1 -> DASHDIETEXP,) (wrattbr2 -> DASHDIETEXP,) (wrattbr3 -> DASHDIETEXP,) (wrattbr4 -> DASHDIETEXP,)(smoke1 -> DASHDIETEXP,) (smoke9 -> DASHDIETEXP,)(currdrugs1 -> DASHDIETEXP,) (currdrugs9 -> DASHDIETEXP,) (bmi_C -> DASHDIETEXP,) (SRHbr2 -> DASHDIETEXP,) (SRHbr3 -> DASHDIETEXP,)(kcalw1w3mean_C -> DASHDIETEXP,) (energystoresw1w3mean_C -> DASHDIETEXP,) (invmillssem -> DASHDIETEXP,) , nocapslatent method(ml) group(finalsample)

estat ginvariant

estat gof, stats(all)
estat teffects

By sex

sem (FCOSTEXP -> WHRW4,) (DASHDIETEXP -> WHRW4,) (Agew1_C-> WHRW4,) (Agew3_C -> WHRW4,) (race -> WHRW4,) (pir -> WHRW4,) (edubr2 -> WHRW4,) (edubr3 -> WHRW4,) (employed1 -> WHRW4,) (wrattbr2 -> WHRW4,) (wrattbr3 -> WHRW4,) (wrattbr4 -> WHRW4,)(smoke1 -> WHRW4,) (smoke9 -> WHRW4,)(currdrugs1 -> WHRW4,) (currdrugs9 -> WHRW4,) (bmi_C -> WHRW4,) (SRHbr2 -> WHRW4,) (SRHbr3 -> WHRW4,)(kcalw1w3mean_C -> WHRW4,) (energystoresw1w3mean_C -> WHRW4,) (invmillssem -> WHRW4,) ///
(FCOSTEXP -> DASHDIETEXP,)(Agew1_C-> DASHDIETEXP,) (Agew3_C -> DASHDIETEXP,) (race -> DASHDIETEXP,) (pir -> DASHDIETEXP,) (edubr2 -> DASHDIETEXP,) (edubr3 -> DASHDIETEXP,) (employed1 -> DASHDIETEXP,) (wrattbr2 -> DASHDIETEXP,) (wrattbr3 -> DASHDIETEXP,)(wrattbr4 -> DASHDIETEXP,) (smoke1 -> DASHDIETEXP,) (smoke9 -> DASHDIETEXP,)(currdrugs1 -> DASHDIETEXP,) (currdrugs9 -> DASHDIETEXP,) (bmi_C -> DASHDIETEXP,) (SRHbr2 -> DASHDIETEXP,) (SRHbr3 -> DASHDIETEXP,)(kcalw1w3mean_C -> DASHDIETEXP,) (energystoresw1w3mean_C -> DASHDIETEXP,) (invmillssem -> DASHDIETEXP,) if finalsample==1 , nocapslatent method(ml) group(sex)

estat ginvariant

By race

sem (FCOSTEXP -> WHRW4,) (DASHDIETEXP -> WHRW4,) (Agew1_C-> WHRW4,) (Agew3_C -> WHRW4,) (sex -> WHRW4,) (pir -> WHRW4,) (edubr2 -> WHRW4,) (edubr3 -> WHRW4,) (employed1 -> WHRW4,) (wrattbr2 -> WHRW4,) (wrattbr3 -> WHRW4,) (wrattbr4 -> WHRW4,)(smoke1 -> WHRW4,) (smoke9 -> WHRW4,)(currdrugs1 -> WHRW4,) (currdrugs9 -> WHRW4,) (bmi_C -> WHRW4,) (SRHbr2 -> WHRW4,) (SRHbr3 -> WHRW4,)(kcalw1w3mean_C -> WHRW4,) (energystoresw1w3mean_C -> WHRW4,) (invmillssem -> WHRW4,) ///

(FCOSTEXP -> DASHDIETEXP,)(Agew1_C-> DASHDIETEXP,) (Agew3_C -> DASHDIETEXP,) (sex -> DASHDIETEXP,) (pir -> DASHDIETEXP,) (edubr2 -> DASHDIETEXP,) (edubr3 -> DASHDIETEXP,) (employed1 -> DASHDIETEXP,) (wrattbr2 -> DASHDIETEXP,) (wrattbr3 -> DASHDIETEXP,) (wrattbr4 -> DASHDIETEXP,)(smoke1 -> DASHDIETEXP,) (smoke9 -> DASHDIETEXP,)(currdrugs1 -> DASHDIETEXP,) (currdrugs9 -> DASHDIETEXP,) (bmi_C -> DASHDIETEXP,) (SRHbr2 -> DASHDIETEXP,) (SRHbr3 -> DASHDIETEXP,)(kcalw1w3mean_C -> DASHDIETEXP,) (energystoresw1w3mean_C -> DASHDIETEXP,) (invmillssem -> DASHDIETEXP,) if finalsample==1 , nocapslatent method(ml) group(race)

estat ginvariant

By pir

sem (FCOSTEXP -> WHRW4,) (DASHDIETEXP -> WHRW4,) (Agew1_C-> WHRW4,) (Agew3_C -> WHRW4,) (sex -> WHRW4,) (race -> WHRW4,) (edubr2 -> WHRW4,) (edubr3 -> WHRW4,) (employed1 -> WHRW4,) (wrattbr2 -> WHRW4,) (wrattbr3 -> WHRW4,) (wrattbr4 -> WHRW4,)(smoke1 -> WHRW4,) (smoke9 -> WHRW4,)(currdrugs1 -> WHRW4,) (currdrugs9 -> WHRW4,) (bmi_C -> WHRW4,) (SRHbr2 -> WHRW4,) (SRHbr3 -> WHRW4,)(kcalw1w3mean_C -> WHRW4,) (energystoresw1w3mean_C -> WHRW4,) (invmillssem -> WHRW4,) ///
(FCOSTEXP -> DASHDIETEXP,)(Agew1_C-> DASHDIETEXP,) (Agew3_C -> DASHDIETEXP,) (sex -> DASHDIETEXP,) (race -> DASHDIETEXP,) (edubr2 -> DASHDIETEXP,) (edubr3 -> DASHDIETEXP,) (employed1 -> DASHDIETEXP,) (wrattbr2 -> DASHDIETEXP,) (wrattbr3 -> DASHDIETEXP,) (wrattbr4 -> DASHDIETEXP,)(smoke1 -> DASHDIETEXP,) (smoke9 -> DASHDIETEXP,)(currdrugs1 -> DASHDIETEXP,) (currdrugs9 -> DASHDIETEXP,) (bmi_C -> DASHDIETEXP,) (SRHbr2 -> DASHDIETEXP,) (SRHbr3 -> DASHDIETEXP,)(kcalw1w3mean_C -> DASHDIETEXP,) (energystoresw1w3mean_C -> DASHDIETEXP,) (invmillssem -> DASHDIETEXP,) if finalsample==1 , nocapslatent method(ml) group(pir)

estat ginvariant

********************STRATIFIED ANALYSIS*******************************

//MEN//
sem (FCOSTEXP -> WHRW4,) (DASHDIETEXP -> WHRW4,) (Agew1_C-> WHRW4,) (Agew3_C -> WHRW4,) (race -> WHRW4,) (pir -> WHRW4,) (edubr2 -> WHRW4,) (edubr3 -> WHRW4,) (employed1 -> WHRW4,) (wrattbr2 -> WHRW4,) (wrattbr3 -> WHRW4,) (wrattbr4 -> WHRW4,)(smoke1 -> WHRW4,) (smoke9 -> WHRW4,)(currdrugs1 -> WHRW4,) (currdrugs9 -> WHRW4,) (bmi_C -> WHRW4,) (SRHbr2 -> WHRW4,) (SRHbr3 -> WHRW4,)(kcalw1w3mean_C -> WHRW4,) (energystoresw1w3mean_C -> WHRW4,) (invmillssem -> WHRW4,) ///
(FCOSTEXP -> DASHDIETEXP,)(Agew1_C-> DASHDIETEXP,) (Agew3_C -> DASHDIETEXP,) (race -> DASHDIETEXP,) (pir -> DASHDIETEXP,) (edubr2 -> DASHDIETEXP,) (edubr3 -> DASHDIETEXP,) (employed1 -> DASHDIETEXP,) (wrattbr2 -> DASHDIETEXP,) (wrattbr3 -> DASHDIETEXP,)(wrattbr4 -> DASHDIETEXP,) (smoke1 -> DASHDIETEXP,) (smoke9 -> DASHDIETEXP,)(currdrugs1 -> DASHDIETEXP,) (currdrugs9 -> DASHDIETEXP,) (bmi_C -> DASHDIETEXP,) (SRHbr2 -> DASHDIETEXP,) (SRHbr3 -> DASHDIETEXP,)(kcalw1w3mean_C -> DASHDIETEXP,) (energystoresw1w3mean_C -> DASHDIETEXP,) (invmillssem -> DASHDIETEXP,) if finalsample==1 & sex==1 , nocapslatent method(ml)

```
estat gof, stats(all)
estat teffects



//WOMEN//
sem (FCOSTEXP -> WHRW4, ) (DASHDIETEXP -> WHRW4, ) ( Agew1_C-> WHRW4, ) (Agew3_C -> WHRW4, ) (race -> WHRW4, ) (pir -> WHRW4, ) (edubr2 -> WHRW4, ) (edubr3 -> WHRW4, ) (employed1 -> WHRW4, )  (wrattbr2 -> WHRW4, ) (wrattbr3 -> WHRW4, ) (wrattbr4 -> WHRW4, )(smoke1 -> WHRW4, ) (smoke9 -> WHRW4, )(currdrugs1 -> WHRW4, ) (currdrugs9 -> WHRW4, ) (bmi_C -> WHRW4, ) (SRHbr2 -> WHRW4, ) (SRHbr3 -> WHRW4, )(kcalw1w3mean_C -> WHRW4, ) (energystoresw1w3mean_C -> WHRW4, ) (invmillssem -> WHRW4, ) ///
(FCOSTEXP -> DASHDIETEXP, )( Agew1_C-> DASHDIETEXP, ) (Agew3_C -> DASHDIETEXP, ) (race -> DASHDIETEXP, ) (pir -> DASHDIETEXP, ) (edubr2 -> DASHDIETEXP, ) (edubr3 -> DASHDIETEXP, ) (employed1 -> DASHDIETEXP, ) (wrattbr2 -> DASHDIETEXP, ) (wrattbr3 -> DASHDIETEXP, ) (wrattbr4 -> DASHDIETEXP, )(smoke1 -> DASHDIETEXP, ) (smoke9 -> DASHDIETEXP, )(currdrugs1 -> DASHDIETEXP, ) (currdrugs9 -> DASHDIETEXP, ) (bmi_C -> DASHDIETEXP, ) (SRHbr2 -> DASHDIETEXP, ) (SRHbr3 -> DASHDIETEXP, )(kcalw1w3mean_C -> DASHDIETEXP, ) (energystoresw1w3mean_C -> DASHDIETEXP, ) (invmillssem -> DASHDIETEXP, ) if finalsample==1 & sex==0 , nocapslatent method(ml)

estat gof, stats(all)
estat teffects



//WHITES//
 sem (FCOSTEXP -> WHRW4, ) (DASHDIETEXP -> WHRW4, ) ( Agew1_C-> WHRW4, ) (Agew3_C -> WHRW4, ) (sex -> WHRW4, ) (pir -> WHRW4, ) (edubr2 -> WHRW4, ) (edubr3 -> WHRW4, ) (employed1 -> WHRW4, )  (wrattbr2 -> WHRW4, ) (wrattbr3 -> WHRW4, )(wrattbr4 -> WHRW4, ) (smoke1 -> WHRW4, ) (smoke9 -> WHRW4, )(currdrugs1 -> WHRW4, ) (currdrugs9 -> WHRW4, ) (bmi_C -> WHRW4, ) (SRHbr2 -> WHRW4, ) (SRHbr3 -> WHRW4, )(kcalw1w3mean_C -> WHRW4, ) (energystoresw1w3mean_C -> WHRW4, ) (invmillssem -> WHRW4, ) ///
(FCOSTEXP -> DASHDIETEXP, )( Agew1_C-> DASHDIETEXP, ) (Agew3_C -> DASHDIETEXP, ) (sex -> DASHDIETEXP, ) (pir -> DASHDIETEXP, ) (edubr2 -> DASHDIETEXP, ) (edubr3 -> DASHDIETEXP, ) (employed1 -> DASHDIETEXP, ) (wrattbr2 -> DASHDIETEXP, ) (wrattbr3 -> DASHDIETEXP, ) (wrattbr4 -> DASHDIETEXP, )(smoke1 -> DASHDIETEXP, ) (smoke9 -> DASHDIETEXP, )(currdrugs1 -> DASHDIETEXP, ) (currdrugs9 -> DASHDIETEXP, ) (bmi_C -> DASHDIETEXP, ) (SRHbr2 -> DASHDIETEXP, ) (SRHbr3 -> DASHDIETEXP, )(kcalw1w3mean_C -> DASHDIETEXP, ) (energystoresw1w3mean_C -> DASHDIETEXP, ) (invmillssem -> DASHDIETEXP, ) if finalsample==1 & race==0 , nocapslatent method(ml)

estat gof, stats(all)
estat teffects



//African-Americans//
 sem (FCOSTEXP -> WHRW4, ) (DASHDIETEXP -> WHRW4, ) ( Agew1_C-> WHRW4, ) (Agew3_C -> WHRW4, ) (sex -> WHRW4, ) (pir -> WHRW4, ) (edubr2 -> WHRW4, ) (edubr3 -> WHRW4, ) (employed1 -> WHRW4, )  (wrattbr2 -> WHRW4, ) (wrattbr3 -> WHRW4, ) (wrattbr4 -> WHRW4, )(smoke1 -> WHRW4, ) (smoke9 -> WHRW4, )(currdrugs1 -> WHRW4, )
```

(currdrugs9 -> WHRW4,) (bmi_C -> WHRW4,) (SRHbr2 -> WHRW4,) (SRHbr3 -> WHRW4,)(kcalw1w3mean_C -> WHRW4,) (energystoresw1w3mean_C -> WHRW4,) (invmillssem -> WHRW4,) ///
(FCOSTEXP -> DASHDIETEXP,)(Agew1_C-> DASHDIETEXP,) (Agew3_C -> DASHDIETEXP,) (sex -> DASHDIETEXP,) (pir -> DASHDIETEXP,) (edubr2 -> DASHDIETEXP,) (edubr3 -> DASHDIETEXP,) (employed1 -> DASHDIETEXP,) (wrattbr2 -> DASHDIETEXP,) (wrattbr3 -> DASHDIETEXP,) (wrattbr4 -> DASHDIETEXP,)(smoke1 -> DASHDIETEXP,) (smoke9 -> DASHDIETEXP,)(currdrugs1 -> DASHDIETEXP,) (currdrugs9 -> DASHDIETEXP,) (bmi_C -> DASHDIETEXP,) (SRHbr2 -> DASHDIETEXP,) (SRHbr3 -> DASHDIETEXP,)(kcalw1w3mean_C -> DASHDIETEXP,) (energystoresw1w3mean_C -> DASHDIETEXP,) (invmillssem -> DASHDIETEXP,) if finalsample==1 & race==1 , nocapslatent method(ml)

estat gof, stats(all)
estat teffects

//BELOW POVERTY//
sem (FCOSTEXP -> WHRW4,) (DASHDIETEXP -> WHRW4,) (Agew1_C-> WHRW4,) (Agew3_C -> WHRW4,) (sex -> WHRW4,) (race -> WHRW4,) (edubr2 -> WHRW4,) (edubr3 -> WHRW4,) (employed1 -> WHRW4,) (wrattbr2 -> WHRW4,) (wrattbr3 -> WHRW4,) (wrattbr4 -> WHRW4,)(smoke1 -> WHRW4,) (smoke9 -> WHRW4,)(currdrugs1 -> WHRW4,) (currdrugs9 -> WHRW4,) (bmi_C -> WHRW4,) (SRHbr2 -> WHRW4,) (SRHbr3 -> WHRW4,)(kcalw1w3mean_C -> WHRW4,) (energystoresw1w3mean_C -> WHRW4,) (invmillssem -> WHRW4,) ///
(FCOSTEXP -> DASHDIETEXP,)(Agew1_C-> DASHDIETEXP,) (Agew3_C -> DASHDIETEXP,) (sex -> DASHDIETEXP,) (race -> DASHDIETEXP,) (edubr2 -> DASHDIETEXP,) (edubr3 -> DASHDIETEXP,) (employed1 -> DASHDIETEXP,) (wrattbr2 -> DASHDIETEXP,) (wrattbr3 -> DASHDIETEXP,) (wrattbr4 -> DASHDIETEXP,)(smoke1 -> DASHDIETEXP,) (smoke9 -> DASHDIETEXP,)(currdrugs1 -> DASHDIETEXP,) (currdrugs9 -> DASHDIETEXP,) (bmi_C -> DASHDIETEXP,) (SRHbr2 -> DASHDIETEXP,) (SRHbr3 -> DASHDIETEXP,)(kcalw1w3mean_C -> DASHDIETEXP,) (energystoresw1w3mean_C -> DASHDIETEXP,) (invmillssem -> DASHDIETEXP,) if finalsample==1 & pir==0 , nocapslatent method(ml)

estat gof, stats(all)
estat teffects

//ABOVE POVERTY//
sem (FCOSTEXP -> WHRW4,) (DASHDIETEXP -> WHRW4,) (Agew1_C-> WHRW4,) (Agew3_C -> WHRW4,) (sex -> WHRW4,) (race -> WHRW4,) (edubr2 -> WHRW4,) (edubr3 -> WHRW4,) (employed1 -> WHRW4,) (wrattbr2 -> WHRW4,) (wrattbr3 -> WHRW4,) (wrattbr4 -> WHRW4,)(smoke1 -> WHRW4,) (smoke9 -> WHRW4,)(currdrugs1 -> WHRW4,) (currdrugs9 -> WHRW4,) (bmi_C -> WHRW4,) (SRHbr2 -> WHRW4,) (SRHbr3 -> WHRW4,)(kcalw1w3mean_C -> WHRW4,) (energystoresw1w3mean_C -> WHRW4,) (invmillssem -> WHRW4,) ///
(FCOSTEXP -> DASHDIETEXP,)(Agew1_C-> DASHDIETEXP,) (Agew3_C -> DASHDIETEXP,) (sex -> DASHDIETEXP,) (race -> DASHDIETEXP,) (edubr2 -> DASHDIETEXP,) (edubr3 -> DASHDIETEXP,) (employed1 -> DASHDIETEXP,) (wrattbr2 -> DASHDIETEXP,) (wrattbr3 -> DASHDIETEXP,) (wrattbr4 -> DASHDIETEXP,)(smoke1 -> DASHDIETEXP,) (smoke9 -> DASHDIETEXP,)(currdrugs1 -> DASHDIETEXP,) (currdrugs9 -> DASHDIETEXP,) (bmi_C -> DASHDIETEXP,) (SRHbr2 -> DASHDIETEXP,) (SRHbr3 -> DASHDIETEXP,)(kcalw1w3mean_C -> DASHDIETEXP,) (energystoresw1w3mean_C -> DASHDIETEXP,) (invmillssem -> DASHDIETEXP,) if finalsample==1 & pir==1 , nocapslatent method(ml)

estat gof, stats(all)
estat teffects

****************************SBP WAVE 4**

//MULTI-GROUP//

All

sem (FCOSTEXP -> BPSYSW4 ,) (DASHDIETEXP -> BPSYSW4 ,) (Agew1_C-> BPSYSW4 ,) (Agew3_C -> BPSYSW4 ,) (sex -> BPSYSW4 ,) (race -> BPSYSW4 ,) (pir -> BPSYSW4 ,) (edubr2 -> BPSYSW4 ,) (edubr3 -> BPSYSW4 ,) (employed1 -> BPSYSW4 ,) (wrattbr2 -> BPSYSW4 ,) (wrattbr3 -> BPSYSW4 ,) (wrattbr4 -> BPSYSW4 ,)(smoke1 -> BPSYSW4 ,) (smoke9 -> BPSYSW4 ,)(currdrugs1 -> BPSYSW4 ,) (currdrugs9 -> BPSYSW4 ,) (bmi_C -> BPSYSW4 ,) (SRHbr2 -> BPSYSW4 ,) (SRHbr3 -> BPSYSW4 ,)(kcalw1w3mean_C -> BPSYSW4 ,) (energystoresw1w3mean_C -> BPSYSW4 ,) (invmillssem -> BPSYSW4 ,) /// (FCOSTEXP -> DASHDIETEXP,)(Agew1_C-> DASHDIETEXP,) (Agew3_C -> DASHDIETEXP,) (sex -> DASHDIETEXP,) (race -> DASHDIETEXP,) (pir -> DASHDIETEXP,) (edubr2 -> DASHDIETEXP,) (edubr3 -> DASHDIETEXP,) (employed1 -> DASHDIETEXP,) (wrattbr2 -> DASHDIETEXP,) (wrattbr3 -> DASHDIETEXP,) (wrattbr4 -> DASHDIETEXP,)(smoke1 -> DASHDIETEXP,) (smoke9 -> DASHDIETEXP,)(currdrugs1 -> DASHDIETEXP,) (currdrugs9 -> DASHDIETEXP,) (bmi_C -> DASHDIETEXP,) (SRHbr2 -> DASHDIETEXP,) (SRHbr3 -> DASHDIETEXP,)(kcalw1w3mean_C -> DASHDIETEXP,) (energystoresw1w3mean_C -> DASHDIETEXP,) (invmillssem -> DASHDIETEXP,) , nocapslatent method(ml) group(finalsample)

estat ginvariant

estat gof, stats(all)
estat teffects

By sex

sem (FCOSTEXP -> BPSYSW4 ,) (DASHDIETEXP -> BPSYSW4 ,) (Agew1_C-> BPSYSW4 ,) (Agew3_C -> BPSYSW4 ,) (race -> BPSYSW4 ,) (pir -> BPSYSW4 ,) (edubr2 -> BPSYSW4 ,) (edubr3 -> BPSYSW4 ,) (employed1 -> BPSYSW4 ,) (wrattbr2 -> BPSYSW4 ,) (wrattbr3 -> BPSYSW4 ,) (wrattbr4 -> BPSYSW4 ,)(smoke1 -> BPSYSW4 ,) (smoke9 -> BPSYSW4 ,)(currdrugs1 -> BPSYSW4 ,) (currdrugs9 -> BPSYSW4 ,) (bmi_C -> BPSYSW4 ,) (SRHbr2 -> BPSYSW4 ,) (SRHbr3 -> BPSYSW4 ,)(kcalw1w3mean_C -> BPSYSW4 ,) (energystoresw1w3mean_C -> BPSYSW4 ,) (invmillssem -> BPSYSW4 ,) /// (FCOSTEXP -> DASHDIETEXP,)(Agew1_C-> DASHDIETEXP,) (Agew3_C -> DASHDIETEXP,) (race -> DASHDIETEXP,) (pir -> DASHDIETEXP,) (edubr2 -> DASHDIETEXP,) (edubr3 -> DASHDIETEXP,) (employed1 -> DASHDIETEXP,) (wrattbr2 -> DASHDIETEXP,) (wrattbr3 -> DASHDIETEXP,)(wrattbr4 -> DASHDIETEXP,) (smoke1 -> DASHDIETEXP,) (smoke9 -> DASHDIETEXP,)(currdrugs1 -> DASHDIETEXP,) (currdrugs9 -> DASHDIETEXP,) (bmi_C -> DASHDIETEXP,) (SRHbr2 -> DASHDIETEXP,) (SRHbr3 -> DASHDIETEXP,)(kcalw1w3mean_C -> DASHDIETEXP,)

(energystoresw1w3mean_C -> DASHDIETEXP,) (invmillssem -> DASHDIETEXP,) if finalsample==1 , nocapslatent method(ml) group(sex)

estat ginvariant

By race

sem (FCOSTEXP -> BPSYSW4 ,) (DASHDIETEXP -> BPSYSW4 ,) (Agew1_C-> BPSYSW4 ,) (Agew3_C -> BPSYSW4 ,) (sex -> BPSYSW4 ,) (pir -> BPSYSW4 ,) (edubr2 -> BPSYSW4 ,) (edubr3 -> BPSYSW4 ,) (employed1 -> BPSYSW4 ,) (wrattbr2 -> BPSYSW4 ,) (wrattbr3 -> BPSYSW4 ,) (wrattbr4 -> BPSYSW4 ,)(smoke1 -> BPSYSW4 ,) (smoke9 -> BPSYSW4 ,)(currdrugs1 -> BPSYSW4 ,) (currdrugs9 -> BPSYSW4 ,) (bmi_C -> BPSYSW4 ,) (SRHbr2 -> BPSYSW4 ,) (SRHbr3 -> BPSYSW4 ,)(kcalw1w3mean_C -> BPSYSW4 ,) (energystoresw1w3mean_C -> BPSYSW4 ,) (invmillssem -> BPSYSW4 ,) /// (FCOSTEXP -> DASHDIETEXP,)(Agew1_C-> DASHDIETEXP,) (Agew3_C -> DASHDIETEXP,) (sex -> DASHDIETEXP,) (pir -> DASHDIETEXP,) (edubr2 -> DASHDIETEXP,) (edubr3 -> DASHDIETEXP,) (employed1 -> DASHDIETEXP,) (wrattbr2 -> DASHDIETEXP,) (wrattbr3 -> DASHDIETEXP,) (wrattbr4 -> DASHDIETEXP,)(smoke1 -> DASHDIETEXP,) (smoke9 -> DASHDIETEXP,)(currdrugs1 -> DASHDIETEXP,) (currdrugs9 -> DASHDIETEXP,) (bmi_C -> DASHDIETEXP,) (SRHbr2 -> DASHDIETEXP,) (SRHbr3 -> DASHDIETEXP,)(kcalw1w3mean_C -> DASHDIETEXP,) (energystoresw1w3mean_C -> DASHDIETEXP,) (invmillssem -> DASHDIETEXP,) if finalsample==1 , nocapslatent method(ml) group(race)

estat ginvariant

By pir

sem (FCOSTEXP -> BPSYSW4 ,) (DASHDIETEXP -> BPSYSW4 ,) (Agew1_C-> BPSYSW4 ,) (Agew3_C -> BPSYSW4 ,) (sex -> BPSYSW4 ,) (race -> BPSYSW4 ,) (edubr2 -> BPSYSW4 ,) (edubr3 -> BPSYSW4 ,) (employed1 -> BPSYSW4 ,) (wrattbr2 -> BPSYSW4 ,) (wrattbr3 -> BPSYSW4 ,) (wrattbr4 -> BPSYSW4 ,)(smoke1 -> BPSYSW4 ,) (smoke9 -> BPSYSW4 ,)(currdrugs1 -> BPSYSW4 ,) (currdrugs9 -> BPSYSW4 ,) (bmi_C -> BPSYSW4 ,) (SRHbr2 -> BPSYSW4 ,) (SRHbr3 -> BPSYSW4 ,)(kcalw1w3mean_C -> BPSYSW4 ,) (energystoresw1w3mean_C -> BPSYSW4 ,) (invmillssem -> BPSYSW4 ,) /// (FCOSTEXP -> DASHDIETEXP,)(Agew1_C-> DASHDIETEXP,) (Agew3_C -> DASHDIETEXP,) (sex -> DASHDIETEXP,) (race -> DASHDIETEXP,) (edubr2 -> DASHDIETEXP,) (edubr3 -> DASHDIETEXP,) (employed1 -> DASHDIETEXP,) (wrattbr2 -> DASHDIETEXP,) (wrattbr3 -> DASHDIETEXP,) (wrattbr4 -> DASHDIETEXP,)(smoke1 -> DASHDIETEXP,) (smoke9 -> DASHDIETEXP,)(currdrugs1 -> DASHDIETEXP,) (currdrugs9 -> DASHDIETEXP,) (bmi_C -> DASHDIETEXP,) (SRHbr2 -> DASHDIETEXP,) (SRHbr3 -> DASHDIETEXP,)(kcalw1w3mean_C -> DASHDIETEXP,) (energystoresw1w3mean_C -> DASHDIETEXP,) (invmillssem -> DASHDIETEXP,) if finalsample==1 , nocapslatent method(ml) group(pir)

estat ginvariant

*********************STRATIFIED ANALYSIS********************************

//MEN//

sem (FCOSTEXP -> BPSYSW4 ,) (DASHDIETEXP -> BPSYSW4 ,) (Agew1_C-> BPSYSW4 ,) (Agew3_C -> BPSYSW4 ,) (race -> BPSYSW4 ,) (pir -> BPSYSW4 ,) (edubr2 -> BPSYSW4 ,) (edubr3 -> BPSYSW4 ,) (employed1 -> BPSYSW4 ,) (wrattbr2 -> BPSYSW4 ,) (wrattbr3 -> BPSYSW4 ,) (wrattbr4 -> BPSYSW4 ,)(smoke1 -> BPSYSW4 ,) (smoke9 -> BPSYSW4 ,)(currdrugs1 -> BPSYSW4 ,) (currdrugs9 -> BPSYSW4 ,) (bmi_C -> BPSYSW4 ,) (SRHbr2 -> BPSYSW4 ,) (SRHbr3 -> BPSYSW4 ,)(kcalw1w3mean_C -> BPSYSW4 ,) (energystoresw1w3mean_C -> BPSYSW4 ,) (invmillssem -> BPSYSW4 ,) /// (FCOSTEXP -> DASHDIETEXP,)(Agew1_C-> DASHDIETEXP,) (Agew3_C -> DASHDIETEXP,) (race -> DASHDIETEXP,) (pir -> DASHDIETEXP,) (edubr2 -> DASHDIETEXP,) (edubr3 -> DASHDIETEXP,) (employed1 -> DASHDIETEXP,) (wrattbr2 -> DASHDIETEXP,) (wrattbr3 -> DASHDIETEXP,)(wrattbr4 -> DASHDIETEXP,) (smoke1 -> DASHDIETEXP,) (smoke9 -> DASHDIETEXP,)(currdrugs1 -> DASHDIETEXP,) (currdrugs9 -> DASHDIETEXP,) (bmi_C -> DASHDIETEXP,) (SRHbr2 -> DASHDIETEXP,) (SRHbr3 -> DASHDIETEXP,)(kcalw1w3mean_C -> DASHDIETEXP,) (energystoresw1w3mean_C -> DASHDIETEXP,) (invmillssem -> DASHDIETEXP,) if finalsample==1 & sex==1 , nocapslatent method(ml)

estat gof, stats(all)
estat teffects

//WOMEN//
sem (FCOSTEXP -> BPSYSW4 ,) (DASHDIETEXP -> BPSYSW4 ,) (Agew1_C-> BPSYSW4 ,) (Agew3_C -> BPSYSW4 ,) (race -> BPSYSW4 ,) (pir -> BPSYSW4 ,) (edubr2 -> BPSYSW4 ,) (edubr3 -> BPSYSW4 ,) (employed1 -> BPSYSW4 ,) (wrattbr2 -> BPSYSW4 ,) (wrattbr3 -> BPSYSW4 ,) (wrattbr4 -> BPSYSW4 ,)(smoke1 -> BPSYSW4 ,) (smoke9 -> BPSYSW4 ,)(currdrugs1 -> BPSYSW4 ,) (currdrugs9 -> BPSYSW4 ,) (bmi_C -> BPSYSW4 ,) (SRHbr2 -> BPSYSW4 ,) (SRHbr3 -> BPSYSW4 ,)(kcalw1w3mean_C -> BPSYSW4 ,) (energystoresw1w3mean_C -> BPSYSW4 ,) (invmillssem -> BPSYSW4 ,) /// (FCOSTEXP -> DASHDIETEXP,)(Agew1_C-> DASHDIETEXP,) (Agew3_C -> DASHDIETEXP,) (race -> DASHDIETEXP,) (pir -> DASHDIETEXP,) (edubr2 -> DASHDIETEXP,) (edubr3 -> DASHDIETEXP,) (employed1 -> DASHDIETEXP,) (wrattbr2 -> DASHDIETEXP,) (wrattbr3 -> DASHDIETEXP,) (wrattbr4 -> DASHDIETEXP,)(smoke1 -> DASHDIETEXP,) (smoke9 -> DASHDIETEXP,)(currdrugs1 -> DASHDIETEXP,) (currdrugs9 -> DASHDIETEXP,) (bmi_C -> DASHDIETEXP,) (SRHbr2 -> DASHDIETEXP,) (SRHbr3 -> DASHDIETEXP,)(kcalw1w3mean_C -> DASHDIETEXP,) (energystoresw1w3mean_C -> DASHDIETEXP,) (invmillssem -> DASHDIETEXP,) if finalsample==1 & sex==0 , nocapslatent method(ml)

estat gof, stats(all)
estat teffects

//WHITES//
sem (FCOSTEXP -> BPSYSW4 ,) (DASHDIETEXP -> BPSYSW4 ,) (Agew1_C-> BPSYSW4 ,) (Agew3_C -> BPSYSW4 ,) (sex -> BPSYSW4 ,) (pir -> BPSYSW4 ,) (edubr2 -> BPSYSW4 ,) (edubr3 -> BPSYSW4 ,) (employed1 -> BPSYSW4 ,) (wrattbr2 -> BPSYSW4 ,) (wrattbr3 -> BPSYSW4 ,)(wrattbr4 -> BPSYSW4 ,) (smoke1 -> BPSYSW4 ,) (smoke9 -> BPSYSW4 ,)(currdrugs1 -> BPSYSW4 ,) (currdrugs9 -> BPSYSW4 ,) (bmi_C -> BPSYSW4 ,) (SRHbr2 -> BPSYSW4 ,) (SRHbr3 -> BPSYSW4 ,)(kcalw1w3mean_C -> BPSYSW4 ,) (energystoresw1w3mean_C -> BPSYSW4 ,) (invmillssem -> BPSYSW4 ,) /// (FCOSTEXP -> DASHDIETEXP,)(Agew1_C-> DASHDIETEXP,) (Agew3_C -> DASHDIETEXP,) (sex -> DASHDIETEXP,) (pir -> DASHDIETEXP,) (edubr2 -> DASHDIETEXP,) (edubr3 -> DASHDIETEXP,) (employed1 -> DASHDIETEXP,) (wrattbr2 -> DASHDIETEXP,) (wrattbr3 -> DASHDIETEXP,) (wrattbr4 -> DASHDIETEXP,)(smoke1 -> DASHDIETEXP,)

(smoke9 -> DASHDIETEXP,)(currdrugs1 -> DASHDIETEXP,) (currdrugs9 -> DASHDIETEXP,) (bmi_C -> DASHDIETEXP,) (SRHbr2 -> DASHDIETEXP,) (SRHbr3 -> DASHDIETEXP,)(kcalw1w3mean_C -> DASHDIETEXP,) (energystoresw1w3mean_C -> DASHDIETEXP,) (invmillssem -> DASHDIETEXP,) if finalsample==1 & race==0 , nocapslatent method(ml)

estat gof, stats(all)
estat teffects

//African-Americans//
sem (FCOSTEXP -> BPSYSW4 ,) (DASHDIETEXP -> BPSYSW4 ,) (Agew1_C-> BPSYSW4 ,) (Agew3_C -> BPSYSW4 ,) (sex -> BPSYSW4 ,) (pir -> BPSYSW4 ,) (edubr2 -> BPSYSW4 ,) (edubr3 -> BPSYSW4 ,) (employed1 -> BPSYSW4 ,) (wrattbr2 -> BPSYSW4 ,) (wrattbr3 -> BPSYSW4 ,) (wrattbr4 -> BPSYSW4 ,)(smoke1 -> BPSYSW4 ,) (smoke9 -> BPSYSW4 ,)(currdrugs1 -> BPSYSW4 ,) (currdrugs9 -> BPSYSW4 ,) (bmi_C -> BPSYSW4 ,) (SRHbr2 -> BPSYSW4 ,) (SRHbr3 -> BPSYSW4 ,)(kcalw1w3mean_C -> BPSYSW4 ,) (energystoresw1w3mean_C -> BPSYSW4 ,) (invmillssem -> BPSYSW4 ,) /// (FCOSTEXP -> DASHDIETEXP,)(Agew1_C-> DASHDIETEXP,) (Agew3_C -> DASHDIETEXP,) (sex -> DASHDIETEXP,) (pir -> DASHDIETEXP,) (edubr2 -> DASHDIETEXP,) (edubr3 -> DASHDIETEXP,) (employed1 -> DASHDIETEXP,) (wrattbr2 -> DASHDIETEXP,) (wrattbr3 -> DASHDIETEXP,) (wrattbr4 -> DASHDIETEXP,)(smoke1 -> DASHDIETEXP,) (smoke9 -> DASHDIETEXP,)(currdrugs1 -> DASHDIETEXP,) (currdrugs9 -> DASHDIETEXP,) (bmi_C -> DASHDIETEXP,) (SRHbr2 -> DASHDIETEXP,) (SRHbr3 -> DASHDIETEXP,)(kcalw1w3mean_C -> DASHDIETEXP,) (energystoresw1w3mean_C -> DASHDIETEXP,) (invmillssem -> DASHDIETEXP,) if finalsample==1 & race==1 , nocapslatent method(ml)

estat gof, stats(all)
estat teffects

//BELOW POVERTY//
sem (FCOSTEXP -> BPSYSW4 ,) (DASHDIETEXP -> BPSYSW4 ,) (Agew1_C-> BPSYSW4 ,) (Agew3_C -> BPSYSW4 ,) (sex -> BPSYSW4 ,) (race -> BPSYSW4 ,) (edubr2 -> BPSYSW4 ,) (edubr3 -> BPSYSW4 ,) (employed1 -> BPSYSW4 ,) (wrattbr2 -> BPSYSW4 ,) (wrattbr3 -> BPSYSW4 ,) (wrattbr4 -> BPSYSW4 ,)(smoke1 -> BPSYSW4 ,) (smoke9 -> BPSYSW4 ,)(currdrugs1 -> BPSYSW4 ,) (currdrugs9 -> BPSYSW4 ,) (bmi_C -> BPSYSW4 ,) (SRHbr2 -> BPSYSW4 ,) (SRHbr3 -> BPSYSW4 ,)(kcalw1w3mean_C -> BPSYSW4 ,) (energystoresw1w3mean_C -> BPSYSW4 ,) (invmillssem -> BPSYSW4 ,) /// (FCOSTEXP -> DASHDIETEXP,)(Agew1_C-> DASHDIETEXP,) (Agew3_C -> DASHDIETEXP,) (sex -> DASHDIETEXP,) (race -> DASHDIETEXP,) (edubr2 -> DASHDIETEXP,) (edubr3 -> DASHDIETEXP,) (employed1 -> DASHDIETEXP,) (wrattbr2 -> DASHDIETEXP,) (wrattbr3 -> DASHDIETEXP,) (wrattbr4 -> DASHDIETEXP,)(smoke1 -> DASHDIETEXP,) (smoke9 -> DASHDIETEXP,)(currdrugs1 -> DASHDIETEXP,) (currdrugs9 -> DASHDIETEXP,) (bmi_C -> DASHDIETEXP,) (SRHbr2 -> DASHDIETEXP,) (SRHbr3 -> DASHDIETEXP,)(kcalw1w3mean_C -> DASHDIETEXP,) (energystoresw1w3mean_C -> DASHDIETEXP,) (invmillssem -> DASHDIETEXP,) if finalsample==1 & pir==0 , nocapslatent method(ml)

estat gof, stats(all)
estat teffects

//ABOVE POVERTY//

sem (FCOSTEXP -> BPSYSW4 ,) (DASHDIETEXP -> BPSYSW4 ,) (Agew1_C-> BPSYSW4 ,) (Agew3_C -> BPSYSW4 ,) (sex -> BPSYSW4 ,) (race -> BPSYSW4 ,) (edubr2 -> BPSYSW4 ,) (edubr3 -> BPSYSW4 ,) (employed1 -> BPSYSW4 ,) (wrattbr2 -> BPSYSW4 ,) (wrattbr3 -> BPSYSW4 ,) (wrattbr4 -> BPSYSW4 ,)(smoke1 -> BPSYSW4 ,) (smoke9 -> BPSYSW4 ,)(currdrugs1 -> BPSYSW4 ,) (currdrugs9 -> BPSYSW4 ,) (bmi_C -> BPSYSW4 ,) (SRHbr2 -> BPSYSW4 ,) (SRHbr3 -> BPSYSW4 ,)(kcalw1w3mean_C -> BPSYSW4 ,) (energystoresw1w3mean_C -> BPSYSW4 ,) (invmillssem -> BPSYSW4 ,) /// (FCOSTEXP -> DASHDIETEXP,)(Agew1_C-> DASHDIETEXP,) (Agew3_C -> DASHDIETEXP,) (sex -> DASHDIETEXP,) (race -> DASHDIETEXP,) (edubr2 -> DASHDIETEXP,) (edubr3 -> DASHDIETEXP,) (employed1 -> DASHDIETEXP,) (wrattbr2 -> DASHDIETEXP,) (wrattbr3 -> DASHDIETEXP,) (wrattbr4 -> DASHDIETEXP,)(smoke1 -> DASHDIETEXP,) (smoke9 -> DASHDIETEXP,)(currdrugs1 -> DASHDIETEXP,) (currdrugs9 -> DASHDIETEXP,) (bmi_C -> DASHDIETEXP,) (SRHbr2 -> DASHDIETEXP,) (SRHbr3 -> DASHDIETEXP,)(kcalw1w3mean_C -> DASHDIETEXP,) (energystoresw1w3mean_C -> DASHDIETEXP,) (invmillssem -> DASHDIETEXP,) if finalsample==1 & pir==1 , nocapslatent method(ml)

estat gof, stats(all)

estat teffects

****************************DBP WAVE 4***

//MULTI-GROUP//

All

sem (FCOSTEXP -> BPDIAW4 ,) (DASHDIETEXP -> BPDIAW4 ,) (Agew1_C-> BPDIAW4 ,) (Agew3_C -> BPDIAW4 ,) (sex -> BPDIAW4 ,) (race -> BPDIAW4 ,) (pir -> BPDIAW4 ,) (edubr2 -> BPDIAW4 ,) (edubr3 -> BPDIAW4 ,) (employed1 -> BPDIAW4 ,) (wrattbr2 -> BPDIAW4 ,) (wrattbr3 -> BPDIAW4 ,) (wrattbr4 -> BPDIAW4 ,)(smoke1 -> BPDIAW4 ,) (smoke9 -> BPDIAW4 ,)(currdrugs1 -> BPDIAW4 ,) (currdrugs9 -> BPDIAW4 ,) (bmi_C -> BPDIAW4 ,) (SRHbr2 -> BPDIAW4 ,) (SRHbr3 -> BPDIAW4 ,)(kcalw1w3mean_C -> BPDIAW4 ,) (energystoresw1w3mean_C -> BPDIAW4 ,) (invmillssem -> BPDIAW4 ,) ///

(FCOSTEXP -> DASHDIETEXP,)(Agew1_C-> DASHDIETEXP,) (Agew3_C -> DASHDIETEXP,) (sex -> DASHDIETEXP,) (race -> DASHDIETEXP,) (pir -> DASHDIETEXP,) (edubr2 -> DASHDIETEXP,) (edubr3 -> DASHDIETEXP,) (employed1 -> DASHDIETEXP,) (wrattbr2 -> DASHDIETEXP,) (wrattbr3 -> DASHDIETEXP,) (wrattbr4 -> DASHDIETEXP,)(smoke1 -> DASHDIETEXP,) (smoke9 -> DASHDIETEXP,)(currdrugs1 -> DASHDIETEXP,) (currdrugs9 -> DASHDIETEXP,) (bmi_C -> DASHDIETEXP,) (SRHbr2 -> DASHDIETEXP,) (SRHbr3 -> DASHDIETEXP,)(kcalw1w3mean_C -> DASHDIETEXP,) (energystoresw1w3mean_C -> DASHDIETEXP,) (invmillssem -> DASHDIETEXP,) , nocapslatent method(ml) group(finalsample)

estat ginvariant

estat gof, stats(all)

estat teffects

By sex

sem (FCOSTEXP -> BPDIAW4 ,) (DASHDIETEXP -> BPDIAW4 ,) (Agew1_C-> BPDIAW4 ,) (Agew3_C -> BPDIAW4 ,) (race -> BPDIAW4 ,) (pir -> BPDIAW4 ,) (edubr2 -> BPDIAW4 ,) (edubr3 -> BPDIAW4 ,) (employed1 -> BPDIAW4 ,) (wrattbr2 -> BPDIAW4 ,) (wrattbr3 -> BPDIAW4 ,) (wrattbr4 -> BPDIAW4 ,)(smoke1 -> BPDIAW4 ,) (smoke9 -> BPDIAW4 ,)(currdrugs1 -> BPDIAW4 ,) (currdrugs9 -> BPDIAW4 ,) (bmi_C -> BPDIAW4 ,) (SRHbr2 -> BPDIAW4 ,) (SRHbr3 -> BPDIAW4 ,)(kcalw1w3mean_C -> BPDIAW4 ,) (energystoresw1w3mean_C -> BPDIAW4 ,) (invmillssem -> BPDIAW4 ,) /// (FCOSTEXP -> DASHDIETEXP,)(Agew1_C-> DASHDIETEXP,) (Agew3_C -> DASHDIETEXP,) (race -> DASHDIETEXP,) (pir -> DASHDIETEXP,) (edubr2 -> DASHDIETEXP,) (edubr3 -> DASHDIETEXP,) (employed1 -> DASHDIETEXP,) (wrattbr2 -> DASHDIETEXP,) (wrattbr3 -> DASHDIETEXP,)(wrattbr4 -> DASHDIETEXP,) (smoke1 -> DASHDIETEXP,) (smoke9 -> DASHDIETEXP,)(currdrugs1 -> DASHDIETEXP,) (currdrugs9 -> DASHDIETEXP,) (bmi_C -> DASHDIETEXP,) (SRHbr2 -> DASHDIETEXP,) (SRHbr3 -> DASHDIETEXP,)(kcalw1w3mean_C -> DASHDIETEXP,) (energystoresw1w3mean_C -> DASHDIETEXP,) (invmillssem -> DASHDIETEXP,) if finalsample==1 , nocapslatent method(ml) group(sex)

estat ginvariant

By race

sem (FCOSTEXP -> BPDIAW4 ,) (DASHDIETEXP -> BPDIAW4 ,) (Agew1_C-> BPDIAW4 ,) (Agew3_C -> BPDIAW4 ,) (sex -> BPDIAW4 ,) (pir -> BPDIAW4 ,) (edubr2 -> BPDIAW4 ,) (edubr3 -> BPDIAW4 ,) (employed1 -> BPDIAW4 ,) (wrattbr2 -> BPDIAW4 ,) (wrattbr3 -> BPDIAW4 ,) (wrattbr4 -> BPDIAW4 ,)(smoke1 -> BPDIAW4 ,) (smoke9 -> BPDIAW4 ,)(currdrugs1 -> BPDIAW4 ,) (currdrugs9 -> BPDIAW4 ,) (bmi_C -> BPDIAW4 ,) (SRHbr2 -> BPDIAW4 ,) (SRHbr3 -> BPDIAW4 ,)(kcalw1w3mean_C -> BPDIAW4 ,) (energystoresw1w3mean_C -> BPDIAW4 ,) (invmillssem -> BPDIAW4 ,) /// (FCOSTEXP -> DASHDIETEXP,)(Agew1_C-> DASHDIETEXP,) (Agew3_C -> DASHDIETEXP,) (sex -> DASHDIETEXP,) (pir -> DASHDIETEXP,) (edubr2 -> DASHDIETEXP,) (edubr3 -> DASHDIETEXP,) (employed1 -> DASHDIETEXP,) (wrattbr2 -> DASHDIETEXP,) (wrattbr3 -> DASHDIETEXP,) (wrattbr4 -> DASHDIETEXP,)(smoke1 -> DASHDIETEXP,) (smoke9 -> DASHDIETEXP,)(currdrugs1 -> DASHDIETEXP,) (currdrugs9 -> DASHDIETEXP,) (bmi_C -> DASHDIETEXP,) (SRHbr2 -> DASHDIETEXP,) (SRHbr3 -> DASHDIETEXP,)(kcalw1w3mean_C -> DASHDIETEXP,) (energystoresw1w3mean_C -> DASHDIETEXP,) (invmillssem -> DASHDIETEXP,) if finalsample==1 , nocapslatent method(ml) group(race)

estat ginvariant

By pir

sem (FCOSTEXP -> BPDIAW4 ,) (DASHDIETEXP -> BPDIAW4 ,) (Agew1_C-> BPDIAW4 ,) (Agew3_C -> BPDIAW4 ,) (sex -> BPDIAW4 ,) (race -> BPDIAW4 ,) (edubr2 -> BPDIAW4 ,) (edubr3 -> BPDIAW4 ,) (employed1 -> BPDIAW4 ,) (wrattbr2 -> BPDIAW4 ,) (wrattbr3 -> BPDIAW4 ,) (wrattbr4 -> BPDIAW4 ,)(smoke1 -> BPDIAW4 ,) (smoke9 -> BPDIAW4 ,)(currdrugs1 -> BPDIAW4 ,) (currdrugs9 -> BPDIAW4 ,) (bmi_C -> BPDIAW4 ,) (SRHbr2 -> BPDIAW4 ,) (SRHbr3 -> BPDIAW4 ,)(kcalw1w3mean_C -> BPDIAW4 ,) (energystoresw1w3mean_C -> BPDIAW4 ,) (invmillssem -> BPDIAW4 ,) ///

(FCOSTEXP -> DASHDIETEXP,)(Agew1_C-> DASHDIETEXP,) (Agew3_C -> DASHDIETEXP,) (sex -> DASHDIETEXP,) (race -> DASHDIETEXP,) (edubr2 -> DASHDIETEXP,) (edubr3 -> DASHDIETEXP,) (employed1 -> DASHDIETEXP,) (wrattbr2 -> DASHDIETEXP,) (wrattbr3 -> DASHDIETEXP,) (wrattbr4 -> DASHDIETEXP,)(smoke1 -> DASHDIETEXP,) (smoke9 -> DASHDIETEXP,)(currdrugs1 -> DASHDIETEXP,) (currdrugs9 -> DASHDIETEXP,) (bmi_C -> DASHDIETEXP,) (SRHbr2 -> DASHDIETEXP,) (SRHbr3 -> DASHDIETEXP,)(kcalw1w3mean_C -> DASHDIETEXP,) (energystoresw1w3mean_C -> DASHDIETEXP,) (invmillssem -> DASHDIETEXP,) if finalsample==1 , nocapslatent method(ml) group(pir)

estat ginvariant

********************STRATIFIED ANALYSIS******************************

//MEN//

sem (FCOSTEXP -> BPDIAW4 ,) (DASHDIETEXP -> BPDIAW4 ,) (Agew1_C-> BPDIAW4 ,) (Agew3_C -> BPDIAW4 ,) (race -> BPDIAW4 ,) (pir -> BPDIAW4 ,) (edubr2 -> BPDIAW4 ,) (edubr3 -> BPDIAW4 ,) (employed1 -> BPDIAW4 ,) (wrattbr2 -> BPDIAW4 ,) (wrattbr3 -> BPDIAW4 ,) (wrattbr4 -> BPDIAW4 ,)(smoke1 -> BPDIAW4 ,) (smoke9 -> BPDIAW4 ,)(currdrugs1 -> BPDIAW4 ,) (currdrugs9 -> BPDIAW4 ,) (bmi_C -> BPDIAW4 ,) (SRHbr2 -> BPDIAW4 ,) (SRHbr3 -> BPDIAW4 ,)(kcalw1w3mean_C -> BPDIAW4 ,) (energystoresw1w3mean_C -> BPDIAW4 ,) (invmillssem -> BPDIAW4 ,) /// (FCOSTEXP -> DASHDIETEXP,)(Agew1_C-> DASHDIETEXP,) (Agew3_C -> DASHDIETEXP,) (race -> DASHDIETEXP,) (pir -> DASHDIETEXP,) (edubr2 -> DASHDIETEXP,) (edubr3 -> DASHDIETEXP,) (employed1 -> DASHDIETEXP,) (wrattbr2 -> DASHDIETEXP,) (wrattbr3 -> DASHDIETEXP,)(wrattbr4 -> DASHDIETEXP,) (smoke1 -> DASHDIETEXP,) (smoke9 -> DASHDIETEXP,)(currdrugs1 -> DASHDIETEXP,) (currdrugs9 -> DASHDIETEXP,) (bmi_C -> DASHDIETEXP,) (SRHbr2 -> DASHDIETEXP,) (SRHbr3 -> DASHDIETEXP,)(kcalw1w3mean_C -> DASHDIETEXP,) (energystoresw1w3mean_C -> DASHDIETEXP,) (invmillssem -> DASHDIETEXP,) if finalsample==1 & sex==1 , nocapslatent method(ml)

estat gof, stats(all)
estat teffects

//WOMEN//

sem (FCOSTEXP -> BPDIAW4 ,) (DASHDIETEXP -> BPDIAW4 ,) (Agew1_C-> BPDIAW4 ,) (Agew3_C -> BPDIAW4 ,) (race -> BPDIAW4 ,) (pir -> BPDIAW4 ,) (edubr2 -> BPDIAW4 ,) (edubr3 -> BPDIAW4 ,) (employed1 -> BPDIAW4 ,) (wrattbr2 -> BPDIAW4 ,) (wrattbr3 -> BPDIAW4 ,) (wrattbr4 -> BPDIAW4 ,)(smoke1 -> BPDIAW4 ,) (smoke9 -> BPDIAW4 ,)(currdrugs1 -> BPDIAW4 ,) (currdrugs9 -> BPDIAW4 ,) (bmi_C -> BPDIAW4 ,) (SRHbr2 -> BPDIAW4 ,) (SRHbr3 -> BPDIAW4 ,)(kcalw1w3mean_C -> BPDIAW4 ,) (energystoresw1w3mean_C -> BPDIAW4 ,) (invmillssem -> BPDIAW4 ,) /// (FCOSTEXP -> DASHDIETEXP,)(Agew1_C-> DASHDIETEXP,) (Agew3_C -> DASHDIETEXP,) (race -> DASHDIETEXP,) (pir -> DASHDIETEXP,) (edubr2 -> DASHDIETEXP,) (edubr3 -> DASHDIETEXP,) (employed1 -> DASHDIETEXP,) (wrattbr2 -> DASHDIETEXP,) (wrattbr3 -> DASHDIETEXP,) (wrattbr4 -> DASHDIETEXP,)(smoke1 -> DASHDIETEXP,) (smoke9 -> DASHDIETEXP,)(currdrugs1 -> DASHDIETEXP,) (currdrugs9 -> DASHDIETEXP,) (bmi_C -> DASHDIETEXP,) (SRHbr2 -> DASHDIETEXP,) (SRHbr3 -> DASHDIETEXP,)(kcalw1w3mean_C -> DASHDIETEXP,) (energystoresw1w3mean_C -> DASHDIETEXP,) (invmillssem -> DASHDIETEXP,) if finalsample==1 & sex==0 , nocapslatent method(ml)

```
estat gof, stats(all)
estat teffects



//WHITES//
  sem (FCOSTEXP -> BPDIAW4 , ) (DASHDIETEXP -> BPDIAW4 , ) ( Agew1_C-> BPDIAW4 , ) (Agew3_C -> BPDIAW4 , ) (sex -> BPDIAW4 , ) (pir -> BPDIAW4 , ) (edubr2 -> BPDIAW4 , ) (edubr3 -> BPDIAW4 , ) (employed1 -> BPDIAW4 , )   (wrattbr2 -> BPDIAW4 , ) (wrattbr3 -> BPDIAW4 , )(wrattbr4 -> BPDIAW4 , ) (smoke1 -> BPDIAW4 , ) (smoke9 -> BPDIAW4 , )(currdrugs1 -> BPDIAW4 , ) (currdrugs9 -> BPDIAW4 , ) (bmi_C -> BPDIAW4 , ) (SRHbr2 -> BPDIAW4 , ) (SRHbr3 -> BPDIAW4 , )(kcalw1w3mean_C -> BPDIAW4 , ) (energystoresw1w3mean_C -> BPDIAW4 , ) (invmillssem -> BPDIAW4 , ) ///
(FCOSTEXP -> DASHDIETEXP, )( Agew1_C-> DASHDIETEXP, ) (Agew3_C -> DASHDIETEXP, ) (sex -> DASHDIETEXP, ) (pir -> DASHDIETEXP, ) (edubr2 -> DASHDIETEXP, ) (edubr3 -> DASHDIETEXP, ) (employed1 -> DASHDIETEXP, ) (wrattbr2 -> DASHDIETEXP, ) (wrattbr3 -> DASHDIETEXP, ) (wrattbr4 -> DASHDIETEXP, )(smoke1 -> DASHDIETEXP, ) (smoke9 -> DASHDIETEXP, )(currdrugs1 -> DASHDIETEXP, ) (currdrugs9 -> DASHDIETEXP, ) (bmi_C -> DASHDIETEXP, ) (SRHbr2 -> DASHDIETEXP, ) (SRHbr3 -> DASHDIETEXP, )(kcalw1w3mean_C -> DASHDIETEXP, ) (energystoresw1w3mean_C -> DASHDIETEXP, ) (invmillssem -> DASHDIETEXP, ) if finalsample==1 & race==0 , nocapslatent method(ml)

estat gof, stats(all)
estat teffects



//African-Americans//
  sem (FCOSTEXP -> BPDIAW4 , ) (DASHDIETEXP -> BPDIAW4 , ) ( Agew1_C-> BPDIAW4 , ) (Agew3_C -> BPDIAW4 , ) (sex -> BPDIAW4 , ) (pir -> BPDIAW4 , ) (edubr2 -> BPDIAW4 , ) (edubr3 -> BPDIAW4 , ) (employed1 -> BPDIAW4 , )   (wrattbr2 -> BPDIAW4 , ) (wrattbr3 -> BPDIAW4 , ) (wrattbr4 -> BPDIAW4 , )(smoke1 -> BPDIAW4 , ) (smoke9 -> BPDIAW4 , )(currdrugs1 -> BPDIAW4 , ) (currdrugs9 -> BPDIAW4 , ) (bmi_C -> BPDIAW4 , ) (SRHbr2 -> BPDIAW4 , ) (SRHbr3 -> BPDIAW4 , )(kcalw1w3mean_C -> BPDIAW4 , ) (energystoresw1w3mean_C -> BPDIAW4 , ) (invmillssem -> BPDIAW4 , ) ///
(FCOSTEXP -> DASHDIETEXP, )( Agew1_C-> DASHDIETEXP, ) (Agew3_C -> DASHDIETEXP, ) (sex -> DASHDIETEXP, ) (pir -> DASHDIETEXP, ) (edubr2 -> DASHDIETEXP, ) (edubr3 -> DASHDIETEXP, ) (employed1 -> DASHDIETEXP, ) (wrattbr2 -> DASHDIETEXP, ) (wrattbr3 -> DASHDIETEXP, ) (wrattbr4 -> DASHDIETEXP, )(smoke1 -> DASHDIETEXP, ) (smoke9 -> DASHDIETEXP, )(currdrugs1 -> DASHDIETEXP, ) (currdrugs9 -> DASHDIETEXP, ) (bmi_C -> DASHDIETEXP, ) (SRHbr2 -> DASHDIETEXP, ) (SRHbr3 -> DASHDIETEXP, )(kcalw1w3mean_C -> DASHDIETEXP, ) (energystoresw1w3mean_C -> DASHDIETEXP, ) (invmillssem -> DASHDIETEXP, ) if finalsample==1 & race==1 , nocapslatent method(ml)

estat gof, stats(all)
estat teffects



//BELOW POVERTY//
sem (FCOSTEXP -> BPDIAW4 , ) (DASHDIETEXP -> BPDIAW4 , ) ( Agew1_C-> BPDIAW4 , ) (Agew3_C -> BPDIAW4 , ) (sex -> BPDIAW4 , ) (race -> BPDIAW4 , ) (edubr2 -> BPDIAW4 , ) (edubr3 -> BPDIAW4 , ) (employed1 -> BPDIAW4 , )   (wrattbr2
```

-> BPDIAW4 ,) (wrattbr3 -> BPDIAW4 ,) (wrattbr4 -> BPDIAW4 ,)(smoke1 -> BPDIAW4 ,) (smoke9 -> BPDIAW4 ,)(currdrugs1 -> BPDIAW4 ,) (currdrugs9 -> BPDIAW4 ,) (bmi_C -> BPDIAW4 ,) (SRHbr2 -> BPDIAW4 ,) (SRHbr3 -> BPDIAW4 ,)(kcalw1w3mean_C -> BPDIAW4 ,) (energystoresw1w3mean_C -> BPDIAW4 ,) (invmillssem -> BPDIAW4 ,) ///
(FCOSTEXP -> DASHDIETEXP,)(Agew1_C-> DASHDIETEXP,) (Agew3_C -> DASHDIETEXP,) (sex -> DASHDIETEXP,) (race -> DASHDIETEXP,) (edubr2 -> DASHDIETEXP,) (edubr3 -> DASHDIETEXP,) (employed1 -> DASHDIETEXP,) (wrattbr2 -> DASHDIETEXP,) (wrattbr3 -> DASHDIETEXP,) (wrattbr4 -> DASHDIETEXP,)(smoke1 -> DASHDIETEXP,) (smoke9 -> DASHDIETEXP,)(currdrugs1 -> DASHDIETEXP,) (currdrugs9 -> DASHDIETEXP,) (bmi_C -> DASHDIETEXP,) (SRHbr2 -> DASHDIETEXP,) (SRHbr3 -> DASHDIETEXP,)(kcalw1w3mean_C -> DASHDIETEXP,) (energystoresw1w3mean_C -> DASHDIETEXP,) (invmillssem -> DASHDIETEXP,) if finalsample==1 & pir==0 , nocapslatent method(ml)

estat gof, stats(all)
estat teffects

//ABOVE POVERTY//
sem (FCOSTEXP -> BPDIAW4 ,) (DASHDIETEXP -> BPDIAW4 ,) (Agew1_C-> BPDIAW4 ,) (Agew3_C -> BPDIAW4 ,) (sex -> BPDIAW4 ,) (race -> BPDIAW4 ,) (edubr2 -> BPDIAW4 ,) (edubr3 -> BPDIAW4 ,) (employed1 -> BPDIAW4 ,) (wrattbr2 -> BPDIAW4 ,) (wrattbr3 -> BPDIAW4 ,) (wrattbr4 -> BPDIAW4 ,)(smoke1 -> BPDIAW4 ,) (smoke9 -> BPDIAW4 ,)(currdrugs1 -> BPDIAW4 ,) (currdrugs9 -> BPDIAW4 ,) (bmi_C -> BPDIAW4 ,) (SRHbr2 -> BPDIAW4 ,) (SRHbr3 -> BPDIAW4 ,)(kcalw1w3mean_C -> BPDIAW4 ,) (energystoresw1w3mean_C -> BPDIAW4 ,) (invmillssem -> BPDIAW4 ,) ///
(FCOSTEXP -> DASHDIETEXP,)(Agew1_C-> DASHDIETEXP,) (Agew3_C -> DASHDIETEXP,) (sex -> DASHDIETEXP,) (race -> DASHDIETEXP,) (edubr2 -> DASHDIETEXP,) (edubr3 -> DASHDIETEXP,) (employed1 -> DASHDIETEXP,) (wrattbr2 -> DASHDIETEXP,) (wrattbr3 -> DASHDIETEXP,) (wrattbr4 -> DASHDIETEXP,)(smoke1 -> DASHDIETEXP,) (smoke9 -> DASHDIETEXP,)(currdrugs1 -> DASHDIETEXP,) (currdrugs9 -> DASHDIETEXP,) (bmi_C -> DASHDIETEXP,) (SRHbr2 -> DASHDIETEXP,) (SRHbr3 -> DASHDIETEXP,)(kcalw1w3mean_C -> DASHDIETEXP,) (energystoresw1w3mean_C -> DASHDIETEXP,) (invmillssem -> DASHDIETEXP,) if finalsample==1 & pir==1 , nocapslatent method(ml)

estat gof, stats(all)
estat teffects

****************************HR WAVE 4**
//MULTI-GROUP//

All
sem (FCOSTEXP -> HRW4,) (DASHDIETEXP -> HRW4,) (Agew1_C-> HRW4,) (Agew3_C -> HRW4,) (sex -> HRW4,) (race -> HRW4,) (pir -> HRW4,) (edubr2 -> HRW4,) (edubr3 -> HRW4,) (employed1 -> HRW4,) (wrattbr2 -> HRW4,) (wrattbr3 -> HRW4,) (wrattbr4 -> HRW4,)(smoke1 -> HRW4,) (smoke9 -> HRW4,)(currdrugs1 -> HRW4,) (currdrugs9 -> HRW4,) (bmi_C -> HRW4,) (SRHbr2 -> HRW4,) (SRHbr3 -> HRW4,)(kcalw1w3mean_C -> HRW4,) (energystoresw1w3mean_C -> HRW4,) (invmillssem -> HRW4,) ///
(FCOSTEXP -> DASHDIETEXP,)(Agew1_C-> DASHDIETEXP,) (Agew3_C -> DASHDIETEXP,) (sex -> DASHDIETEXP,) (race -> DASHDIETEXP,) (pir -> DASHDIETEXP,) (edubr2 -> DASHDIETEXP,) (edubr3 -> DASHDIETEXP,) (employed1 ->

DASHDIETEXP,) (wrattbr2 -> DASHDIETEXP,) (wrattbr3 -> DASHDIETEXP,) (wrattbr4 -> DASHDIETEXP,)(smoke1 -> DASHDIETEXP,) (smoke9 -> DASHDIETEXP,)(currdrugs1 -> DASHDIETEXP,) (currdrugs9 -> DASHDIETEXP,) (bmi_C -> DASHDIETEXP,) (SRHbr2 -> DASHDIETEXP,) (SRHbr3 -> DASHDIETEXP,)(kcalw1w3mean_C -> DASHDIETEXP,) (energystoresw1w3mean_C -> DASHDIETEXP,) (invmillssem -> DASHDIETEXP,) , nocapslatent method(ml) group(finalsample)

estat ginvariant

estat gof, stats(all)
estat teffects

By sex

sem (FCOSTEXP -> HRW4,) (DASHDIETEXP -> HRW4,) (Agew1_C-> HRW4,) (Agew3_C -> HRW4,) (race -> HRW4,) (pir -> HRW4,) (edubr2 -> HRW4,) (edubr3 -> HRW4,) (employed1 -> HRW4,) (wrattbr2 -> HRW4,) (wrattbr3 -> HRW4,) (wrattbr4 -> HRW4,)(smoke1 -> HRW4,) (smoke9 -> HRW4,)(currdrugs1 -> HRW4,) (currdrugs9 -> HRW4,) (bmi_C -> HRW4,) (SRHbr2 -> HRW4,) (SRHbr3 -> HRW4,)(kcalw1w3mean_C -> HRW4,) (energystoresw1w3mean_C -> HRW4,) (invmillssem -> HRW4,) ///
(FCOSTEXP -> DASHDIETEXP,)(Agew1_C-> DASHDIETEXP,) (Agew3_C -> DASHDIETEXP,) (race -> DASHDIETEXP,) (pir -> DASHDIETEXP,) (edubr2 -> DASHDIETEXP,) (edubr3 -> DASHDIETEXP,) (employed1 -> DASHDIETEXP,) (wrattbr2 -> DASHDIETEXP,) (wrattbr3 -> DASHDIETEXP,)(wrattbr4 -> DASHDIETEXP,) (smoke1 -> DASHDIETEXP,) (smoke9 -> DASHDIETEXP,)(currdrugs1 -> DASHDIETEXP,) (currdrugs9 -> DASHDIETEXP,) (bmi_C -> DASHDIETEXP,) (SRHbr2 -> DASHDIETEXP,) (SRHbr3 -> DASHDIETEXP,)(kcalw1w3mean_C -> DASHDIETEXP,) (energystoresw1w3mean_C -> DASHDIETEXP,) (invmillssem -> DASHDIETEXP,) if finalsample==1 , nocapslatent method(ml) group(sex)

estat ginvariant

By race

sem (FCOSTEXP -> HRW4,) (DASHDIETEXP -> HRW4,) (Agew1_C-> HRW4,) (Agew3_C -> HRW4,) (sex -> HRW4,) (pir -> HRW4,) (edubr2 -> HRW4,) (edubr3 -> HRW4,) (employed1 -> HRW4,) (wrattbr2 -> HRW4,) (wrattbr3 -> HRW4,) (wrattbr4 -> HRW4,)(smoke1 -> HRW4,) (smoke9 -> HRW4,)(currdrugs1 -> HRW4,) (currdrugs9 -> HRW4,) (bmi_C -> HRW4,) (SRHbr2 -> HRW4,) (SRHbr3 -> HRW4,)(kcalw1w3mean_C -> HRW4,) (energystoresw1w3mean_C -> HRW4,) (invmillssem -> HRW4,) ///
(FCOSTEXP -> DASHDIETEXP,)(Agew1_C-> DASHDIETEXP,) (Agew3_C -> DASHDIETEXP,) (sex -> DASHDIETEXP,) (pir -> DASHDIETEXP,) (edubr2 -> DASHDIETEXP,) (edubr3 -> DASHDIETEXP,) (employed1 -> DASHDIETEXP,) (wrattbr2 -> DASHDIETEXP,) (wrattbr3 -> DASHDIETEXP,) (wrattbr4 -> DASHDIETEXP,)(smoke1 -> DASHDIETEXP,) (smoke9 -> DASHDIETEXP,)(currdrugs1 -> DASHDIETEXP,) (currdrugs9 -> DASHDIETEXP,) (bmi_C -> DASHDIETEXP,) (SRHbr2 -> DASHDIETEXP,) (SRHbr3 -> DASHDIETEXP,)(kcalw1w3mean_C -> DASHDIETEXP,)

(energystoresw1w3mean_C -> DASHDIETEXP,) (invmillssem -> DASHDIETEXP,) if finalsample==1 , nocapslatent method(ml) group(race)

estat ginvariant

By pir

sem (FCOSTEXP -> HRW4,) (DASHDIETEXP -> HRW4,) (Agew1_C-> HRW4,) (Agew3_C -> HRW4,) (sex -> HRW4,) (race -> HRW4,) (edubr2 -> HRW4,) (edubr3 -> HRW4,) (employed1 -> HRW4,) (wrattbr2 -> HRW4,) (wrattbr3 -> HRW4,) (wrattbr4 -> HRW4,)(smoke1 -> HRW4,) (smoke9 -> HRW4,)(currdrugs1 -> HRW4,) (currdrugs9 -> HRW4,) (bmi_C -> HRW4,) (SRHbr2 -> HRW4,) (SRHbr3 -> HRW4,)(kcalw1w3mean_C -> HRW4,) (energystoresw1w3mean_C -> HRW4,) (invmillssem -> HRW4,) ///
(FCOSTEXP -> DASHDIETEXP,)(Agew1_C-> DASHDIETEXP,) (Agew3_C -> DASHDIETEXP,) (sex -> DASHDIETEXP,) (race -> DASHDIETEXP,) (edubr2 -> DASHDIETEXP,) (edubr3 -> DASHDIETEXP,) (employed1 -> DASHDIETEXP,) (wrattbr2 -> DASHDIETEXP,) (wrattbr3 -> DASHDIETEXP,) (wrattbr4 -> DASHDIETEXP,)(smoke1 -> DASHDIETEXP,) (smoke9 -> DASHDIETEXP,)(currdrugs1 -> DASHDIETEXP,) (currdrugs9 -> DASHDIETEXP,) (bmi_C -> DASHDIETEXP,) (SRHbr2 -> DASHDIETEXP,) (SRHbr3 -> DASHDIETEXP,)(kcalw1w3mean_C -> DASHDIETEXP,) (energystoresw1w3mean_C -> DASHDIETEXP,) (invmillssem -> DASHDIETEXP,) if finalsample==1 , nocapslatent method(ml) group(pir)

estat ginvariant

*********************STRATIFIED ANALYSIS********************************

//MEN//
sem (FCOSTEXP -> HRW4,) (DASHDIETEXP -> HRW4,) (Agew1_C-> HRW4,) (Agew3_C -> HRW4,) (race -> HRW4,) (pir -> HRW4,) (edubr2 -> HRW4,) (edubr3 -> HRW4,) (employed1 -> HRW4,) (wrattbr2 -> HRW4,) (wrattbr3 -> HRW4,) (wrattbr4 -> HRW4,)(smoke1 -> HRW4,) (smoke9 -> HRW4,)(currdrugs1 -> HRW4,) (currdrugs9 -> HRW4,) (bmi_C -> HRW4,) (SRHbr2 -> HRW4,) (SRHbr3 -> HRW4,)(kcalw1w3mean_C -> HRW4,) (energystoresw1w3mean_C -> HRW4,) (invmillssem -> HRW4,) ///
(FCOSTEXP -> DASHDIETEXP,)(Agew1_C-> DASHDIETEXP,) (Agew3_C -> DASHDIETEXP,) (race -> DASHDIETEXP,) (pir -> DASHDIETEXP,) (edubr2 -> DASHDIETEXP,) (edubr3 -> DASHDIETEXP,) (employed1 -> DASHDIETEXP,) (wrattbr2 -> DASHDIETEXP,) (wrattbr3 -> DASHDIETEXP,)(wrattbr4 -> DASHDIETEXP,) (smoke1 -> DASHDIETEXP,) (smoke9 -> DASHDIETEXP,)(currdrugs1 -> DASHDIETEXP,) (currdrugs9 -> DASHDIETEXP,) (bmi_C -> DASHDIETEXP,) (SRHbr2 -> DASHDIETEXP,) (SRHbr3 -> DASHDIETEXP,)(kcalw1w3mean_C -> DASHDIETEXP,) (energystoresw1w3mean_C -> DASHDIETEXP,) (invmillssem -> DASHDIETEXP,) if finalsample==1 & sex==1 , nocapslatent method(ml)

estat gof, stats(all)
estat teffects

//WOMEN//

sem (FCOSTEXP -> HRW4,) (DASHDIETEXP -> HRW4,) (Agew1_C-> HRW4,) (Agew3_C -> HRW4,) (race -> HRW4,) (pir -> HRW4,) (edubr2 -> HRW4,) (edubr3 -> HRW4,) (employed1 -> HRW4,) (wrattbr2 -> HRW4,) (wrattbr3 -> HRW4,) (wrattbr4 -> HRW4,)(smoke1 -> HRW4,) (smoke9 -> HRW4,)(currdrugs1 -> HRW4,) (currdrugs9 -> HRW4,) (bmi_C -> HRW4,) (SRHbr2 -> HRW4,) (SRHbr3 -> HRW4,)(kcalw1w3mean_C -> HRW4,) (energystoresw1w3mean_C -> HRW4,) (invmillssem -> HRW4,) ///
(FCOSTEXP -> DASHDIETEXP,)(Agew1_C-> DASHDIETEXP,) (Agew3_C -> DASHDIETEXP,) (race -> DASHDIETEXP,) (pir -> DASHDIETEXP,) (edubr2 -> DASHDIETEXP,) (edubr3 -> DASHDIETEXP,) (employed1 -> DASHDIETEXP,) (wrattbr2 -> DASHDIETEXP,) (wrattbr3 -> DASHDIETEXP,) (wrattbr4 -> DASHDIETEXP,)(smoke1 -> DASHDIETEXP,) (smoke9 -> DASHDIETEXP,)(currdrugs1 -> DASHDIETEXP,) (currdrugs9 -> DASHDIETEXP,) (bmi_C -> DASHDIETEXP,) (SRHbr2 -> DASHDIETEXP,) (SRHbr3 -> DASHDIETEXP,)(kcalw1w3mean_C -> DASHDIETEXP,) (energystoresw1w3mean_C -> DASHDIETEXP,) (invmillssem -> DASHDIETEXP,) if finalsample==1 & sex==0 , nocapslatent method(ml)

estat gof, stats(all)
estat teffects

//WHITES//
sem (FCOSTEXP -> HRW4,) (DASHDIETEXP -> HRW4,) (Agew1_C-> HRW4,) (Agew3_C -> HRW4,) (sex -> HRW4,) (pir -> HRW4,) (edubr2 -> HRW4,) (edubr3 -> HRW4,) (employed1 -> HRW4,) (wrattbr2 -> HRW4,) (wrattbr3 -> HRW4,)(wrattbr4 -> HRW4,) (smoke1 -> HRW4,) (smoke9 -> HRW4,)(currdrugs1 -> HRW4,) (currdrugs9 -> HRW4,) (bmi_C -> HRW4,) (SRHbr2 -> HRW4,) (SRHbr3 -> HRW4,)(kcalw1w3mean_C -> HRW4,) (energystoresw1w3mean_C -> HRW4,) (invmillssem -> HRW4,) ///
(FCOSTEXP -> DASHDIETEXP,)(Agew1_C-> DASHDIETEXP,) (Agew3_C -> DASHDIETEXP,) (sex -> DASHDIETEXP,) (pir -> DASHDIETEXP,) (edubr2 -> DASHDIETEXP,) (edubr3 -> DASHDIETEXP,) (employed1 -> DASHDIETEXP,) (wrattbr2 -> DASHDIETEXP,) (wrattbr3 -> DASHDIETEXP,) (wrattbr4 -> DASHDIETEXP,)(smoke1 -> DASHDIETEXP,) (smoke9 -> DASHDIETEXP,)(currdrugs1 -> DASHDIETEXP,) (currdrugs9 -> DASHDIETEXP,) (bmi_C -> DASHDIETEXP,) (SRHbr2 -> DASHDIETEXP,) (SRHbr3 -> DASHDIETEXP,)(kcalw1w3mean_C -> DASHDIETEXP,) (energystoresw1w3mean_C -> DASHDIETEXP,) (invmillssem -> DASHDIETEXP,) if finalsample==1 & race==0 , nocapslatent method(ml)

estat gof, stats(all)
estat teffects

//African-Americans//
sem (FCOSTEXP -> HRW4,) (DASHDIETEXP -> HRW4,) (Agew1_C-> HRW4,) (Agew3_C -> HRW4,) (sex -> HRW4,) (pir -> HRW4,) (edubr2 -> HRW4,) (edubr3 -> HRW4,) (employed1 -> HRW4,) (wrattbr2 -> HRW4,) (wrattbr3 -> HRW4,) (wrattbr4 -> HRW4,)(smoke1 -> HRW4,) (smoke9 -> HRW4,)(currdrugs1 -> HRW4,) (currdrugs9 -> HRW4,) (bmi_C -> HRW4,) (SRHbr2 -> HRW4,) (SRHbr3 -> HRW4,)(kcalw1w3mean_C -> HRW4,) (energystoresw1w3mean_C -> HRW4,) (invmillssem -> HRW4,) ///
(FCOSTEXP -> DASHDIETEXP,)(Agew1_C-> DASHDIETEXP,) (Agew3_C -> DASHDIETEXP,) (sex -> DASHDIETEXP,) (pir -> DASHDIETEXP,) (edubr2 -> DASHDIETEXP,) (edubr3 -> DASHDIETEXP,) (employed1 -> DASHDIETEXP,) (wrattbr2 -> DASHDIETEXP,) (wrattbr3 -> DASHDIETEXP,) (wrattbr4 -> DASHDIETEXP,)(smoke1 -> DASHDIETEXP,)

(smoke9 -> DASHDIETEXP,)(currdrugs1 -> DASHDIETEXP,) (currdrugs9 -> DASHDIETEXP,) (bmi_C -> DASHDIETEXP,) (SRHbr2 -> DASHDIETEXP,) (SRHbr3 -> DASHDIETEXP,)(kcalw1w3mean_C -> DASHDIETEXP,) (energystoresw1w3mean_C -> DASHDIETEXP,) (invmillssem -> DASHDIETEXP,) if finalsample==1 & race==1 , nocapslatent method(ml)

estat gof, stats(all)
estat teffects

//BELOW POVERTY//
sem (FCOSTEXP -> HRW4,) (DASHDIETEXP -> HRW4,) (Agew1_C-> HRW4,) (Agew3_C -> HRW4,) (sex -> HRW4,) (race -> HRW4,) (edubr2 -> HRW4,) (edubr3 -> HRW4,) (employed1 -> HRW4,) (wrattbr2 -> HRW4,) (wrattbr3 -> HRW4,) (wrattbr4 -> HRW4,)(smoke1 -> HRW4,) (smoke9 -> HRW4,)(currdrugs1 -> HRW4,) (currdrugs9 -> HRW4,) (bmi_C -> HRW4,) (SRHbr2 -> HRW4,) (SRHbr3 -> HRW4,)(kcalw1w3mean_C -> HRW4,) (energystoresw1w3mean_C -> HRW4,) (invmillssem -> HRW4,) ///
(FCOSTEXP -> DASHDIETEXP,)(Agew1_C-> DASHDIETEXP,) (Agew3_C -> DASHDIETEXP,) (sex -> DASHDIETEXP,) (race -> DASHDIETEXP,) (edubr2 -> DASHDIETEXP,) (edubr3 -> DASHDIETEXP,) (employed1 -> DASHDIETEXP,) (wrattbr2 -> DASHDIETEXP,) (wrattbr3 -> DASHDIETEXP,) (wrattbr4 -> DASHDIETEXP,)(smoke1 -> DASHDIETEXP,) (smoke9 -> DASHDIETEXP,)(currdrugs1 -> DASHDIETEXP,) (currdrugs9 -> DASHDIETEXP,) (bmi_C -> DASHDIETEXP,) (SRHbr2 -> DASHDIETEXP,) (SRHbr3 -> DASHDIETEXP,)(kcalw1w3mean_C -> DASHDIETEXP,) (energystoresw1w3mean_C -> DASHDIETEXP,) (invmillssem -> DASHDIETEXP,) if finalsample==1 & pir==0 , nocapslatent method(ml)

estat gof, stats(all)
estat teffects

//ABOVE POVERTY//
sem (FCOSTEXP -> HRW4,) (DASHDIETEXP -> HRW4,) (Agew1_C-> HRW4,) (Agew3_C -> HRW4,) (sex -> HRW4,) (race -> HRW4,) (edubr2 -> HRW4,) (edubr3 -> HRW4,) (employed1 -> HRW4,) (wrattbr2 -> HRW4,) (wrattbr3 -> HRW4,) (wrattbr4 -> HRW4,)(smoke1 -> HRW4,) (smoke9 -> HRW4,)(currdrugs1 -> HRW4,) (currdrugs9 -> HRW4,) (bmi_C -> HRW4,) (SRHbr2 -> HRW4,) (SRHbr3 -> HRW4,)(kcalw1w3mean_C -> HRW4,) (energystoresw1w3mean_C -> HRW4,) (invmillssem -> HRW4,) ///
(FCOSTEXP -> DASHDIETEXP,)(Agew1_C-> DASHDIETEXP,) (Agew3_C -> DASHDIETEXP,) (sex -> DASHDIETEXP,) (race -> DASHDIETEXP,) (edubr2 -> DASHDIETEXP,) (edubr3 -> DASHDIETEXP,) (employed1 -> DASHDIETEXP,) (wrattbr2 -> DASHDIETEXP,) (wrattbr3 -> DASHDIETEXP,) (wrattbr4 -> DASHDIETEXP,)(smoke1 -> DASHDIETEXP,) (smoke9 -> DASHDIETEXP,)(currdrugs1 -> DASHDIETEXP,) (currdrugs9 -> DASHDIETEXP,) (bmi_C -> DASHDIETEXP,) (SRHbr2 -> DASHDIETEXP,) (SRHbr3 -> DASHDIETEXP,)(kcalw1w3mean_C -> DASHDIETEXP,) (energystoresw1w3mean_C -> DASHDIETEXP,) (invmillssem -> DASHDIETEXP,) if finalsample==1 & pir==1 , nocapslatent method(ml)

estat gof, stats(all)
estat teffects
